# Network analysis of driver genes in human cancers

**DOI:** 10.3389/fbinf.2024.1365200

**Published:** 2024-07-08

**Authors:** Shruti S. Patil, Steven A. Roberts, Assefaw H. Gebremedhin

**Affiliations:** ^1^ School of Electrical Engineering and Computer Science, Washington State University, Pullman, WA, United States; ^2^ School of Molecular Biosciences, Washington State University, Pullman, WA, United States; ^3^ Department of Microbiology and Molecular Genetics, University of Vermont, Burlington, VT, United States; ^4^ UVM’s Larner College of Medicine, University of Vermont Cancer Center, Burlington, VT, United States

**Keywords:** network analysis, cancer genomics, driver genes, sequence similarity network, bipartite network

## Abstract

Cancer is a heterogeneous disease that results from genetic alteration of cell cycle and proliferation controls. Identifying mutations that drive cancer, understanding cancer type specificities, and delineating how driver mutations interact with each other to establish disease is vital for identifying therapeutic vulnerabilities. Such cancer specific patterns and gene co-occurrences can be identified by studying tumor genome sequences, and networks have proven effective in uncovering relationships between sequences. We present two network-based approaches to identify driver gene patterns among tumor samples. The first approach relies on analysis using the Directed Weighted All Nearest Neighbors (DiWANN) model, which is a variant of sequence similarity network, and the second approach uses bipartite network analysis. A data reduction framework was implemented to extract the minimal relevant information for the sequence similarity network analysis, where a transformed reference sequence is generated for constructing the driver gene network. This data reduction process combined with the efficiency of the DiWANN network model, greatly lowered the computational cost (in terms of execution time and memory usage) of generating the networks enabling us to work at a much larger scale than previously possible. The DiWANN network helped us identify cancer types in which samples were more closely connected to each other suggesting they are less heterogeneous and potentially susceptible to a common drug. The bipartite network analysis provided insight into gene associations and co-occurrences. We identified genes that were broadly mutated in multiple cancer types and mutations exclusive to only a few. Additionally, weighted one-mode gene projections of the bipartite networks revealed a pattern of occurrence of driver genes in different cancers. Our study demonstrates that network-based approaches can be an effective tool in cancer genomics. The analysis identifies co-occurring and exclusive driver genes and mutations for specific cancer types, providing a better understanding of the driver genes that lead to tumor initiation and evolution.

## 1 Introduction

Cancer is caused by genetic alterations in cells that affect growth regulatory genes. The application of next-generation sequencing technologies to characterize tumor genomes has greatly expanded the number of known cancer mutations that contribute to disease progression and may be therapeutic targets (Goodwin et al., 2016). In the past decade, hundreds of sequencing efforts have been made, including large scale efforts led by the International Cancer Genome Consortium (ICGC) (Hudson et al., 2010) and The Cancer Genome Atlas (Weinstein et al., 2013). Despite the vast amount of sequencing data available, understanding how different cancers develop at a mutation level has been difficult to ascertain. This difficulty largely originates from the large number of mutations accumulated in each tumor, the vast majority of which are not involved in driving tumorigenesis (termed passenger mutations) (Vogelstein et al., 2013). One of the main goals of cancer genomics is to identify and study gene mutations that actively drive cancer progression. These mutations give us insight into why the tumor developed and how it might be treated. Cancer driver mutations occur in two classes of genes--oncogenes and tumor suppressors—that function as positive and negative growth regulators, respectively. However, not all mutations occurring in oncogenes and tumor suppressor genes play a role in carcinogenesis (Bányai et al., 2021). Therefore, most driver mutations are identified through their recurrence in multiple tumor genomes.

Pan-cancer analyses of driver mutations have additional complexity in that the number of coding mutations in tumors varies depending on the cancer type, and that some driver mutations occur more frequently or exclusively in specific cancer types (van de Haar et al., 2019). The genetic basis of this heterogeneity is mostly due to the differences in time and intensity of exposure to mutational processes (Martínez-Jiménez et al., 2020). Notable among all tumors that have high mutation rates are skin and lung tumors, which contain almost 200 nonsynonymous mutations per tumor (Vogelstein et al., 2013; Hoadley et al., 2018). Rather than studying individual cancer types, studying multiple cancers together can be advantageous in elucidating common principles and patterns in cancer. A better understanding of how common different mutations and driver genes are in cancer patients could also help in prioritizing genetic alterations and thus benefit drug development. Many clinical trials employ a basket-trial format (Park et al., 2020), which involves treating mutated forms of a protein the same way across different types of cancer. This characterization of overall occurrence of mutations in targetable genes can help with drug development as well as design of personalized medicine clinical trials (Mendiratta et al., 2021).

Several studies have used computational and statistical approaches to identify driver genes and understand their significance in cancer (Cheng et al., 2016; Bailey et al., 2018). Among these approaches, network-based approaches greatly increase the precision of identifying cancer genes and their role in cancer (Ozturk et al., 2018; Oulas et al., 2019; Song et al., 2019). Network analysis of human diseases can be employed in various biological and clinical applications. For instance, when genes are represented as networks, complex patterns of gene associations can be found. Network analysis metrics and tools can then be used to prioritize genes for therapeutic targets and identify crucial diseases associated with them (Ramadan et al., 2016; Shah and Braun, 2019). Disease networks provide gene-disease associations (Goh et al., 2007), which, in turn, could help identify better targets for drug development and drug repurposing (Barabási et al., 2011; García del Valle et al., 2019). Though many studies have grown our understanding of genes and their role in cancer, most cover only a few cancer types. Additionally, pan cancer analyses of genomic mutations are computationally expensive. To further expand our understanding of cancer biology, it is crucial to study cancer genes across all cancers and within each cancer type using a computationally efficient approach.

Since driver mutations cause cancer (Balmain, 2020), we focus our study on recurrent mutations in known cancer-associated genes. We construct a sequence similarity network (SSN) with “transformed” sequences generated via a data reduction preprocessing step. SSNs are networks in which nodes are sequences and edges show the distance (typically, edit distance) between a pair of sequences, which shows the extent of their dissimilarity. In this work, we have used a variant of SSN called the Directed Weighted All Nearest Neighbors (DiWANN) network (Catanese et al., 2018), which connects every node (sequence) via a directed edge to its “nearest neighbor”–the sequence that is the closest to it in terms of edit distance–among the set of sequences considered. If multiple sequences are tied as having the same edit distance from a given “source” sequence, directed edges are added in the DiWANN model from the source sequence to all the target sequences (hence the phrase All Nearest Neighbors). The weights on edges in the DiWANN model are the edit distances.

The DiWANN model, in essence, represents the “backbone” of the similarity relationship among sequences of interest. It is much sparser than a typical threshold-based sequence similarity network and is yet amenable to meaningful analysis, including cluster analysis and centrality analysis, as previous studies have shown (Catanese et al., 2018; Patil et al., 2022). The DiWANN network model uses an efficient algorithm that incorporates several pruning and optimization strategies to construct the SSN. The algorithm is found to be much faster to execute than an all-to-all distance matrix computation that is commonly used to construct an SSN. In this study, we further reduce the computational complexity of the DiWANN network-based analysis by employing a data reduction step, which drastically reduces sequence length.

Complementing our analysis based on the DiWANN model, we also employ in this study a bipartite network analysis. The latter was performed for two purposes: to identify links between genes and tumor samples and to identify gene associations pertinent to different cancer types (Venkatraman et al., 2021). Bipartite networks represent interactions between two sets of nodes where the connections run across the two sets but not within the sets. Such networks can, for instance, be used to study gene disease associations. Additionally, bipartite graphs can be converted into one-mode projections (Network Science by Albert-László Barabási, 2023) for analysis focused on one of the sets. These projections are composed of nodes from one set of the bipartite network and the edges represent interconnections via connections to the nodes of the other set. Our study, in sum, shows how network-based approaches, namely, SSNs and bipartite networks, can effectively be used in cancer biology.

## 2 Results

### 2.1 Mutation data

We obtained a list of single-nucleotide variants (SNVs) and aggregated mutation information [in mutation annotation format (maf)] from the Pan-Cancer Analysis of Whole Genomes (PCAWG) in the ICGC data portal (Campbell et al., 2020). The maf file (DCC Data Releases | ICGC Data Portal, 2023) contained over 23 million mutations in tumors from 1,830 donors and 25 cancer types. To focus our network analysis on mutations likely contributing to disease, we filtered the data using two steps: (i) identifying variant classes that are likely to change protein function and (ii) assessing mutations in genes that occur in significant frequency. Most of the mutations in the ICGC dataset were classified as “inter-genic region” which we excluded along with Intron, 5’ Flank, and lincRNA variants as non-coding drivers are rare compared to coding regions (Rheinbay et al., 2020), and they are less likely to be functionally important (Brown et al., 2019). This filtering resulted in a dataset of 1.2 million mutations from 1,830 donors and 25 cancer types.

The second filtering step involved two different filtering approaches based on whether the gene was present in the Catalogue of Somatic Mutations in Cancer (COSMIC) Cancer Gene Census (CGC) catalogue (Sondka et al., 2018). The COSMIC CGC catalogue consists of 714 documented genes and our dataset included 708 of them. The first approach applied to COSMIC CGC genes involved keeping only mutations that were recurrent, which subsequently resulted in a dataset comprising 293 driver genes, potentially eliminating random passenger mutations for this study. The second filtering approach was applied to genes that were not present in COSMIC CGC. Gene mutations that represented the top 4% of frequency within each cancer type were retained. This threshold was chosen aiming to ensure distributed and normalized selection of genes for all cancers. This resulted in an additional 972 genes, making 1,264 genes in total.

**FIGURE 1:**
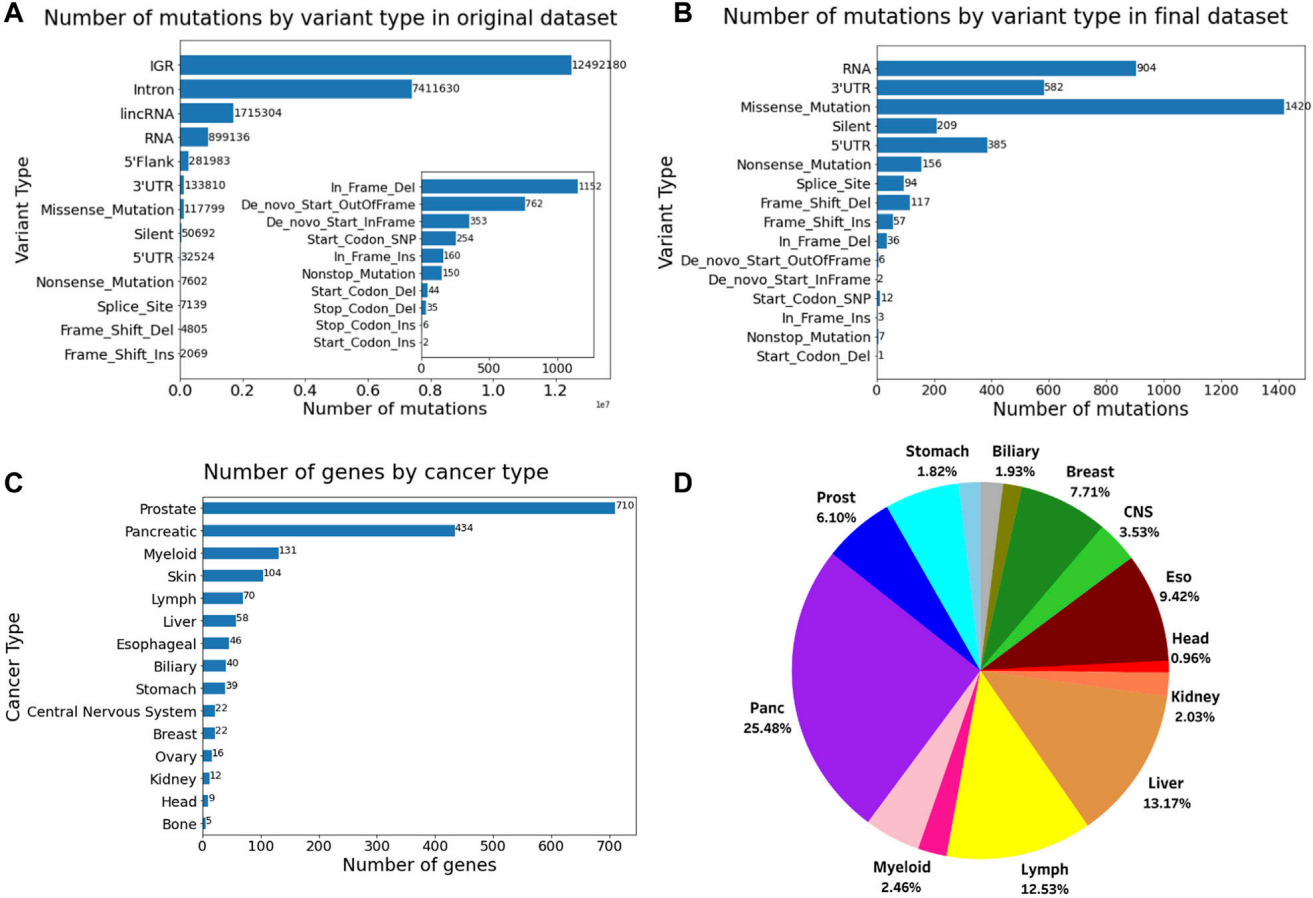
Number of mutations by variant type **(A)** before filtering and **(B)** after filtering. **(C)** Number of genes and their role in cancer. **(D)** Pie chart showing the proportion of different cancer types in the final dataset comprising 934 samples. The inset in Figure A shows the number of mutations for the variants having the 10 lowest frequencies.

The number of variant types of the dataset before and after filtering, the number of the final genes for each role in cancer, and the proportion of different cancer types in this dataset is shown in Figure 1. The final dataset consists of 1,264 genes and 3,900 mutations from 934 donor samples across 15 distinct tissues or cancer types. The final reduced mutational list for each donor sample has been provided in Supplementary Table S1. The data filtering process reduced the computational cost while making sure to maintain the relevant information from the original dataset.

### 2.2 Transformed sequence

**FIGURE 2:**
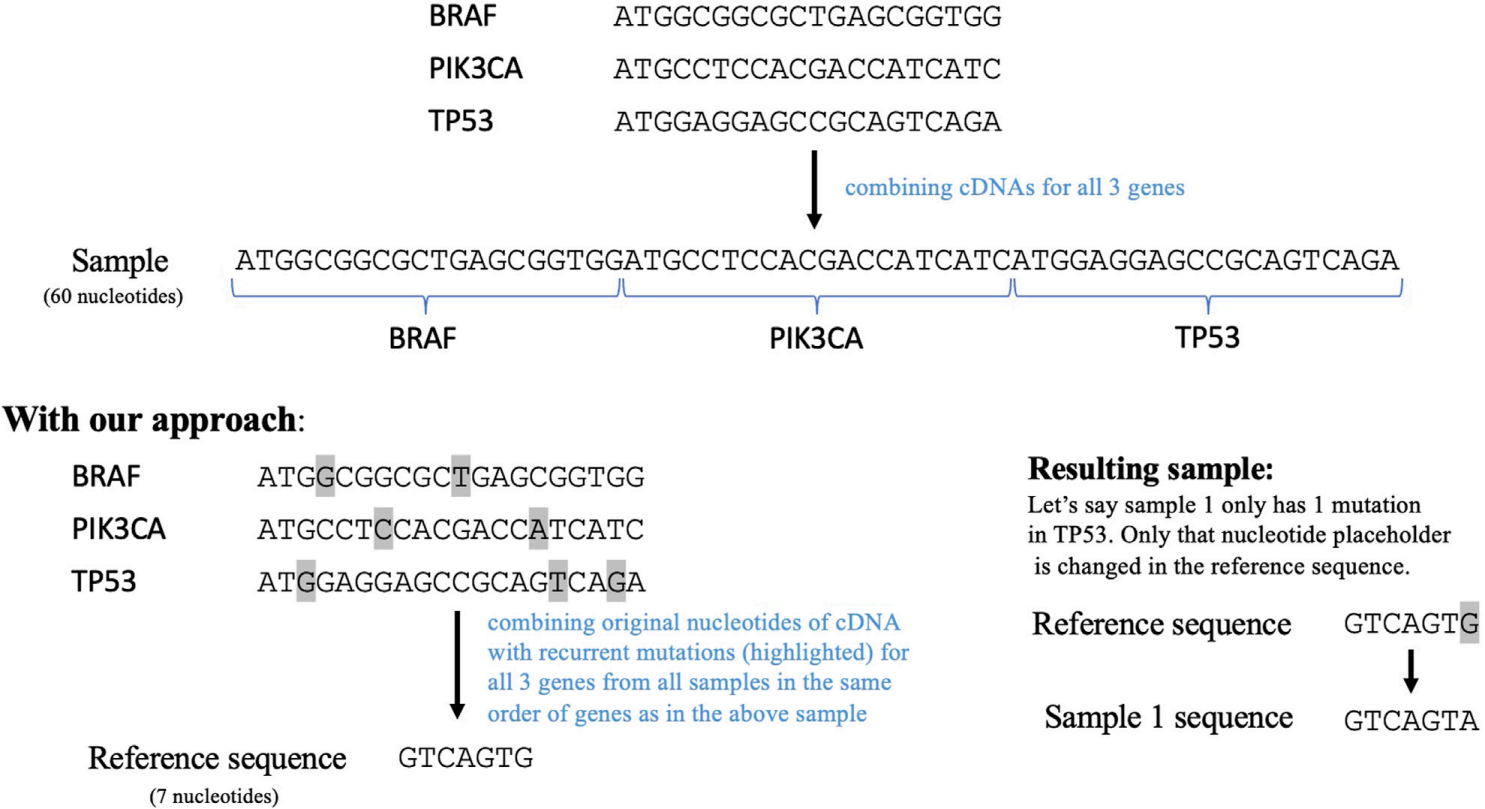
The top part shows how cDNAs for the selected genes can be combined to form a sequence for each sample and the bottom part shows our approach that combines only the regions with recurrent mutations in the selected genes. The transformed sample sequence generated using our approach is much shorter in length than the original sequence. This reference sequence is then mutated according to the mutations in a particular sample. Note: the figure only demonstrates the approach and thus does not contain the entire cDNAs for the given genes.

To employ a sequence similarity network analysis on this data, we next needed to create a sequence for each tumor containing all the relevant mutations. Therefore, we implemented another data reduction step to enable efficient usage of a sequence similarity network. The conventional way of doing this is to combine the cDNA sequences of the 1,264 genes remaining in our dataset post filtering. This resulted in a very long sequence comprising more than 300,000 nucleotides. The computational complexity of creating a network using this sequence as a reference would be tremendously high. Thus, we transformed this sequence to only include the nucleotides that have mutated in the previously filtered data. This transformed sequence contained the original nucleotides for all the recurrent mutations in each gene from all the samples, as shown in Figure 2. This serves as a reference sequence or wild type for all samples. This reference sequence would be mutated according to the mutations reported for each sample. The transformation step reduced the reference sequence to 2,169 nucleotides and the length of the mutated sequences for all the samples ranges from 2074 to 2,186 nucleotides. Importantly, the order in which this sequence was constructed will not impact subsequent DiWANN analyses.

**FIGURE 3:**
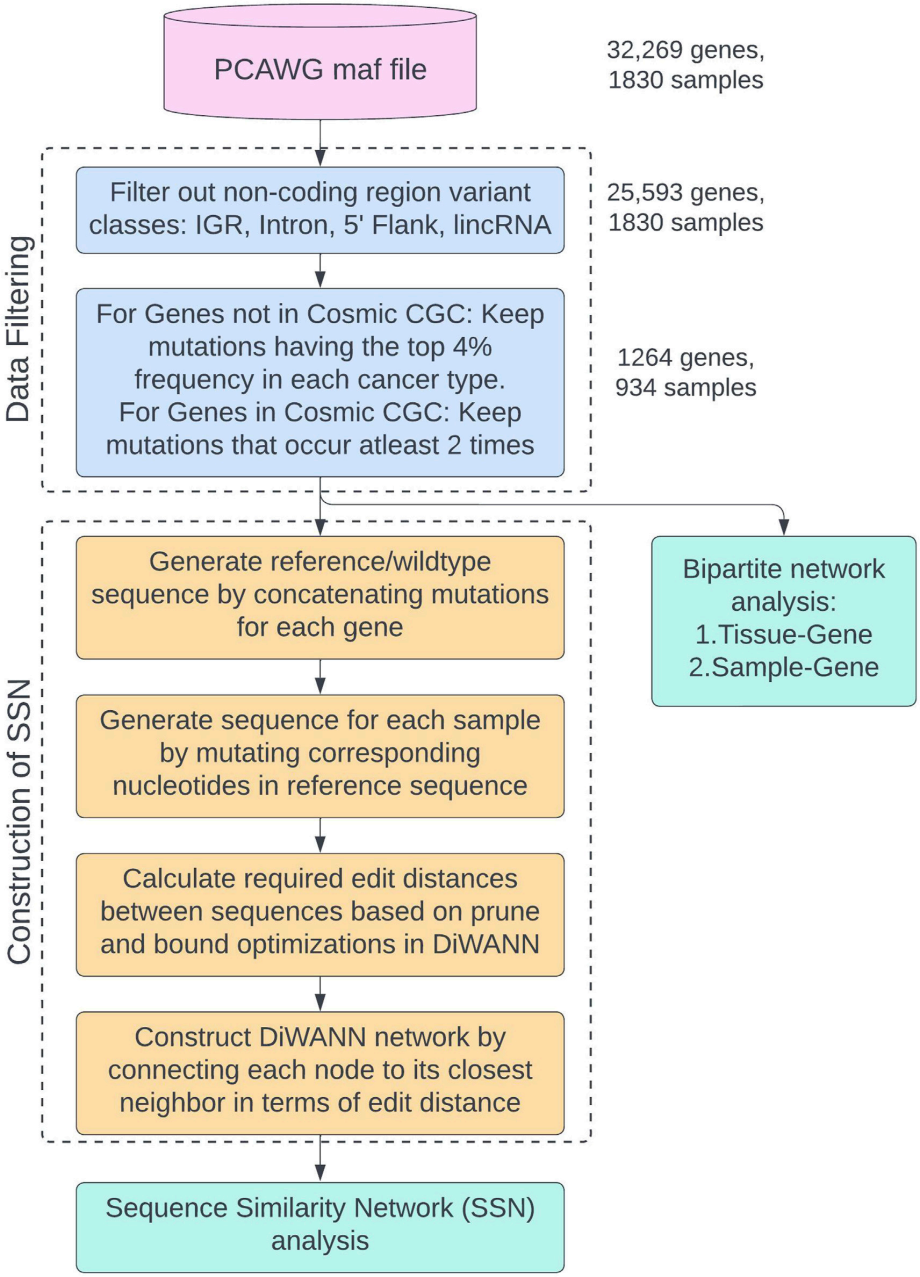
Flowchart showing the process of data filtering and preparing the data for network analyses. The different sub-processes are placed in named containers and colored differently. The number of genes and samples remaining after each filtering step are provided.

The complete process of filtering the original dataset, generating the transformed reference sequence, and preparing the data for network analyses is shown in the flowchart in Figure 3. This study uses the PCAWG maf file from ICGC and data filtering is performed as shown in the “Data Filtering” container. The final data is then used for two analyses: Sequence Similarity Network (SSN) analysis and bipartite network analysis. The process of generating the transformed sequences and constructing the SSN using DiWANN is shown in the “Construction of SSN” container in the flowchart. Two bipartite analyses are performed for tissue-gene bipartite network and sample-gene bipartite network.

### 2.3 Sequence similarity network analysis

**FIGURE 4:**
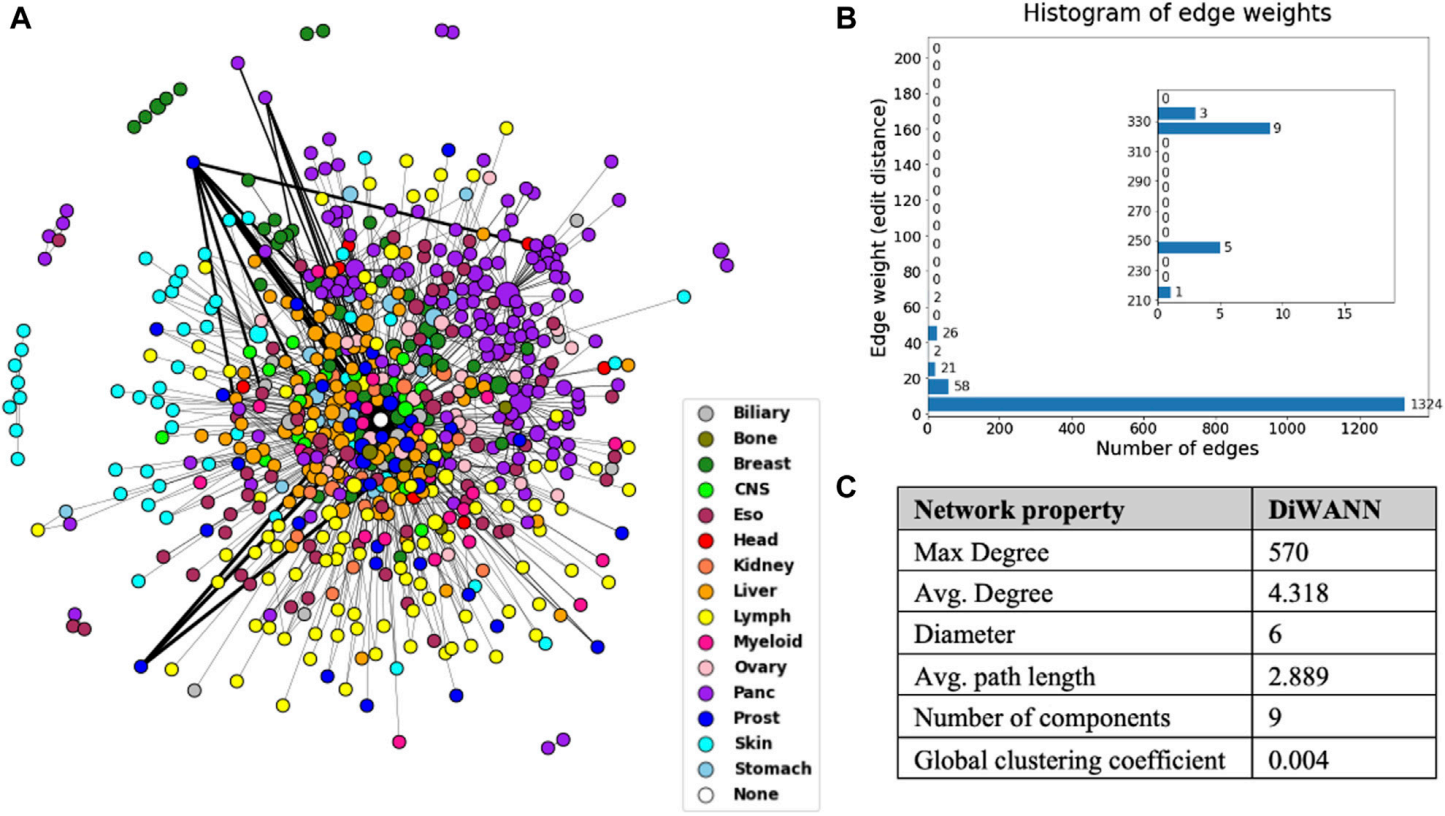
**(A)** DiWANN network consisting of 672 nodes colored by tissue, as shown in the legend. **(B)** Histogram of the edge weights of the network. **(C)** Structural network properties of the DiWANN network. The inset in Figure B shows histogram for edge weights more than 210.

A DiWANN network was built where the nodes are the mutated transformed sequences for samples, and the edges represent the edit distance between these sequences. A node representing the wild type sequence that would contain the unmutated transformed sequence was added to the network. Only unique sequences were used for the DiWANN network, which resulted in 672 nodes and 1,451 edges, as shown in Figure 4. The edge list for the network has been provided in Supplementary Table S2. The edge weight represents the edit distance (dissimilarity) and has a maximum value of 335 in this network, as shown in the histogram in Figure 4B. Over 70% of the edges have a weight of one indicating that majority of the sequences are just one edit distance away from each other. Since only unique sequences were considered for generating the network, there were some sequences that occurred more than once. These nodes were sized according to the number of occurrences of the sequence. The nodes were also colored by tumor tissue to aid in identifying patterns among different cancer types. There were about 16 instances where a node represented a sample sequence found in more than one cancer type, highlighting the uniqueness of each cancer. In rare instances of the same sequences occurring in multiple cancer types, the cancer type with the highest count of the sample sequence was considered for the node color. The structural properties of the network were also analyzed and are shown in Figure 4C. The maximum degree of the network is 570, however, the average degree of this network is just 4.318 as there are very few high degree nodes. The network has nine weak components which can also be seen in the plotted network. The global clustering coefficient is 0.004, indicating low clustering in the network.

**FIGURE 5:**
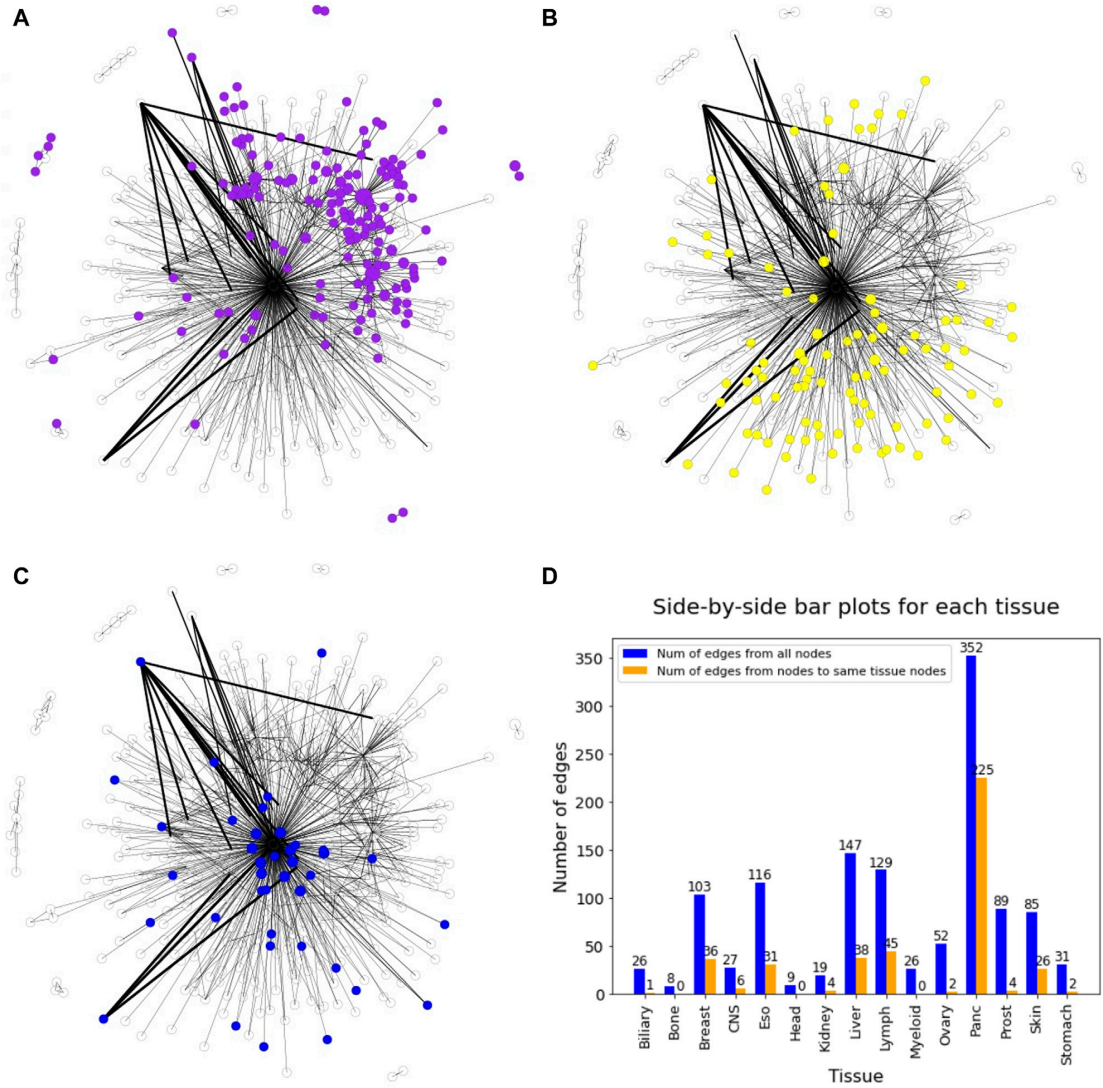
DiWANN networks consisting of 672 nodes with colored nodes for **(A)** Pancreatic cancer, **(B)** Lymph cancer and **(C)** Prostate cancer. **(D)** Barplot containing the number of edges from nodes of each tissue in blue and the number of edges to nodes of the same tissue in orange.

In general, the DiWANN network indicated significant heterogeneity among the tumors in the analysis. However, some cancer types were more closely associated than others. For instance, pancreatic and lymph cancers have a significant number of common edges between their nodes, indicating more similar routes of evolution between tumors in these cancer types. Prostate, esophageal, myeloid, and liver cancers just have a few common edges, that is, they only have a few common mutations in these cancers, suggesting high variability in the genetic alterations that cause these cancers. The subgraph of pancreatic nodes has a global clustering coefficient of 0.006, while for prostate cancer subgraph, the value is 0, indicating no tendency of the subgraph to cluster. These patterns are clearer when seen on individual views of the network for selected tissues as shown in Figure 5. The figure consists of the network nodes highlighted for pancreatic, lymph, and prostate tissue tumors. We can see that the pancreatic and lymph nodes are more connected amongst themselves than the prostate nodes. These patterns are quantitatively confirmed with the barplot in Figure 5 showing how many edges out of the total edges from nodes representing a tissue are connected to nodes of the same tissue. Networks with individual views for other tissue types have been shown in Supplementary Figures S1 and S2. Since there is a network for each cancer type, we did not need to select the highest count of the cancer type for the node color. If a sample sequence was found in more than one cancer type, the nodes were colored the assigned cancer type color in the respective network view.

The construction of the DiWANN network with the transformed sequences took 410 min, which is about 2/3rd the time taken for construction of an SSN with an optimal threshold. The threshold based SSN generated with an optimal threshold of edit distance as two had 672 nodes and 32,151 edges. The threshold was selected in such a way that the network had a balance between providing enough useful relationships and not becoming too dense.

**FIGURE 6:**
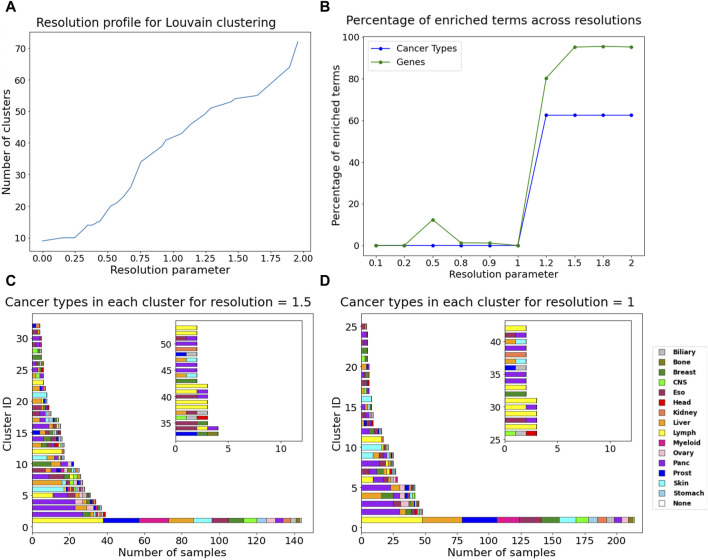
Community detection using Louvain algorithm **(A)** Resolution profile showing the cluster size at resolutions ranging from 0 to 2. **(B)** Percentage of enriched terms for all annotations across resolutions. Barplots containing the count of different cancer type samples in each cluster for **(C)** resolution = 1, **(D)** resolution = 1.5. The inset in plots C and D show cluster information for cluster IDs more than 32 for resolution = 1.5 and for cluster IDs more than 25 for resolution = 1.

Additionally, community detection on the DiWANN network revealed patterns among cancer types. Using the Louvain algorithm (resolution = 1), we obtained 44 clusters. With resolution >1, the network clusters were enriched with 6–10 cancer types and up to 997 causative genes based on the Fischer exact test. Resolutions equal to and greater than 1.5 had the highest number of enriched causative genes in their communities, as shown in Figure 6B. For values lower than the default resolution value (resolution = 1), only causative genes were enriched for resolution of 0.5. The cluster information for resolution 1, which was used as the background for the test has been provided in Supplementary Table S3. Overall, resolution value of 1.5 seems to be optimal in terms of cancer types and causative genes. Enriched genes tended to group together amongst themselves as the number of clusters reduced. The distribution of cancer types and cluster sizes are shown for resolution values one and 1.5 in Figure 6. Clusters for all the selected resolutions include one large cluster containing almost all cancer types.

Community detection using resolution value of 1.5 resulted in 53 clusters, among which one large cluster contained 143 nodes from all cancer types except head. About half of the clusters contain just one or two cancer types. The complete information about these clusters has been provided in Supplementary Table S4. Thirteen clusters were found exclusive to one cancer type. Pancreatic and lymph cancer were dominant or exclusively present in half the number of clusters. Skin and breast cancer also seem to dominate in the clusters they are present in or form their own clusters. Esophageal cancer was a part of many clusters, as it grouped with almost all other cancer types and did not have any pattern of its own. Among enriched cancer types were prostate, stomach, biliary, myeloid, CNS, ovary, kidney, bone, and head. They were more represented in the communities now due to smaller clusters, or they formed their own clusters. The largest cluster had half of the liver cancer samples and only 20% of the CTNNB1 gene mutations that were present in the largest cluster for resolution of 1. CTNNB1 is an important driver gene for liver cancer (Wang and Zhu, 2023) and hence their removal highlights the disease subtype.

Over 3/4ths of the clusters that are dominated by TP53 and KRAS mutations are dominated by pancreatic cancer. Clusters in which breast cancer was exclusive or dominant contained PIK3CA or GATA3 genes. Clusters dominated by Skin cancer were dominated by BRAF. BCL2 was dominant in all clusters that were exclusive to Lymph cancer. The largest cluster, which contains all the cancer types, is dominated by driver gene BCL2 as it mutates in 126 samples, indicating that BCL2 has a significant role in driving multiple cancer types. About 2/3rds of the clusters have a mean mutational load lower than 3, which indicates the number of mutations per sample.

### 2.4 Bipartite network analysis

To assess the cancer type-specificity of driver gene mutations and identify mutations that work in concert to promote disease, we analyzed two bipartite networks--a tissue-gene bipartite network and a sample-gene bipartite network--and their corresponding weighted one-mode projections. The tissue-gene bipartite network shows interactions between the tissue and gene set. A connection between a node in the tissue set to a node in the gene set means the gene has mutated in that tissue. The edge list for this bipartite network has been provided in Supplementary Table S5. Our dataset consists of 1,264 genes and 15 tissues from our dataset of 934 samples. Thus, we have 1,264 nodes in the gene set and 15 in the tissue set. This resulted in a bipartite network consisting of 1,279 nodes and 1718 edges.

**FIGURE 7:**
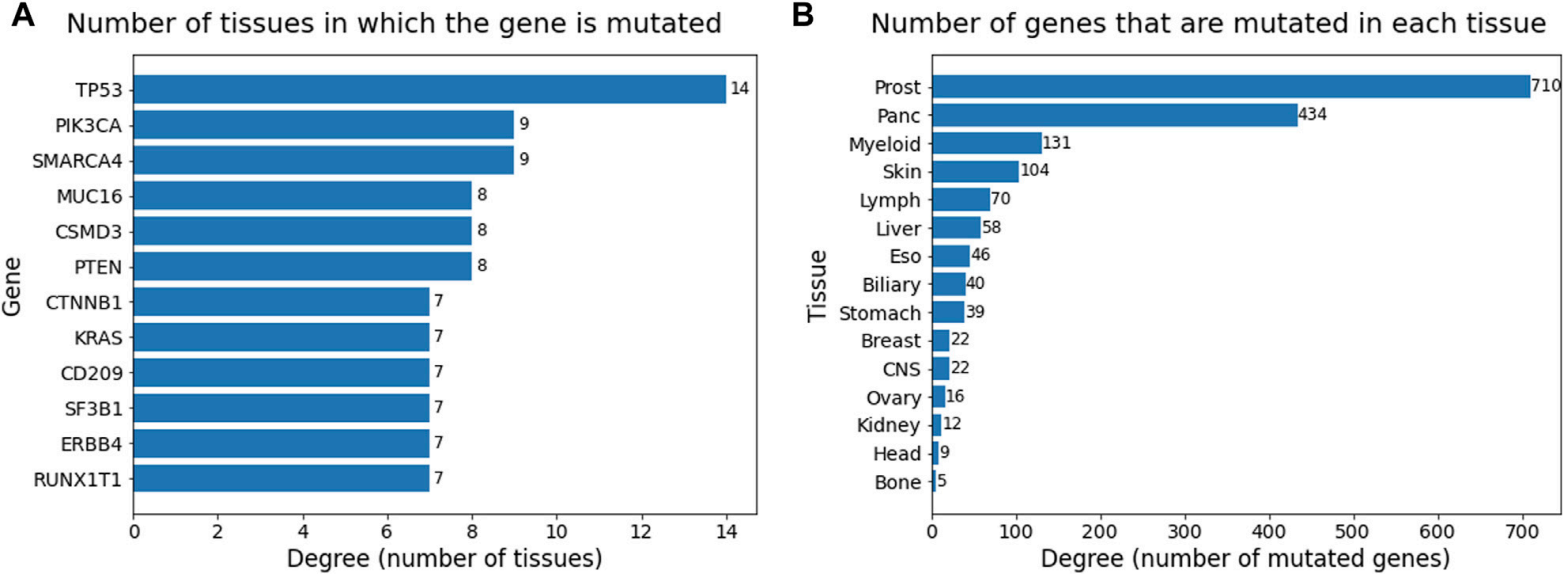
Barplots showing degrees (sorted in descending order) of **(A)** gene node set and **(B)** tissue node set in the bipartite network. Degree ≥ 7 considered for gene set and all 15 tissues were considered for tissue set.

Degree analysis of the two sets of the bipartite network provided information about the occurrence of mutations and genes across all the cancer types. The table in Figure 7A consists of degrees of nodes in the gene set that are greater than or equal to seven and are sorted in descending order. The degree in this table provides the count of tissues in which the gene has mutated. TP53 plays a role in almost all the cancer types present in this dataset, followed by PIK3CA and SMARCA4, which occur in nine cancer types. Genes PTEN, CSMD3, and MUC16 also occur in more than half the cancer types, suggesting their relevance as general cancer driver genes that impact multiple cancer types. The degree table for tissues in Figure 7B provides information on how many genes have been mutated in that cancer type. The degree values have been sorted in descending order. Prostate cancer is heavily mutated in driver genes, followed by pancreatic, myeloid, and skin cancer.

**FIGURE 8:**
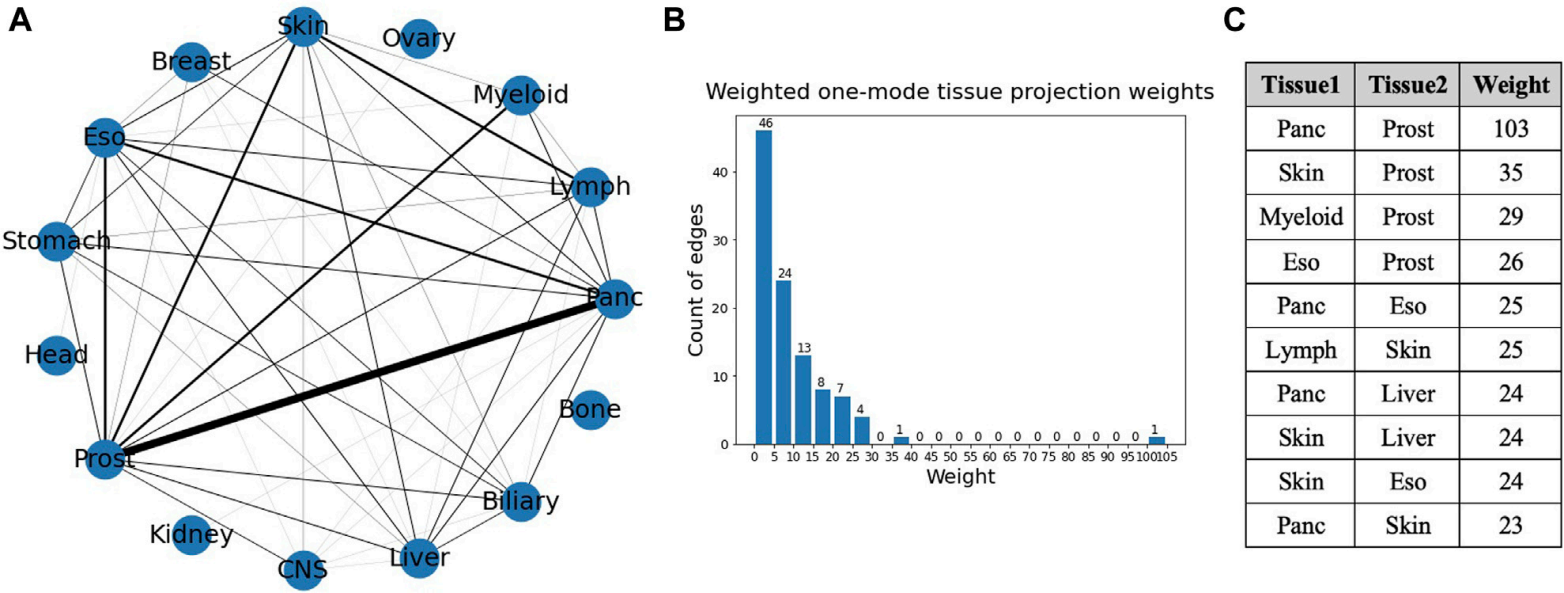
**(A)** Weighted one mode tissue projection from the tissue-gene bipartite network. **(B)** Histogram of the edge weights of the projection. **(C)** Table consisting of the top 10 edge weights in the projection.

**FIGURE 9:**
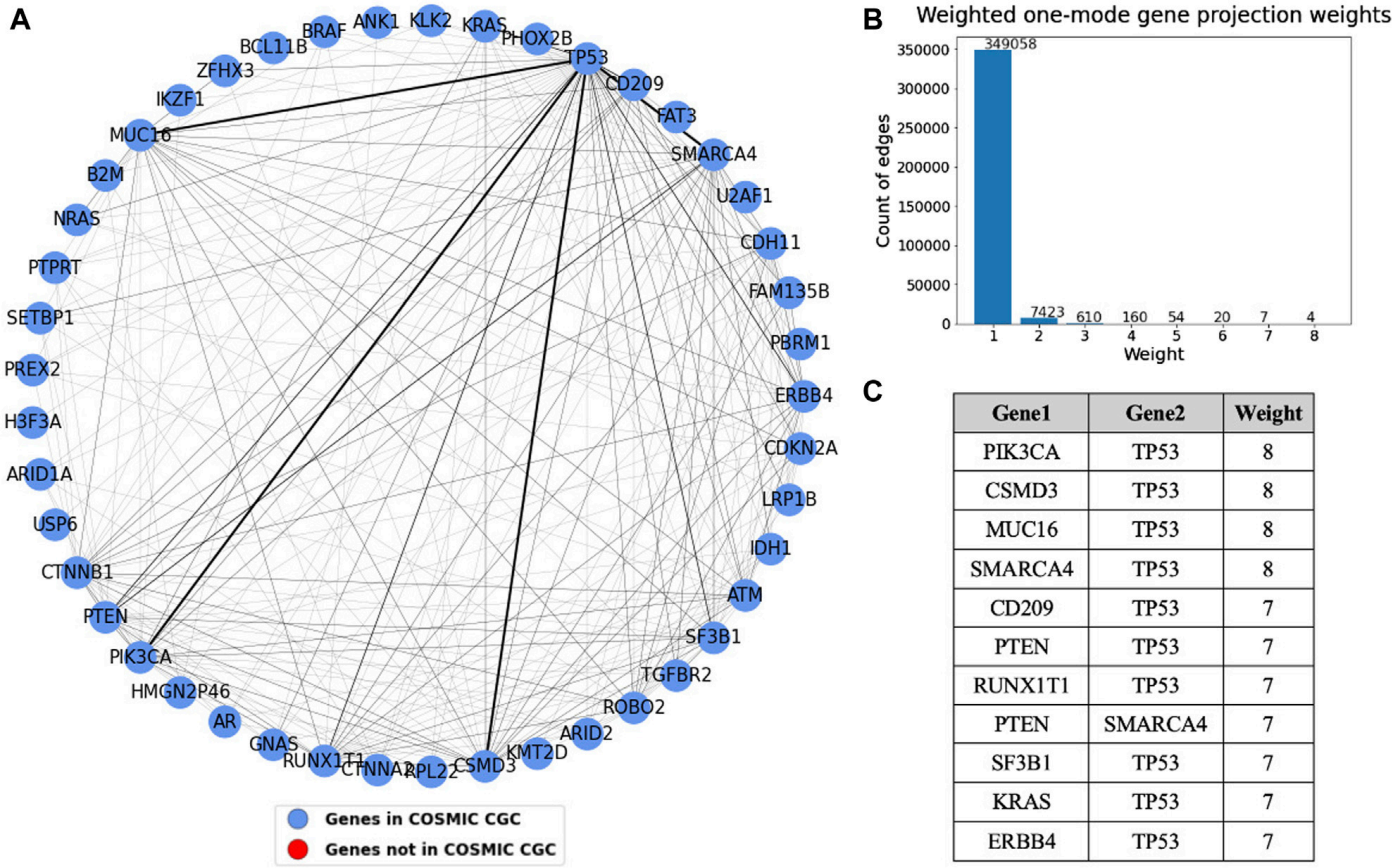
**(A)** Weighted one mode gene projection from the tissue gene bipartite network where the nodes are colored depending on whether the gene is present in COSMIC CGC, as shown in the legend. **(B)** Histogram of the edge weights of the projection. **(C)** Table consisting of the edge weights greater than 6 in the projection.

A weighted projection of each set of the bipartite graph provided us with more information on the similarity of different cancer types in terms of gene mutations. Figure 8 shows the projection on the tissue nodes, and Figure 9 shows the projection on the gene nodes. The edge weights in each case are a count of the different ways in which two nodes are connected in the bipartite graph, for example, the number of different genes through which two tissues are connected. The edge weight cutoff used for the projection in the tissue graph (Figure 8) is 6 as the number of edges drops drastically after that, and, in the visualizations, the widths of the edges were set proportional to the edge weights. The figure also shows a histogram of the weights of the projection and a table showing the top overlaps of genes. The maximum weight in this one mode projection is 103, which can be seen from the histogram, indicating an overlap of 103 genes between pancreatic and prostate cancer.

A one mode weighted gene projection graph was produced from the tissue-gene bipartite graph. A subset of this projection containing only the nodes that had an edge with weight more than or equal to three is shown in Figure 9. This projection helps us identify gene pairs that are relevant in multiple cancer types. For instance, TP53 mutated along with each of the four genes, PIK3CA, SMARCA4, CSMD3, and MUC16 in eight different tissues, though the projection does not provide us information about their co-occurrence. Similar to what was done in Figure 8, we set the edge widths in the visualization proportional to the edge weight. The table depicts gene pairs that have an overlap of at least seven tissues in the projection. We see that TP53 is overlapping with most of the genes in this table. The nodes in the projection are colored blue if they are present in COSMIC CGC, or else they are colored red. We see that the top gene pairs shown in this projection are all documented driver genes.

**FIGURE 10:**
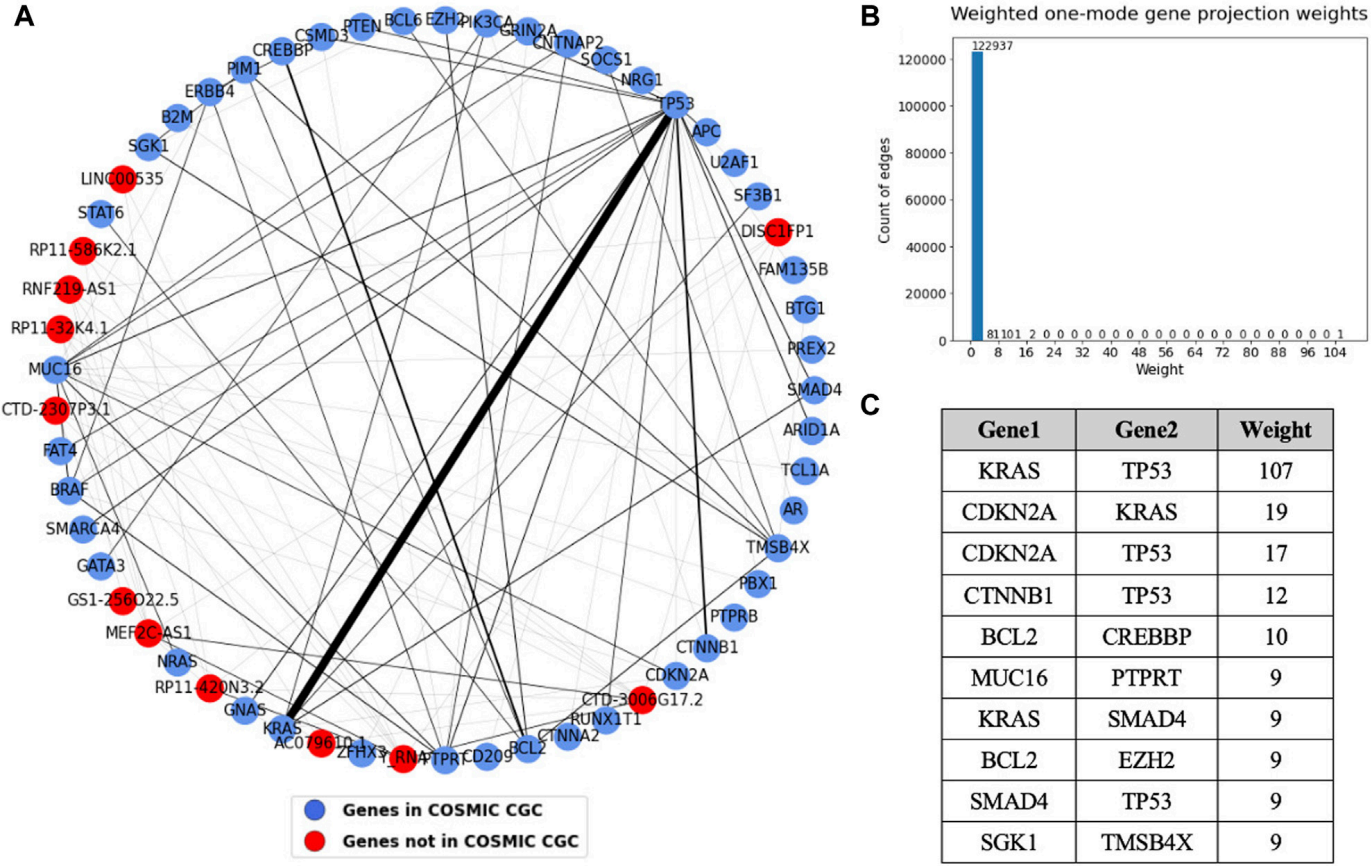
**(A)** Weighted one mode gene projection from the sample gene bipartite network where the nodes are colored depending on if the gene is present in COSMIC CGC, as shown in the legend. **(B)** Histogram of the edge weights of the projection. **(C)** Table consisting of the edge weights greater than 8 in the projection.

To get more precise information about the co-occurrence of driver genes in cancer, we generated a sample-gene bipartite graph and analyzed the weighted one mode weighted gene projection derived from it. The sample-gene bipartite network consists of 2,198 nodes and 3,086 edges. The edge list for this bipartite network has been provided in Supplementary Table S6. A subset of the gene projection containing only the nodes that had an edge with weight more than or equal to four is shown in Figure 10. The table shows the sorted edge weights greater than 8. The sample-gene bipartite network highlights genes that co-occur in the tumor samples. We see that KRAS and TP53 co-occur in many tumor samples, followed by three other pairs that occur in over 10 samples, which is also a significant number considering we analyzed 934 samples.

We know that these pairs co-occur in significant number of samples. However, we do not know if the pairs co-occur exclusively in specific cancer types or if they coexist in multiple cancer types. One mode gene projection from the sample gene bipartite network for each of the 15 cancer types helped us perform a deeper analysis and identify cancer specific patterns. The edge weights of these gene projections provided in Supplementary Table S7 give us the exact number of co-occurrences of genes in different cancer types. The table provides gene pairs that have a weight of more than two in the gene projections, that is, they co-occur in more than two samples.

**FIGURE 11:**
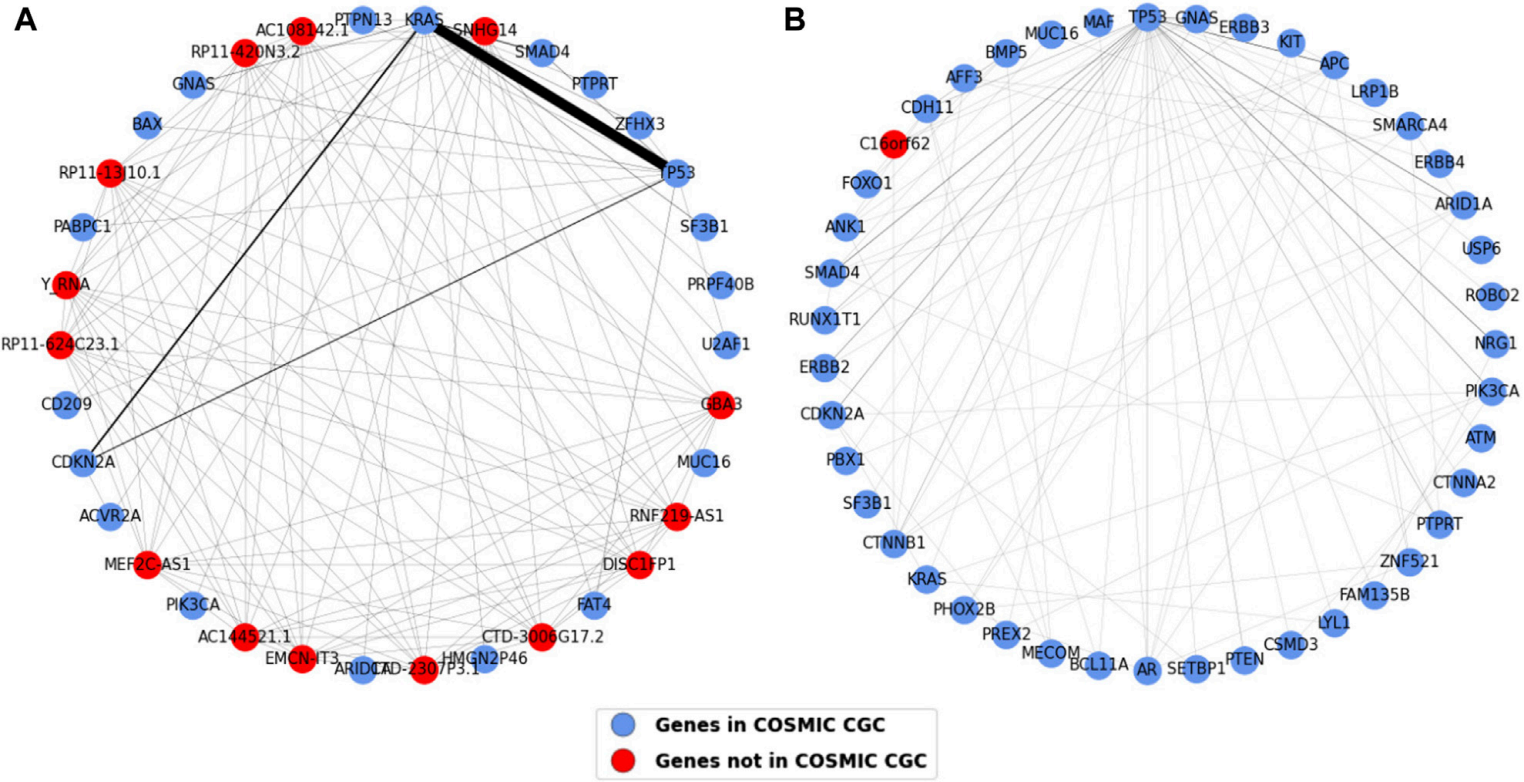
Weighted one mode gene projections for **(A)** Pancreatic and **(B)** Esophageal. The nodes are colored depending on if the gene is present in COSMIC CGC, as shown in the legend, and the edge width is 1/10th of edge weight. The pancreatic projection is a subgraph of the original projection and consists of nodes having an edge weight of two or more.

The one mode projections for pancreatic and esophageal cancer are shown in Figure 11. The most striking result observed in this analysis is that pancreatic cancer contains most of the TP53 and KRAS connections we saw in the gene projection for all the cancer types (i.e. 103 out of the total 107 shown in the weights table in Figure 10C). This strong association highlights a likely requirement for both inactivation of TP53 and oncogenic activation of KRAS in pancreatic cancer development. The second highest co-occurring pair in the common gene projection, CDKN2A and KRAS, also majorly occurs in pancreatic cancer. Most of the nodes in esophageal cancer are connected to TP53, with APC having the highest edge weight of five to TP53, followed by SMAD4, ARID1A, and NRG1, each of which have an edge weight of four to TP53.

The other cancer type projections have been provided in Supplementary Figures S3 and S4. We see myeloid and skin projections have many edges. Although there is a high number of edges in these cancer projections, there is no pair of genes with a high weight; in other words, no pair dominantly co-occurs. Prostate and myeloid cancer are dominated by genes that are not present in COSMIC CGC. Some genes co-occur with most other genes, including TP53 in biliary cancer, CTNNB1 in liver cancer, and PIK3CA in breast cancer. Bone, kidney and central nervous system (CNS) cancer projections have the fewest connections. The genes PIK3CA and TP53 seem to be most connected to other genes in breast cancer, with PIK3CA and GATA3 co-occurring the highest number of times. Prostate cancer does not show an evident co-occurrence pattern, and almost all edges have a weight of 1, however, there were some central nodes including CTNNB1, RB1, and AR. Lymph and skin cancer have multiple edges with mostly low weights and a few genes that are more connected, namely, SGK1 in lymph cancer and MUC16, TP53, and BRAF in skin cancer. The other cancer types have fewer edges, with TP53 connected to almost every other node in ovarian cancer.

## 3 Discussion

This study reduces the computational complexity of sequence similarity network analyses applied to human cancer genomes by focusing on likely driver mutations to identify cancer type mutation specificities and patterns of co-occurrence. Additionally, the computational burden is further lowered by filtering the dataset to keep only recurrent gene mutations and generating a reference sequence containing only the needed genetic information. The final dataset comprised 934 samples involving 1,264 genes. The genes were filtered using different approaches depending on whether the genes were present in the COSMIC Cancer Gene Census (CGC) catalogue. This list is the most comprehensive and accurate set of validated cancer genes annotated from the literature (Forbes et al., 2017). Hence, a more lenient filtering step was used for COSMIC CGC genes compared to genes not in COSMIC CGC.

Two network-based approaches, SSN analysis and bipartite network analysis, were implemented to study cancer driver genes and their role in tumors. Using DiWANN, a variant of SSN that has not been previously used to study cancer genes, we got insights into the general pattern of different cancers. For instance, it is evident from the DiWANN network that pancreatic cancer nodes are more connected amongst themselves than to other nodes. The individual views of each cancer type helped us identify patterns more conveniently. These individual networks suggest that cancer types vary in terms of connectedness, that is, how similar samples are to other samples of the same cancer type. Pancreatic and lymph cancer nodes are well connected, while prostate cancer nodes have very few connections to each other. Among the other cancers shown in Supplementary Figures S1 and S2, esophageal, liver, and myeloid seem to have just a few connected nodes.

Community detection results complemented our findings and provided additional insights on the behavior of different cancer types. The Louvain algorithm has proven to be among the most efficient community detection algorithms based on evaluation for appropriate community size and significant representation of groups within communities (Rahiminejad et al., 2019), and hence was chosen for this study. Different resolution values used for Louvain clustering enriched annotations differently, with the resolution value of 1.5 being the most optimal in terms of enrichment of cancer types as well as causative genes. The smaller clusters obtained with resolution values more than one are more enriched and could potentially help prioritize genes or mutations for targeted drug development. Clusters breaking down also signifies disease subtypes.

Cancer specific insights from clustering can be used to set up experiments on identifying drug targets. For instance, the largest cluster comprising all cancer types contained 212 samples, out of which 160 had mutations in BCL2. This indicates that BCL2 has a significant role in pan cancer analysis and could be a common target for cancer treatments. Community detection also showed that certain cancer types tend to cluster more with their own types, such as pancreatic, lymph, skin and breast.

The time complexity of generating the SSN is reduced using DiWANN as DiWANN maintains only the minimum distance edges. The calculation needed for maintaining nearest distances is made efficient by prunings and bounding optimizations, unlike standard threshold-based SSN, where all pairwise distance computations are made. Besides computational efficiency, DiWANN is also advantageous in terms of structural information it retains. Specifically, since an edge in DiWANN is drawn from every node to the node’s nearest neighbor in the similarity space, there are no singleton nodes in the eventual network, unlike threshold-based SSN that could contain singleton nodes, potentially causing loss of information.

Bipartite network analysis provided insights into gene associations in tumor samples and cancer types. The first bipartite network consisting of tissues in one set and genes in the other shows which genes occur in which cancer types (tissues). The degree analysis of this bipartite network suggests that TP53 occurs in almost all cancer types (Olivier et al., 2010), followed by PIK3CA and SMARCA4 occurring in nine cancer types, indicating their importance in multiple cancers (Kang et al., 2020; Peng et al., 2021). The tissues that have the highest degrees are the ones that are driven by many genes and can be said to be more heterogeneous. For instance, prostate, lymph and skin have the highest degrees (genes) and are highly heterogeneous tumors (O’Connor and Tobinai, 2014; Grzywa et al., 2017; Carm et al., 2019), suggesting they might be too diverse for common drugs and treatments. The weighted one mode tissue projection from the tissue gene bipartite graph shows the number of common genes a pair of tissues have, with the highest being 103 among pancreatic and prostate, suggesting they have the greatest number of common genes mutated in the two cancers.

We obtained information about the co-occurrence of genes from the sample gene bipartite network. From the weighted one mode gene projection generated from this bipartite network in Figure 10, we see that TP53 and KRAS are the most frequently co-occurring pair, with an edge weight much higher than any other edge. Other pairs that noticeably co-occur are KRAS and CDKN2A, TP53 and CDKN2A, and TP53 and CTNNB1. Most of these pairs are tumor suppressor gene-oncogene pairs except for TP53 and CDKN2A, which are both TSGs, suggesting a strong need for both inactivation of a TSG and activation of an oncogene to promote cancer progression (Zhu et al., 2015). Weighted one mode projections generated individually for each cancer type suggest that there are certain genes or gene associations that are exclusive to certain cancer types.

Certain gene pairs exhibit distinct mutation patterns in different cancer types (Sinkala, 2023). The gene projections generated for each cancer type show that most of the co-occurring TP53 and KRAS pairs are in pancreatic cancer, as shown in Figure 11A and Supplementary Table S7, suggesting their importance in driving pancreatic cancer (Kim et al., 2021) and highlighting that both mutations are likely required for metastasis on these tumors. Figure 11B suggests that TP53 is required along with most of the other driver genes in esophageal cancer. Supplementary Figures S3 and S4 suggest patterns in occurrence of genes in other cancer types. Genes in CNS, bone and kidney cancer tend to exist individually, indicating there is less co-occurrence of genes driving the cancer. TP53 is an important gene in many cancers, however, it is seen to be co-occurring with most of the other genes in biliary, ovary, pancreatic, and skin suggesting TP53’s vital role in driving these cancers. Lymph, prostate, and skin cancers seem to have many connected genes suggesting they are diverse with high mutational frequency.

Identifying driver mutations and how mutated genes affect the biology of a given tumor are fundamental challenges in cancer genomics. The same somatic mutation in a driver gene may have different effects in different cancer types (Watson et al., 2013; Porta-Pardo et al., 2020). This could depend on the other mutations in the tumor. Different analytical approaches have been used in previous studies to understand the role of driver genes in human cancers. Some studies have performed pathway analysis to understand interactions between genes and mutational heterogeneity in cancer (Leiserson et al., 2015; Reyna et al., 2020). However, these methods focus on the discovery of cancer modules rather than prioritizing individual cancer driver genes. In contrast, this study analyses mutations in the genomic sequences of individual driver genes in 15 human cancers. Some studies have used statistical methods to identify or prioritize driver genes and their interactions (Miller et al., 2011; Jiang et al., 2019) Such methods can be error prone (Nussinov et al., 2019).

This study has used edit distance as the similarity metric to generate the SSNs. There are studies that have constructed gene similarity networks using different metrics, including co-occurrence probabilities (Mirzaei, 2023). Identifying co-occurring genes is one of the important findings in this study, and so is identifying cancer specific behaviors using the DiWANN network. Edit distance accurately quantifies the similarity between sequences of each TCGA sample.

With advances in DNA-sequencing technologies and collaborative projects such as TCGA and ICGC, thousands of cancer genomes have been sequenced and made available. However, there is still not enough diversity and sequences available to identify driver genes in all cancer types. This study focuses on individual cancer driver genes in 15 human cancers and is a data-centric, efficient computational approach. Therefore, as the number of cancer genomes sequences increases over time, we expect the usefulness of this approach will increase.

There are a few limitations of this study that can be further improved. Firstly, the data is slightly imbalanced in terms of number of samples from different cancer types. As seen from the pie chart in Figure 1D, the proportion of pancreatic cancer samples is much higher than the others, while some cancers, such as head, kidney, and bone, have very few tumor samples. Additionally, excluding amplifications and large deletions might have filtered out some tumor samples from the original dataset. Therefore, there might be some bias in the observations we see, however, most of the observations made have been validated and seem to be consistent with all the results.

Another limitation of this study is the way the driver genes have been selected, that is, using mutation frequency more than one for COSMIC CGC genes and the top 4% for others. However, the threshold for COSMIC CGC is very low, reducing chances of missing driver mutations and the threshold for other genes was selected in a way to ensure the top occurring genes were selected while maintaining a fair distribution of cancer types. The dataset can be expanded by taking care of these concerns in the future and with continued accumulation of human tumor whole genome sequences. This increase in dataset size will likely power additional patterns of cancer mutations upon similar network analyses.

## 4 Materials and methods

### 4.1 Data sources

The study focuses on mutational driver genes capable of driving tumorigenesis via single-nucleotide variants (SNVs) and short insertions or deletions. With reduction in sequencing costs, it has become easier to obtain genomic information at the level of SNVs. A mutation annotation format (maf) file containing SNVs and aggregated mutation information at a project level, which is a part of The Pan-Cancer Analysis of Whole Genomes (PCAWG) study, was extracted from International Cancer Genome Consortium (ICGC) (Campbell et al., 2020). The PCAWG is an international collaboration to identify common patterns of mutation in more than 2,600 cancer whole genomes from the ICGC. The maf file contained over 23 million mutations for 1830 donors and 25 projects. Additionally, we have made use of the Catalogue of Somatic Mutations in Cancer (COSMIC) Cancer Gene Census (CGC), which is a catalogue of genes that contain mutations that have been causally implicated in cancer to help us consider only the relevant genes in the dataset (Tate et al., 2019).

### 4.2 Data reduction

The maf file contained over 23 million mutations and 32,269 genes. The mutations were of different variant types and included non-coding region mutations. We are interested in functionally significant regions, so the dataset was filtered to exclude non-coding regions, which include three variant classes, namely, inter-genic region (IGR), intron, lincRNA, and 5’ Flank. Non-coding genes are rarely found to be driver genes (Rheinbay et al., 2020). Furthermore, the dataset was filtered based on inclusion of genes in the COSMIC CGC. Recurrence of a mutation in patients remains one of the most reliable markers of its driver status, and its frequency can be adjusted based on background mutability (Brown et al., 2019). Therefore, for CGC genes, we chose to select mutations that occur more than once, potentially eliminating random passenger mutations for this study. For genes not included in COSMIC CGC, we chose gene–mutation pairs that represented the top 4% of frequency within each cancer type. This ensured distributed and normalized selection of genes for all cancers.

This study required generation of a single sequence for each donor by combining mutation information for all the genes in the final dataset. A straight-forward way of doing this would be to combine the cDNA sequence for each gene into a single sequence and only change the nucleotides that have mutated for that donor. However, this would result in a very long sequence, leading to a computationally extremely expensive process of creating the DiWANN network. To reduce the computational cost of the process, we create a transformed sequence for each donor using only the recurrent mutations for each gene. All the recurrent mutations are considered for each gene, and a sequence is made by concatenating the original nucleotide for each mutation. These sequences representing the wild type nucleotides for each gene are then combined to form a single transformed sequence. The order in which the genes and the mutations for each gene are concatenated is kept the same for every donor, avoiding any possible difference in the significance of the biological sequences. Consequently, we have a reference sequence for each donor in which we change the nucleotides that have mutated for that donor and keep the other wild type nucleotides the same. The source code for data reduction and network analysis done in this study is available at https://github.com/ShrutiPatil13/CancerNetworkAnalysis.

### 4.3 Network analysis

Two network-based approaches were implemented to study cancer driver genes: DiWANN network analysis and bipartite network analysis. The DiWANN network is a variant of sequence similarity network (SSN) where each node (sequence) is connected to only its closest neighbor(s). The distance (dissimilarity) between nodes is measured using edit distance (Kim et al., 2021). The construction algorithm used in the DiWANN model uses a pruning and bounding method to avoid costly distance calculations (Catanese et al., 2018). The algorithm calculates the distances for the first sequence and then prunes out the distance calculations not needed and bounds the calculations needed for other sequences. This avoids calculating pairwise distance matrix for all sequences, reducing the computation needed and ultimately the time to construct the network. Note, we use the reference (wild type) sequence generated as the first sequence.

The implementation was run on Washington State University’s high performance computing cluster, Kamiak, which further reduced the network construction time. The constructed DiWANN network was visualized using tools from igraph and NetworkX in Python (Proceedings of the Python in Science Conference SciPy: Exploring Network Structure, Dynamics, and Function using NetworkX, 2022; Csárdi and Nepusz, 2006). The force directed layout called Fruchterman-Reingold layout was utilized to place nodes on the plane. The nodes were colored by tumor tissue (type) and sized according to the number of occurrences of the sequence in the final dataset. The edge length and width were made proportional to the edge weight, connecting more dissimilar sequences with a longer and thicker edge. Additionally, to see patterns in each cancer type better, individual views of the DiWANN network were generated. The implementation efficiency of a DiWANN network was compared to the standard threshold-based SSN using the same distance metric, edit distance. Community detection was performed on the DiWANN network using the Louvain algorithm. Node annotations, namely, cancer type and causative genes, were analyzed for enrichment at different resolution values using the Fischer exact test (Upton, 1992).

The second network-based approach used bipartite networks to study associations between driver genes in samples and in different cancer types. Two bipartite networks were constructed: a tissue-gene bipartite network and a sample-gene bipartite network. Weighted one mode projections of genes and cancer types (tissues) were then constructed from these bipartite networks. NetworkX was used to construct and study these bipartite networks. Degree analysis of the bipartite networks provided us with information on the occurrences of genetic alterations in genes and different cancer types. Additionally, weighted one mode projections were generated for individual cancer types for a deeper analysis.

## 5 Conclusion

Identifying driver genes and understanding the behavior of mutations is important in cancer genetics. This study implemented two network-based approaches: analysis done using the DiWANN network model, which is a variant of SSN, and analysis done using an underlying bipartite network to identify patterns in driver genes and in different cancer types. A data reduction framework extracted relevant information from a PCAWG maf file provided by ICGC and generated a transformed reference sequence from the selected driver genes to construct the DiWANN network. The data reduction process and utilization of the DiWANN model to study sequences lowered the computational expenses. The DiWANN networks helped us identify cancer types that are more connected than others suggesting which cancers would benefit from generalized treatments and which would need more personalized treatments. We identified many gene associations pertinent to cancer using the bipartite network analysis. Some driver genes played a role in multiple cancer types while some were exclusive to specific cancer types. Therefore, we demonstrated how network analysis can effectively be used to study cancer genetics.

## Data Availability

The original contributions presented in the study are included in the article/Supplementary Material, further inquiries can be directed to the corresponding author.
