# Supplementary material for: Network analysis of driver genes in human cancers
Source: Front Bioinform. 2024 Jul 8;4:1365200. doi: 10.3389/fbinf.2024.1365200 (PMC11260686; doi:10.3389/fbinf.2024.1365200)
Supplement: Supplementary file 1 [file DataSheet1.docx]

Supplementary Material

Network analysis of driver genes in human cancers

Shruti S. Patil, Steven A. Roberts, Assefaw H. Gebremedhin

*** Correspondence:** Corresponding Author: assefaw.gebremedhin@wsu.edu

# Supplementary Figures and Tables

## Supplementary Figures


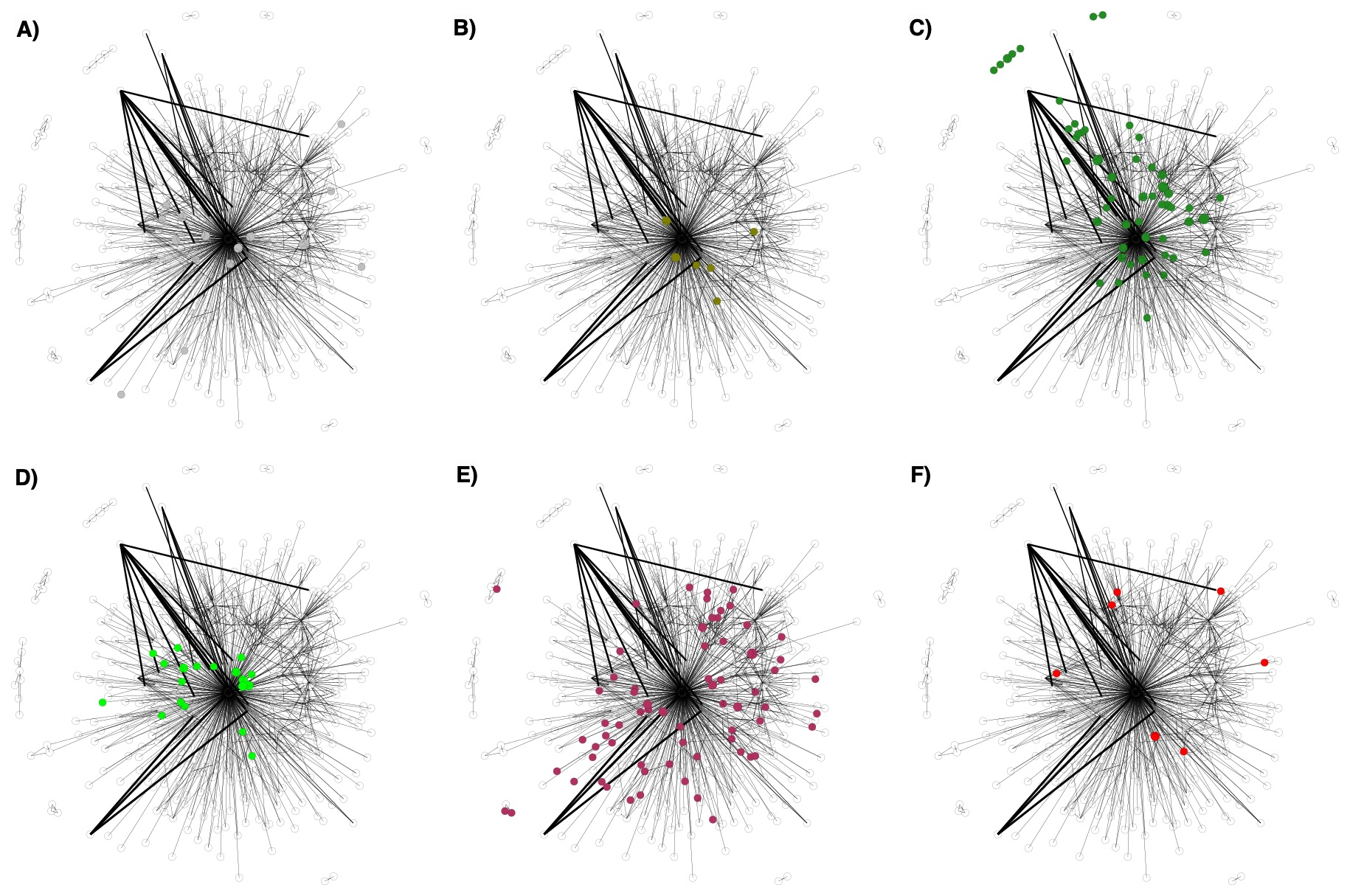


**Supplementary Figure 1.** DiWANN networks consisting of 672 nodes with colored nodes for (A) Billiary cancer, (B) Bone cancer, (C) Breast cancer, (D) CNS cancer, (E) Esophageal cancer, and (F) Head cancer. The nodes are colored if they represent a tumor sample for the cancer type. The edge weight is proportional to the edit distance between the sequences (nodes).


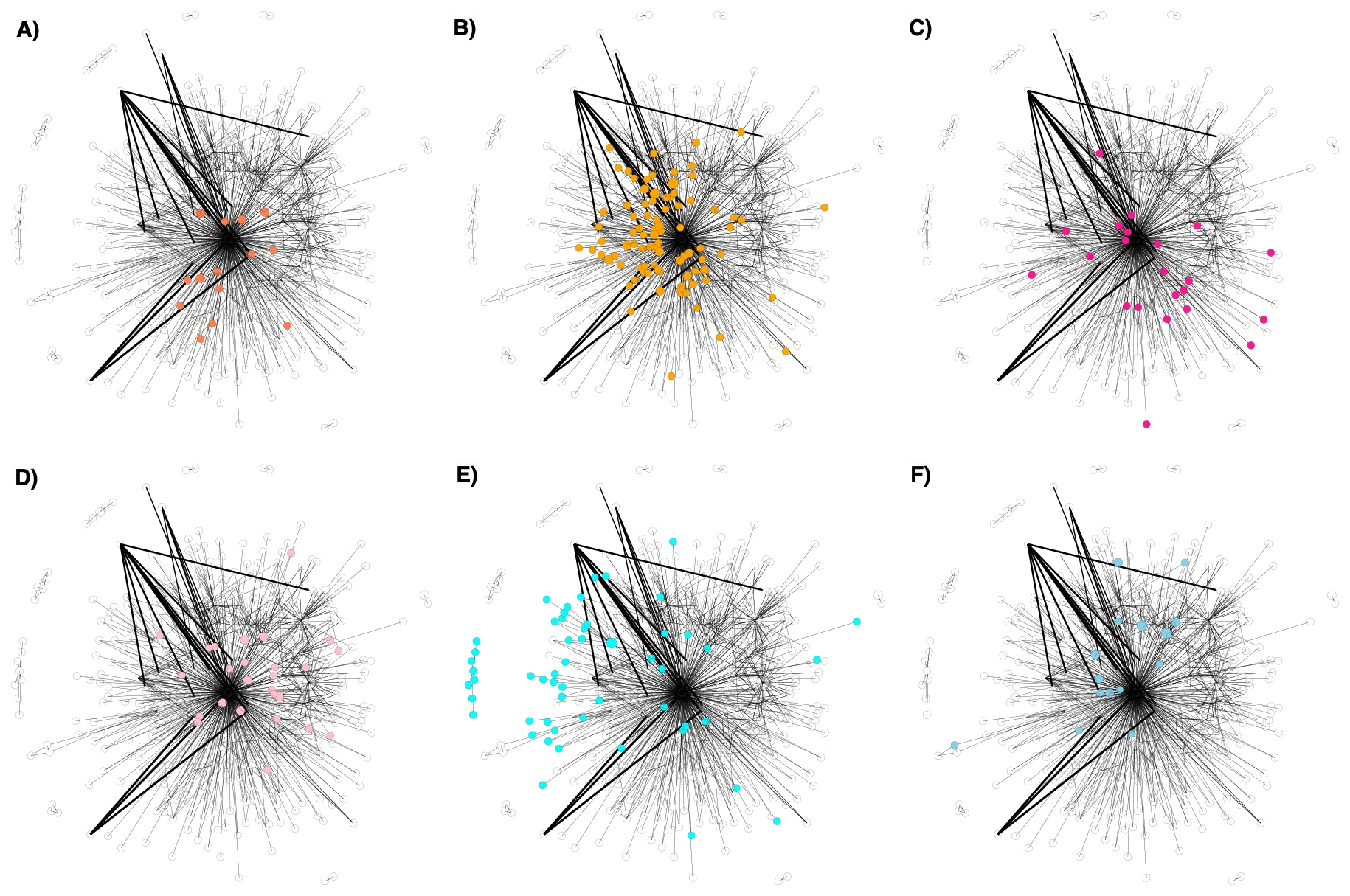


**Supplementary Figure 2.** DiWANN networks consisting of 672 nodes with colored nodes for (A) Kidney cancer, (B) Liver cancer, (C) Myeloid cancer, (D) Ovary cancer, (E) Skin cancer, and (F) Stomach cancer. The nodes are colored if they represent a tumor sample for the cancer type. The edge weight is proportional to the edit distance between the sequences (nodes).


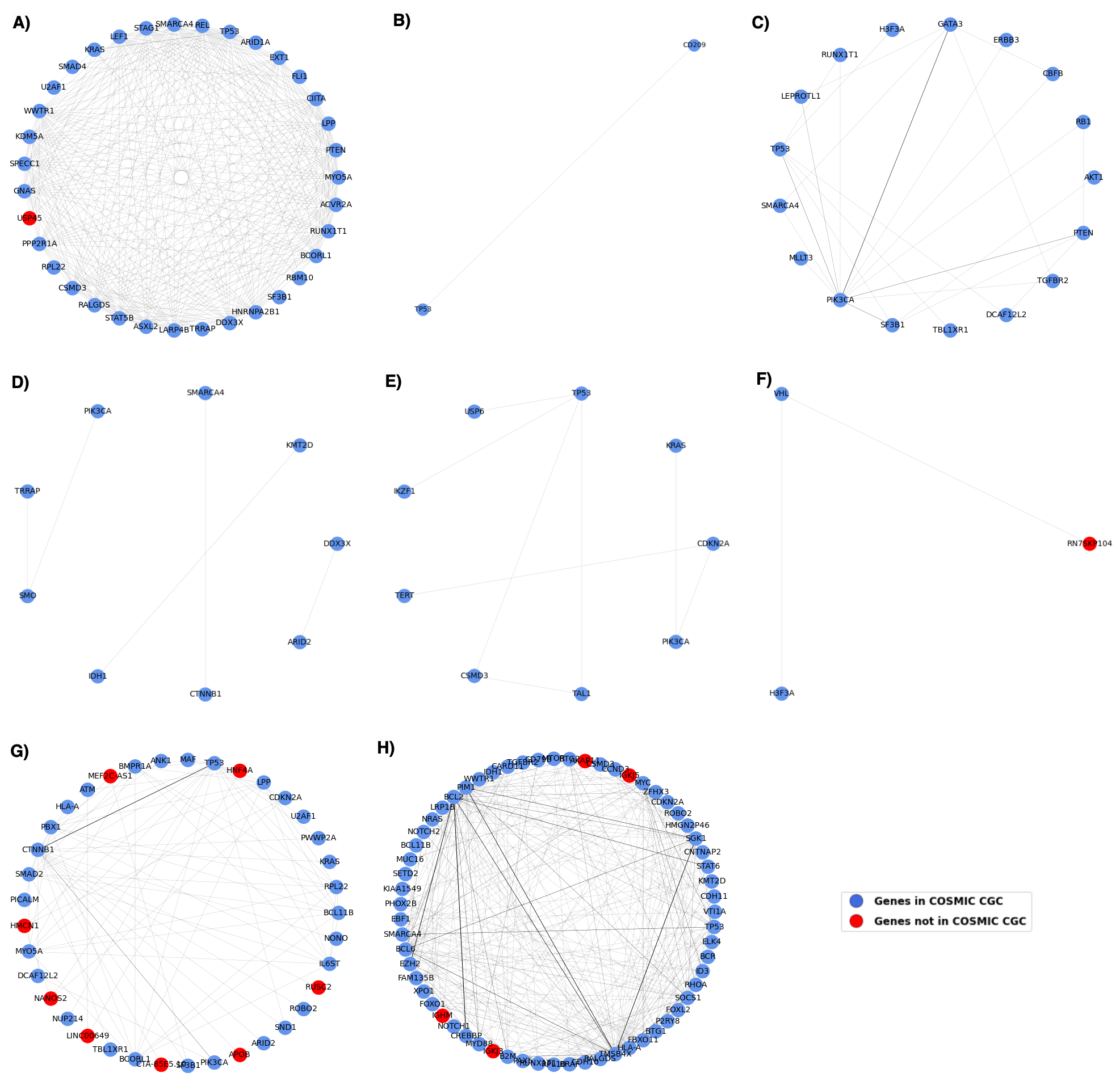


**Supplementary Figure 3.** Weighted one mode gene projections for (A) Billiary, (B) Bone, (C) Breast, (D) CNS, (E) Head, (F) Kidney, (G) Liver, and (H) Lymph. The nodes are colored depending on if the gene is present in COSMIC CGC as shown in the legend and the edge width is 1/10th of edge weight.


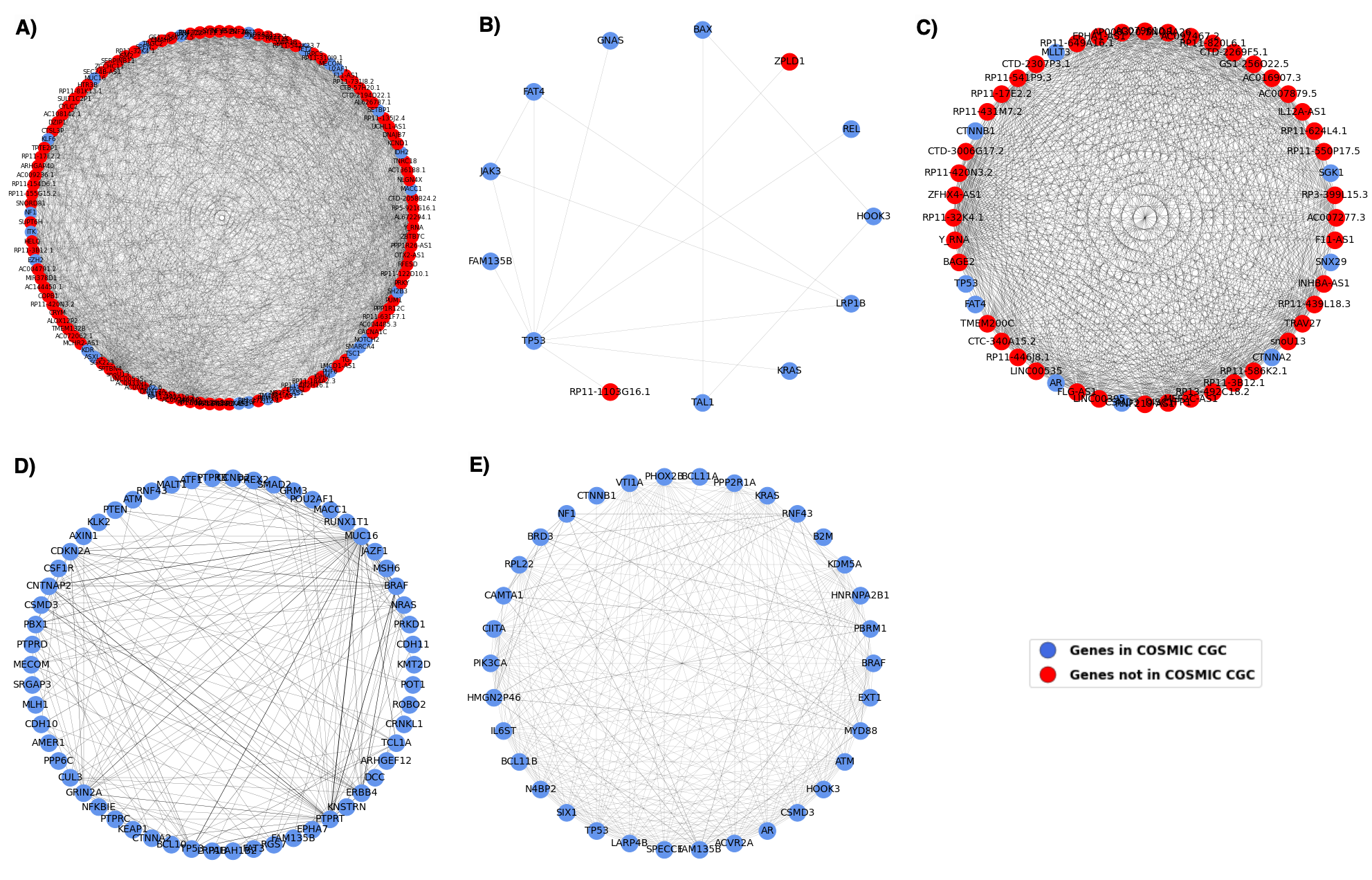


**Supplementary Figure 4.** Weighted one mode gene projections for (A) Myeloid, (B) Ovary, (C) Prostate, (D) Skin, and (E) Stomach. The nodes are colored depending on if the gene is present in COSMIC CGC as shown in the legend and the edge width is 1/10th of edge weight. Projections for Myeloid, Prostate, and Skin are subgraphs of the original projection and consists of nodes having an edge weight of 2 or more.

## Supplementary Tables

**Supplementary Table 1.** The final reduced mutational list for each sample.

| **Donor_ID** | **Hugo_Symbol** | **Genome_Change** | **Tissue** |
| --- | --- | --- | --- |
| DO1001 | TP53 | g.chr17:7577539G>A | Breast |
| DO1002 | USP6 | g.chr17:5042895G>A | Breast |
| DO1005 | TP53, TBL1XR1 | g.chr17:7578212G>A, g.chr3:176771665T>C | Breast |
| DO1007 | PIK3CA, GATA3 | g.chr3:178952085A>G, g.chr10:8111433_8111434delCA | Breast |
| DO1010 | TP53 | g.chr17:7578190T>C | Breast |
| DO1013 | PIK3CA | g.chr3:178952085A>G | Breast |
| DO1016 | TP53 | g.chr17:7576852C>T | Breast |
| DO1017 | AKT1 | g.chr14:105246551C>T | Breast |
| DO1076 | GATA3, PIK3CA, LEPROTL1 | g.chr10:8106058T>A, g.chr3:178952085A>G, g.chr8:29952919G>A | Breast |
| DO10809 | TFEB | g.chr6:41655684G>A | Prost |
| DO10815 | TP53 | g.chr17:7578265A>G | Prost |
| DO10821 | GRIN2A | g.chr16:9856044C>T | Prost |
| DO10839 | TP53 | g.chr17:7577539G>A | Eso |
| DO10840 | TP53, ARID1A, PBX1 | g.chr17:7577120C>T, g.chr1:27105930_27105931insG, g.chr1:164821059delA | Eso |
| DO10841 | TP53, AR | g.chr17:7578268A>C, g.chrX:66948781T>G | Eso |
| DO10843 | TP53 | g.chr17:7578406C>T | Eso |
| DO10844 | TGFBR2 | g.chr3:30732969C>T | Eso |
| DO10858 | TP53, NRG1, FAM135B | g.chr17:7578394T>A, g.chr8:32617831T>C, g.chr8:139180251G>A | Eso |
| DO217786 | SF3B1, PIK3CA | g.chr2:198266834T>C, g.chr3:178936091G>A | Breast |
| DO217800 | TP53 | g.chr17:7578534C>G | Breast |
| DO217814 | AC108142.1 | g.chr4:182915295delA | Stomach |
| DO217818 | TP53 | g.chr17:7577570C>T | Stomach |
| DO217822 | TP53 | g.chr17:7578406C>T | Stomach |
| DO217826 | SF3B1, PIK3CA, PTEN | g.chr2:198266834T>C, g.chr3:178936091G>A, g.chr10:89720799_89720802delTACT | Breast |
| DO217836 | PIK3CA | g.chr3:178936082G>A | Head |
| DO217844 | JAK3 | g.chr19:17935777G>A | Breast |
| DO217850 | KRAS, TP53, RBM10, USP45 | g.chr12:25398284C>T, g.chr17:7578205C>T, g.chrX:47041266G>A, g.chr6:99963300_99963306delGGGGAGA | Biliary |
| DO217887 | PIK3CA | g.chr3:178952085A>T | Breast |
| DO217896 | NRG1 | g.chr8:32463091T>G | Stomach |
| DO217907 | PTEN | g.chr10:89692904C>T | Breast |
| DO217908 | PIK3CA, LEPROTL1 | g.chr3:178952085A>G, g.chr8:29952919G>A | Breast |
| DO217931 | SF3B1 | g.chr2:198266834T>C | Breast |
| DO217934 | GATA3 | g.chr10:8111433_8111434delCA | Breast |
| DO217939 | PIK3CA, GATA3 | g.chr3:178936094C>A, g.chr10:8111433_8111434delCA | Breast |
| DO217950 | PIK3CA, CDKN2A | g.chr3:178936091G>A, g.chr9:21971120G>A | Head |
| DO217953 | GATA3 | g.chr10:8111433_8111434delCA | Breast |
| DO217962 | CBFB, PIK3CA, GATA3 | g.chr16:67063717G>C, g.chr3:178936091G>A, g.chr10:8111433_8111434delCA | Breast |
| DO217987 | TP53 | g.chr17:7578479G>A | Breast |
| DO218030 | TP53 | g.chr17:7577538C>T | Stomach |
| DO218031 | CAMTA1, HMGN2P46, NF1, RNF43, MYD88, PBRM1, FAM135B, BRD3 | g.chr1:7811392A>C, g.chr15:45819288A>C, g.chr17:29556986T>C, g.chr17:56435421T>G, g.chr3:38183963C>T, g.chr3:52637659A>C, g.chr8:139160865A>G, g.chr9:136905165G>A | Stomach |
| DO218060 | PIK3CA | g.chr3:178952085A>G | Breast |
| DO218065 | AKT1 | g.chr14:105246551C>T | Breast |
| DO218072 | PIK3CA | g.chr3:178952085A>G | Breast |
| DO218075 | USP6, TP53 | g.chr17:5042895G>A, g.chr17:7577022G>A | Head |
| DO218088 | TP53 | g.chr17:7577121G>A | Stomach |
| DO218121 | CBFB | g.chr16:67063717G>C | Breast |
| DO218139 | KRAS, TP53, U2AF1 | g.chr12:25398281C>T, g.chr17:7577120C>T, g.chr21:44524456G>A | Biliary |
| DO218173 | GATA3, TGFBR2, PIK3CA | g.chr10:8106058T>A, g.chr3:30732956C>T, g.chr3:178936091G>A | Breast |
| DO218174 | PIK3CA | g.chr3:178936091G>A | Breast |
| DO218180 | KRAS, PIK3CA | g.chr12:25398284C>T, g.chr3:178936094C>A | Head |
| DO218205 | TP53, PIK3CA | g.chr17:7576855G>A, g.chr3:178952085A>G | Breast |
| DO218223 | CD209 | g.chr19:7806355C>T | Stomach |
| DO218227 | ARID1A, TP53 | g.chr1:27101099C>T, g.chr17:7577085C>T | Biliary |
| DO218243 | CAMTA1, HMGN2P46, NF1, RNF43, MYD88, PBRM1, FAM135B, BRD3 | g.chr1:7811392A>C, g.chr15:45819288A>C, g.chr17:29556986T>C, g.chr17:56435421T>G, g.chr3:38183963C>T, g.chr3:52637659A>C, g.chr8:139160865A>G, g.chr9:136905165G>A | Stomach |
| DO218269 | TP53 | g.chr17:7578382G>C | Breast |
| DO218280 | TP53, IKZF1 | g.chr17:7577085C>T, g.chr7:50367289G>A | Head |
| DO218282 | TP53 | g.chr17:7578212G>A | Stomach |
| DO218306 | TP53, CSMD3, TAL1 | g.chr17:7577539G>A, g.chr8:113323333G>T, g.chr1:47682037_47682038insT | Head |
| DO218333 | CHST11 | g.chr12:105150997G>A | Breast |
| DO218347 | ERBB3, PIK3CA | g.chr12:56492633A>G, g.chr3:178952085A>G | Breast |
| DO218408 | GATA3 | g.chr10:8111433_8111434delCA | Breast |
| DO218411 | ANK1 | g.chr8:41512976G>A | Biliary |
| DO218417 | TP53 | g.chr17:7578527A>G | Biliary |
| DO218428 | TP53 | g.chr17:7578394T>C | Breast |
| DO218440 | TP53 | g.chr17:7577560A>G | Biliary |
| DO218442 | TP53 | g.chr17:7578397_7578398insG | Stomach |
| DO218443 | FBXW7 | g.chr4:153249385G>A | Stomach |
| DO218462 | TP53 | g.chr17:7578406C>T | Head |
| DO218478 | DCAF12L2, PTEN | g.chrX:125298907G>A, g.chr10:89720799_89720802delTACT | Breast |
| DO218489 | PIK3CA | g.chr3:178952085A>T | Breast |
| DO218491 | IKZF1 | g.chr7:50468997C>T | Biliary |
| DO218502 | TP53 | g.chr17:7578416C>A | Breast |
| DO218506 | SMARCA4, PIK3CA, GATA3 | g.chr19:11118598G>A, g.chr3:178952085A>G, g.chr10:8111433_8111434delCA | Breast |
| DO218535 | TP53 | g.chr17:7577120C>T | Biliary |
| DO218547 | ERBB4 | g.chr2:212288967G>A | Stomach |
| DO218550 | FLI1, TP53, SMAD4 | g.chr11:128628193C>T, g.chr17:7577574T>C, g.chr18:48575152C>T | Biliary |
| DO218553 | GATA3 | g.chr10:8111433_8111434delCA | Breast |
| DO218560 | AKT1, SF3B1 | g.chr14:105246551C>T, g.chr2:198266834T>C | Breast |
| DO218611 | PIK3CA | g.chr3:178917478G>A | Breast |
| DO218621 | GNAS | g.chr20:57484420C>T | Breast |
| DO218651 | TP53 | g.chr17:7574018G>A | Breast |
| DO218656 | PIK3CA | g.chr3:178952085A>G | Breast |
| DO218673 | TP53, CTNNB1 | g.chr17:7578212G>A, g.chr3:41266101C>G | Stomach |
| DO218684 | TP53 | g.chr17:7578266T>A | Breast |
| DO218693 | KRAS, TP53, PIK3CA, CSMD3, RPL22, LARP4B, VTI1A, ATM, KDM5A, SIX1, BCL11B, CIITA, SPECC1, PPP2R1A, ACVR2A, N4BP2, PHOX2B, IL6ST, HNRNPA2B1, BRAF, HOOK3, EXT1, AR | g.chr12:25398284C>T, g.chr17:7574003G>A, g.chr3:178952085A>G, g.chr8:114449220C>T, g.chr1:6257785delT, g.chr10:890939delT, g.chr10:114578212_114578213insT, g.chr11:108160451delA, g.chr12:390703delA, g.chr14:61110397delA, g.chr14:99638831delA, g.chr16:11022451delC, g.chr17:20108263delA, g.chr19:52729587delT, g.chr2:148683686delA, g.chr4:40158194delT, g.chr4:41746280delA, g.chr5:55236037delT, g.chr7:26231741delT, g.chr7:140434295delA, g.chr8:42884886delT, g.chr8:118808897delT, g.chrX:66943842delT | Stomach |
| DO218695 | ARID1A, TP53 | g.chr1:27101138C>T, g.chr17:7578406C>T | Biliary |
| DO218697 | TERT, CDKN2A | g.chr5:1295161T>G, g.chr9:21971120G>A | Head |
| DO218698 | PRRC2C | g.chr1:171562157delT | Breast |
| DO218709 | TP53 | g.chr17:7577581A>G | Breast |
| DO218736 | TP53, SF3B1, PIK3CA | g.chr17:7578205C>A, g.chr2:198266834T>C, g.chr3:178952085A>G | Breast |
| DO218742 | PIK3CA | g.chr3:178936091G>A | Breast |
| DO218769 | CTNNB1, B2M | g.chr3:41266097G>A, g.chr15:45003786_45003789delTCTT | Stomach |
| DO218770 | PIK3CA | g.chr3:178952085A>G | Breast |
| DO218773 | TP53 | g.chr17:7578394T>C | Head |
| DO218796 | TP53 | g.chr17:7577538C>T | Breast |
| DO218828 | TP53 | g.chr17:7577580T>C | Breast |
| DO220820 | PIK3CA, MLLT3 | g.chr3:178952085A>G, g.chr9:20622295delC | Breast |
| DO220821 | PTEN, PIK3CA | g.chr10:89692905G>A, g.chr3:178952085A>G | Breast |
| DO220822 | PIK3CA | g.chr3:178936091G>A | Breast |
| DO220823 | AKT1 | g.chr14:105246551C>T | Breast |
| DO220825 | PIK3CA | g.chr3:178952085A>G | Breast |
| DO220828 | TP53, PIK3CA, RUNX1T1 | g.chr17:7578442T>C, g.chr3:178936082G>A, g.chr8:92970812T>A | Breast |
| DO220844 | ARID2 | g.chr12:46287446C>T | Skin |
| DO220845 | BRAF | g.chr7:140453136A>T | Skin |
| DO220846 | RUNX1T1 | g.chr8:92970812T>A | Skin |
| DO220847 | BRAF | g.chr7:140481402C>T | Skin |
| DO220848 | BRAF | g.chr7:140453136A>T | Skin |
| DO220849 | CD209, CNTNAP2 | g.chr19:7805166C>T, g.chr7:148115135G>A | Skin |
| DO220851 | PBX1, CDH11, SMAD2, MALT1, CTNNA2, CTNNA2, ERBB4, ERBB4, PTPRT, CTNND2, NFKBIE, MACC1, GRM3, RUNX1T1, CSMD3, FAM47C | g.chr1:164816639G>A, g.chr16:64979190G>A, g.chr18:45359384G>A, g.chr18:56338637C>T, g.chr2:80085165G>A, g.chr2:80875001G>A, g.chr2:212247212C>T, g.chr2:212566827C>T, g.chr20:40704390G>A, g.chr5:10972773G>A, g.chr6:44233400C>T, g.chr7:20177809C>T, g.chr7:86493793G>A, g.chr8:93017364C>T, g.chr8:113267561G>A, g.chrX:37029325G>A | Skin |
| DO220852 | RBM15, NRAS, LIFR, AC016725.4 | g.chr1:110881938T>C, g.chr1:115256529T>C, g.chr5:38527329C>T, g.chr2:135501452delT | Skin |
| DO220853 | NRAS, MUC16 | g.chr1:115256529T>C, g.chr19:9084835G>A | Skin |
| DO220855 | ERBB4, PTPRT, PTPRT, BRAF | g.chr2:212241957C>T, g.chr20:40704913C>T, g.chr20:40707448C>T, g.chr7:140453136A>T | Skin |
| DO220857 | BCL10, NRAS, RGS7, ARHGEF12, TCL1A, TP53, MUC16, MUC16, FAM135B, CDKN2A, PPP6C | g.chr1:85742425C>T, g.chr1:115256529T>C, g.chr1:240939096C>T, g.chr11:120336011C>T, g.chr14:96176389G>A, g.chr17:7577099C>T, g.chr19:9046028G>A, g.chr19:9071139G>A, g.chr8:139143877C>T, g.chr9:21971120_21971121GG>AA, g.chr9:127912080G>A | Skin |
| DO220858 | BRAF | g.chr7:140453136A>T | Skin |
| DO220859 | PAX5 | g.chr9:37015150C>T | Skin |
| DO220860 | DDR2, PTPRT | g.chr1:162745609C>T, g.chr20:40704825C>T | Skin |
| DO220861 | NRAS, CUL3, PTPRT, PGM5P2 | g.chr1:115256530G>T, g.chr2:225336094A>G, g.chr20:40707448C>T, g.chr9:69133936delG | Skin |
| DO220862 | NRAS, PBX1, CCND2, MUC16, RUNX1T1 | g.chr1:115256530G>T, g.chr1:164816639G>A, g.chr12:4410138G>A, g.chr19:9063486G>A, g.chr8:92972737C>T | Skin |
| DO220863 | BRAF | g.chr7:140453136A>T | Skin |
| DO220866 | ELK4, CTNND2 | g.chr1:205581993G>A, g.chr5:10972773G>A | Skin |
| DO220868 | BRAF | g.chr7:140453136A>T | Skin |
| DO220869 | BRAF | g.chr7:140453136A>T | Skin |
| DO220872 | ERBB4, PTPRT, BRAF, CSMD3, TMEM121 | g.chr2:212295748G>A, g.chr20:40790142C>T, g.chr7:140453136_140453137AC>TT, g.chr8:113275983G>A, g.chr14:105995761delT | Skin |
| DO220873 | NRAS, NBEA, CTNNB1, B2M | g.chr1:115256529T>C, g.chr13:36226005C>T, g.chr3:41266137C>A, g.chr15:45003781_45003784delCTCT | Skin |
| DO220874 | PRRX1, TP53, PTPRT, BRAF, MEPCE, PDE4B | g.chr1:170695508G>A, g.chr17:7577559G>A, g.chr20:40702531C>T, g.chr7:140453136A>T, g.chr7:100031463delT, g.chr1:66838793_66838795delAAT | Skin |
| DO220875 | PBX1, CCND2, PTPRB, MUC16, MUC16, CRNKL1, MLH1, EZH2, PREX2 | g.chr1:164820752C>T, g.chr12:4410138G>A, g.chr12:70911409C>T, g.chr19:9060584G>A, g.chr19:9061005C>T, g.chr20:20033106G>A, g.chr3:37067242C>T, g.chr7:148508728A>T, g.chr8:68981315C>T | Skin |
| DO220877 | PTPRC, RGS7, FAT3, PAFAH1B2, NBEA, PRKD1, GRIN2A, TP53, TP53, MUC16, MUC16, MUC16, PTPRT, MLH1, ROBO2, MECOM, GRM3, KIAA1549, CNTNAP2, CNTNAP2, PREX2, CSMD3, SLC24A5 | g.chr1:198697571G>A, g.chr1:240939096C>T, g.chr11:92088438G>A, g.chr11:117040414C>T, g.chr13:36226005C>T, g.chr14:30066932G>A, g.chr16:9856827C>T, g.chr17:7578212G>A, g.chr17:7578235T>C, g.chr19:9071434G>A, g.chr19:9075760C>T, g.chr19:9087904G>A, g.chr20:40703756G>A, g.chr3:37067242C>T, g.chr3:77147468G>A, g.chr3:168812978G>A, g.chr7:86493832G>A, g.chr7:138554308G>A, g.chr7:148113202C>T, g.chr7:148113314G>A, g.chr8:68995495G>A, g.chr8:113267561G>A, g.chr15:48429143delT | Skin |
| DO220878 | NRAS, RGS7, TCF7L2, ATM, HMGN2P46, DCC, SMARCA4, ERBB4, CUL3, MECOM, MECOM, DDR2, ELK4, SIX1, SMAD2, NONO, DIAPH2-AS1 | g.chr1:115256529T>C, g.chr1:240964790G>A, g.chr10:114926552G>A, g.chr11:108117798C>T, g.chr15:45842835A>T, g.chr18:50936877G>A, g.chr19:11118598G>A, g.chr2:212240845G>A, g.chr2:225449667G>A, g.chr3:168838897G>A, g.chr3:168840382C>T, g.chr1:162752532_162752533insT, g.chr1:205582792delC, g.chr14:61110397delA, g.chr18:45358789delA, g.chrX:70520707delA, g.chrX:96893512_96893517delGCTGGG | Skin |
| DO220879 | RBM15, RGS7, PTPRB, GRIN2A, MACC1, SND1, BRAF, CDKN2A | g.chr1:110881938T>C, g.chr1:240964790G>A, g.chr12:70928698G>A, g.chr16:9856827C>T, g.chr7:20177809C>T, g.chr7:127341308C>T, g.chr7:140453136_140453137AC>TT, g.chr9:21971028C>T | Skin |
| DO220880 | NRAS, GRIN2A, CD28 | g.chr1:115256530G>T, g.chr16:9855184C>T, g.chr2:204599740C>T | Skin |
| DO220881 | TP53, MUC16, BRAF, TM9SF4 | g.chr17:7576897G>A, g.chr19:9064481C>T, g.chr7:140453136_140453137AC>TT, g.chr20:30738658delT | Skin |
| DO220882 | NRAS, PDE4DIP, POU2AF1, MUC16, PTPRT, SRGAP3, PTEN | g.chr1:115256529T>C, g.chr1:144879284G>A, g.chr11:111223082G>A, g.chr19:9072091G>A, g.chr20:40704390G>A, g.chr3:9022849C>T, g.chr10:89693002_89693003insA | Skin |
| DO220883 | BRAF, PREX2 | g.chr7:140453136A>T, g.chr8:69031679C>T | Skin |
| DO220884 | PREX2 | g.chr8:68995495G>A | Skin |
| DO220885 | NRAS, PBX1, GRIN2A, KLK2, ERBB4, CDKN2A | g.chr1:115256530G>T, g.chr1:164816821C>T, g.chr16:9853354C>T, g.chr19:51382043C>T, g.chr2:212240845G>A, g.chr9:21971177C>A | Skin |
| DO220886 | CCND2, PRKD1, TCL1A, AXIN1, GRIN2A, RNF43, MALT1, MUC16, MUC16, KEAP1, BMP5, MACC1, GRM3, PREX2, FAM135B | g.chr12:4410599C>T, g.chr14:30066932G>A, g.chr14:96177989G>A, g.chr16:337958G>A, g.chr16:9855184C>T, g.chr17:56435092G>A, g.chr18:56338637C>T, g.chr19:9002184G>A, g.chr19:9069603C>T, g.chr19:10610444G>A, g.chr6:55625290C>T, g.chr7:20177809C>T, g.chr7:86493832G>A, g.chr8:69147753G>A, g.chr8:139143877C>T | Skin |
| DO220887 | ERBB4, BRAF, RUNX1T1 | g.chr2:212241957C>T, g.chr7:140453136A>T, g.chr8:92972737C>T | Skin |
| DO220889 | FAT3, ATF1, GRIN2A, CDH11, MUC16, MSH6, PTPRT, CNTNAP2 | g.chr11:92088438G>A, g.chr12:51213615C>T, g.chr16:9854265C>T, g.chr16:64979190G>A, g.chr19:9063486G>A, g.chr2:47935464G>A, g.chr20:40707582C>T, g.chr7:148112915C>T | Skin |
| DO220890 | NRAS, A1CF, ARHGEF12, ERCC4, ERBB4, MECOM, CSMD3 | g.chr1:115256529T>C, g.chr10:52561583C>T, g.chr11:120336011C>T, g.chr16:14042516C>T, g.chr2:212566827C>T, g.chr3:168812978G>A, g.chr8:113267561G>A | Skin |
| DO220891 | PBX1, PTPRC, KMT2D, TCL1A, TP53, MUC16, MUC16, CTNNA2, CD28, CRNKL1, PTPRT, CDH10, CSF1R, POT1, CNTNAP2, CDKN2A, RP11-231I13.2 | g.chr1:164820752C>T, g.chr1:198697571G>A, g.chr12:49426068G>A, g.chr14:96177989G>A, g.chr17:7578406C>T, g.chr19:9007840G>A, g.chr19:9088132G>A, g.chr2:80085165G>A, g.chr2:204599740C>T, g.chr20:20033106G>A, g.chr20:40703756G>A, g.chr5:24488123G>A, g.chr5:149492899C>T, g.chr7:124463636G>A, g.chr7:148115135G>A, g.chr9:21971120_21971121GG>AA, g.chr3:70357189delT | Skin |
| DO220892 | MUC16, BRAF, TRPM1 | g.chr19:9091402C>T, g.chr7:140453136A>T, g.chr15:31453357delT | Skin |
| DO220893 | NRAS, PTPRB, MUC16, IDH1, EPHA7 | g.chr1:115256530G>T, g.chr12:70928698G>A, g.chr19:9069548C>T, g.chr2:209113113G>A, g.chr6:93949924G>A | Skin |
| DO220894 | MECOM, BRAF, AMER1 | g.chr3:168840382C>T, g.chr7:140453136A>T, g.chrX:63408851G>A | Skin |
| DO220895 | KNSTRN, ERCC4, SETBP1, SMAD2, BRAF, PREX2 | g.chr15:40675107C>T, g.chr16:14042516C>T, g.chr18:42281368C>T, g.chr18:45359384G>A, g.chr7:140453136A>T, g.chr8:69147676C>T | Skin |
| DO220896 | CCND2 | g.chr12:4410599C>T | Skin |
| DO220897 | BRAF | g.chr7:140453136A>T | Skin |
| DO220898 | PAFAH1B2, ROBO2, BRAF | g.chr11:117040414C>T, g.chr3:77147467C>T, g.chr7:140453136_140453137AC>TT | Skin |
| DO220899 | MUC16, MUC16, MSH6, ERBB4, PTPRT, SRGAP3, JAZF1, CNTNAP2, ANK1, RUNX1T1 | g.chr19:9071139G>A, g.chr19:9072091G>A, g.chr2:47935464G>A, g.chr2:212495235C>T, g.chr20:40702531C>T, g.chr3:9022849C>T, g.chr7:27934895G>A, g.chr7:148112915C>T, g.chr8:41529889C>T, g.chr8:93017364C>T | Skin |
| DO220900 | ERBB4, GRM3, POT1, BRAF, CNTNAP2, CDKN2A | g.chr2:212288967G>A, g.chr7:86493793G>A, g.chr7:124463636G>A, g.chr7:140453136A>T, g.chr7:146829338G>A, g.chr9:21971208C>T | Skin |
| DO220901 | BRAF | g.chr7:140453136_140453137AC>TT | Skin |
| DO220902 | ELK4, A1CF, PTPRB, PTPRB, MUC16, MUC16, MUC16, MUC16, MUC16, LRP1B, PTPRT, PTPRT, PTPRT, NFKBIE, BRAF, CNTNAP2, PREX2, PAX5, AMER1, SMARCD2 | g.chr1:205581993G>A, g.chr10:52561583C>T, g.chr12:70911409C>T, g.chr12:70956848G>A, g.chr19:9002184G>A, g.chr19:9061005C>T, g.chr19:9067930C>T, g.chr19:9072100C>T, g.chr19:9088132G>A, g.chr2:141215150C>T, g.chr20:40704825C>T, g.chr20:40704913C>T, g.chr20:40708509G>A, g.chr6:44233400C>T, g.chr7:140453136_140453137AC>TT, g.chr7:146829338G>A, g.chr8:69147753G>A, g.chr9:37015150C>T, g.chrX:63408851G>A, g.chr17:61911573delT | Skin |
| DO220903 | BCL10, PBX1, POU2AF1, ATF1, TCL1A, AXIN1, GRIN2A, USP6, TP53, MUC16, MUC16, MUC16, MUC16, MUC16, PTPRT, EPHA7, BRAF, PTPRD, CDKN2A | g.chr1:85742425C>T, g.chr1:164816821C>T, g.chr11:111223082G>A, g.chr12:51213615C>T, g.chr14:96176389G>A, g.chr16:337958G>A, g.chr16:9853354C>T, g.chr17:5033275C>T, g.chr17:7577082C>T, g.chr19:9007840G>A, g.chr19:9064481C>T, g.chr19:9067930C>T, g.chr19:9069603C>T, g.chr19:9076168G>A, g.chr20:40790142C>T, g.chr6:93949924G>A, g.chr7:140453136A>T, g.chr9:8500976C>T, g.chr9:21971120G>A | Skin |
| DO220904 | TP53 | g.chr17:7578479G>C | Skin |
| DO220905 | TERT, BRAF | g.chr5:1295161T>G, g.chr7:140453136A>T | Skin |
| DO220906 | ATM, GRIN2A, TP53, SETBP1, DCC, MUC16, KEAP1, CTNNA2, LRP1B, CUL3, PTPRT, PTPRT, CDH10, CSF1R, JAZF1, PTPRD, IBTK | g.chr11:108117798C>T, g.chr16:9854265C>T, g.chr17:7577097C>T, g.chr18:42281368C>T, g.chr18:50936877G>A, g.chr19:9075760C>T, g.chr19:10610444G>A, g.chr2:80875001G>A, g.chr2:141215150C>T, g.chr2:225449667G>A, g.chr20:40707448C>T, g.chr20:40707582C>T, g.chr5:24488123G>A, g.chr5:149492899C>T, g.chr7:27934895G>A, g.chr9:8500976C>T, g.chr6:82924077delC | Skin |
| DO220907 | KMT2D, PTPRB, MUC16, MUC16, KLK2, ERBB4, ERBB4, CUL3, MECOM, TRRAP, BRAF, PTEN | g.chr12:49426068G>A, g.chr12:70956848G>A, g.chr19:9060584G>A, g.chr19:9072100C>T, g.chr19:51382043C>T, g.chr2:212248293G>A, g.chr2:212295748G>A, g.chr2:225336094A>G, g.chr3:168838897G>A, g.chr7:98509802C>T, g.chr7:140453136_140453137AC>TT, g.chr10:89693002_89693003insA | Skin |
| DO220908 | TRRAP, PREX2 | g.chr7:98509802C>T, g.chr8:69147676C>T | Skin |
| DO220909 | PTPRT, BRAF | g.chr20:40708509G>A, g.chr7:140453136_140453137AC>TT | Skin |
| DO220910 | ZFHX3, BRAF, CNTNAP2 | g.chr16:72828405G>C, g.chr7:140453136A>T, g.chr7:148113314G>A | Skin |
| DO220911 | RNF43, MUC16, MUC16, LIFR, MACC1, BRAF, CDKN2A, DDX46 | g.chr17:56435092G>A, g.chr19:9084835G>A, g.chr19:9087904G>A, g.chr5:38527329C>T, g.chr7:20177809C>T, g.chr7:140453136A>T, g.chr9:21971120G>A, g.chr5:134094502delC | Skin |
| DO220912 | KNSTRN, MUC16, ERBB4, ERBB4, PTPRT, BRAF, CNTNAP2, CNTNAP2, CSMD3, CSMD3, PPP6C, PTEN | g.chr15:40675107C>T, g.chr19:9069548C>T, g.chr2:212248293G>A, g.chr2:212495235C>T, g.chr20:40707448C>T, g.chr7:140481402C>T, g.chr7:148113202C>T, g.chr7:148116956G>A, g.chr8:113275983G>A, g.chr8:114326852C>T, g.chr9:127912080G>A, g.chr10:89693002_89693003insA | Skin |
| DO220913 | NRAS, MUC16, CDH10 | g.chr1:115256530G>T, g.chr19:9091402C>T, g.chr5:24488123G>A | Skin |
| DO221123 | MTOR, BTG1, BCL6, SGK1, BRAF, TMSB4X, TMSB4X, TMSB4X | g.chr1:11188078C>A, g.chr12:92539204C>T, g.chr3:187463242A>G, g.chr6:134495693G>A, g.chr7:140453136A>T, g.chrX:12993709C>G, g.chrX:12993758C>G, g.chrX:12993823C>T | Lymph |
| DO221124 | CREBBP, BCL2, BCL2, BCL2, BCL2, BCL2, BCL2, BCL2, BCL2, BCL2, BCL2, BCL2, BCL2, BCR | g.chr16:3786703T>A, g.chr18:60986163A>G, g.chr18:60986210C>T, g.chr18:60986300A>T, g.chr18:60986321A>G, g.chr18:60986323_60986324TT>GC, g.chr18:60986420G>A, g.chr18:60986442G>C, g.chr18:60986526A>G, g.chr18:60986652C>T, g.chr18:60986773G>A, g.chr18:60986793C>T, g.chr18:60986888C>G, g.chr22:23523353G>C | Lymph |
| DO221129 | CD79B, MYD88 | g.chr17:62006798T>C, g.chr3:38182641T>C | Lymph |
| DO221539 | KRAS, TP53 | g.chr12:25398285C>G, g.chr17:7577094G>A | Panc |
| DO221540 | KRAS, PBRM1 | g.chr12:25398284C>T, g.chr3:52678783_52678784insT | Panc |
| DO221541 | KRAS, B2M | g.chr12:25398284C>T, g.chr15:45003786_45003789delTCTT | Panc |
| DO221542 | KRAS, TP53 | g.chr12:25398284C>T, g.chr17:7577120C>T | Panc |
| DO221543 | KRAS | g.chr12:25398285C>G | Panc |
| DO221544 | KRAS, SF3B1 | g.chr12:25380276T>A, g.chr2:198266834T>C | Panc |
| DO221545 | KRAS, AFF3 | g.chr12:25398284C>T, g.chr2:100167832G>A | Panc |
| DO221546 | KRAS, CDKN2A | g.chr12:25398284C>T, g.chr9:21974715_21974732delGCAGCGCCCCCGCCTCCA | Panc |
| DO221547 | KRAS | g.chr12:25398284C>T | Panc |
| DO221548 | NRAS | g.chr1:115256528T>G | Myeloid |
| DO222299 | TP53, BCL11A | g.chr17:7577097C>T, g.chr2:60687244G>A | Stomach |
| DO23508 | TP53 | g.chr17:7578461C>A | Liver |
| DO23509 | TP53, NUP214 | g.chr17:7578235T>C, g.chr9:134000975delG | Liver |
| DO23513 | TP53 | g.chr17:7577574T>C | Liver |
| DO23514 | CTNNB1 | g.chr3:41266110A>C | Liver |
| DO23517 | CDH11 | g.chr16:64980577A>G | Liver |
| DO23518 | CTNNB1 | g.chr3:41266104G>A | Liver |
| DO23522 | PPP2R2A | g.chr8:26228523_26228527delTAAAG | Liver |
| DO23525 | CDKN2A | g.chr9:21971120G>A | Liver |
| DO23526 | TP53 | g.chr17:7574018G>A | Liver |
| DO23527 | CTNNB1 | g.chr3:41266136T>C | Liver |
| DO23529 | CTNNB1 | g.chr3:41266100T>C | Liver |
| DO23534 | CTNNB1 | g.chr3:41266124A>G | Liver |
| DO23535 | TP53 | g.chr17:7577102C>T | Liver |
| DO23539 | TP53 | g.chr17:7578190T>C | Liver |
| DO23542 | CTNNB1 | g.chr3:41266137C>T | Liver |
| DO23543 | CTNNB1, TBL1XR1, CTNNB1 | g.chr3:41266098A>T, g.chr3:176771665T>C, g.chr3:41266100T>C | Liver |
| DO23545 | TP53 | g.chr17:7578542G>A | Liver |
| DO23548 | CTNNB1 | g.chr3:41266101C>G | Liver |
| DO23549 | CTNNB1 | g.chr3:41266124A>G | Liver |
| DO23550 | TP53 | g.chr17:7577570C>T | Liver |
| DO23551 | TP53 | g.chr17:7578212G>A | Liver |
| DO27763 | ID3, SMARCA4, MYC | g.chr1:23885677G>A, g.chr19:11134252G>A, g.chr8:128748858G>A | Lymph |
| DO27764 | RHOA | g.chr3:49413009C>T | Lymph |
| DO27765 | CCND3 | g.chr6:41903688A>T | Lymph |
| DO27767 | ID3, FBXO11, RHOA, BCL6, MYC, ZFHX3, PHOX2B | g.chr1:23885677G>A, g.chr2:48040518T>C, g.chr3:49413009C>T, g.chr3:187463212T>C, g.chr8:128748858G>A, g.chr16:72818686_72818687insA, g.chr4:41746280delA | Lymph |
| DO27769 | TP53, RHOA, MYC, MYC | g.chr17:7577120C>T, g.chr3:49413009C>T, g.chr8:128748832C>T, g.chr8:128748858G>A | Lymph |
| DO27771 | IGHJ6 | g.chr14:106329791_106329796delTGAGAA | Lymph |
| DO27773 | BCL2 | g.chr18:60986082T>G | Lymph |
| DO27775 | TP53, MYC | g.chr17:7577120C>T, g.chr8:128751036G>A | Lymph |
| DO27779 | SOCS1, BCL6, PIM1, SGK1, SGK1, KIAA1549, TMSB4X, CDH10 | g.chr16:11349141C>T, g.chr3:187463255C>T, g.chr6:37139063G>A, g.chr6:134495654G>A, g.chr6:134495725C>T, g.chr7:138554308G>A, g.chrX:12993637C>T, g.chr5:24487658_24487659insA | Lymph |
| DO27783 | NOTCH2, KMT2D, BCL6, TMSB4X, TMSB4X | g.chr1:120458147G>A, g.chr12:49441770T>C, g.chr3:187463261A>T, g.chrX:12993554C>T, g.chrX:12993637C>T | Lymph |
| DO27785 | CREBBP, BCL2, BCL2, BCL2, BCL2, BCL2, BCL2, BCL2 | g.chr16:3786704A>G, g.chr18:60985444C>T, g.chr18:60985916G>C, g.chr18:60985997T>C, g.chr18:60986280A>G, g.chr18:60986420G>A, g.chr18:60986669G>T, g.chr18:60986888C>T | Lymph |
| DO27787 | CREBBP, BCL2, BCL2 | g.chr16:3786704A>C, g.chr18:60985916G>A, g.chr18:60986442G>C | Lymph |
| DO27789 | TMSB4X, TMSB4X | g.chrX:12993999G>A, g.chrX:12994443_12994444delAG | Lymph |
| DO27791 | CARD11, EZH2 | g.chr7:2985459T>C, g.chr7:148508728A>G | Lymph |
| DO27793 | ID3, MYC, RPL10 | g.chr1:23885728G>A, g.chr8:128750953C>G, g.chrX:153627713T>C | Lymph |
| DO27795 | TP53, SMARCA4, RPL10 | g.chr17:7577548C>T, g.chr19:11144146C>T, g.chrX:153627713T>C | Lymph |
| DO27797 | BCL2, BCL2, BCL2, BCL2, BCL2, BCL2 | g.chr18:60986069T>C, g.chr18:60986144A>T, g.chr18:60986148G>A, g.chr18:60986210C>T, g.chr18:60986280A>G, g.chr18:60986669G>T | Lymph |
| DO27799 | WIF1 | g.chr12:65444775G>A | Lymph |
| DO27801 | STAT6, BCL2, BCL2, BCL2, BCL2, TMSB4X | g.chr12:57496671C>G, g.chr18:60985760C>T, g.chr18:60985793C>T, g.chr18:60986744G>A, g.chr18:60986926T>C, g.chrX:12993823C>T | Lymph |
| DO27803 | BCL2, BCL2, BCL2, BCL2, BCL2, BCL2, BCL2, BCL2, BCL2, BCL2, BCL2, EZH2 | g.chr18:60985916G>A, g.chr18:60986138G>A, g.chr18:60986278G>C, g.chr18:60986331A>C, g.chr18:60986382C>T, g.chr18:60986516C>G, g.chr18:60986697T>C, g.chr18:60986805T>C, g.chr18:60986888C>G, g.chr18:60986926T>C, g.chr18:60986968T>C, g.chr7:148508727T>A | Lymph |
| DO27805 | BCL2, BCL2, BCL2, BCL2, BCL2, BCL2, BCL2, BCL2, BCL2, FAM135B | g.chr18:60985492C>T, g.chr18:60985905C>T, g.chr18:60986138G>C, g.chr18:60986192G>C, g.chr18:60986382C>T, g.chr18:60986420G>C, g.chr18:60986423C>T, g.chr18:60986906T>G, g.chr18:60986968T>C, g.chr8:139180251G>A | Lymph |
| DO27807 | TMSB4X | g.chrX:12993910C>T | Lymph |
| DO27809 | BCL2, BCL2, BCL2, BCL2, BCL2, BCL2, BCL2, SGK1, CSMD3 | g.chr18:60985562G>C, g.chr18:60985834C>T, g.chr18:60986152G>A, g.chr18:60986195T>A, g.chr18:60986279T>G, g.chr18:60986526A>G, g.chr18:60986917G>A, g.chr6:134495725C>T, g.chr8:113323333G>T | Lymph |
| DO27811 | BTG2, BCL6 | g.chr1:203274876G>A, g.chr3:187463242A>G | Lymph |
| DO27815 | CCND3 | g.chr6:41903745_41903746insG | Lymph |
| DO27817 | CREBBP, BCL2 | g.chr16:3786748G>A, g.chr18:60986652C>T | Lymph |
| DO27819 | MUC16, MYC | g.chr19:9056233C>T, g.chr8:128750953C>G | Lymph |
| DO27821 | TP53, SMARCA4, FBXO11 | g.chr17:7577560A>G, g.chr19:11144113G>A, g.chr2:48040518T>C | Lymph |
| DO27825 | TP53, SMARCA4, CARD11, MYC, MYC | g.chr17:7577538C>T, g.chr19:11134252G>A, g.chr7:2979559C>T, g.chr8:128748551G>A, g.chr8:128748863A>G | Lymph |
| DO27827 | SGK1, CNTNAP2, TMSB4X | g.chr6:134495693G>A, g.chr7:148116956G>A, g.chrX:12994220C>T | Lymph |
| DO27829 | ID3, CCND3, P2RY8, TP53 | g.chr1:23885728G>A, g.chr6:41903688A>T, g.chrX:1584907G>A, g.chr17:7577086_7577087delTG | Lymph |
| DO27833 | TP53, XPO1, PIM1 | g.chr17:7577120C>T, g.chr2:61719472C>T, g.chr6:37138355C>T | Lymph |
| DO27835 | STAT6, BCL2, BCL2, BCL2 | g.chr12:57498345C>T, g.chr18:60985974G>A, g.chr18:60986192G>C, g.chr18:60986386T>C | Lymph |
| DO27837 | BCL2, EZH2 | g.chr18:60986138G>C, g.chr7:148508727T>A | Lymph |
| DO27849 | KMT2D, EZH2, TMSB4X | g.chr12:49426613G>A, g.chr7:148508728A>T, g.chrX:12993674C>T | Lymph |
| DO27851 | SOCS1, TP53, SMARCA4, IDH1, TGFBR2, TMSB4X, B2M, RUNX1T1 | g.chr16:11349141C>T, g.chr17:7578508C>T, g.chr19:11134252G>A, g.chr2:209113112C>T, g.chr3:30732956C>T, g.chrX:12993410G>A, g.chr15:45003781_45003782delCT, g.chr8:92967699_92967700insA | Lymph |
| DO27853 | NOTCH2, BCL6, PIM1, PIM1, SGK1, CSMD3, TMSB4X, TMSB4X, IGHM | g.chr1:120458147G>A, g.chr3:187463255C>T, g.chr6:37138937C>T, g.chr6:37139077G>A, g.chr6:134495724G>A, g.chr8:114449220C>T, g.chrX:12993637C>T, g.chrX:12994364G>C, g.chr14:106323408delG | Lymph |
| DO27855 | BTG1, TMSB4X | g.chr12:92539204C>T, g.chrX:12993910C>T | Lymph |
| DO27857 | BCL2, BCL2, BCL2, BCL2, BCL2, BCL2, MYD88, EZH2, ZFHX3 | g.chr18:60985833G>A, g.chr18:60986189A>G, g.chr18:60986205A>G, g.chr18:60986243A>C, g.chr18:60986444T>C, g.chr18:60986888C>T, g.chr3:38182032C>G, g.chr7:148508727T>A, g.chr16:72818686_72818687insA | Lymph |
| DO27859 | KMT2D, BCL2, BCL2, BCL2, EBF1 | g.chr12:49441770T>C, g.chr18:60985833G>A, g.chr18:60986516C>G, g.chr18:60986744G>A, g.chr5:158526445G>A | Lymph |
| DO32831 | KRAS, TP53 | g.chr12:25398284C>T, g.chr17:7578197delC | Panc |
| DO32833 | KRAS | g.chr12:25398284C>T | Panc |
| DO32837 | KRAS, TP53 | g.chr12:25398284C>T, g.chr17:7578406C>T | Panc |
| DO32860 | KRAS, TP53 | g.chr12:25398284C>T, g.chr17:7578266T>A | Panc |
| DO32863 | KRAS | g.chr12:25398284C>T | Panc |
| DO32875 | KRAS | g.chr12:25398284C>T | Panc |
| DO32878 | KRAS, CDKN2A | g.chr12:25398284C>A, g.chr9:21970969A>T | Panc |
| DO32893 | KRAS, TP53 | g.chr12:25398284C>A, g.chr17:7577094G>A | Panc |
| DO32900 | KRAS, TP53 | g.chr12:25398284C>T, g.chr17:7578445A>T | Panc |
| DO32916 | KRAS | g.chr12:25398285C>G | Panc |
| DO32972 | KRAS | g.chr12:25398284C>T | Panc |
| DO32980 | TP53 | g.chr17:7578474_7578475insG | Panc |
| DO32984 | KRAS, CDKN2A | g.chr12:25398285C>G, g.chr9:21971120G>A | Panc |
| DO33000 | KRAS, TP53 | g.chr12:25398284C>A, g.chr17:7578246_7578247insA | Panc |
| DO33008 | KRAS, TP53 | g.chr12:25398284C>A, g.chr17:7578508C>T | Panc |
| DO33016 | GNAS | g.chr20:57484420C>T | Panc |
| DO33028 | KRAS | g.chr12:25398284C>T | Panc |
| DO33032 | TP53 | g.chr17:7578388C>T | Panc |
| DO33042 | KRAS, TP53 | g.chr12:25398284C>T, g.chr17:7577538C>T | Panc |
| DO33128 | KRAS, TP53 | g.chr12:25398284C>T, g.chr17:7578212G>A | Panc |
| DO33152 | KRAS, CDKN2A, DDR2, ACVR2A, ACVR2A | g.chr12:25398285C>G, g.chr9:21971028C>T, g.chr1:162752532_162752533insT, g.chr2:148683685_148683686insA, g.chr2:148683686delA | Panc |
| DO33160 | KRAS, TP53 | g.chr12:25398285C>G, g.chr17:7578461C>A | Panc |
| DO33168 | KRAS, TP53, PABPC1 | g.chr12:25398285C>G, g.chr17:7577120C>T, g.chr8:101734603T>G | Panc |
| DO33184 | KRAS | g.chr12:25398284C>A | Panc |
| DO33200 | PDE4DIP, KRAS, TP53 | g.chr1:144879284G>A, g.chr12:25398284C>T, g.chr17:7577121G>A | Panc |
| DO33208 | KRAS, TP53 | g.chr12:25380275T>G, g.chr17:7577120C>T | Panc |
| DO33248 | KRAS, TP53 | g.chr12:25398285C>G, g.chr17:7578474_7578475insG | Panc |
| DO33264 | KRAS, CTNNA2, GNAS, PIK3CA | g.chr12:25398284C>T, g.chr2:79971659A>G, g.chr20:57484421G>A, g.chr3:178952085A>G | Panc |
| DO33288 | KRAS | g.chr12:25398284C>T | Panc |
| DO33344 | KRAS, TP53 | g.chr12:25398284C>T, g.chr17:7578212G>A | Panc |
| DO33368 | KRAS, MUC16 | g.chr12:25398284C>T, g.chr19:9046028G>A | Panc |
| DO33376 | KRAS, TP53 | g.chr12:25398285C>G, g.chr17:7577058C>A | Panc |
| DO33392 | KRAS, TP53 | g.chr12:25380275T>G, g.chr17:7577121G>A | Panc |
| DO33400 | KRAS, TP53 | g.chr12:25398285C>G, g.chr17:7577094G>A | Panc |
| DO33408 | KRAS, TP53 | g.chr12:25398284C>T, g.chr17:7578190T>C | Panc |
| DO33472 | KRAS | g.chr12:25398284C>A | Panc |
| DO33480 | KRAS | g.chr12:25380275T>G | Panc |
| DO33488 | GNAS | g.chr20:57484420C>T | Panc |
| DO33512 | KRAS, TP53 | g.chr12:25398285C>G, g.chr17:7577535C>A | Panc |
| DO33544 | KRAS, TP53 | g.chr12:25380275T>G, g.chr17:7578206T>C | Panc |
| DO33552 | KRAS | g.chr12:25398284C>T | Panc |
| DO33600 | KRAS, PTPRT, TP53 | g.chr12:25398284C>T, g.chr20:40708927C>T, g.chr17:7578246_7578247insA | Panc |
| DO33960 | KRAS | g.chr12:25398284C>T | Panc |
| DO33984 | KRAS | g.chr12:25398284C>A | Panc |
| DO34264 | KRAS | g.chr12:25398285C>G | Panc |
| DO34288 | KRAS | g.chr12:25398285C>G | Panc |
| DO34312 | KRAS, TP53, CDKN2A, MED12 | g.chr12:25398284C>T, g.chr17:7578445A>T, g.chr9:21971120G>A, g.chrX:70349258C>T | Panc |
| DO34336 | KRAS | g.chr12:25398285C>G | Panc |
| DO34432 | BRAF | g.chr7:140477837_140477851delTAGGTGCTGTCACAT | Panc |
| DO34448 | KRAS, ZNF521 | g.chr12:25398284C>A, g.chr18:22806441A>C | Panc |
| DO34504 | TP53, FAT4, BRAF | g.chr17:7577094G>A, g.chr4:126411849C>T, g.chr7:140477837_140477851delTAGGTGCTGTCACAT | Panc |
| DO34600 | KRAS | g.chr12:25398284C>T | Panc |
| DO34608 | KRAS, TP53 | g.chr12:25398284C>A, g.chr17:7578190T>C | Panc |
| DO34616 | KRAS, TP53 | g.chr12:25398284C>A, g.chr17:7577094G>A | Panc |
| DO34640 | KRAS | g.chr12:25398284C>T | Panc |
| DO34656 | KRAS, SMAD4, TP53 | g.chr12:25398284C>T, g.chr18:48575152C>T, g.chr17:7578213_7578214delAA | Panc |
| DO34680 | KRAS | g.chr12:25398284C>A | Panc |
| DO34696 | KRAS | g.chr12:25398284C>A | Panc |
| DO34720 | KRAS, TP53 | g.chr12:25398284C>T, g.chr17:7578395G>A | Panc |
| DO34728 | KRAS, TP53, CCR7, CDKN2A | g.chr12:25398285C>G, g.chr17:7578205C>T, g.chr17:38715177C>T, g.chr9:21971120G>A | Panc |
| DO34736 | KRAS, TP53, CEP170P1 | g.chr12:25398284C>A, g.chr17:7578208T>C, g.chr4:119466233delC | Panc |
| DO34785 | KRAS, TP53, FAT4 | g.chr12:25398285C>G, g.chr17:7578555C>A, g.chr4:126411849C>T | Panc |
| DO34793 | KRAS, TP53 | g.chr12:25398284C>T, g.chr17:7574018G>A | Panc |
| DO34801 | KRAS | g.chr12:25398284C>T | Panc |
| DO34809 | KRAS | g.chr12:25398284C>A | Panc |
| DO34817 | KRAS | g.chr12:25398285C>G | Panc |
| DO34849 | KRAS, CHST11 | g.chr12:25398285C>G, g.chr12:105150997G>A | Panc |
| DO34905 | KRAS, ARID1A | g.chr12:25398284C>T, g.chr1:27105930_27105931insG | Panc |
| DO35082 | KRAS | g.chr12:25380275T>G | Panc |
| DO35083 | BCL9, KRAS, HMGN2P46, TP53, CD209, MUC16, PTPN13, CDKN2A, CDKN2A, RPL22, LARP4B, BCL11B, MYO5A, LYL1, ASXL2, ACVR2A, N4BP2, MLLT3, DDX3X, NONO | g.chr1:147091961G>A, g.chr12:25398284C>A, g.chr15:45843418C>T, g.chr17:7579312C>T, g.chr19:7806922C>T, g.chr19:9071434G>A, g.chr4:87610220C>T, g.chr9:21971120G>A, g.chr9:21971186G>A, g.chr1:6257785delT, g.chr10:890939delT, g.chr14:99636599delT, g.chr15:52603368delA, g.chr19:13209934delT, g.chr2:25966872_25966873insG, g.chr2:148683686delA, g.chr4:40158194delT, g.chr9:20622295delC, g.chrX:41207100delT, g.chrX:70520707delA | Panc |
| DO35085 | KRAS, TP53 | g.chr12:25398284C>T, g.chr17:7578275G>A | Panc |
| DO35098 | KRAS, KMT2C | g.chr12:25398285C>G, g.chr7:151882660C>A | Panc |
| DO35116 | KRAS, SMAD4, HLA-A | g.chr12:25398284C>A, g.chr18:48603032C>T, g.chr6:29910685G>C | Panc |
| DO35118 | KRAS | g.chr12:25398284C>T | Panc |
| DO35126 | KRAS, TP53 | g.chr12:25398284C>A, g.chr17:7573999delT | Panc |
| DO35128 | KRAS, TP53 | g.chr12:25398284C>A, g.chr17:7578263G>A | Panc |
| DO35132 | KRAS | g.chr12:25398284C>T | Panc |
| DO35136 | KRAS, SMAD4 | g.chr12:25380276T>C, g.chr18:48591888G>C | Panc |
| DO35138 | BRAF | g.chr7:140477837_140477851delTAGGTGCTGTCACAT | Panc |
| DO35144 | KRAS, CDKN2A | g.chr12:25398284C>A, g.chr9:21974777_21974780delGCCA | Panc |
| DO35148 | KRAS, TP53 | g.chr12:25398284C>T, g.chr17:7577094G>A | Panc |
| DO35152 | KRAS, TP53 | g.chr12:25398284C>T, g.chr17:7576897G>A | Panc |
| DO35184 | KRAS, CDKN2A | g.chr12:25398284C>T, g.chr9:21970969A>T | Panc |
| DO35186 | KRAS | g.chr12:25398284C>A | Panc |
| DO35198 | KRAS | g.chr12:25398284C>A | Panc |
| DO35200 | KRAS, TP53 | g.chr12:25398284C>A, g.chr17:7577094G>A | Panc |
| DO35216 | KRAS, SF3B1 | g.chr12:25398284C>A, g.chr2:198266834T>C | Panc |
| DO35222 | KRAS | g.chr12:25380275T>A | Panc |
| DO35228 | KRAS, TP53, SMAD4 | g.chr12:25398284C>T, g.chr17:7577153C>A, g.chr18:48603032C>T | Panc |
| DO35230 | KRAS | g.chr12:25398284C>T | Panc |
| DO35236 | KRAS, TP53, CDKN2A | g.chr12:25380275T>G, g.chr17:7579315_7579316insC, g.chr9:21974715_21974732delGCAGCGCCCCCGCCTCCA | Panc |
| DO35258 | KRAS, TP53 | g.chr12:25398284C>T, g.chr17:7577141C>T | Panc |
| DO35290 | KRAS | g.chr12:25398285C>G | Panc |
| DO35305 | KRAS | g.chr12:25398284C>A | Panc |
| DO35350 | KRAS, RP11-1129I3.1 | g.chr12:25398285C>G, g.chr15:56837521_56837522delTT | Panc |
| DO35360 | KRAS | g.chr12:25398285C>G | Panc |
| DO35376 | KRAS, TP53 | g.chr12:25398284C>T, g.chr17:7577124C>T | Panc |
| DO35406 | KRAS, TP53 | g.chr12:25398284C>T, g.chr17:7578555C>T | Panc |
| DO35424 | KRAS, TP53 | g.chr12:25398284C>A, g.chr17:7574003G>A | Panc |
| DO35442 | CCR7, SMARCA4, SMARCA4, GNAS, CSMD3, BMPR1A, LEF1, EXT1 | g.chr17:38715177C>T, g.chr19:11144113G>A, g.chr19:11144146C>T, g.chr20:57484421G>A, g.chr8:114326852C>T, g.chr10:88683715delT, g.chr4:108969353delA, g.chr8:119123617delA | Panc |
| DO35454 | KRAS, TP53 | g.chr12:25398285C>G, g.chr17:7578212G>A | Panc |
| DO35496 | KRAS | g.chr12:25380275T>G | Panc |
| DO35544 | AC007040.8 | g.chr2:71254415_71254421delTAGGATA | CNS |
| DO35547 | SMO | g.chr7:128846398C>T | CNS |
| DO35555 | SMARCA4, CTNNB1 | g.chr19:11144113G>A, g.chr3:41266113C>A | CNS |
| DO35568 | CTNNB1 | g.chr3:41266103G>A | CNS |
| DO35582 | CTNNB1 | g.chr3:41266101C>G | CNS |
| DO35620 | PIK3CA | g.chr3:178952085A>G | CNS |
| DO35704 | EED | g.chr11:85956145_85956146insG | CNS |
| DO35733 | ROBO2 | g.chr3:77147468G>A | CNS |
| DO35753 | CDH11 | g.chr16:64980684_64980685insA | CNS |
| DO35937 | FGFR1 | g.chr8:38272308T>C | CNS |
| DO35982 | FGFR1 | g.chr8:38274849G>T | CNS |
| DO35994 | BRAF | g.chr7:140453136A>T | CNS |
| DO36009 | FGFR1 | g.chr8:38272308T>C | CNS |
| DO36030 | FGFR1 | g.chr8:38274849G>T | CNS |
| DO36080 | PIK3CA | g.chr3:178917478G>A | CNS |
| DO36147 | BRAF | g.chr7:140453136A>T | CNS |
| DO36163 | BRAF | g.chr7:140453136A>T | CNS |
| DO36171 | FGFR1 | g.chr8:38274849G>T | CNS |
| DO36221 | KLK2, SF3B1, MB21D2, KIT, SDHA, PDGFRB, KLK2, SF3B1, MB21D2, KIT, PDGFRB | g.chr19:51383603G>A, g.chr2:198266834T>C, g.chr3:192514964A>C, g.chr4:55561845C>A, g.chr5:218471A>G, g.chr5:149535179G>T, g.chr19:51383603G>A, g.chr2:198266834T>C, g.chr3:192514964A>C, g.chr4:55561845C>A, g.chr5:149535179G>T | Prost |
| DO36223 | MED12, ATM, ZCCHC8, CHD2, MUC16, NSD1, ATM, ZCCHC8, CHD2, MUC16, NSD1 | g.chrX:70349258C>T, g.chr11:108160408T>C, g.chr12:122977299T>C, g.chr15:93569143G>T, g.chr19:9074443C>T, g.chr5:176723558G>T, g.chr11:108160408T>C, g.chr12:122977299T>C, g.chr15:93569143G>T, g.chr19:9074443C>T, g.chr5:176723558G>T | Prost |
| DO44740 | CTNNB1, NONO | g.chr3:41266137C>T, g.chrX:70520707delA | Liver |
| DO44806 | CTNNB1 | g.chr3:41266113C>G | Liver |
| DO44828 | CTNNB1 | g.chr3:41266101C>G | Liver |
| DO44832 | CTNNB1 | g.chr3:41266098A>G | Liver |
| DO45035 | TP53 | g.chr17:7578555C>T | Liver |
| DO45039 | TP53, CTNNB1 | g.chr17:7578393A>T, g.chr3:41266113C>T | Liver |
| DO45041 | TP53, CTNNB1, SND1 | g.chr17:7578479G>C, g.chr3:41266124A>G, g.chr7:127341308C>T | Liver |
| DO45047 | TP53 | g.chr17:7578403C>A | Liver |
| DO45049 | CCNB1IP1 | g.chr14:20781884T>C | Liver |
| DO45055 | PAX5 | g.chr9:36834661C>T | Liver |
| DO45057 | TP53 | g.chr17:7577581A>G | Liver |
| DO45064 | CTNNB1, CTA-85E5.10 | g.chr3:41266113C>A, g.chr22:30453761delC | Liver |
| DO45065 | TP53 | g.chr17:7578197delC | Liver |
| DO45067 | CTNNB1, PIK3CA | g.chr3:41266104G>A, g.chr3:178936091G>A | Liver |
| DO45071 | TP53, CTNNB1, ANK1 | g.chr17:7578266T>A, g.chr3:41266104G>A, g.chr8:41566407G>A | Liver |
| DO45073 | CTNNB1 | g.chr3:41266101C>G | Liver |
| DO45075 | TP53, MAF | g.chr17:7577082C>T, g.chr16:79630783_79630784insT | Liver |
| DO45077 | CTNNB1 | g.chr3:41266113C>G | Liver |
| DO45079 | FLT4 | g.chr5:180028508C>T | Liver |
| DO45081 | PREX2 | g.chr8:69145730G>A | Liver |
| DO45083 | TP53, CTNNB1 | g.chr17:7578190T>C, g.chr3:41266124A>G | Liver |
| DO45091 | TP53 | g.chr17:7578208T>C | Liver |
| DO45093 | TP53 | g.chr17:7577117A>G | Liver |
| DO45094 | PICALM, CTNNB1 | g.chr11:85685824G>A, g.chr3:41266136T>C | Liver |
| DO45096 | ATM, ATM, HNF4A, LINC00649 | g.chr11:108122587_108122588delTG, g.chr11:108122587_108122588delTG, g.chr20:43047101_43047102delCT, g.chr21:35352038G>A | Liver |
| DO45097 | CTNNB1 | g.chr3:41266103G>C | Liver |
| DO45115 | TCF7L2 | g.chr10:114926552G>A | Liver |
| DO45117 | CTNNB1 | g.chr3:41266097G>T | Liver |
| DO45121 | DDR2 | g.chr1:162745609C>T | Liver |
| DO45123 | KLK2 | g.chr19:51382261G>C | Liver |
| DO45127 | CTNNB1 | g.chr3:41266097G>A | Liver |
| DO45129 | CTNNB1 | g.chr3:41266098A>T | Liver |
| DO45133 | CTNNB1 | g.chr3:41266101C>G | Liver |
| DO45141 | CTNNB1, RUSC2 | g.chr3:41266113C>G, g.chr9:35561837_35561838insTATT | Liver |
| DO45149 | KMT2D | g.chr12:49426613G>A | Liver |
| DO45153 | ANK1 | g.chr8:41566407G>A | Liver |
| DO45159 | RUNX1T1 | g.chr8:92972737C>T | Liver |
| DO45161 | CTNNB1, PWWP2A, TP53 | g.chr3:41266136T>C, g.chr5:159519495T>C, g.chr17:7578397_7578398insG | Liver |
| DO45171 | CTNNB1 | g.chr3:41266137C>T | Liver |
| DO45173 | TP53 | g.chr17:7578542G>A | Liver |
| DO45177 | CTNNB1, PIK3CA | g.chr3:41266124A>G, g.chr3:178952085A>G | Liver |
| DO45179 | CTNNB1 | g.chr3:41266137C>A | Liver |
| DO45183 | NRAS | g.chr1:115256529T>C | Liver |
| DO45185 | TP53 | g.chr17:7578442T>C | Liver |
| DO45189 | STAG1 | g.chr3:136056728_136056729insT | Liver |
| DO45197 | ATRX | g.chrX:76938401_76938402delTT | Liver |
| DO45199 | ARID2, CTNNB1 | g.chr12:46287446C>T, g.chr3:41266124A>G | Liver |
| DO45201 | PHOX2B | g.chr4:41747895C>T | Liver |
| DO45207 | ERBB4 | g.chr2:212247212C>T | Liver |
| DO45251 | KRAS | g.chr12:25380275T>G | Biliary |
| DO45255 | CTNNB1, ROBO2 | g.chr3:41266101C>A, g.chr3:77147467C>T | Liver |
| DO45259 | ARID2 | g.chr12:46287315C>T | Biliary |
| DO45265 | TP53 | g.chr17:7574017C>A | Liver |
| DO45267 | TP53, CTNNB1 | g.chr17:7578493C>T, g.chr3:41266098A>G | Liver |
| DO45273 | TP53 | g.chr17:7578206T>C | Liver |
| DO45277 | ARID2 | g.chr12:46287315C>T | Liver |
| DO45281 | CTNNB1 | g.chr3:41266113C>T | Liver |
| DO45287 | TRRAP, RPL22 | g.chr7:98522789C>T, g.chr1:6257785delT | Biliary |
| DO45293 | PTEN | g.chr10:89692911G>T | Biliary |
| DO45297 | KRAS, SF3B1 | g.chr12:25398284C>A, g.chr2:198266834T>C | Biliary |
| DO45299 | TP53, GNAS, RUNX1T1, RALGDS, LARP4B, PTEN, KDM5A, MYO5A, CIITA, SPECC1, STAT5B, SMARCA4, PPP2R1A, ASXL2, REL, ACVR2A, ACVR2A, STAG1, WWTR1, LPP, LEF1, HNRNPA2B1, CSMD3, EXT1, EXT1, DDX3X, BCORL1 | g.chr17:7577121G>A, g.chr20:57430990C>T, g.chr8:92970812T>A, g.chr9:135984123G>A, g.chr10:890939delT, g.chr10:89726584_89726585insA, g.chr12:390703delA, g.chr15:52603368delA, g.chr16:11022451delC, g.chr17:20108263delA, g.chr17:40352476_40352477insT, g.chr19:11172713_11172714delAG, g.chr19:52729587delT, g.chr2:25966872_25966873insG, g.chr2:61154081_61154082insA, g.chr2:148683685_148683686insA, g.chr2:148683686delA, g.chr3:136056728_136056729insT, g.chr3:149237163delT, g.chr3:188598162delA, g.chr4:108969353delA, g.chr7:26231741delT, g.chr8:113236642delT, g.chr8:118808897delT, g.chr8:119123617delA, g.chrX:41207100delT, g.chrX:129190011delC | Biliary |
| DO45303 | PIK3CA | g.chr3:178936082G>A | Biliary |
| DO45305 | IDH1 | g.chr2:209113113G>A | Biliary |
| DO46327 | TP53 | g.chr17:7577124C>T | Ovary |
| DO46328 | TP53 | g.chr17:7578394T>C | Ovary |
| DO46330 | TP53, JAK3, LRP1B, FAT4 | g.chr17:7577120C>T, g.chr19:17935777G>A, g.chr2:141027892G>C, g.chr4:126240900G>A | Ovary |
| DO46331 | TP53 | g.chr17:7577094G>A | Ovary |
| DO46333 | TP53 | g.chr17:7578449C>T | Ovary |
| DO46334 | TP53 | g.chr17:7577120C>T | Ovary |
| DO46338 | TP53 | g.chr17:7577120C>T | Ovary |
| DO46350 | TP53 | g.chr17:7579315_7579316insC | Ovary |
| DO46352 | TP53 | g.chr17:7578416C>A | Ovary |
| DO46354 | TP53 | g.chr17:7577538C>T | Ovary |
| DO46356 | TP53 | g.chr17:7578265A>G | Ovary |
| DO46358 | TP53 | g.chr17:7577120C>A | Ovary |
| DO46362 | TP53 | g.chr17:7578442T>C | Ovary |
| DO46366 | TP53 | g.chr17:7577082C>T | Ovary |
| DO46372 | TP53 | g.chr17:7578493C>T | Ovary |
| DO46374 | TP53 | g.chr17:7578416C>A | Ovary |
| DO46376 | TP53 | g.chr17:7578190T>C | Ovary |
| DO46378 | TP53, GNAS | g.chr17:7574003G>A, g.chr20:57430058G>A | Ovary |
| DO46380 | TP53 | g.chr17:7577559G>A | Ovary |
| DO46388 | TP53 | g.chr17:7573999delT | Ovary |
| DO46390 | TP53 | g.chr17:7577120C>T | Ovary |
| DO46396 | TP53 | g.chr17:7577547C>T | Ovary |
| DO46398 | TP53, FAM135B | g.chr17:7577570C>T, g.chr8:139165111G>T | Ovary |
| DO46400 | IKZF1 | g.chr7:50468997C>T | Ovary |
| DO46402 | TP53, ZPLD1 | g.chr17:7578535T>C, g.chr3:101948028_101948032delAGCTT | Ovary |
| DO46404 | TP53 | g.chr17:7577105G>C | Ovary |
| DO46408 | TP53 | g.chr17:7578406C>T | Ovary |
| DO46412 | TP53, RP11-1103G16.1 | g.chr17:7578190T>C, g.chr14:30914903_30914907delGGCCT | Ovary |
| DO46416 | TP53 | g.chr17:7577058C>A | Ovary |
| DO46420 | TP53, REL | g.chr17:7578406C>T, g.chr2:61154081_61154082insA | Ovary |
| DO46424 | FAT3 | g.chr11:92086572G>A | Ovary |
| DO46448 | TP53 | g.chr17:7578275G>A | Ovary |
| DO46488 | LRRC4C | g.chr11:40136571delG | Ovary |
| DO46493 | TP53 | g.chr17:7577538C>T | Ovary |
| DO46542 | TP53 | g.chr17:7578265A>G | Ovary |
| DO46551 | TP53 | g.chr17:7578393A>T | Ovary |
| DO46561 | TP53 | g.chr17:7578190T>C | Ovary |
| DO46568 | TP53 | g.chr17:7578526C>A | Ovary |
| DO46571 | TP53 | g.chr17:7577120C>T | Ovary |
| DO46581 | TP53 | g.chr17:7577547C>T | Ovary |
| DO46586 | KRAS, TP53 | g.chr12:25398284C>A, g.chr17:7578275G>A | Ovary |
| DO46591 | TAL1, BAX, HOOK3 | g.chr1:47682037_47682038insT, g.chr19:49458970_49458971insG, g.chr8:42884886delT | Ovary |
| DO46597 | TP53 | g.chr17:7578265A>G | Ovary |
| DO46606 | TP53 | g.chr17:7578271T>C | Ovary |
| DO46611 | TP53 | g.chr17:7577570C>T | Ovary |
| DO46779 | DAXX | g.chr6:33288561C>A | Panc |
| DO46783 | TP53, CDKN2A | g.chr17:7578263G>A, g.chr9:21971186G>A | Panc |
| DO46787 | SF3B1 | g.chr2:198266834T>C | Prost |
| DO46792 | SPOP | g.chr17:47696426A>C | Prost |
| DO46795 | SPOP | g.chr17:47696425A>G | Prost |
| DO46832 | VHL | g.chr3:10183692_10183693insG | Kidney |
| DO46834 | DTX4 | g.chr11:58975864_58975868delTGGGA | Kidney |
| DO46838 | SNORA63 | g.chr7:64790950_64790954delGTTTG | Kidney |
| DO46877 | ZFHX3 | g.chr16:72993516C>T | Kidney |
| DO46881 | RP11-804A23.2 | g.chr11:60604801_60604810delTCTCAGTGGC | Kidney |
| DO46885 | VHL, RN7SKP104 | g.chr3:10183692_10183693insG, g.chr7:97228271delC | Kidney |
| DO46893 | TP53 | g.chr17:7578534C>A | Kidney |
| DO46897 | MUC16 | g.chr19:9056233C>T | Kidney |
| DO46909 | VHL | g.chr3:10183797T>A | Kidney |
| DO46913 | TP53 | g.chr17:7576852C>T | Kidney |
| DO46917 | VHL | g.chr3:10183797T>A | Kidney |
| DO46957 | PBRM1 | g.chr3:52678783_52678784insT | Kidney |
| DO46980 | PLEKHG6 | g.chr12:6420199delC | Kidney |
| DO47016 | H3F3A, VHL | g.chr1:226253357G>A, g.chr3:10188200C>T | Kidney |
| DO47048 | MTOR | g.chr1:11188078C>A | Kidney |
| DO47068 | VHL | g.chr3:10188200C>T | Kidney |
| DO47072 | PBRM1 | g.chr3:52678783_52678784insT | Kidney |
| DO47165 | VHL | g.chr3:10188200C>T | Kidney |
| DO47174 | MTOR | g.chr1:11188078C>A | Kidney |
| DO48541 | GNAS | g.chr20:57430265C>T | Panc |
| DO48557 | CTNNB1 | g.chr3:41266113C>G | Panc |
| DO48577 | REL, CREB1, PDGFRB, CREB1, REL, PDGFRB | g.chr2:61153726C>T, g.chr2:208468084G>T, g.chr5:149514372T>A, g.chr2:208468084G>T, g.chr2:61153726C>T, g.chr5:149514372T>A | Prost |
| DO48578 | PTEN, MEN1 | g.chr10:89720749delC, g.chr11:64577330_64577333delAGAC | Panc |
| DO48672 | CTNNB1 | g.chr3:41266097G>T | Liver |
| DO48679 | TP53, CTNNB1 | g.chr17:7578208T>C, g.chr3:41266113C>G | Liver |
| DO48682 | TP53, RPL22, PBX1, BMPR1A, BCL11B, MYO5A, SMAD2, LPP, IL6ST, BCORL1 | g.chr17:7577111G>C, g.chr1:6257785delT, g.chr1:164821059delA, g.chr10:88683715delT, g.chr14:99636599delT, g.chr15:52603368delA, g.chr18:45358789delA, g.chr3:188598162delA, g.chr5:55236037delT, g.chrX:129190011delC | Liver |
| DO48684 | TP53 | g.chr17:7577094G>A | Liver |
| DO48686 | CTNNB1 | g.chr3:41266124A>G | Liver |
| DO48689 | CTNNB1 | g.chr3:41266107T>G | Liver |
| DO48692 | KRAS, CTNNB1 | g.chr12:25380275T>G, g.chr3:41266124A>G | Liver |
| DO48695 | CD209 | g.chr19:7805166C>T | Liver |
| DO48703 | CTNNB1 | g.chr3:41266136T>C | Liver |
| DO48715 | TP53 | g.chr17:7577551C>A | Liver |
| DO48719 | LRP1B | g.chr2:141816617G>A | Liver |
| DO48721 | CTNNB1 | g.chr3:41266124A>G | Liver |
| DO48723 | BTG2 | g.chr1:203276414G>A | Liver |
| DO48727 | RP11-541P9.3 | g.chr5:162536524delA | Liver |
| DO48730 | TP53 | g.chr17:7578212G>A | Liver |
| DO48732 | PWWP2A | g.chr5:159519495T>C | Liver |
| DO48741 | TP53 | g.chr17:7577121G>A | Liver |
| DO48742 | CTNNB1 | g.chr3:41266136T>C | Liver |
| DO48751 | CTNNB1 | g.chr3:41266101C>G | Liver |
| DO48757 | TP53, CTNNB1 | g.chr17:7577610T>C, g.chr3:41266103G>A | Liver |
| DO48759 | CTNNB1 | g.chr3:41266103G>C | Liver |
| DO48760 | MUC16 | g.chr19:9076168G>A | Liver |
| DO48888 | TRRAP, SMO | g.chr7:98522789C>T, g.chr7:128846398C>T | CNS |
| DO48889 | PTEN | g.chr10:89692911G>T | CNS |
| DO48891 | DDX3X | g.chrX:41204458C>T | CNS |
| DO48893 | SETBP1 | g.chr18:42531907G>A | CNS |
| DO48895 | KMT2A | g.chr11:118348808G>A | CNS |
| DO48899 | FBXW7 | g.chr4:153249385G>A | CNS |
| DO48900 | MAX | g.chr14:65544747C>T | CNS |
| DO48909 | SMO | g.chr7:128846398C>T | CNS |
| DO48911 | IDH1 | g.chr2:209113113G>A | CNS |
| DO48914 | BCL9 | g.chr1:147091961G>A | CNS |
| DO48915 | PIK3CA, SMO | g.chr3:178952085A>T, g.chr7:128846398C>T | CNS |
| DO48939 | ARID2, DDX3X | g.chr12:46287446C>T, g.chrX:41204458C>T | CNS |
| DO48940 | IKZF1 | g.chr7:50367289G>A | CNS |
| DO48945 | IDH1, KMT2D, KMT2D | g.chr2:209113113G>A, g.chr12:49431019_49431021delGTG, g.chr12:49431019_49431021delGTG | CNS |
| DO48964 | SMARCA4, SMARCA4 | g.chr19:11132513C>T, g.chr19:11132542G>A | CNS |
| DO49074 | KRAS, TP53 | g.chr12:25380275T>A, g.chr17:7577120C>T | Panc |
| DO49076 | FAM47C | g.chrX:37029325G>A | Panc |
| DO49078 | KRAS | g.chr12:25398284C>A | Panc |
| DO49079 | KRAS, TP53, PABPC1 | g.chr12:25398284C>A, g.chr17:7578265A>G, g.chr8:101734603T>G | Panc |
| DO49080 | KRAS, CDKN2A | g.chr12:25398285C>A, g.chr9:21971028C>T | Panc |
| DO49087 | NRAS, TP53, GNAS | g.chr1:115258747C>T, g.chr17:7577538C>T, g.chr20:57484420C>T | Panc |
| DO49113 | KRAS, ZFHX3 | g.chr12:25398284C>A, g.chr16:72993516C>T | Panc |
| DO49127 | KRAS, TP53 | g.chr12:25398285C>G, g.chr17:7577105G>C | Panc |
| DO49129 | KRAS | g.chr12:25398284C>T | Panc |
| DO49135 | KRAS, TP53, CDKN2A | g.chr12:25398285C>G, g.chr17:7578176C>A, g.chr9:21971149_21971172delGGCTCCGCGCCGTGGAGCAGCAGC | Panc |
| DO49137 | KRAS | g.chr12:25398284C>T | Panc |
| DO49138 | KRAS, TP53, PIK3CA | g.chr12:25398284C>A, g.chr17:7578382G>C, g.chr3:178936082G>A | Panc |
| DO49166 | KRAS | g.chr12:25398284C>T | Panc |
| DO49168 | KRAS, TP53, FLT4 | g.chr12:25398284C>T, g.chr17:7577121G>A, g.chr5:180028508C>T | Panc |
| DO49172 | KRAS, TP53 | g.chr12:25398284C>T, g.chr17:7577094G>A | Panc |
| DO49178 | KRAS, TP53 | g.chr12:25380275T>A, g.chr17:7578542G>A | Panc |
| DO49181 | KRAS, TP53 | g.chr12:25398285C>G, g.chr17:7577094G>A | Panc |
| DO49183 | KRAS | g.chr12:25398284C>T | Panc |
| DO49184 | KRAS | g.chr12:25398285C>G | Panc |
| DO49193 | KRAS, TP53, SMAD4 | g.chr12:25398284C>T, g.chr17:7577538C>T, g.chr18:48603032C>T | Panc |
| DO49204 | KRAS, TP53 | g.chr12:25398284C>A, g.chr17:7578406C>T | Panc |
| DO49418 | TP53, TP53, APC, BAX | g.chr17:7574003G>A, g.chr17:7578388C>T, g.chr5:112175639C>T, g.chr19:49458970_49458971insG | Panc |
| DO49419 | KRAS, KIT | g.chr12:25398285C>G, g.chr4:55605608T>G | Panc |
| DO49420 | KRAS, TP53, PTPN13, FAT4 | g.chr12:25398284C>T, g.chr17:7578212G>A, g.chr4:87610220C>T, g.chr4:126240900G>A | Panc |
| DO49421 | TP53, GNAS | g.chr17:7577610T>C, g.chr20:57484421G>A | Panc |
| DO49422 | KRAS | g.chr12:25398284C>T | Panc |
| DO49424 | KRAS, TP53 | g.chr12:25398284C>A, g.chr17:7577120C>T | Panc |
| DO49427 | KRAS | g.chr12:25398285C>G | Panc |
| DO49433 | KRAS | g.chr12:25398284C>T | Panc |
| DO49436 | KRAS, TP53, DCAF12L2 | g.chr12:25398285C>G, g.chr17:7577548C>T, g.chrX:125298905G>A | Panc |
| DO49439 | KRAS, SF3B1 | g.chr12:25398284C>T, g.chr2:198266834T>C | Panc |
| DO49442 | PDE4DIP, H3F3A, HOXC11, ITGAV, PDE4DIP, H3F3A, HOXC11, ITGAV, AACSP1, AB015752.3, AC002485.1, AC004053.1, AC009312.1, AC012501.2, AC012671.1, AC013463.2, AC018737.1, AC018890.6, AC024560.3, AC079610.1, AC079610.1, AC079613.1, AC079756.1, AC091736.1, AC104434.1, AC108142.1, AC108448.3, AC108696.1, AC129929.5, AC131097.3, AC144521.1, ADD2, AJ003147.9, AJAP1, AL117380.1, AL133247.2, AL138963.1, AL590874.1, ANKRD19P, AP000640.2, ARPC5, B3GALTL, BDNF-AS, BMS1P8, BTAF1, C18orf8, C1orf132, C1orf132, CAMSAP3, CASC2, CASC2, CCNL1, CCR10, CDPF1, CLHC1, CNGB3, CNNM3, COPA, CST7, CTBP1-AS2, CTC-260E6.6, CTD-2203K17.1, CTD-2307P3.1, CTD-3006G17.2, CTH, CYCS, DCTN4, DEAF1, DGKI, DISC1FP1, DISC1FP1, DNER, DSTN, DTX4, EGF, EMCN-IT3, EOGT, FAM210B, FAM27E3, FAM3B, FAM86JP, FZD3, GBA3, GCM1, GGTA1P, GLTSCR2, GPR158-AS1, GS1-256O22.5, GTF2A1, GTPBP10, GUCY1B2, HCG18, HELZ2, HS3ST1, IFNG-AS1, IGHD3OR15-3B, IGHV3OR16-13, IGLV1-51, IL12A-AS1, INHBA-AS1, KCND2, KCNRG, LAMA2, LIFR-AS1, LINC00535, LINC00535, LRRIQ1, MED15P9, MEF2C-AS1, MEF2C-AS1, MEGF10, MGAM, MIR4472-1, MKLN1-AS1, NABP1, NCLN, NDC80, NFKBID, NUFIP1, OR2H1, PARP4, PCDHGA6, PKD1L3, PPP1R15A, PRKCA, PROX1-AS1, RABGEF1, RAPGEF4-AS1, REXO1L1, RHOU, RN7SL139P, RN7SL300P, RNA5SP508, RNF219-AS1, RNF219-AS1, RNU6-1163P, RNU6-204P, RNU6-439P, RNU7-24P, RP1-13P20.6, RP1-167A14.2, RP11-115D19.1, RP11-130F10.1, RP11-13J10.1, RP11-152L20.3, RP11-167H9.4, RP11-193H5.1, RP11-193H5.1, RP11-193H5.1, RP11-193H5.1, RP11-193H5.1, RP11-193H5.1, RP11-193H5.1, RP11-193H5.1, RP11-193H5.1, RP11-193H5.1, RP11-193H5.1, RP11-193H5.1, RP11-193H5.1, RP11-193H5.1, RP11-193H5.1, RP11-193H5.1, RP11-193H5.1, RP11-193H5.1, RP11-193H5.1, RP11-193H5.1, RP11-193H5.1, RP11-193H5.1, RP11-193H5.1, RP11-193H5.1, RP11-193H5.1, RP11-193H5.1, RP11-193H5.1, RP11-193H5.1, RP11-193H5.1, RP11-193H5.1, RP11-193H5.1, RP11-193H5.1, RP11-193H5.1, RP11-290F24.3, RP11-32K4.1, RP11-342D14.1, RP11-356I2.4, RP11-380P13.1, RP11-418J17.1, RP11-418J17.1, RP11-418J17.1, RP11-418J17.1, RP11-420N3.2, RP11-438D8.2, RP11-521M14.1, RP11-525K10.3, RP11-556E13.1, RP11-57H12.5, RP11-624C23.1, RP11-624C23.1, RP11-649A16.1, RP11-665G4.1, RP11-680B3.2, RP11-693J15.4, RP11-800A3.4, RP11-846C15.2, RP11-84A1.3, RP11-933H2.4, RP3-428L16.1, RP4-605O3.4, RP4-798P15.3, RP5-921G16.1, SCARNA17, SFXN1, SIGLECL1, SLC25A44, SNHG11, SNHG14, SNX7, SPATA3, STXBP5-AS1, SUSD2, SVILP1, THEMIS2, TMEM161B-AS1, TREML3P, UCK1, UGDH-AS1, UHRF1, UNC5B, VPS37A, WDR11-AS1, Y_RNA, Y_RNA, Y_RNA, Z93241.1, ZC3H11A, ZNF75D | g.chr1:144921929C>T, g.chr1:226250448C>A, g.chr12:54369904G>C, g.chr2:187503163T>A, g.chr1:144921929C>T, g.chr1:226250448C>A, g.chr12:54369904G>C, g.chr2:187503163T>A, g.chr5:178211315T>A, g.chr8:91563814A>T, g.chr6:67070080delC, g.chr4:105471796G>A, g.chr2:118550611G>A, g.chr2:154348726C>T, g.chr2:88760745C>G, g.chr2:165877995T>G, g.chr2:122460846G>A, g.chr2:175586396A>G, g.chr3:197340178G>T, g.chr2:213471410A>T, g.chr2:213728493A>G, g.chr2:189517419G>C, g.chr7:9632112A>G, g.chr7:135483925C>T, g.chr3:43251638_43251639insA, g.chr4:182964250C>T, g.chr11:3210791T>A, g.chr3:84337505T>A, g.chr11:2375643T>G, g.chr2:242959313C>T, g.chr3:18777713T>A, g.chr2:70995054C>T, g.chr16:3266876C>T, g.chr1:4847195C>T, g.chr20:55628288G>A, g.chr2:31749870T>A, g.chr13:45871419delA, g.chr6:67860870C>T, g.chr9:95582731C>T, g.chr11:59445195G>A, g.chr1:183595455A>G, g.chr13:31906193G>T, g.chr11:27626415G>A, g.chr16:33495285_33495286insA, g.chr10:93770839C>T, g.chr18:21086980_21086982delAAT, g.chr1:207972259A>T, g.chr1:207973646T>C, g.chr19:7680136G>A, g.chr10:119818582T>G, g.chr10:119965253_119965254insA, g.chr3:156877601T>G, g.chr17:40832095G>A, g.chr22:46640385T>C, g.chr2:55459143delT, g.chr8:87679163C>T, g.chr2:97492594C>G, g.chr1:160295426_160295427insT, g.chr20:24940434C>T, g.chr4:1254429C>T, g.chr19:20400975G>C, g.chr5:33427678G>A, g.chr14:43099982T>A, g.chr14:27894378A>T, g.chr1:70904807C>T, g.chr7:25160539T>C, g.chr5:150133109G>C, g.chr11:688434C>A, g.chr7:137069007G>T, g.chr11:90537477delT, g.chr11:90550534A>G, g.chr2:230231783C>T, g.chr20:17588352C>A, g.chr11:58975012C>T, g.chr4:110890159_110890162delAGAG, g.chr4:101578481T>C, g.chr3:69062790G>C, g.chr20:54943341C>T, g.chr9:67785215G>A, g.chr21:42716407A>C, g.chr3:125638563C>A, g.chr8:28420701T>A, g.chr4:22751513C>A, g.chr6:53010452G>C, g.chr9:124262939C>T, g.chr19:48259809C>T, g.chr10:25454526A>G, g.chrX:142495436C>T, g.chr14:81687250C>A, g.chr7:89983834C>A, g.chr13:51568594A>G, g.chr6:30279237C>G, g.chr20:62193649A>C, g.chr4:11397435A>G, g.chr12:68442955C>G, g.chr15:21215144A>T, g.chr16:33629661C>A, g.chr22:22675908G>A, g.chr3:159843829G>T, g.chr7:41809177G>T, g.chr7:120389048T>A, g.chr13:50589653T>C, g.chr6:129704367G>A, g.chr5:38660428A>C, g.chr8:94393441delG, g.chr8:94461891G>A, g.chr12:85450871G>T, g.chr2:130891661G>T, g.chr5:88429733C>T, g.chr5:88583445C>A, g.chr5:126794210G>A, g.chr7:141705372T>G, g.chr8:143260658G>A, g.chr7:130674877C>A, g.chr2:192551266T>G, g.chr19:3208147C>T, g.chr18:2571556_2571586delAAATTCGAACGGCTTTGGCGGGCCGAGGAAG, g.chr19:36388619G>T, g.chr13:45563559T>A, g.chr6:29429741_29429743delCTT, g.chr13:25068806C>A, g.chr5:140754893G>A, g.chr16:71989577G>T, g.chr19:49376797T>A, g.chr17:64801613delA, g.chr1:214130277C>A, g.chr7:66276345C>A, g.chr2:173561115G>T, g.chr8:86572555C>A, g.chr1:228871080C>T, g.chrX:48861976G>A, g.chr3:152920191_152920192GC>CT, g.chrX:75874205C>T, g.chr13:78998139C>A, g.chr13:79057983C>T, g.chr6:113291686T>A, g.chr4:3047917C>G, g.chr2:49462074T>C, g.chr16:24409378A>G, g.chr1:152918409C>A, g.chr6:167381607_167381611delATGAG, g.chr4:90544879C>T, g.chr4:178518991T>A, g.chr2:105541208T>A, g.chr15:92868229C>T, g.chr3:149846731C>G, g.chr1:238070699G>A, g.chr1:238070731G>A, g.chr1:238070749G>A, g.chr1:238070895G>A, g.chr1:238071034G>A, g.chr1:238071117G>A, g.chr1:238071135G>A, g.chr1:238071190G>A, g.chr1:238071266G>A, g.chr1:238071287G>A, g.chr1:238071306G>A, g.chr1:238071342G>A, g.chr1:238071347G>A, g.chr1:238071370G>A, g.chr1:238071451G>A, g.chr1:238071478G>A, g.chr1:238071484G>A, g.chr1:238071598_238071599GG>AA, g.chr1:238071608G>A, g.chr1:238071658G>A, g.chr1:238071677G>A, g.chr1:238071714G>A, g.chr1:238071723G>A, g.chr1:238071775G>A, g.chr1:238071797G>A, g.chr1:238071992G>A, g.chr1:238072113G>A, g.chr1:238072194G>A, g.chr1:238072237G>A, g.chr1:238072255G>A, g.chr1:238072300G>A, g.chr1:238072311G>A, g.chr1:238072345G>A, g.chr11:6196597_6196598insTATA, g.chr8:64733877T>A, g.chrX:45073270G>C, g.chr6:138158635_138158636insA, g.chr4:23692585T>C, g.chr1:119768875G>A, g.chr1:119768887G>A, g.chr1:119768926G>A, g.chr1:119769055G>A, g.chr16:5837011C>T, g.chr3:141067920A>G, g.chr8:27053531A>G, g.chr16:80394906_80394907insT, g.chr10:54510012G>A, g.chr1:95730653A>G, g.chr8:24391138C>T, g.chr8:24451317A>G, g.chr3:147069411G>T, g.chr4:15195943C>T, g.chr3:148630468A>T, g.chr12:92851841A>G, g.chr11:72950829G>A, g.chr18:42728207_42728208insA, g.chr4:62962231G>T, g.chr3:131083395T>C, g.chr6:161373103C>A, g.chr12:50517602C>A, g.chr1:177972975G>A, g.chr7:123688343C>T, g.chr1:26330648T>C, g.chr5:174956329G>C, g.chr19:51754498C>A, g.chr1:156180190C>T, g.chr20:37079029G>T, g.chr15:25339034G>T, g.chr1:99127301G>A, g.chr2:231865096_231865112delCCGGCCCGCATTCCTGC, g.chr6:147489421G>A, g.chr22:24585001G>A, g.chr10:30983825C>T, g.chr1:28206558C>T, g.chr5:87670463A>T, g.chr6:41182486G>C, g.chr9:134399303C>G, g.chr4:39543652_39543653insTATAT, g.chr19:4946212C>A, g.chr10:73061701C>T, g.chr8:17104648C>G, g.chr10:122573373A>T, g.chr11:118706497A>T, g.chr4:84558796C>T, g.chr6:18274906_18274907insT, g.chr22:42945257C>A, g.chr1:203768523A>G, g.chrX:134382987G>A | Panc |
| DO49445 | KRAS | g.chr12:25398284C>A | Panc |
| DO49448 | KRAS, TP53 | g.chr12:25398285C>G, g.chr17:7578455_7578460delCGCGGA | Panc |
| DO49451 | KRAS, TP53 | g.chr12:25398284C>A, g.chr17:7574018G>A | Panc |
| DO49454 | GATA3, KRAS, ARHGAP35, EIF4A2, KAT6A, GATA3, KRAS, ARHGAP35, EIF4A2, KAT6A, TP53, A2ML1-AS1, AC000370.2, AC004460.1, AC004538.3, AC004673.1, AC005592.2, AC007128.1, AC007970.1, AC009950.2, AC016723.4, AC092965.1, AC105245.1, AC108142.1, AC126177.1, AC144521.1, AC144521.1, ACO1, ADAM5, AE000661.37, AF121898.3, AF121898.3, AF121898.3, AL354806.1, AL512640.1, AP001596.6, ARSK, ATG16L1, BAI3, BEND4, CASKIN1, CCT6P3, CDC20B, COL6A1, COL6A5, CRYGEP, CSDE1, CSMD1, CTB-118P15.2, CTBS, CTC-340A15.2, CTC-340A15.2, CTC-340A15.2, CTC-340A15.2, CTC-457E21.6, CTD-2288O8.1, CTD-2307P3.1, CTD-3006G17.2, DCST2, DGKE, DISC1FP1, DISC1FP1, DNAJB9, DUT, EMCN-IT3, EMR4P, FAM179A, FAM84B, FLG-AS1, FNIP2, FOLH1B, FREM2, GABRB1, GBA3, GDF7, GFRAL, GRM6, GRPEL1, HLA-AS1, HPN-AS1, HTR5BP, IGHV2-5, IL1RAP, IL21R-AS1, ILDR2, JAG2, KCNC1, LINC00189, LINC00475, LINC00634, LLNLF-65H9.1, LRRC37A16P, MEF2C-AS1, MIR1268A, MIR4431, MIR4454, MTMR9LP, MTRNR2L7, Metazoa_SRP, NALCN-AS1, NFATC3, NPAS2, NPSR1-AS1, NTF4, NUP188, OR52M1, OTX2-AS1, PAIP2B, PCDHA5, PKD1L1, PLEKHA8P1, PNMA5, RASGRP1, RN7SKP149, RN7SL278P, RN7SL33P, RN7SL644P, RN7SL865P, RNA5SP24, RNA5SP474, RNF215, RNF219-AS1, RNF219-AS1, RNF219-AS1, RNU2-54P, RNU6-133P, RNU6-227P, RNU6-533P, RNU7-174P, RP1-23K20.2, RP1-261D10.2, RP1-272J12.1, RP11-103J8.1, RP11-114H24.5, RP11-13J10.1, RP11-152P17.2, RP11-15B17.1, RP11-1L12.3, RP11-230B22.1, RP11-231I13.2, RP11-274B21.1, RP11-274B21.1, RP11-284M14.1, RP11-286H14.4, RP11-313J2.1, RP11-333E1.1, RP11-33N16.3, RP11-368J21.3, RP11-382A20.4, RP11-382A20.4, RP11-402C9.1, RP11-420N3.2, RP11-420N3.2, RP11-428C19.4, RP11-562L8.1, RP11-572M11.4, RP11-586K2.1, RP11-586K2.1, RP11-586K2.1, RP11-624C23.1, RP11-634B7.4, RP11-679C8.2, RP11-679C8.2, RP11-679C8.2, RP11-680F20.9, RP11-707M1.1, RP11-708B6.2, RP11-742B18.1, RP11-770E5.1, RP11-77K12.5, RP11-804A23.1, RP11-867G2.8, RP11-87M18.2, RP11-89K10.1, RP4-724E13.2, RP4-755D9.1, RP5-1091N2.9, SCN10A, SCRG1, SEC63, SEMA5A, SHANK3, SIGLEC9, SLAIN2, SLC46A1, SMARCA2, SNHG14, SNHG14, SPHK1, SRGAP2-AS1, SSPO, STEAP2-AS1, SUGT1P3, SWT1, TECTA, TFAP2C, TLE3, TLR8-AS1, TMEM230, TOP3B, TRBV28, TRIM3, TRPM6, TSPAN32, TYW1B, U3, USP2-AS1, WDR83OS, WSCD1, XKR8, Y_RNA, ZFHX4, ZFHX4-AS1, hsa-mir-490, hsa-mir-490, hsa-mir-490, snoU13 | g.chr10:8100695G>A, g.chr12:25398285C>G, g.chr19:47422279A>G, g.chr3:186504990G>A, g.chr8:41906720G>T, g.chr10:8100695G>A, g.chr12:25398285C>G, g.chr19:47422279A>G, g.chr3:186504990G>A, g.chr8:41906720G>T, g.chr17:7578474_7578475insG, g.chr12:8965792G>A, g.chr7:126032708delT, g.chr15:31094559G>T, g.chr7:11315632C>A, g.chrX:23137250C>T, g.chr5:141727428G>A, g.chr7:8380794G>A, g.chr2:211279376T>C, g.chr2:231020857G>A, g.chr2:168745143G>A, g.chr3:166743575G>A, g.chr18:26774281G>A, g.chr4:183047254G>C, g.chr12:108190719C>T, g.chr3:18552875C>A, g.chr3:18885890A>G, g.chr9:32433797G>A, g.chr8:39186251_39186262delCAGCTGCAGTGA, g.chr14:22877706G>A, g.chr8:88683624C>A, g.chr8:88726699G>A, g.chr8:88762847C>T, g.chr13:81780623G>C, g.chr10:44527168_44527171delTAAC, g.chr21:27759125G>A, g.chr5:94939567C>T, g.chr2:234202930G>A, g.chr6:69943208C>A, g.chr4:42114408C>T, g.chr16:2230984A>G, g.chr7:64522155C>G, g.chr5:54409721C>A, g.chr21:47412310C>T, g.chr3:130137764C>T, g.chr2:208978191T>C, g.chr1:115279426C>T, g.chr8:3000092C>T, g.chr8:89024435C>T, g.chr1:85018566T>G, g.chr5:163869581_163869582insA, g.chr5:164272364delG, g.chr5:164527486G>A, g.chr5:164569711A>G, g.chr19:22783844T>G, g.chr5:52080396C>A, g.chr14:42848883A>T, g.chr14:28040574G>A, g.chr1:155006160C>A, g.chr17:54945495T>G, g.chr11:90353686C>T, g.chr11:90523402_90523412delTTTCACTGTGT, g.chr7:108214552_108214555delATGG, g.chr15:48635569A>G, g.chr4:101581551C>T, g.chr19:6973209A>G, g.chr2:29280913A>T, g.chr8:127569465C>T, g.chr1:152248244A>G, g.chr4:159789407A>G, g.chr11:89380318G>A, g.chr13:39458431A>G, g.chr4:47427808G>A, g.chr4:22741615C>G, g.chr2:20870736G>A, g.chr6:55266935C>T, g.chr5:178408233C>A, g.chr4:7062207G>A, g.chr1:221045325G>A, g.chr19:35577341C>T, g.chr2:118628173_118628174insT, g.chr14:106497539T>A, g.chr3:190366996A>G, g.chr16:27467023A>G, g.chr1:166887433G>A, g.chr14:105613687A>T, g.chr11:17803421C>T, g.chr21:30574780T>A, g.chr9:94925106G>A, g.chr22:42350158C>T, g.chr19:28467264C>T, g.chr17:66143684A>G, g.chr5:88584183A>T, g.chr15:22512321G>T, g.chr2:52931307G>A, g.chr5:170178141T>C, g.chr1:32708921G>A, g.chr10:37891478G>T, g.chrX:133350511C>T, g.chr13:101374028G>A, g.chr16:68260342T>C, g.chr2:101612576_101612577insT, g.chr7:34520424C>T, g.chr19:49564657C>T, g.chr9:131765150C>T, g.chr11:4566959C>T, g.chr14:57527053G>T, g.chr2:71415064A>C, g.chr5:140203035G>A, g.chr7:47814701delA, g.chr12:45590944C>T, g.chrX:152159087G>A, g.chr15:38791141C>T, g.chrX:142282556G>T, g.chr15:77271778C>T, g.chr17:2461107G>A, g.chr10:94420884A>G, g.chr12:116153722T>A, g.chr13:19662128A>C, g.chr20:5076340C>G, g.chr22:30776322G>C, g.chr13:78652529G>C, g.chr13:78860028G>C, g.chr13:78961485A>G, g.chr8:77180740C>T, g.chrX:21230759C>T, g.chr17:74651293C>T, g.chr8:36165827G>A, g.chr8:58468946T>A, g.chrX:130165116C>T, g.chr14:71769634G>T, g.chr22:35366940C>T, g.chr20:10785921T>A, g.chr15:78280234delG, g.chr2:105499880G>A, g.chr8:107050156_107050157insT, g.chr4:100971517_100971518insT, g.chr11:27163098A>G, g.chr1:63172471G>A, g.chr3:70284800C>T, g.chr7:128191538_128191542delTAATG, g.chr7:128271319_128271321delCAA, g.chr4:144088927C>T, g.chr7:128770473G>A, g.chr10:42839883_42839884insT, g.chr17:5107771_5107772insA, g.chr14:90159101_90159102insA, g.chr16:22559660C>T, g.chr15:83980583G>A, g.chr15:84024037G>A, g.chr4:183083633G>A, g.chr16:5406078C>T, g.chr16:5899149G>A, g.chr11:19236984A>T, g.chr14:29775865C>T, g.chr3:112791074T>A, g.chr8:89382688T>C, g.chr8:89489570_89489571delAC, g.chr8:89539770C>T, g.chr8:24622417G>A, g.chr1:247822116T>A, g.chr4:121002196A>C, g.chr4:121178192A>G, g.chr4:121302503A>G, g.chr11:125798378C>T, g.chr11:49666826A>T, g.chr11:109343419C>T, g.chr4:88657647A>G, g.chr8:49594102G>A, g.chr16:75549576A>G, g.chr11:60590323delA, g.chr11:94434889C>T, g.chrX:36407802C>T, g.chr8:127726974G>A, g.chr7:50978753C>T, g.chrX:119270004_119270005delCA, g.chrX:70416006C>A, g.chr3:38740002G>A, g.chr4:174320624T>A, g.chr6:108246070G>T, g.chr5:9066581C>T, g.chr22:51117328C>T, g.chr19:51639499T>C, g.chr4:48371962_48371970delTGTTGAGTG, g.chr17:26723037C>T, g.chr9:2033060C>T, g.chr15:25450527G>T, g.chr15:25536776G>T, g.chr17:74381586_74381587GG>CT, g.chr1:206550372G>A, g.chr7:149512731G>A, g.chr7:89538001A>G, g.chr13:41496624G>A, g.chr1:185143722T>C, g.chr11:121028629G>A, g.chr20:55214184G>A, g.chr15:70345597C>T, g.chrX:12919339C>T, g.chr20:5081172C>A, g.chr22:22324598C>T, g.chr7:142427617C>G, g.chr11:6495573G>T, g.chr9:77359063T>A, g.chr11:2339130C>T, g.chr7:72036904delT, g.chr6:71839320G>A, g.chr11:119308341A>G, g.chr19:12779153G>A, g.chr17:5675757G>A, g.chr1:28286799C>T, g.chr2:129105810T>C, g.chr8:77767860G>A, g.chr8:77541409G>T, g.chr7:136404523C>A, g.chr7:136526023A>T, g.chr7:136545865T>C, g.chrX:135214028_135214029insT | Panc |
| DO49457 | KRAS, CDKN2A | g.chr12:25398284C>T, g.chr9:21971111G>A | Panc |
| DO49460 | KRAS | g.chr12:25398285C>G | Panc |
| DO49463 | KRAS, TP53 | g.chr12:25380275T>G, g.chr17:7577548C>T | Panc |
| DO49466 | KRAS, CTNNB1 | g.chr12:25398285C>G, g.chr3:41266137C>T | Panc |
| DO49478 | TP53 | g.chr17:7577120C>T | Panc |
| DO49481 | KRAS | g.chr12:25398284C>A | Panc |
| DO49484 | KRAS, PREX2 | g.chr12:25398285C>G, g.chr8:69145730G>A | Panc |
| DO49533 | NRAS | g.chr1:115256530G>T | Myeloid |
| DO49537 | ERBB4 | g.chr2:212570063C>T | Myeloid |
| DO50306 | TP53, NRG1 | g.chr17:7577094G>A, g.chr8:32463091T>G | Eso |
| DO50307 | KRAS | g.chr12:25398284C>T | Eso |
| DO50309 | TP53, SF3B1 | g.chr17:7577094G>A, g.chr2:198266834T>C | Eso |
| DO50311 | TP53 | g.chr17:7578413C>T | Eso |
| DO50314 | TP53, SMAD4, RUNX1T1 | g.chr17:7578530A>G, g.chr18:48591919G>A, g.chr8:92970335G>A | Eso |
| DO50315 | BMP5 | g.chr6:55625290C>T | Eso |
| DO50316 | TP53, GNAS, CTNNB1, C16orf62 | g.chr17:7577559G>C, g.chr20:57484421G>A, g.chr3:41266113C>A, g.chr16:19639113_19639134delTCTGGTTTCCCCAAGGTAGGCT | Eso |
| DO50318 | TP53, ERBB2 | g.chr17:7577022G>A, g.chr17:37881332G>A | Eso |
| DO50319 | TP53 | g.chr17:7578406C>T | Eso |
| DO50320 | TP53 | g.chr17:7577574T>C | Eso |
| DO50323 | TP53, PIK3CA | g.chr17:7576855G>A, g.chr3:178936091G>A | Eso |
| DO50325 | TP53, GNAS | g.chr17:7577548C>T, g.chr20:57484420C>T | Eso |
| DO50326 | ARID1A, TP53, AR | g.chr1:27101138C>T, g.chr17:7578479G>A, g.chrX:66948781T>G | Eso |
| DO50327 | TP53 | g.chr17:7577548C>T | Eso |
| DO50328 | TP53, NRG1 | g.chr17:7578205C>A, g.chr8:32599593A>G | Eso |
| DO50329 | TP53, ROBO2 | g.chr17:7577094G>A, g.chr3:77147279C>T | Eso |
| DO50330 | CDKN2A | g.chr9:21971186G>A | Eso |
| DO50331 | TP53, ERBB2, SETBP1 | g.chr17:7578406C>T, g.chr17:37881332G>A, g.chr18:42647922A>T | Eso |
| DO50332 | TP53, ERBB2, APC | g.chr17:7577556C>A, g.chr17:37881332G>A, g.chr5:112175951_112175952insA | Eso |
| DO50334 | TP53 | g.chr17:7577099C>T | Eso |
| DO50336 | TP53 | g.chr17:7574003G>A | Eso |
| DO50337 | TP53, PTEN | g.chr17:7578555C>T, g.chr10:89726584_89726585insA | Eso |
| DO50338 | TP53, SMAD4, SMARCA4, SMARCA4, PTPRT | g.chr17:7577559G>A, g.chr18:48591918C>T, g.chr19:11132513C>T, g.chr19:11144113G>A, g.chr20:40704941T>A | Eso |
| DO50340 | AR, MAF | g.chrX:66764848A>G, g.chr16:79630783_79630784insT | Eso |
| DO50341 | TP53 | g.chr17:7577539G>A | Eso |
| DO50342 | ZNF521, PHOX2B, KIT, AR, APC | g.chr18:22806441A>C, g.chr4:41747895C>T, g.chr4:55605608T>G, g.chrX:66764848A>G, g.chr5:112175951_112175952insA | Eso |
| DO50343 | TP53, CTNNB1 | g.chr17:7577560A>G, g.chr3:41266104G>A | Eso |
| DO50345 | TP53, TP53 | g.chr17:7577120C>T, g.chr17:7578526C>A | Eso |
| DO50346 | CDH11, TP53, BCL11A | g.chr16:64980577A>G, g.chr17:7577538C>T, g.chr2:60687244G>A | Eso |
| DO50348 | ERBB3, TP53, RUNX1T1 | g.chr12:56492633A>G, g.chr17:7578534C>G, g.chr8:92970335G>A | Eso |
| DO50350 | TP53, TP53, NRG1 | g.chr17:7578394T>A, g.chr17:7578534C>G, g.chr8:32599593A>G | Eso |
| DO50355 | TP53, SMAD4 | g.chr17:7577548C>T, g.chr18:48591918C>T | Eso |
| DO50357 | TP53 | g.chr17:7578212G>A | Eso |
| DO50362 | CDH11, TP53 | g.chr16:65005898T>G, g.chr17:7574017C>A | Eso |
| DO50364 | TP53 | g.chr17:7577022G>A | Eso |
| DO50365 | PIK3CA | g.chr3:178936091G>A | Eso |
| DO50367 | CD209 | g.chr19:7806922C>T | Eso |
| DO50370 | TP53 | g.chr17:7577086_7577087delTG | Eso |
| DO50372 | TP53 | g.chr17:7578526C>T | Eso |
| DO50381 | ARID1A, TP53 | g.chr1:27101099C>T, g.chr17:7578455_7578460delCGCGGA | Eso |
| DO50382 | SMARCA4, AFF3, CTNNB1, ARID1A | g.chr19:11132542G>A, g.chr2:100167832G>A, g.chr3:41266113C>T, g.chr1:27023151_27023179delGCGGAGCCGGCAGCGGCGGCGGGCCCGGC | Eso |
| DO50383 | TP53 | g.chr17:7577547C>T | Eso |
| DO50384 | SMAD4, MUC16 | g.chr18:48591888G>C, g.chr19:9083708T>G | Eso |
| DO50385 | TP53 | g.chr17:7577538C>T | Eso |
| DO50387 | TP53, PTPRT, GNAS | g.chr17:7577114C>T, g.chr20:40704941T>A, g.chr20:57430058G>A | Eso |
| DO50388 | TP53 | g.chr17:7577538C>T | Eso |
| DO50389 | TP53 | g.chr17:7578370C>T | Eso |
| DO50390 | TP53, TP53 | g.chr17:7577547C>A, g.chr17:7578403C>A | Eso |
| DO50392 | PIK3CA | g.chr3:178936091G>A | Eso |
| DO50393 | TP53, APC | g.chr17:7578206T>C, g.chr5:112175951_112175952insA | Eso |
| DO50396 | KRAS | g.chr12:25398284C>T | Eso |
| DO50398 | TP53, CDKN2A | g.chr17:7577556C>A, g.chr9:21971111G>A | Eso |
| DO50399 | CTNNA2 | g.chr2:80874952G>A | Eso |
| DO50401 | TP53 | g.chr17:7574003G>A | Eso |
| DO50402 | NRG1 | g.chr8:32617831T>C | Eso |
| DO50406 | TP53, ATM, CSMD3 | g.chr17:7577120C>T, g.chr11:108160451delA, g.chr8:113236642delT | Eso |
| DO50407 | TP53 | g.chr17:7578213_7578214delAA | Eso |
| DO50408 | TP53, CDKN2A | g.chr17:7577538C>T, g.chr9:21971186G>A | Eso |
| DO50409 | TP53 | g.chr17:7574003G>A | Eso |
| DO50410 | USP6, GNAS | g.chr17:5033275C>T, g.chr20:57430265C>T | Eso |
| DO50411 | TP53, SMAD4, APC | g.chr17:7577574T>C, g.chr18:48591918C>T, g.chr5:112175951_112175952insA | Eso |
| DO50412 | PTEN, TP53 | g.chr10:89717708C>T, g.chr17:7577121G>A | Eso |
| DO50419 | APC | g.chr5:112102920A>G | Prost |
| DO50430 | TP53, FAT4 | g.chr17:7577547C>A, g.chr4:126369688C>T | Prost |
| DO50431 | SPOP | g.chr17:47696426A>T | Prost |
| DO50434 | TP53 | g.chr17:7578190T>C | Eso |
| DO50436 | WIF1 | g.chr12:65444775G>A | Eso |
| DO50437 | TP53 | g.chr17:7578212G>A | Eso |
| DO50438 | TP53 | g.chr17:7578212G>A | Eso |
| DO50439 | TP53 | g.chr17:7577551C>A | Eso |
| DO50440 | ARID1A, TP53, TP53 | g.chr1:27105930_27105931insG, g.chr17:7578179_7578186delCAGGCGGC, g.chr17:7578179_7578186delCAGGCGGC | Eso |
| DO50441 | SETBP1 | g.chr18:42647922A>T | Eso |
| DO50442 | TP53 | g.chr17:7577121G>A | Eso |
| DO50443 | TP53 | g.chr17:7578268A>C | Eso |
| DO50444 | TP53, CTNNA2 | g.chr17:7577120C>A, g.chr2:79971659A>G | Eso |
| DO50445 | TP53, APC | g.chr17:7578406C>T, g.chr5:112175639C>T | Eso |
| DO50446 | TP53, ANK1, APC | g.chr17:7578413C>T, g.chr8:41512976G>A, g.chr5:112175951_112175952insA | Eso |
| DO50447 | CDH11, MECOM, BMP5, ANK1 | g.chr16:65005898T>G, g.chr3:168812978G>A, g.chr6:55620178A>C, g.chr8:41529889C>T | Eso |
| DO50448 | TP53 | g.chr17:7577120C>T | Eso |
| DO50449 | TP53, PIK3CA, PREX2, CDKN2A | g.chr17:7577548C>T, g.chr3:178936091G>A, g.chr8:69031679C>T, g.chr9:21971186G>A | Eso |
| DO50450 | MUC16, ERBB4 | g.chr19:9083708T>G, g.chr2:212570063C>T | Eso |
| DO50451 | KRAS, CDKN2A | g.chr12:25398285C>A, g.chr9:21974715_21974732delGCAGCGCCCCCGCCTCCA | Eso |
| DO50452 | FOXO1, CDH11, BMP5 | g.chr13:41130185T>A, g.chr16:65005898T>G, g.chr6:55620178A>C | Eso |
| DO50453 | KRAS, TP53, PIK3CA, LYL1 | g.chr12:25398281C>T, g.chr17:7578403C>A, g.chr3:178936082G>A, g.chr19:13209934delT | Eso |
| DO50454 | TP53, LRP1B | g.chr17:7577022G>A, g.chr2:141643809T>C | Eso |
| DO50778 | TP53 | g.chr17:7578212G>A | Liver |
| DO50780 | CTNNB1 | g.chr3:41266136T>C | Liver |
| DO50785 | CDKN2A | g.chr9:21971111G>A | Liver |
| DO50787 | CTNNB1 | g.chr3:41266097G>A | Liver |
| DO50791 | TP53 | g.chr17:7578271T>C | Liver |
| DO50793 | CTNNB1, PIK3CA, DCAF12L2 | g.chr3:41266137C>T, g.chr3:178952085A>G, g.chrX:125298907G>A | Liver |
| DO50799 | TP53 | g.chr17:7577580T>C | Liver |
| DO50802 | CTNNB1 | g.chr3:41266113C>T | Liver |
| DO50804 | CTNNB1 | g.chr3:41266124A>G | Liver |
| DO50806 | TP53 | g.chr17:7578235T>C | Liver |
| DO50809 | ATRX | g.chrX:76938401_76938402delTT | Liver |
| DO50815 | TP53 | g.chr17:7577547C>T | Liver |
| DO50817 | CTNNB1 | g.chr3:41266107T>G | Liver |
| DO50818 | SF3B1, CTNNB1, MEF2C-AS1 | g.chr2:198266611C>T, g.chr3:41266136T>C, g.chr5:88663783_88663784delTG | Liver |
| DO50819 | LRP1B | g.chr2:141816617G>A | Liver |
| DO50820 | TP53 | g.chr17:7577559G>C | Liver |
| DO50822 | CTNNB1 | g.chr3:41266110A>C | Liver |
| DO50829 | TP53 | g.chr17:7577579G>C | Liver |
| DO50832 | TP53, U2AF1 | g.chr17:7577121G>A, g.chr21:44524456G>A | Liver |
| DO50834 | CTNNB1 | g.chr3:41266125C>T | Liver |
| DO50842 | CTNNB1, HLA-A | g.chr3:41266101C>G, g.chr6:29910685G>C | Liver |
| DO50844 | TP53, CDKN2A, HMCN1, NANOS2 | g.chr17:7577539G>A, g.chr9:21971177C>A, g.chr1:185988781G>T, g.chr19:46417907C>A | Liver |
| DO50850 | TP53 | g.chr17:7578370C>T | Liver |
| DO50851 | CTNNB1, APOB | g.chr3:41266124A>G, g.chr2:21229611_21229616delGTGCAT | Liver |
| DO50857 | CTNNB1 | g.chr3:41266101C>A | Liver |
| DO51046 | TP53 | g.chr17:7578527A>G | Prost |
| DO51049 | FAT3 | g.chr11:92086572G>A | Prost |
| DO51050 | KDM5A | g.chr12:393307A>T | Prost |
| DO51056 | PRRX1 | g.chr1:170695508G>A | Prost |
| DO51057 | CTNNB1 | g.chr3:41266124A>G | Prost |
| DO51061 | MED12 | g.chrX:70349258C>T | Prost |
| DO51062 | KMT2A | g.chr11:118348808G>A | Prost |
| DO51063 | IDH1 | g.chr2:209113112C>T | Prost |
| DO51064 | MED12 | g.chrX:70349258C>T | Prost |
| DO51070 | TP53, FAT4 | g.chr17:7577102C>T, g.chr4:126369688C>T | Prost |
| DO51074 | TP53 | g.chr17:7578525G>C | Prost |
| DO51078 | NUP214 | g.chr9:134000975delG | Prost |
| DO51084 | MED12 | g.chrX:70349258C>T | Prost |
| DO51086 | SPOP | g.chr17:47696426A>T | Prost |
| DO51087 | TP53 | g.chr17:7577568C>A | Prost |
| DO51088 | SPOP | g.chr17:47696425A>G | Prost |
| DO51090 | SMARCA4 | g.chr19:11172713_11172714delAG | Prost |
| DO51103 | TP53 | g.chr17:7577539G>A | Prost |
| DO51111 | KMT2C | g.chr7:151882660C>A | Prost |
| DO51114 | KDM5A | g.chr12:393307A>T | Prost |
| DO51126 | RUNX1T1 | g.chr8:92967699_92967700insA | Prost |
| DO51128 | TP53 | g.chr17:7577120C>T | Prost |
| DO51133 | CTNNB1, PIK3CA | g.chr3:41266125C>T, g.chr3:178952085A>G | Prost |
| DO51137 | TP53 | g.chr17:7577094G>A | Prost |
| DO51141 | FOXL2 | g.chr3:138663667A>T | Prost |
| DO51148 | GRIN2A | g.chr16:9856044C>T | Prost |
| DO51150 | CCNB1IP1 | g.chr14:20781884T>C | Prost |
| DO51158 | SPOP | g.chr17:47696426A>C | Prost |
| DO51178 | MEN1 | g.chr11:64577330_64577333delAGAC | Panc |
| DO51187 | ATM | g.chr11:108117798C>T | Panc |
| DO51465 | FLI1, KRAS | g.chr11:128628193C>T, g.chr12:25398284C>T | Panc |
| DO51466 | KRAS, TP53 | g.chr12:25398284C>A, g.chr17:7578525G>C | Panc |
| DO51467 | KRAS, TP53 | g.chr12:25398284C>A, g.chr17:7577559G>A | Panc |
| DO51468 | KRAS | g.chr12:25398284C>A | Panc |
| DO51469 | KRAS | g.chr12:25398284C>T | Panc |
| DO51470 | KRAS, TP53 | g.chr12:25398284C>A, g.chr17:7577539G>A | Panc |
| DO51472 | KRAS | g.chr12:25398284C>A | Panc |
| DO51473 | KRAS, RBM10 | g.chr12:25398284C>A, g.chrX:47041266G>A | Panc |
| DO51474 | KRAS, SDHA, STAT5B, BRAF | g.chr12:25398285C>G, g.chr5:218471A>G, g.chr17:40352476_40352477insT, g.chr7:140434295delA | Panc |
| DO51475 | KRAS, ZFHX3, TP53 | g.chr12:25398284C>A, g.chr16:73093410A>T, g.chr17:7577539G>A | Panc |
| DO51476 | TP53 | g.chr17:7578406C>T | Panc |
| DO51477 | KRAS | g.chr12:25398284C>A | Panc |
| DO51478 | KRAS, TP53 | g.chr12:25398284C>T, g.chr17:7578555C>A | Panc |
| DO51479 | TP53 | g.chr17:7577121G>A | Panc |
| DO51480 | KRAS, TP53 | g.chr12:25398284C>A, g.chr17:7578406C>T | Panc |
| DO51481 | KRAS, TP53, U2AF1, CDKN2A | g.chr12:25398284C>A, g.chr17:7577058C>A, g.chr21:44524456G>A, g.chr9:21971149_21971172delGGCTCCGCGCCGTGGAGCAGCAGC | Panc |
| DO51483 | KRAS, TP53 | g.chr12:25398284C>T, g.chr17:7578190T>C | Panc |
| DO51484 | KRAS, TP53, SMARCA4, CDKN2A | g.chr12:25398284C>A, g.chr17:7577097C>T, g.chr19:11132542G>A, g.chr9:21971108C>T | Panc |
| DO51485 | KRAS, TP53, U2AF1 | g.chr12:25398284C>T, g.chr17:7577121G>A, g.chr21:44524456G>A | Panc |
| DO51486 | KRAS, PRPF40B | g.chr12:25398284C>A, g.chr12:50017647T>G | Panc |
| DO51488 | KRAS, TP53 | g.chr12:25398284C>A, g.chr17:7574003G>A | Panc |
| DO51489 | TP53 | g.chr17:7577538C>T | Panc |
| DO51490 | KRAS, TP53, CDKN2A | g.chr12:25398284C>T, g.chr17:7576897G>A, g.chr9:21971111G>A | Panc |
| DO51491 | CTNNA2, PREX2 | g.chr2:80874952G>A, g.chr8:68981315C>T | Panc |
| DO51492 | KRAS | g.chr12:25398284C>A | Panc |
| DO51493 | KRAS, TP53 | g.chr12:25398284C>A, g.chr17:7578555C>T | Panc |
| DO51494 | KRAS | g.chr12:25398284C>A | Panc |
| DO51495 | KRAS, TP53 | g.chr12:25398284C>A, g.chr17:7578406C>T | Panc |
| DO51496 | KRAS, PTPRT | g.chr12:25398285C>G, g.chr20:40708927C>T | Panc |
| DO51497 | PTEN, KRAS, TP53, BAX | g.chr10:89692905G>A, g.chr12:25398285C>G, g.chr17:7578406C>T, g.chr19:49458970_49458971insG | Panc |
| DO51498 | KRAS, TP53 | g.chr12:25398284C>T, g.chr17:7578394T>C | Panc |
| DO51499 | KRAS | g.chr12:25398285C>G | Panc |
| DO51500 | KRAS, MAX, GNAS | g.chr12:25398281C>T, g.chr14:65544747C>T, g.chr20:57484420C>T | Panc |
| DO51501 | KRAS, TP53 | g.chr12:25398284C>T, g.chr17:7578263G>A | Panc |
| DO51502 | CDKN2A | g.chr9:21971108C>T | Panc |
| DO51504 | KRAS, TP53 | g.chr12:25398285C>G, g.chr17:7577579G>C | Panc |
| DO51505 | KRAS, TP53 | g.chr12:25380276T>A, g.chr17:7577120C>T | Panc |
| DO51506 | KRAS, TP53 | g.chr12:25398285C>G, g.chr17:7577124C>T | Panc |
| DO51507 | KRAS | g.chr12:25380276T>C | Panc |
| DO51509 | KRAS, TP53 | g.chr12:25398284C>A, g.chr17:7577539G>A | Panc |
| DO51510 | AR | g.chrX:66943842delT | Panc |
| DO51511 | KRAS, TP53 | g.chr12:25398285C>G, g.chr17:7578190T>C | Panc |
| DO51512 | KRAS, TP53 | g.chr12:25398284C>T, g.chr17:7578530A>G | Panc |
| DO51513 | KRAS | g.chr12:25398284C>T | Panc |
| DO51514 | KRAS | g.chr12:25398284C>A | Panc |
| DO51515 | KRAS, TP53, GNAS | g.chr12:25398284C>A, g.chr17:7577548C>T, g.chr20:57484421G>A | Panc |
| DO51518 | KRAS, TP53 | g.chr12:25398284C>T, g.chr17:7577539G>A | Panc |
| DO51519 | KRAS | g.chr12:25398284C>T | Panc |
| DO51520 | KRAS, SF3B1 | g.chr12:25398284C>T, g.chr2:198266834T>C | Panc |
| DO51521 | PRPF40B | g.chr12:50017647T>G | Panc |
| DO51522 | KRAS | g.chr12:25398284C>T | Panc |
| DO51523 | KRAS, TP53 | g.chr12:25398284C>T, g.chr17:7577117A>G | Panc |
| DO51524 | KRAS, SMAD4 | g.chr12:25398284C>T, g.chr18:48591919G>A | Panc |
| DO51527 | KRAS | g.chr12:25398284C>T | Panc |
| DO51528 | KRAS, GNAS, GNAS | g.chr12:25398284C>A, g.chr20:57430990C>T, g.chr20:57484420C>T | Panc |
| DO51529 | KRAS, TP53, CDKN2A | g.chr12:25398285C>G, g.chr17:7577094G>A, g.chr9:21974777_21974780delGCCA | Panc |
| DO51530 | KRAS | g.chr12:25398285C>G | Panc |
| DO51531 | KRAS | g.chr12:25398284C>T | Panc |
| DO51532 | KRAS, TP53 | g.chr12:25398284C>A, g.chr17:7578406C>T | Panc |
| DO51533 | KRAS, TP53 | g.chr12:25398284C>A, g.chr17:7577022G>A | Panc |
| DO51534 | KRAS, SMAD4, SMAD4, CDKN2A | g.chr12:25398284C>A, g.chr18:48591918C>T, g.chr18:48603032C>T, g.chr9:21971120G>A | Panc |
| DO51535 | KRAS, TP53 | g.chr12:25398284C>A, g.chr17:7578449C>T | Panc |
| DO51536 | KRAS, TP53 | g.chr12:25398285C>G, g.chr17:7576855G>A | Panc |
| DO51537 | KRAS, TP53 | g.chr12:25398284C>T, g.chr17:7578479G>C | Panc |
| DO51538 | KRAS, PRPF40B, ZFHX3, TP53, ARID1A | g.chr12:25398284C>T, g.chr12:50017647T>G, g.chr16:73093410A>T, g.chr17:7578395G>A, g.chr1:27023151_27023179delGCGGAGCCGGCAGCGGCGGCGGGCCCGGC | Panc |
| DO51540 | KRAS, TP53 | g.chr12:25398284C>T, g.chr17:7577121G>A | Panc |
| DO51542 | KRAS, TP53 | g.chr12:25398284C>T, g.chr17:7578535T>C | Panc |
| DO51543 | KRAS, TP53, SMAD4, CD209 | g.chr12:25398285C>G, g.chr17:7577570C>T, g.chr18:48591919G>A, g.chr19:7806334C>T | Panc |
| DO51544 | KRAS, TP53 | g.chr12:25398284C>T, g.chr17:7578190T>C | Panc |
| DO51546 | KRAS | g.chr12:25398284C>A | Panc |
| DO51548 | PICALM, KRAS, SMAD4, KLK2 | g.chr11:85685824G>A, g.chr12:25398284C>A, g.chr18:48591918C>T, g.chr19:51382261G>C | Panc |
| DO51549 | KRAS, HMGN2P46, TGFBR2 | g.chr12:25398284C>A, g.chr15:45842835A>T, g.chr3:30732969C>T | Panc |
| DO51953 | BCL9, PTEN, SETBP1, CUL3, CRNKL1, MUC4, CTNND2, IL6ST, NPM1, FOXA1, FOXA1, CDK12, CDK12, BCL9, PTEN, SETBP1, CUL3, CRNKL1, MUC4, CTNND2, IL6ST, NPM1, FOXA1, FOXA1, CDK12, CDK12, GNA11, BCL9, PTEN, CUL3, CRNKL1, MUC4, IL6ST, KDM6A, FOXA1, FOXA1, CDK12, CDK12, GNA11, BCL9, PTEN, CUL3, CRNKL1, MUC4, IL6ST, KDM6A, FOXA1, FOXA1, CDK12, CDK12, GNA11 | g.chr1:147013183T>G, g.chr10:89692904C>T, g.chr18:42643830C>T, g.chr2:225336268T>G, g.chr20:20030069C>A, g.chr3:195493607G>A, g.chr5:11565151C>T, g.chr5:55236129G>C, g.chr5:170827858C>G, g.chr14:38059226_38059227delCA, g.chr14:38059230_38059231insG, g.chr17:37657662delT, g.chr17:37676234delT, g.chr1:147013183T>G, g.chr10:89692904C>T, g.chr18:42643830C>T, g.chr2:225336268T>G, g.chr20:20030069C>A, g.chr3:195493607G>A, g.chr5:11565151C>T, g.chr5:55236129G>C, g.chr5:170827858C>G, g.chr14:38059226_38059227delCA, g.chr14:38059230_38059231insG, g.chr17:37657662delT, g.chr17:37676234delT, g.chr19:3121461_3121462insC, g.chr1:147013183T>G, g.chr10:89692904C>T, g.chr2:225336268T>G, g.chr20:20030069C>A, g.chr3:195493607G>A, g.chr5:55236129G>C, g.chrX:44870225G>T, g.chr14:38059226_38059227delCA, g.chr14:38059230_38059231insG, g.chr17:37657662delT, g.chr17:37676234delT, g.chr19:3121461_3121462insC, g.chr1:147013183T>G, g.chr10:89692904C>T, g.chr2:225336268T>G, g.chr20:20030069C>A, g.chr3:195493607G>A, g.chr5:55236129G>C, g.chrX:44870225G>T, g.chr14:38059226_38059227delCA, g.chr14:38059230_38059231insG, g.chr17:37657662delT, g.chr17:37676234delT, g.chr19:3121461_3121462insC | Prost |
| DO51954 | PRCC, DDX6, RB1, TBL1XR1, MUC4, PRCC, DDX6, RB1, TBL1XR1, SFRP4, PRCC, DDX6, HOXC13, RB1, TBL1XR1, SFRP4, AR, PRCC, DDX6, RB1, TBL1XR1, MUC4, PRCC, DDX6, HOXC13, RB1, TBL1XR1, SFRP4, AR, PRCC, DDX6, RB1, TBL1XR1, MUC4, EPHA7, PRCC, DDX6, RB1, TBL1XR1, SFRP4, AR, EPHA7, PRCC, DDX6, RB1, TBL1XR1, MUC4, PRCC, DDX6, RB1, TBL1XR1, MUC4, PRCC, DDX6, RB1, TBL1XR1, AACSP1, AC004053.1, AC004053.1, AC004791.2, AC004901.1, AC007277.3, AC007879.5, AC007879.5, AC009120.6, AC012501.2, AC015922.5, AC016907.3, AC024908.1, AC027612.3, AC027612.3, AC061961.2, AC079610.1, AC079610.1, AC091878.1, AC097467.2, AC097468.7, AC102953.4, AC144521.1, ACE, ADCY1, ADPRHL2, ADRBK2, AF186996.1, AL133247.2, AL159977.1, AL162511.1, AL353791.1, AL590726.1, AL928742.2, ANGPTL7, ANKRD20A1, ANKRD26P1, ANKRD6, AOC1, AP000320.7, AP000765.1, AP001525.1, AP002954.4, ARHGEF26-AS1, ARHGEF37, BAGE2, BCORP1, BRINP2, C11orf84, C2orf16, C6, CAB39L, CASC8, CCT6P3, CDHR5, CLRN1-AS1, COL26A1, CPNE4, CSTF3-AS1, CTB-186H2.2, CTBP1-AS2, CTC-260E6.6, CTC-297N7.5, CTC-340A15.2, CTD-2001E22.1, CTD-2015H6.3, CTD-2176I21.2, CTD-2215L10.1, CTD-2269F5.1, CTD-2307P3.1, CTD-3006G17.2, CTD-3006G17.2, CTD-3006G17.2, CTD-3006G17.2, CTNNA3, CXXC1P1, CXXC4, DARC, DISC1FP1, DISC1FP1, DISC1FP1, DLG2, ELOVL2, ENTPD3-AS1, EPHA1-AS1, ERMP1, FAM120AOS, FAM153B, FAM181A-AS1, FAM184B, FAM230B, FETUB, FLG-AS1, FNDC1, FOLH1B, FREM2, GATAD2A, GBA3, GPR156, GPR4, GS1-256O22.5, GS1-256O22.5, GTF2H5, GTPBP10, GUCY1B2, HEATR6, HEG1, HHIPL1, HMX3, HTR5BP, IGHV3OR16-13, IGKV1D-37, IL12A-AS1, INHBA-AS1, INTS4, INTS4L1, KCNMB3P1, KIAA1257, KIAA1683, KIF25, KIRREL3-AS3, KLHL3, LINC00395, LINC00395, LINC00467, LINC00535, LINC00535, LINC00535, LINC00963, LINC01088, LMCD1-AS1, MEF2C-AS1, MEF2C-AS1, MEF2C-AS1, MIR31HG, MIR548H4, MIR596, MKLN1-AS1, MMP8, MSLN, MTHFD2P1, MTRNR2L5, MTSS1L, NELFA, NPSR1-AS1, NPSR1-AS1, NRGN, NT5DC4, OR5D18, OR8B8, PASD1, PCBP1-AS1, PCDHB3, PDE10A, PEG10, PEX7, PI3, PPFIA1, PPP1R35, PSTK, RFTN1P1, RHBDL3, RN7SKP227, RN7SL564P, RN7SL7P, RN7SL89P, RNA5SP358, RNA5SP403, RNF219-AS1, RNF219-AS1, RNU1-59P, RNU4-57P, RNU6-102P, RNU6-1047P, RNU6-442P, RNU6-52P, RNU6-543P, RNU6-656P, RP1-240B8.3, RP1-28O10.1, RP1-65P5.1, RP1-93I3.1, RP11-100L22.2, RP11-1017G21.4, RP11-1084E5.1, RP11-1103G16.1, RP11-1129I3.1, RP11-125B21.2, RP11-1277A3.2, RP11-13G14.4, RP11-13J10.1, RP11-142C4.6, RP11-144L1.4, RP11-145A3.1, RP11-158J3.2, RP11-159L20.2, RP11-166B2.8, RP11-17A1.3, RP11-17E2.2, RP11-17E2.2, RP11-17E2.2, RP11-231I13.2, RP11-231P20.2, RP11-23D24.2, RP11-274B21.1, RP11-280O1.2, RP11-285J16.1, RP11-324H6.5, RP11-326E22.1, RP11-32K4.1, RP11-32K4.1, RP11-32K4.1, RP11-343D2.11, RP11-379F4.4, RP11-3B12.1, RP11-3B12.1, RP11-420N3.2, RP11-420N3.2, RP11-420N3.2, RP11-420N3.2, RP11-431M7.2, RP11-431M7.3, RP11-439L18.3, RP11-445F12.1, RP11-446J8.1, RP11-454C18.2, RP11-513G19.1, RP11-525K10.3, RP11-525K10.3, RP11-526D8.7, RP11-538P18.2, RP11-541P9.3, RP11-541P9.3, RP11-550P17.5, RP11-550P17.5, RP11-551L14.1, RP11-556E13.1, RP11-586K2.1, RP11-586K2.1, RP11-58B2.1, RP11-624L4.1, RP11-624L4.1, RP11-649A16.1, RP11-649A16.1, RP11-692D12.1, RP11-692D12.1, RP11-702H23.4, RP11-706C16.8, RP11-707M1.1, RP11-707M1.1, RP11-742B18.1, RP11-744N12.3, RP11-752G15.9, RP11-75N4.2, RP11-770E5.1, RP11-770E5.1, RP11-788M5.3, RP11-804A23.2, RP11-820L6.1, RP11-86L19.2, RP11-978I15.10, RP13-492C18.2, RP13-492C18.2, RP3-323P13.2, RP3-399L15.3, RP4-630C24.3, RP5-1121H13.4, RP5-905H7.3, RPL23AP79, RPL23AP79, RPL23AP79, RPL23AP79, RPL23AP79, RPL23AP79, RPL23AP79, RPL23AP79, SCN4A, SCN4A, SEC14L4, SEC24B-AS1, SLAMF6, SLC18A2, SLC26A2, SLC31A1, SLC46A1, SLC5A3, SLC8A1, SLC8A1-AS1, SNAPC1, SNORA26, SNORA51, SNORD29, SNRPD2, SORL1, SRD5A3-AS1, SRGAP2B, SSPO, SUPT20H, TCHH, TFAP2B, TGS1, THSD1, TMEM200C, TMEM254-AS1, TMPRSS4-AS1, TMPRSS4-AS1, TNS3, TPTE2P1, TRAV27, TTN, TYW1B, USP15, WDR74, XRCC5, Y_RNA, Y_RNA, Y_RNA, Y_RNA, ZFHX4-AS1, ZFYVE21, ZNF207, ZNF527, ZNF665, ZNF844, ZNRD1-AS1, ZRANB2-AS1, ZRANB2-AS2, hsa-mir-490, snoU13, snoU13, snoU13, snoU13 | g.chr1:156737855C>T, g.chr11:118635963T>G, g.chr13:48937093G>C, g.chr3:176782763C>A, g.chr3:195510833G>A, g.chr1:156737855C>T, g.chr11:118635963T>G, g.chr13:48937093G>C, g.chr3:176782763C>A, g.chr7:37955824C>T, g.chr1:156737855C>T, g.chr11:118635963T>G, g.chr12:54338981C>T, g.chr13:48937093G>C, g.chr3:176782763C>A, g.chr7:37955824C>T, g.chrX:66943552A>G, g.chr1:156737855C>T, g.chr11:118635963T>G, g.chr13:48937093G>C, g.chr3:176782763C>A, g.chr3:195510833G>A, g.chr1:156737855C>T, g.chr11:118635963T>G, g.chr12:54338981C>T, g.chr13:48937093G>C, g.chr3:176782763C>A, g.chr7:37955824C>T, g.chrX:66943552A>G, g.chr1:156737855C>T, g.chr11:118635963T>G, g.chr13:48937093G>C, g.chr3:176782763C>A, g.chr3:195510833G>A, g.chr6:93950846_93950847insA, g.chr1:156737855C>T, g.chr11:118635963T>G, g.chr13:48937093G>C, g.chr3:176782763C>A, g.chr7:37955824C>T, g.chrX:66943552A>G, g.chr6:93950846_93950847insA, g.chr1:156737855C>T, g.chr11:118635963T>G, g.chr13:48937093G>C, g.chr3:176782763C>A, g.chr3:195510833G>A, g.chr1:156737855C>T, g.chr11:118635963T>G, g.chr13:48937093G>C, g.chr3:176782763C>A, g.chr3:195510833G>A, g.chr1:156737855C>T, g.chr11:118635963T>G, g.chr13:48937093G>C, g.chr3:176782763C>A, g.chr5:178206316G>A, g.chr4:105558990T>C, g.chr4:105564995T>C, g.chr19:15968570C>T, g.chr7:46991143G>C, g.chr2:171522445T>A, g.chr2:208145200T>C, g.chr2:208223262C>T, g.chr16:74347122T>C, g.chr2:154364884G>A, g.chr17:15754491A>C, g.chr2:30284027C>T, g.chr10:2492635T>C, g.chr2:91874637C>A, g.chr2:91878252C>T, g.chr2:155551215T>C, g.chr2:213446201A>G, g.chr2:213688752C>A, g.chr5:17170004delG, g.chr4:156257699A>G, g.chr2:219818241G>A, g.chr7:1504386C>A, g.chr3:18801032C>A, g.chr17:61557751G>A, g.chr7:45719309A>G, g.chr1:36556924G>A, g.chr22:26119048G>A, g.chr3:125453225G>A, g.chr2:31757329G>C, g.chr13:27892375T>G, g.chr14:36663296T>A, g.chr9:40029092A>G, g.chr9:30773609T>C, g.chr14:106059816C>T, g.chr1:11255668G>A, g.chr9:67938643A>G, g.chr16:46533885_46533886insT, g.chr6:90272351C>G, g.chr7:150554516G>A, g.chr21:35539587_35539588insC, g.chr11:94148067delT, g.chr18:13821808C>A, g.chr11:118614556C>A, g.chr3:153808753A>G, g.chr5:149013121G>A, g.chr21:11095601_11095602CC>AA, g.chrY:21650123C>T, g.chr1:177250332A>G, g.chr11:63580945C>T, g.chr2:27803164T>C, g.chr5:41181559_41181560insC, g.chr13:50008359C>A, g.chr8:128454361A>G, g.chr7:64538055G>C, g.chr11:619860G>A, g.chr3:150776145A>G, g.chr7:101132407_101132417delATCTTTGACAC, g.chr3:131306365G>A, g.chr11:33190283G>A, g.chr17:34288737C>T, g.chr4:1263761C>G, g.chr19:20401239G>C, g.chr17:10650546C>A, g.chr5:164372755G>A, g.chr5:9625518C>A, g.chr5:79780392A>T, g.chr5:58160501C>T, g.chr5:9007217G>A, g.chr5:83710437C>T, g.chr14:43007538A>T, g.chr14:27793484A>G, g.chr14:27909309_27909310insA, g.chr14:28028517C>T, g.chr14:28121721C>T, g.chr10:67678106T>A, g.chrX:47594333C>T, g.chr4:105389495C>A, g.chr1:159173199G>A, g.chr11:90362371G>A, g.chr11:90399305_90399306insC, g.chr11:90455817C>T, g.chr11:83166173C>T, g.chr6:10982314G>A, g.chr3:40474705G>A, g.chr7:143170798_143170799insA, g.chr9:5785700T>A, g.chr9:96215507A>G, g.chr5:175557727_175557728insT, g.chr14:94371689C>T, g.chr4:17690076G>A, g.chr22:21549338G>A, g.chr3:186370126G>A, g.chr1:152237736C>A, g.chr6:159660729C>A, g.chr11:89392833G>T, g.chr13:39262337C>T, g.chr19:19616682G>A, g.chr4:22804098G>T, g.chr3:119887076G>C, g.chr19:46094722C>T, g.chrX:142470260_142470261insT, g.chrX:142484151_142484152insA, g.chr6:158614418C>T, g.chr7:90017884A>T, g.chr13:51642887C>T, g.chr17:58134525G>A, g.chr3:124731996_124731997insA, g.chr14:100129357C>T, g.chr10:124896844C>T, g.chr2:118617900C>T, g.chr16:33627651G>A, g.chr2:89924960A>C, g.chr3:159758579T>C, g.chr7:41811524G>A, g.chr11:77618829T>C, g.chr7:64651980T>C, g.chr22:17061986A>G, g.chr3:128689017C>T, g.chr19:18368756C>T, g.chr6:168442776G>A, g.chr11:126876992C>A, g.chr5:136974648T>C, g.chr13:64254502T>C, g.chr13:64254540A>T, g.chr1:211597425T>A, g.chr8:94397910G>A, g.chr8:94541228C>T, g.chr8:94613121G>A, g.chr9:132253920C>A, g.chr4:79950739A>G, g.chr3:8381781G>A, g.chr5:88443649T>C, g.chr5:88443730A>G, g.chr5:88760910G>A, g.chr9:21496833G>A, g.chr8:26904853A>G, g.chr8:1765402G>C, g.chr7:130650301T>A, g.chr11:102583977_102583978delTT, g.chr16:816465A>G, g.chr3:95404891C>A, g.chr10:57358755C>A, g.chr16:70696564G>A, g.chr4:1985120C>A, g.chr7:34411726A>T, g.chr7:34483192T>A, g.chr11:124616569C>G, g.chr2:113488837C>A, g.chr11:55587626C>A, g.chr11:124310920G>A, g.chrX:150732139C>T, g.chr2:70298018C>A, g.chr5:140481478C>T, g.chr6:166075457C>G, g.chr7:94294007C>T, g.chr6:137187867A>G, g.chr20:43804694G>A, g.chr11:70189931C>T, g.chr7:100033921G>A, g.chr10:124742516G>A, g.chrY:7613522A>T, g.chr17:30611785T>C, g.chr3:36729601G>T, g.chr6:123068647G>C, g.chr7:92600146G>A, g.chr4:106212485G>T, g.chr12:38557386G>A, g.chr16:9659997C>T, g.chr13:78890633T>C, g.chr13:78938389A>T, g.chr1:144532957C>T, g.chr1:223547430delA, g.chr7:124286247A>C, g.chr3:126960385delG, g.chr8:126912505G>A, g.chr13:19714322T>C, g.chr10:64868319C>T, g.chr8:47740355C>T, g.chr6:62385079delA, g.chr1:209832298C>T, g.chr11:32363856G>T, g.chrX:114774698C>T, g.chr8:93749557G>A, g.chr14:102520793C>G, g.chr8:110032615_110032616insT, g.chr14:30951115C>A, g.chr15:56852431C>T, g.chr9:2448798A>G, g.chr5:177080745G>A, g.chr12:108862116G>A, g.chr2:105509722G>A, g.chr11:58774589_58774590GG>TA, g.chr1:158464000G>A, g.chr1:225941368G>A, g.chr5:63262166A>G, g.chr14:31276768G>C, g.chr16:11958843_11958845delGAA, g.chr11:22918350C>T, g.chr4:22070146C>T, g.chr4:22192378G>A, g.chr4:22204306C>T, g.chr3:70302227G>C, g.chr1:26554424G>A, g.chr3:153118892T>G, g.chr7:128270833C>T, g.chr1:165548240_165548241insA, g.chr3:37222310C>T, g.chr10:51797822delC, g.chr8:72106336C>T, g.chr8:64945837A>G, g.chr8:64953943C>G, g.chr8:65151600C>A, g.chr3:97822626G>A, g.chr3:158485571C>T, g.chr7:124637064G>T, g.chr7:124752844A>G, g.chr16:5585218C>T, g.chr16:5697075A>C, g.chr16:5898460C>A, g.chr16:5997027A>G, g.chr4:36386922_36386923insT, g.chr4:36257013T>A, g.chr6:143325896G>A, g.chr17:35258460C>T, g.chr4:16409984G>A, g.chr3:151638418C>G, g.chr12:26483179G>T, g.chr16:80191483C>T, g.chr16:80465546C>T, g.chr9:95650752C>T, g.chr3:158279335delG, g.chr5:162452218C>T, g.chr5:162497988G>A, g.chr1:159380739G>A, g.chr1:159392327G>A, g.chr12:31264086C>T, g.chr10:54497064G>A, g.chr8:89520867C>A, g.chr8:89580524T>C, g.chr5:101838721C>A, g.chr15:39220242C>G, g.chr15:39337723A>G, g.chr3:146762428G>A, g.chr3:146809234C>T, g.chr4:74138282C>T, g.chr4:74170811A>G, g.chr11:74116880C>T, g.chr8:143892731T>C, g.chr11:49708078delA, g.chr11:49735246G>A, g.chr4:88589393T>C, g.chr11:128502317T>A, g.chr15:83394179T>G, g.chr18:29135009T>C, g.chr8:49542874G>C, g.chr8:49542902G>C, g.chr11:101063552G>T, g.chr11:60601910C>G, g.chr11:122169903T>C, g.chr9:107035194T>A, g.chr1:247797195G>A, g.chr7:56509630C>T, g.chr7:56517893C>A, g.chr6:133879375C>T, g.chr6:114763544C>A, g.chr7:123652942T>C, g.chr20:40688834C>A, g.chr7:62690798C>G, g.chr19:59097579G>C, g.chr19:59097623G>C, g.chr19:59097677G>C, g.chr19:59097754G>C, g.chr19:59097781G>A, g.chr19:59097848G>C, g.chr19:59098063G>T, g.chr19:59098153G>C, g.chr17:62034677G>A, g.chr17:62050108C>T, g.chr22:30885840A>G, g.chr4:110280894A>C, g.chr1:160456458A>G, g.chr10:119029638G>A, g.chr5:149360067A>T, g.chr9:116024560G>C, g.chr17:26732953G>A, g.chr21:35470575T>A, g.chr2:40337916G>A, g.chr2:40230216G>A, g.chr14:62233722A>G, g.chr3:53419001T>C, g.chr8:60047712G>A, g.chr5:24808792G>T, g.chr19:46195530C>A, g.chr11:121437724_121437725insCT, g.chr4:56248596A>G, g.chr1:144005851_144005852insT, g.chr7:149477883C>T, g.chr13:37586392delC, g.chr1:152081633G>A, g.chr6:50811606C>G, g.chr8:56695349C>G, g.chr13:52951668C>G, g.chr18:5895893C>A, g.chr10:81811513C>T, g.chr11:117891951G>A, g.chr11:117927408C>T, g.chr7:47409154C>T, g.chr13:25543141C>G, g.chr14:22616378A>G, g.chr2:179650719G>A, g.chr7:72081337T>G, g.chr12:62799150T>C, g.chr11:62609254G>A, g.chr2:216977853G>C, g.chr1:111308388_111308389insA, g.chr6:88537093A>T, g.chr7:72819446G>T, g.chr8:114964737G>C, g.chr8:77546221C>T, g.chr14:104195487T>C, g.chr17:30705128T>A, g.chr19:37880747G>A, g.chr19:53667683G>A, g.chr19:12190004A>G, g.chr6:29980858C>A, g.chr1:71520545T>A, g.chr1:71678401C>T, g.chr7:136453393G>T, g.chr10:70470748G>T, g.chr21:43027820C>T, g.chr5:50618476C>G, g.chr9:135966651G>A] | Prost |
| DO51955 | COL2A1, CTNND2, AFF4, GOPC, CNTNAP2, FAM135B, CDH1, COL2A1, CTNND2, AFF4, GOPC, CNTNAP2, FAM135B, CDH1 | g.chr12:48367304G>A, g.chr5:11082827G>T, g.chr5:132214909C>A, g.chr6:117894656G>A, g.chr7:148118069A>G, g.chr8:139142502G>C, g.chr16:68846080delC, g.chr12:48367304G>A, g.chr5:11082827G>T, g.chr5:132214909C>A, g.chr6:117894656G>A, g.chr7:148118069A>G, g.chr8:139142502G>C, g.chr16:68846080delC | Prost |
| DO51956 | ASXL2, FAT1, ASXL2, FAT1, ASXL2, ASXL2, FAT1, ASXL2, FAT1 | g.chr2:25982508_25982509AT>TA, g.chr4:187628408C>T, g.chr2:25982508_25982509AT>TA, g.chr4:187628408C>T, g.chr2:25982508_25982509AT>TA, g.chr2:25982508_25982509AT>TA, g.chr4:187628408C>T, g.chr2:25982508_25982509AT>TA, g.chr4:187628408C>T | Prost |
| DO51958 | NBEA, TP53, CTNNA2, TBL1XR1, CSMD3, MLLT3, SNX29, RABEP1, PMS2, NBEA, TP53, CTNNA2, CTNNB1, TBL1XR1, SGK1, CSMD3, MLLT3, SNX29, RABEP1, NBEA, TP53, CTNNA2, CTNNB1, TBL1XR1, SGK1, CSMD3, CSMD3, CSMD3, MLLT3, SNX29, RABEP1, NBEA, TP53, CTNNA2, CTNNB1, TBL1XR1, SGK1, CSMD3, CSMD3, CSMD3, MLLT3, SNX29, RABEP1, PMS2, NBEA, TP53, CTNNA2, CTNNB1, TBL1XR1, SGK1, CSMD3, MLLT3, SNX29, RABEP1 | g.chr13:36220423G>A, g.chr17:7578534C>A, g.chr2:80831250A>T, g.chr3:176755884A>T, g.chr8:113331102G>A, g.chr9:20342019C>A, g.chr16:12136785delC, g.chr17:5212109_5212113delCTAAA, g.chr7:6022500_6022507delTCGAAGTT, g.chr13:36220423G>A, g.chr17:7578534C>A, g.chr2:80831250A>T, g.chr3:41266113C>G, g.chr3:176755884A>T, g.chr6:134581872T>G, g.chr8:113331102G>A, g.chr9:20342019C>A, g.chr16:12136785delC, g.chr17:5212109_5212113delCTAAA, g.chr13:36220423G>A, g.chr17:7578534C>A, g.chr2:80831250A>T, g.chr3:41266113C>G, g.chr3:176755884A>T, g.chr6:134581872T>G, g.chr8:113299415C>A, g.chr8:113323391G>C, g.chr8:113331102G>A, g.chr9:20342019C>A, g.chr16:12136785delC, g.chr17:5212109_5212113delCTAAA, g.chr13:36220423G>A, g.chr17:7578534C>A, g.chr2:80831250A>T, g.chr3:41266113C>G, g.chr3:176755884A>T, g.chr6:134581872T>G, g.chr8:113299415C>A, g.chr8:113323391G>C, g.chr8:113331102G>A, g.chr9:20342019C>A, g.chr16:12136785delC, g.chr17:5212109_5212113delCTAAA, g.chr7:6022500_6022507delTCGAAGTT, g.chr13:36220423G>A, g.chr17:7578534C>A, g.chr2:80831250A>T, g.chr3:41266113C>G, g.chr3:176755884A>T, g.chr6:134581872T>G, g.chr8:113331102G>A, g.chr9:20342019C>A, g.chr16:12136785delC, g.chr17:5212109_5212113delCTAAA | Prost |
| DO51959 | VAV1, DGCR8, NF2, ROBO2, STAG2, CDH11, ZFHX3, TP53, LEF1, KMT2C, VAV1, DGCR8, NF2, ROBO2, STAG2, CDH11, ZFHX3, TP53, LEF1, KMT2C, VAV1, DGCR8, NF2, STAG2, CDH11, ZFHX3, TP53, LEF1, KMT2C | g.chr19:6828470A>G, g.chr22:20099223C>T, g.chr22:29999585C>A, g.chr3:77637908G>A, g.chrX:123234734A>T, g.chr16:65005926delC, g.chr16:72828623_72828624insGTTGTTCCGGTGTGATG, g.chr17:7577566_7577567insA, g.chr4:108969328_108969329insA, g.chr7:152007060_152007068delCCCTGAGAC, g.chr19:6828470A>G, g.chr22:20099223C>T, g.chr22:29999585C>A, g.chr3:77637908G>A, g.chrX:123234734A>T, g.chr16:65005926delC, g.chr16:72828623_72828624insGTTGTTCCGGTGTGATG, g.chr17:7577566_7577567insA, g.chr4:108969328_108969329insA, g.chr7:152007060_152007068delCCCTGAGAC, g.chr19:6828470A>G, g.chr22:20099223C>T, g.chr22:29999585C>A, g.chrX:123234734A>T, g.chr16:65005926delC, g.chr16:72828623_72828624insGTTGTTCCGGTGTGATG, g.chr17:7577566_7577567insA, g.chr4:108969328_108969329insA, g.chr7:152007060_152007068delCCCTGAGAC | Prost |
| DO51960 | RMI2, SPECC1, NF1, CD209, EML4, KIAA1549, AR, PTEN, TP53, ASXL2, RMI2, SPECC1, NF1, CD209, EML4, KIAA1549, AR, PTEN, TP53, ASXL2, RMI2, SPECC1, NF1, CD209, EML4, KIAA1549, AR, PTEN, TP53, ASXL2, RMI2, SPECC1, NF1, CD209, EML4, KIAA1549, AR, PTEN, TP53, ASXL2 | g.chr16:11445008C>G, g.chr17:19990336G>T, g.chr17:29546053G>A, g.chr19:7806355C>T, g.chr2:42558510C>G, g.chr7:138554407T>C, g.chrX:66943552A>G, g.chr10:89720817_89720819delATG, g.chr17:7578222_7578223delTC, g.chr2:26101088delT, g.chr16:11445008C>G, g.chr17:19990336G>T, g.chr17:29546053G>A, g.chr19:7806355C>T, g.chr2:42558510C>G, g.chr7:138554407T>C, g.chrX:66943552A>G, g.chr10:89720817_89720819delATG, g.chr17:7578222_7578223delTC, g.chr2:26101088delT, g.chr16:11445008C>G, g.chr17:19990336G>T, g.chr17:29546053G>A, g.chr19:7806355C>T, g.chr2:42558510C>G, g.chr7:138554407T>C, g.chrX:66943552A>G, g.chr10:89720817_89720819delATG, g.chr17:7578222_7578223delTC, g.chr2:26101088delT, g.chr16:11445008C>G, g.chr17:19990336G>T, g.chr17:29546053G>A, g.chr19:7806355C>T, g.chr2:42558510C>G, g.chr7:138554407T>C, g.chrX:66943552A>G, g.chr10:89720817_89720819delATG, g.chr17:7578222_7578223delTC, g.chr2:26101088delT | Prost |
| DO51962 | ARID1A, RB1, SOX21, KTN1, AKT1, SNX29, MAF, ASXL2, CTNNA2, ERBB4, TGFBR2, FAT4, NRG1, CSMD3, MLLT3, STIL, CEBPA, CEBPA, CEBPA, ARID1A, RB1, SOX21, KTN1, AKT1, SNX29, SNX29, MAF, ASXL2, CTNNA2, ERBB4, TGFBR2, FAT4, MACC1, NRG1, CSMD3, MLLT3, STIL, CEBPA, CEBPA, CEBPA, QKI, ARID1A, RB1, SOX21, KTN1, AKT1, SNX29, SNX29, MAF, ASXL2, CTNNA2, ERBB4, TGFBR2, FAT4, MACC1, NRG1, CSMD3, MLLT3, STIL, CEBPA, QKI | g.chr1:27099947C>T, g.chr13:48954333C>T, g.chr13:95362232T>G, g.chr14:56119799A>C, g.chr14:105259106G>C, g.chr16:12667843C>G, g.chr16:79633971C>A, g.chr2:26101043C>A, g.chr2:80620343G>T, g.chr2:212989624A>G, g.chr3:30735289G>C, g.chr4:126238553C>T, g.chr8:31497509G>C, g.chr8:113323376T>C, g.chr9:20342793C>G, g.chr1:47728681delC, g.chr19:33792654_33792663delCGGGCTGCAG, g.chr19:33792649_33792653delGTGAC, g.chr19:33792659_33792663delTGCAG, g.chr1:27099947C>T, g.chr13:48954333C>T, g.chr13:95362232T>G, g.chr14:56119799A>C, g.chr14:105259106G>C, g.chr16:12093153G>T, g.chr16:12667843C>G, g.chr16:79633971C>A, g.chr2:26101043C>A, g.chr2:80620343G>T, g.chr2:212989624A>G, g.chr3:30735289G>C, g.chr4:126238553C>T, g.chr7:20178057C>T, g.chr8:31497509G>C, g.chr8:113323376T>C, g.chr9:20342793C>G, g.chr1:47728681delC, g.chr19:33792654_33792663delCGGGCTGCAG, g.chr19:33792649_33792653delGTGAC, g.chr19:33792659_33792663delTGCAG, g.chr6:163996834_163996835delGT, g.chr1:27099947C>T, g.chr13:48954333C>T, g.chr13:95362232T>G, g.chr14:56119799A>C, g.chr14:105259106G>C, g.chr16:12093153G>T, g.chr16:12667843C>G, g.chr16:79633971C>A, g.chr2:26101043C>A, g.chr2:80620343G>T, g.chr2:212989624A>G, g.chr3:30735289G>C, g.chr4:126238553C>T, g.chr7:20178057C>T, g.chr8:31497509G>C, g.chr8:113323376T>C, g.chr9:20342793C>G, g.chr1:47728681delC, g.chr19:33792654_33792663delCGGGCTGCAG, g.chr6:163996834_163996835delGT | Prost |
| DO51964 | TRIM33, PDE4DIP, LCP1, SPOP, BCR, KAT6A, TRIM33, PDE4DIP, LMNA, LCP1, SPOP, BCR, KAT6A, AR, TRIM33, PDE4DIP, LCP1, SPOP, PIK3CA, KAT6A, TRIM33, PDE4DIP, LMNA, LCP1, SPOP, BCR, KAT6A, AR, TRIM33, PDE4DIP, LMNA, LCP1, SPOP, BCR, KAT6A, AR, TRIM33, PDE4DIP, LMNA, LCP1, SPOP, KAT6A, AR, TRIM33, PDE4DIP, LCP1, SPOP, BCR, KAT6A, AR, TRIM33, PDE4DIP, LMNA, LCP1, SPOP, BCR, KAT6A, AR, TRIM33, PDE4DIP, LMNA, LCP1, SPOP, BCR, KAT6A, AR, ABCC4, AC004041.2, AC004458.1, AC004538.3, AC004538.3, AC005062.2, AC005592.2, AC006322.1, AC007277.3, AC007319.1, AC007319.1, AC007879.5, AC008836.1, AC009236.1, AC009677.1, AC012363.4, AC016725.4, AC073479.1, AC079135.1, AC079610.1, AC079610.1, AC079613.1, AC079613.1, AC079630.4, AC083906.1, AC092846.2, AC093874.1, AC096579.13, AC097467.2, AC098617.1, AC108696.1, AC138761.1, ADAL, AF121898.3, AF121898.3, AF121898.3, AL139815.1, AL359709.2, ALDH9A1, ANKRD12, AP000320.7, AP001597.1, ARHGAP11B, ARHGEF40, ASNA1, ATG5, BAGE2, BAGE2, BAGE2, BPIFA4P, C10orf12, C17orf80, CD2AP, CDC42SE2, CDH23, CDKN2B-AS1, CHIC1, CNGA1, COL24A1, COL5A3, CTB-49A3.2, CTC-340A15.2, CTC-340A15.2, CTC-340A15.2, CTC-340A15.2, CTC-457E21.9, CTC-525D6.1, CTC-525D6.1, CTD-2269F5.1, CTD-2547L16.1, CTD-3006G17.2, CTD-3006G17.2, CTD-3006G17.2, CTD-3088G3.8, CTTNBP2, CYP4B1, DCAF4L2, DDX11-AS1, DDX50, DDX60, DIO2-AS1, DIRC3, DISC1FP1, DOCK5, DUOX2, EFCAB1, EGFEM1P, EHHADH-AS1, EIF4E, EMILIN1, EPHA1-AS1, EPSTI1, ERICH1-AS1, ESPNP, ESRRAP2, F11-AS1, FAIM2, FAM177A1, FBN1, FBXL5, FBXO28, FLG-AS1, FZD10-AS1, GJD2, GLUD1P2, GPD2, GPR133, GPT, GRID1, GRIPAP1, GS1-256O22.5, GUSBP1, HCG18, HCN4, HEBP2, HIST1H1A, HMGCS1, HNRNPA1P48, IGFBP7-AS1, IGHD3-10, IGHV1-24, IGKV1-27, IGLV7-46, IL12A-AS1, INHBA-AS1, ITGB2, KB-1562D12.1, KCNB2, KCNV1, KHDRBS3, KIAA2022, KLF11, KNTC1, LEF1-AS1, LINC00189, LINC00395, LINC00535, LINC00535, LINC00535, LMTK2, LRP3, LRRC49, MAGI3, MAMDC2, MCC, MCHR2-AS1, MEF2C-AS1, MIR184, MIR3622A, MIR496, MLK7-AS1, MRPL45P2, MYO18A, NALCN-AS1, NOVA1-AS1, OIT3, PDE3A, PICK1, PIK3CG, PPIP5K2, PROX1-AS1, PVRL4, RAD1, RAPGEFL1, RGS18, RN7SL170P, RN7SL251P, RN7SL357P, RN7SL572P, RN7SL600P, RN7SL637P, RN7SL649P, RNA5SP116, RNA5SP504, RNA5SP62, RNF217, RNF219-AS1, RNF219-AS1, RNMT, RNPC3, RNU6-1070P, RNU6-243P, RNU6-741P, RNU7-136P, RNY4P27, RP1-167F1.2, RP11-1069G10.1, RP11-115D19.1, RP11-150C16.1, RP11-152L20.3, RP11-152L20.3, RP11-152L20.3, RP11-152L20.3, RP11-154D6.1, RP11-163M18.1, RP11-175P19.2, RP11-17E2.2, RP11-17E2.2, RP11-196E1.3, RP11-32K4.1, RP11-32K4.1, RP11-32K4.1, RP11-32K4.1, RP11-32K4.1, RP11-33N16.3, RP11-342D14.1, RP11-357C3.3, RP11-357C3.3, RP11-366F6.2, RP11-368M16.3, RP11-370I10.6, RP11-385J1.2, RP11-3B12.1, RP11-420N3.2, RP11-420N3.2, RP11-420N3.2, RP11-420N3.2, RP11-420N3.2, RP11-422J8.1, RP11-423H2.3, RP11-429B14.1, RP11-435M3.2, RP11-439L18.3, RP11-505P4.7, RP11-519G16.3, RP11-532F12.5, RP11-541P9.3, RP11-541P9.3, RP11-54D18.3, RP11-550P17.5, RP11-562L8.1, RP11-572M11.4, RP11-586K2.1, RP11-586K2.1, RP11-586K2.1, RP11-611E13.2, RP11-624C23.1, RP11-624C23.1, RP11-634B7.4, RP11-649A16.1, RP11-665G4.1, RP11-66D17.5, RP11-678G14.2, RP11-679C8.2, RP11-679C8.2, RP11-76N22.2, RP11-804N13.1, RP11-820L6.1, RP11-826N14.1, RP11-89K10.1, RP11-92C4.3, RP11-93K22.6, RP11-98D18.15, RP13-492C18.2, RP3-340N1.5, RP3-399L15.3, RP3-399L15.3, RP4-651E10.4, RP4-694A7.4, RP5-896L10.1, RP5-896L10.1, RP5-991G20.1, RUNDC1, SAGE1, SCRN1, SEPT14, SHANK2, SIGLEC6, SLC10A7, SLC16A10, SLC25A32, SLC25A38, SLC30A2, SLC7A14, SLFN5, SNORA26, SRD5A2, STARD13, SUCO, TAOK3, TCF21, TENC1, TEX26-AS1, TMEM154, TMEM200C, TMEM38B, TRAPPC3L, TRAV27, TRBV6-8, TRIM69, U3, U8, UBE2Q2, UFL1-AS1, UMODL1, UNG, URB1, UVRAG, VNN2, VSTM4, VTI1B, WDR5B, Y_RNA, Y_RNA, Y_RNA, Z95704.4, ZBED3-AS1, ZBED5-AS1, ZBTB38, ZFHX4-AS1, ZFHX4-AS1, ZFHX4-AS1, ZNF106, ZNF341, ZNF815P, ZNF827, snoU13, snoU13 | g.chr1:114939089A>G, g.chr1:144851616A>C, g.chr13:46701701G>A, g.chr17:47696643A>C, g.chr22:23658652A>T, g.chr8:41907140G>A, g.chr1:114939089A>G, g.chr1:144851616A>C, g.chr1:156109658G>T, g.chr13:46701701G>A, g.chr17:47696643A>C, g.chr22:23658652A>T, g.chr8:41907140G>A, g.chrX:66765158_66765159insGCAGCA, g.chr1:114939089A>G, g.chr1:144851616A>C, g.chr13:46701701G>A, g.chr17:47696643A>C, g.chr3:178936094C>A, g.chr8:41907140G>A, g.chr1:114939089A>G, g.chr1:144851616A>C, g.chr1:156109658G>T, g.chr13:46701701G>A, g.chr17:47696643A>C, g.chr22:23658652A>T, g.chr8:41907140G>A, g.chrX:66931463T>A, g.chr1:114939089A>G, g.chr1:144851616A>C, g.chr1:156109658G>T, g.chr13:46701701G>A, g.chr17:47696643A>C, g.chr22:23658652A>T, g.chr8:41907140G>A, g.chrX:66931463T>A, g.chr1:114939089A>G, g.chr1:144851616A>C, g.chr1:156109658G>T, g.chr13:46701701G>A, g.chr17:47696643A>C, g.chr8:41907140G>A, g.chrX:66765158_66765159insGCAGCA, g.chr1:114939089A>G, g.chr1:144851616A>C, g.chr13:46701701G>A, g.chr17:47696643A>C, g.chr22:23658652A>T, g.chr8:41907140G>A, g.chrX:66765158_66765159insGCAGCA, g.chr1:114939089A>G, g.chr1:144851616A>C, g.chr1:156109658G>T, g.chr13:46701701G>A, g.chr17:47696643A>C, g.chr22:23658652A>T, g.chr8:41907140G>A, g.chrX:66931463T>A, g.chr1:114939089A>G, g.chr1:144851616A>C, g.chr1:156109658G>T, g.chr13:46701701G>A, g.chr17:47696643A>C, g.chr22:23658652A>T, g.chr8:41907140G>A, g.chrX:66931463T>A, g.chr13:95735408C>A, g.chr5:131985150C>A, g.chr7:96097189G>A, g.chr7:11336001A>G, g.chr7:11384243delT, g.chr7:19955954A>T, g.chr5:141926728A>G, g.chr7:83547927A>G, g.chr2:171529211G>A, g.chr2:187891882T>A, g.chr2:188060827G>T, g.chr2:208197420_208197421insT, g.chr5:60854093A>C, g.chr2:45444954G>T, g.chr15:70024167G>A, g.chr2:120943173C>T, g.chr2:135586313G>T, g.chr2:6127593_6127594insA, g.chr2:237098161G>A, g.chr2:213611794G>C, g.chr2:213804900G>A, g.chr2:189493247G>C, g.chr2:189552850G>T, g.chr12:40583759_40583760insA, g.chr3:129752696G>A, g.chr4:24430763_24430764insA, g.chr4:167147027_167147028insT, g.chr2:89137232C>T, g.chr4:156301342T>C, g.chr2:192746028G>A, g.chr3:84340560C>T, g.chr17:21819183C>A, g.chr15:43627279G>A, g.chr8:88555808A>G, g.chr8:88559462G>T, g.chr8:88652650T>C, g.chr10:23362997T>G, g.chr6:105644763A>C, g.chr1:165664551A>G, g.chr18:9255858G>T, g.chr21:35702378T>A, g.chr21:27801586G>T, g.chr15:31062570C>G, g.chr14:21543066C>G, g.chr19:12849387C>G, g.chr6:106632829T>C, g.chr21:11045424T>A, g.chr21:11051459T>A, g.chr21:11090986G>A, g.chr20:31786727G>A, g.chr10:98742781G>A, g.chr17:71232594C>T, g.chr6:47541818C>T, g.chr5:130728095T>G, g.chr10:73467024A>G, g.chr9:22089239A>T, g.chrX:72797277C>T, g.chr4:47939078C>T, g.chr1:86453307G>A, g.chr19:10078712delG, g.chr5:132520616C>A, g.chr5:163866279A>G, g.chr5:163883657G>A, g.chr5:164306783A>G, g.chr5:164530606A>G, g.chr19:22886310G>T, g.chr19:29932578T>C, g.chr19:30007203C>A, g.chr5:83707662T>G, g.chr8:17950924C>T, g.chr14:27766746G>A, g.chr14:27830150G>A, g.chr14:27923558_27923559insT, g.chr16:11582655G>A, g.chr7:117375020C>T, g.chr1:47284682C>A, g.chr8:88883299C>G, g.chr12:31180213G>A, g.chr10:70666501C>T, g.chr4:169201551T>C, g.chr14:80798549C>A, g.chr2:218464420_218464421insG, g.chr11:90493020G>T, g.chr8:25265530G>T, g.chr15:45402722delC, g.chr8:49624213delC, g.chr3:168531424delA, g.chr3:184880655C>A, g.chr4:99795868_99795869insA, g.chr2:27303045G>A, g.chr7:143193651T>C, g.chr13:43538257G>A, g.chr8:786232T>C, g.chr1:17044913T>A, g.chr13:21850369T>C, g.chr4:187316970A>G, g.chr12:50261086C>A, g.chr14:35515329G>A, g.chr15:48800902C>T, g.chr4:15627071delA, g.chr1:224345773T>G, g.chr1:152157154G>A, g.chr12:130641669C>T, g.chr15:35043361C>A, g.chr10:48980432C>T, g.chr2:157440193G>A, g.chr12:131624781C>A, g.chr8:145730394_145730406delCAGCTCCGGCATC, g.chr10:87628834G>A, g.chrX:48837715G>C, g.chrX:142452997_142453002delATAACT, g.chr5:21543758T>G, g.chr6:30278337C>T, g.chr15:73617654G>A, g.chr6:138740926T>G, g.chr6:26017895C>T, g.chr5:43289599G>A, g.chr16:51677279G>C, g.chr4:58029032G>C, g.chr14:106371731G>T, g.chr14:106732167A>T, g.chr2:89509919C>G, g.chr22:22722914T>C, g.chr3:159879103G>T, g.chr7:41816080C>A, g.chr21:46340881G>A, g.chr8:102492228_102492229insT, g.chr8:73849957C>A, g.chr8:110979679C>G, g.chr8:136569796C>T, g.chrX:73965444G>A, g.chr2:10193739C>T, g.chr12:123110771C>T, g.chr4:109113804C>T, g.chr21:30610488C>G, g.chr13:64275293_64275294insT, g.chr8:94586379T>C, g.chr8:94589936T>C, g.chr8:94592559G>C, g.chr7:97788729_97788730delTG, g.chr19:33698515C>G, g.chr15:71188277T>C, g.chr1:114228102C>T, g.chr9:72758700G>A, g.chr5:112478999C>T, g.chr6:100507644T>A, g.chr5:88547271_88547272insT, g.chr15:79501873delA, g.chr8:27558089A>G, g.chr14:101524446T>C, g.chr2:174143718T>C, g.chr17:45545006G>A, g.chr17:27493919C>T, g.chr13:101395159G>A, g.chr14:27119431T>C, g.chr10:74692242C>T, g.chr12:20803441G>C, g.chr22:38453582C>T, g.chr7:106509420C>T, g.chr5:102503773_102503776delTAGA, g.chr1:214002964C>G, g.chr1:161059345C>A, g.chr5:34916687C>T, g.chr17:38345135A>G, g.chr1:192153671C>A, g.chr20:55265112C>T, g.chr2:85667223T>C, g.chr4:57672312T>G, g.chr5:23202041delA, g.chr1:150542819C>T, g.chr3:185702278T>C, g.chr1:12097177G>A, g.chr2:208916334A>C, g.chrX:52696646G>C, g.chr1:163437612T>A, g.chr6:125406256A>C, g.chr13:78774131C>A, g.chr13:79131471A>T, g.chr18:13762556C>T, g.chr1:104095982_104095983insT, g.chr7:73675214G>A, g.chr3:35297578_35297579insT, g.chr15:24024873T>C, g.chr3:160192519T>C, g.chr13:95986414C>T, g.chr6:19643473C>A, g.chr15:63167542G>T, g.chr4:90635940G>A, g.chr12:59391680G>C, g.chr15:92762618C>T, g.chr15:92766612_92766613insA, g.chr15:92803251G>A, g.chr15:92855946A>G, g.chr6:71987232C>T, g.chr14:44991621C>A, g.chr3:193297692A>T, g.chr4:22123927C>T, g.chr4:22154379A>T, g.chr11:119487517G>A, g.chr8:64763071_64763072insT, g.chr8:64933877G>C, g.chr8:64978251_64978252insA, g.chr8:65010483G>T, g.chr8:65152787G>A, g.chr14:90239908G>A, g.chrX:45075863G>A, g.chrX:62677659A>G, g.chrX:62763857G>A, g.chrX:151070944C>A, g.chr7:57706048G>A, g.chr12:48766322A>G, g.chr3:178566031T>C, g.chr7:124575131A>G, g.chr16:5303728T>G, g.chr16:5757213A>T, g.chr16:5867707T>A, g.chr16:5882500T>G, g.chr16:5882864G>A, g.chr1:37924728C>G, g.chr5:177382224T>C, g.chr15:90071196T>A, g.chr10:26217596G>A, g.chr6:143289122_143289123insA, g.chr6:74272285G>A, g.chr15:45762746C>T, g.chr15:41126662C>T, g.chr5:162522102A>C, g.chr5:162661309T>C, g.chr9:15063326_15063327insA, g.chr1:159382781_159382782insA, g.chr14:29835730T>A, g.chr3:112898411_112898412insT, g.chr8:89455629A>G, g.chr8:89584217C>A, g.chr8:89725010C>T, g.chr12:70498536T>C, g.chr8:24596655G>T, g.chr8:24611226A>G, g.chr1:247864631G>A, g.chr3:147055146C>A, g.chr4:15078875_15078876insT, g.chr1:156682972C>T, g.chr19:21749069G>C, g.chr4:121020903T>C, g.chr4:121117127T>A, g.chr1:89044883C>G, g.chr5:21721262C>A, g.chr11:122227442_122227443insT, g.chr5:175468205G>C, g.chr8:127689831G>A, g.chr9:101627162T>C, g.chr3:129677222C>G, g.chr1:151726921A>T, g.chr7:56493837C>A, g.chr1:20508228T>A, g.chr6:114340384G>A, g.chr6:114702618G>A, g.chr1:87101486T>G, g.chr1:68989214T>G, g.chr1:99568954A>G, g.chr1:99593370G>T, g.chr16:72704516T>C, g.chr17:41142343G>C, g.chrX:134993427G>T, g.chr7:29994958C>T, g.chr7:55861246C>A, g.chr11:70333538T>C, g.chr19:52033107C>A, g.chr4:147215081_147215105delCTTATGAACAGTATGAGAACAAGGC, g.chr6:111550550G>A, g.chr8:104415396T>C, g.chr3:39437887A>C, g.chr1:26369117C>T, g.chr3:170177529T>C, g.chr17:33599700G>A, g.chr7:152308675T>G, g.chr2:31808770A>C, g.chr13:33678510T>C, g.chr1:172571221C>T, g.chr12:118673402A>G, g.chr6:134213672G>A, g.chr12:53443865G>A, g.chr13:31476381T>C, g.chr4:153601292delG, g.chr18:5883592G>A, g.chr9:108537065_108537066insT, g.chr6:116866676_116866680delATAGT, g.chr14:22617983G>A, g.chr7:142125463A>G, g.chr15:45021417_45021418insA, g.chr8:43232694C>G, g.chr1:234731977T>C, g.chr15:76168554T>C, g.chr6:96918417G>A, g.chr21:43547906G>A, g.chr12:109547776C>G, g.chr21:33686100C>T, g.chr11:75599940A>G, g.chr6:133077069G>T, g.chr10:50285262A>T, g.chr14:68120171G>A, g.chr3:122131893C>A, g.chr12:38670808T>A, g.chr2:121799864G>A, g.chr3:88480682C>A, g.chr4:49956C>T, g.chr5:76430330C>T, g.chr11:10885760C>T, g.chr3:141105724A>G, g.chr8:77476964C>A, g.chr8:77485454_77485455insT, g.chr8:77516099C>T, g.chr15:42708104A>G, g.chr20:32357923A>C, g.chr7:5874411_5874412insCCG, g.chr4:146824252C>T, g.chr18:49072278T>C, g.chrX:141958745C>T] | Prost |
| DO51965 | ELF3, KDM5A, ALDH2, HSP90AA1, USP8, COL1A1, CTNNA2, PBRM1, TET2, CDH10, SGK1, KIAA1549, EIF1AX, STAG2, ARID2, TCF12, TMPRSS2, CTNNB1, ELF3, KDM5A, ALDH2, HSP90AA1, USP8, COL1A1, CTNNA2, PBRM1, TET2, CDH10, SGK1, KIAA1549, EIF1AX, STAG2, ARID2, TCF12, TMPRSS2, CTNNB1, ELF3, KDM5A, ALDH2, HSP90AA1, USP8, COL1A1, CTNNA2, PBRM1, TET2, CDH10, SGK1, KIAA1549, EIF1AX, STAG2, ARID2, TCF12, TMPRSS2, CTNNB1, ELF3, KDM5A, ALDH2, HSP90AA1, USP8, COL1A1, CTNNA2, PBRM1, TET2, CDH10, SGK1, KIAA1549, EIF1AX, STAG2, ARID2, TCF12, TMPRSS2, CTNNB1, ELF3, KDM5A, ALDH2, HSP90AA1, USP8, COL1A1, CTNNA2, PBRM1, TET2, CDH10, SGK1, KIAA1549, EIF1AX, STAG2, ARID2, TCF12, TMPRSS2, CTNNB1 | g.chr1:201980061G>C, g.chr12:416863G>C, g.chr12:112247443A>G, g.chr14:102553387G>A, g.chr15:50782477T>G, g.chr17:48269167G>A, g.chr2:80875715C>A, g.chr3:52643785A>C, g.chr4:106156783C>G, g.chr5:24593514G>A, g.chr6:134495973T>C, g.chr7:138522886T>G, g.chrX:20153865C>G, g.chrX:123095615C>T, g.chr12:46301322_46301349delATGGCTTCCCACTTAGGTTTTTCTTCTT, g.chr15:57565390_57565391insC, g.chr21:42843875_42843876insAATGCCGTCC, g.chr3:41266070_41266141delAGTCACTGGCAGCAACAGTCTTACCTGGACTCTGGAATCCATTCTGGTGCCACTACCACAGCTCCTTCTCTG, g.chr1:201980061G>C, g.chr12:416863G>C, g.chr12:112247443A>G, g.chr14:102553387G>A, g.chr15:50782477T>G, g.chr17:48269167G>A, g.chr2:80875715C>A, g.chr3:52643785A>C, g.chr4:106156783C>G, g.chr5:24593514G>A, g.chr6:134495973T>C, g.chr7:138522886T>G, g.chrX:20153865C>G, g.chrX:123095615C>T, g.chr12:46301322_46301349delATGGCTTCCCACTTAGGTTTTTCTTCTT, g.chr15:57565390_57565391insC, g.chr21:42843875_42843876insAATGCCGTCC, g.chr3:41266070_41266141delAGTCACTGGCAGCAACAGTCTTACCTGGACTCTGGAATCCATTCTGGTGCCACTACCACAGCTCCTTCTCTG, g.chr1:201980061G>C, g.chr12:416863G>C, g.chr12:112247443A>G, g.chr14:102553387G>A, g.chr15:50782477T>G, g.chr17:48269167G>A, g.chr2:80875715C>A, g.chr3:52643785A>C, g.chr4:106156783C>G, g.chr5:24593514G>A, g.chr6:134495973T>C, g.chr7:138522886T>G, g.chrX:20153865C>G, g.chrX:123095615C>T, g.chr12:46301322_46301349delATGGCTTCCCACTTAGGTTTTTCTTCTT, g.chr15:57565390_57565391insC, g.chr21:42843875_42843876insAATGCCGTCC, g.chr3:41266070_41266141delAGTCACTGGCAGCAACAGTCTTACCTGGACTCTGGAATCCATTCTGGTGCCACTACCACAGCTCCTTCTCTG, g.chr1:201980061G>C, g.chr12:416863G>C, g.chr12:112247443A>G, g.chr14:102553387G>A, g.chr15:50782477T>G, g.chr17:48269167G>A, g.chr2:80875715C>A, g.chr3:52643785A>C, g.chr4:106156783C>G, g.chr5:24593514G>A, g.chr6:134495973T>C, g.chr7:138522886T>G, g.chrX:20153865C>G, g.chrX:123095615C>T, g.chr12:46301322_46301349delATGGCTTCCCACTTAGGTTTTTCTTCTT, g.chr15:57565390_57565391insC, g.chr21:42843875_42843876insAATGCCGTCC, g.chr3:41266070_41266141delAGTCACTGGCAGCAACAGTCTTACCTGGACTCTGGAATCCATTCTGGTGCCACTACCACAGCTCCTTCTCTG, g.chr1:201980061G>C, g.chr12:416863G>C, g.chr12:112247443A>G, g.chr14:102553387G>A, g.chr15:50782477T>G, g.chr17:48269167G>A, g.chr2:80875715C>A, g.chr3:52643785A>C, g.chr4:106156783C>G, g.chr5:24593514G>A, g.chr6:134495973T>C, g.chr7:138522886T>G, g.chrX:20153865C>G, g.chrX:123095615C>T, g.chr12:46301322_46301349delATGGCTTCCCACTTAGGTTTTTCTTCTT, g.chr15:57565390_57565391insC, g.chr21:42843875_42843876insAATGCCGTCC, g.chr3:41266070_41266141delAGTCACTGGCAGCAACAGTCTTACCTGGACTCTGGAATCCATTCTGGTGCCACTACCACAGCTCCTTCTCTG | Prost |
| DO51992 | ERBB4 | g.chr2:212288967G>A | Lymph |
| DO52124 | TP53 | g.chr17:7577538C>T | Panc |
| DO52129 | DAXX | g.chr6:33288561C>A | Panc |
| DO52131 | APC | g.chr5:112102920A>G | Panc |
| DO52150 | SETD2 | g.chr3:47088090G>A | Panc |
| DO52161 | TP53 | g.chr17:7577099C>T | Panc |
| DO52506 | TP53 | g.chr17:7577548C>T | Prost |
| DO52509 | MED12, MED12 | g.chrX:70349888T>G, g.chrX:70349888T>G | Prost |
| DO52510 | KCNJ5, PTK6, IKZF1, KCNJ5, PTK6, IKZF1, KCNJ5, PTK6, IKZF1, KCNJ5, PTK6, IKZF1, KCNJ5, KCNJ5, PTK6, IKZF1, PTK6, IKZF1, KCNJ5, ABCA3, AC016907.3, AC079610.1, AC091320.2, AC091320.2, AC092048.1, ADAMTS9-AS2, ATP2C1, BDNF-AS, C19orf35, CTC-297N7.11, CTD-2014E2.5, CTD-2307P3.1, CTD-3006G17.2, F11-AS1, FARSB, FCRL3, FNDC3B, FNDC3B, IFNG-AS1, INHBA, KIAA0513, KIF9-AS1, LL09NC01-254D11.1, MEF2C-AS1, MIR518D, MIR526B, NREP-AS1, OR14A2, PMS2CL, RASA2, RBFOX2, RN7SL575P, RN7SL659P, RNA5SP36, RNU4-65P, RP1-91G5.3, RP11-167N24.3, RP11-17E2.2, RP11-32K4.1, RP11-32K4.1, RP11-431M7.2, RP11-446J8.1, RP11-550I24.2, RP11-586K2.1, RP11-586K2.1, RP11-624L4.1, RP11-644F5.16, RP11-657O9.1, RP11-708B6.2, RP4-735C1.4, RP4-777D9.2, RPA3-AS1, SERPINB9, SLC15A1, SP100, STARD4-AS1, TPT1-AS1, TRAPPC3, TRBV6-5, TSSC2, Y_RNA, ZNF37BP | g.chr11:128788343G>A, g.chr20:62160809C>T, g.chr7:50470493G>A, g.chr11:128786647_128786658delCCTCCTGTCACT, g.chr20:62160809C>T, g.chr7:50470493G>A, g.chr11:128786647_128786658delCCTCCTGTCACT, g.chr20:62160809C>T, g.chr7:50470493G>A, g.chr11:128788343G>A, g.chr20:62160809C>T, g.chr7:50470493G>A, g.chr11:128786647_128786658delCCTCCTGTCACT, g.chr11:128788343G>A, g.chr20:62160809C>T, g.chr7:50470493G>A, g.chr20:62160809C>T, g.chr7:50470493G>A, g.chr11:128786647_128786658delCCTCCTGTCACT, g.chr16:2358525T>G, g.chr2:30272740A>C, g.chr2:213463940A>G, g.chr7:119126573T>C, g.chr7:119126876G>T, g.chr3:42465926T>C, g.chr3:64785780_64785781delAC, g.chr3:130569755C>T, g.chr11:27616645C>A, g.chr19:2278606C>T, g.chr17:10383864C>A, g.chr16:31574467G>T, g.chr14:42837455G>A, g.chr14:27999705G>C, g.chr4:187323597C>A, g.chr2:223435633A>G, g.chr1:157660311C>T, g.chr3:172117667A>G, g.chr3:172117700A>G, g.chr12:68572975G>A, g.chr7:41729033A>G, g.chr16:85124650C>G, g.chr3:47237037C>T, g.chr9:136478485C>T, g.chr5:88479757G>A, g.chr19:54237816G>A, g.chr19:54199867_54199868insT, g.chr5:111350394_111350395insT, g.chr1:247886625G>C, g.chr7:6781938G>A, g.chr3:141295924delT, g.chr22:36152190C>T, g.chr2:95670963G>C, g.chr9:110573934G>T, g.chr13:95301619C>T, g.chr12:70835034G>A, g.chr1:152350432_152350434delTGA, g.chr12:95824518T>C, g.chr4:22132903C>T, g.chr8:64897092G>A, g.chr8:65243554A>G, g.chr4:36376786G>A, g.chr4:16474229G>C, g.chr3:156911284G>A, g.chr8:89526171C>A, g.chr8:89747950C>T, g.chr15:39652672delC, g.chr12:56042105C>T, g.chr3:135095756T>C, g.chr11:109451957C>T, g.chr1:110268431G>A, g.chr20:21143053C>T, g.chr7:7917532G>C, g.chr6:2895638T>A, g.chr13:99336363T>G, g.chr2:231406053C>T, g.chr5:110997495C>T, g.chr13:45923581C>G, g.chr1:36603566A>C, g.chr7:142178803A>G, g.chr11:3418241A>G, g.chr4:159986809A>G, g.chr10:43007904G>A | Prost |
| DO52511 | AFF1, AFF1 | g.chr4:88061259A>G, g.chr4:88061259A>G | Prost |
| DO52512 | WNK2, WNK2, EXT2, KCNJ5, STAT6, ROBO2, CDH10, WNK2, EXT2, STAT6, BCL11A, ROBO2, CDH10, BIRC3, WNK2, KCNJ5, BCL11A, ROBO2, MAP3K13, CDH10, BIRC3, WNK2, MAP3K13, WNK2, AC027612.2, AC079305.8, AC110086.1, CTD-2021J15.1, CTSS, FAM154A, FZD1, KIF1A, NHLH1, P2RY2, PNMA1, RP11-420N3.2, RP11-508N22.8, RP13-578N3.3, SNORD2, SPANXA2-OT1, ZCCHC4 | g.chr9:96069089_96069091delAAG, g.chr9:96069089_96069091delAAG, g.chr11:44117180C>A, g.chr11:128788574C>A, g.chr12:57505001G>A, g.chr3:77612435C>A, g.chr5:24488095C>T, g.chr9:96069089_96069091delAAG, g.chr11:44117180C>A, g.chr12:57505001G>A, g.chr2:60685879G>A, g.chr3:77612435C>A, g.chr5:24488095C>T, g.chr11:102192546_102192574delCTTTTTGTTTGTTTTTGAACAGGTTTACA, g.chr9:96069089_96069091delAAG, g.chr11:128788574C>A, g.chr2:60685879G>A, g.chr3:77612435C>A, g.chr3:185184677C>T, g.chr5:24488095C>T, g.chr11:102192546_102192574delCTTTTTGTTTGTTTTTGAACAGGTTTACA, g.chr9:96069089_96069091delAAG, g.chr3:185184677C>T, g.chr9:96069089_96069091delAAG, g.chr2:91954620G>C, g.chr2:178073684G>T, g.chr2:165281855G>A, g.chr3:98733553T>G, g.chr1:150704624C>T, g.chr9:18928403C>T, g.chr7:90894804C>G, g.chr2:241700140C>T, g.chr1:160342624C>T, g.chr11:72947199A>T, g.chr14:74180087C>G, g.chr16:5810541G>A, g.chr10:38463026G>C, g.chr4:144696182G>A, g.chr10:58353686C>T, g.chrX:140604005C>T, g.chr4:25363480G>A | Prost |
| DO52513 | ZNF384, ELL, PTPRT, ZNF384, ELL, PTPRT, ZNF384, ELL, ZNF384, ELL | g.chr12:6776420G>C, g.chr19:18576695C>T, g.chr20:40757401T>C, g.chr12:6776420G>C, g.chr19:18576695C>T, g.chr20:40757401T>C, g.chr12:6776420G>C, g.chr19:18576695C>T, g.chr12:6776420G>C, g.chr19:18576695C>T | Prost |
| DO52516 | TP53 | g.chr17:7577539G>A | Prost |
| DO52538 | TP53 | g.chr17:7578394T>C | Breast |
| DO52543 | H3F3A, TP53 | g.chr1:226253357G>A, g.chr17:7578263G>A | Breast |
| DO52544 | RB1 | g.chr13:49037866G>C | Breast |
| DO52545 | TP53 | g.chr17:7577094G>A | Breast |
| DO52546 | TP53 | g.chr17:7578212G>A | Breast |
| DO52547 | TP53 | g.chr17:7577153C>A | Breast |
| DO52549 | TP53 | g.chr17:7578263G>A | Breast |
| DO52550 | TP53 | g.chr17:7578406C>T | Breast |
| DO52553 | TP53 | g.chr17:7578271T>C | Breast |
| DO52555 | TP53 | g.chr17:7578271T>C | Breast |
| DO52556 | TP53 | g.chr17:7577141C>T | Breast |
| DO52557 | TP53 | g.chr17:7578176C>A | Breast |
| DO52558 | PTEN, RB1, PIK3CA | g.chr10:89717708C>T, g.chr13:49037866G>C, g.chr3:178952085A>G | Breast |
| DO52561 | TP53, DCAF12L2 | g.chr17:7578508C>T, g.chrX:125298905G>A | Breast |
| DO52564 | TP53 | g.chr17:7577124C>T | Bone |
| DO52574 | TP53 | g.chr17:7578526C>T | Bone |
| DO52575 | H3F3A | g.chr1:226252155G>T | Bone |
| DO52576 | TP53 | g.chr17:7577539G>A | Bone |
| DO52582 | TP53 | g.chr17:7577111G>C | Bone |
| DO52591 | PTEN | g.chr10:89720749delC | Bone |
| DO52594 | TP53 | g.chr17:7577568C>A | Bone |
| DO52597 | TP53, CD209 | g.chr17:7577114C>T, g.chr19:7806334C>T | Bone |
| DO52605 | TP53, TP53 | g.chr17:7574018G>A, g.chr17:7577120C>A | Bone |
| DO52610 | TP53 | g.chr17:7577120C>A | Bone |
| DO52623 | H3F3B | g.chr17:73775146T>A | Bone |
| DO52625 | H3F3B | g.chr17:73775146T>A | Bone |
| DO52629 | H3F3B | g.chr17:73775146T>A | Bone |
| DO52631 | H3F3B | g.chr17:73775146T>A | Bone |
| DO52633 | H3F3A | g.chr1:226252155G>T | Bone |
| DO52647 | BCL2, BCL2, BCL2, BCL2, BCL2, BCL2, BCL2, BCL2, BCL2, PIM1 | g.chr18:60985492C>T, g.chr18:60985974G>A, g.chr18:60986053C>T, g.chr18:60986148G>A, g.chr18:60986377T>G, g.chr18:60986386T>C, g.chr18:60986516C>G, g.chr18:60986744G>A, g.chr18:60986980C>T, g.chr6:37138901C>T | Lymph |
| DO52648 | SOCS1, SOCS1 | g.chr16:11348738G>A, g.chr16:11348808C>G | Lymph |
| DO52649 | MYD88 | g.chr3:38182032C>G | Lymph |
| DO52650 | BCL2, BCL2, BCL2, BCL2, BCL2, BCL2, BCL2, BCL6, PIM1, SGK1, SGK1, SGK1, IGKJ5 | g.chr18:60985760C>T, g.chr18:60985900C>T, g.chr18:60985982G>A, g.chr18:60985994C>T, g.chr18:60986189A>G, g.chr18:60986205A>G, g.chr18:60986697T>C, g.chr3:187463261A>T, g.chr6:37139210C>T, g.chr6:134495648C>T, g.chr6:134495663G>C, g.chr6:134495673G>A, g.chr2:89160260delT | Lymph |
| DO52651 | CD79B, BCL2, BCL2, MYD88, PIM1, PIM1, PIM1 | g.chr17:62006798T>C, g.chr18:60986420G>A, g.chr18:60986652C>T, g.chr3:38182641T>C, g.chr6:37138355C>T, g.chr6:37138937C>T, g.chr6:37138950G>A | Lymph |
| DO52652 | BCL2, BCL2, BCL2, BCL2, BCL2, BCL2, BCL2, CARD11, EZH2, B2M | g.chr18:60985916G>A, g.chr18:60986279T>G, g.chr18:60986299T>A, g.chr18:60986322T>G, g.chr18:60986793C>T, g.chr18:60986935G>C, g.chr18:60987060T>A, g.chr7:2979559C>T, g.chr7:148508728A>T, g.chr15:45003781_45003782delCT | Lymph |
| DO52653 | BCL2, MYD88, EZH2 | g.chr18:60986305G>A, g.chr3:38182032C>G, g.chr7:148508727T>A | Lymph |
| DO52654 | BCL2, BCL2, BCL2, BCL2, BCL2, BCL2 | g.chr18:60985508G>A, g.chr18:60985877C>T, g.chr18:60986100T>C, g.chr18:60986651G>A, g.chr18:60986888C>G, g.chr18:60987040C>T | Lymph |
| DO52655 | B2M, BCL2, BCL2, BCL2, BCL2, BCL2, BCL2, BCL2, BCL2, EZH2, TMSB4X | g.chr15:45003764T>G, g.chr18:60985993G>C, g.chr18:60986234T>A, g.chr18:60986278G>A, g.chr18:60986322T>G, g.chr18:60986407T>C, g.chr18:60986630T>C, g.chr18:60986750T>G, g.chr18:60986793C>T, g.chr7:148508728A>T, g.chrX:12993589G>A | Lymph |
| DO52656 | STAT6, BCL2 | g.chr12:57498345C>T, g.chr18:60985905C>T | Lymph |
| DO52657 | TMSB4X | g.chrX:12994364G>C | Lymph |
| DO52659 | PIM1, MYC | g.chr6:37139063G>A, g.chr8:128751036G>A | Lymph |
| DO52661 | SOCS1, MUC16, MYC, MYC, MYC | g.chr16:11348962C>G, g.chr19:9056233C>T, g.chr8:128748551G>A, g.chr8:128748697G>A, g.chr8:128748863A>G | Lymph |
| DO52662 | CREBBP, BCL2, BCL2, BCL2, PIM1 | g.chr16:3788618G>A, g.chr18:60986152G>A, g.chr18:60986409G>A, g.chr18:60986773G>A, g.chr6:37138901C>T | Lymph |
| DO52663 | SOCS1, BCL2, BCL2, BCL2, BCL2, BCL2, BCL2, BCL2, BCL6, TMSB4X, B2M | g.chr16:11348907G>A, g.chr18:60985900C>T, g.chr18:60986018T>G, g.chr18:60986163A>G, g.chr18:60986305G>A, g.chr18:60986409G>A, g.chr18:60986444T>C, g.chr18:60986509G>A, g.chr3:187463212T>C, g.chrX:12993709C>G, g.chr15:45003781_45003782delCT | Lymph |
| DO52664 | STAT6, HMGN2P46, ZFHX3, TP53, BCL2, BCL2, BCL2, BCL2, BCL2, BCL2, BCL2, BCL2, BCL2, SGK1, FAM135B, TMSB4X | g.chr12:57496671C>G, g.chr15:45843418C>T, g.chr16:72828405G>C, g.chr17:7577097C>T, g.chr18:60985834C>T, g.chr18:60985916G>A, g.chr18:60985982G>A, g.chr18:60986053C>T, g.chr18:60986100T>C, g.chr18:60986278G>A, g.chr18:60986321A>G, g.chr18:60986652C>T, g.chr18:60986744G>A, g.chr6:134495724G>A, g.chr8:139165111G>T, g.chrX:12994443_12994444delAG | Lymph |
| DO52665 | MYD88 | g.chr3:38182641T>C | Lymph |
| DO52666 | PIM1, TMSB4X | g.chr6:37139146G>A, g.chrX:12993709C>G | Lymph |
| DO52667 | STAT6, BCL2, BCL2, BCL2, BCL2, BCL2, BCL2, BCL2, BCL2, BCL2, BCL2 | g.chr12:57496671C>G, g.chr18:60985562G>C, g.chr18:60985917C>T, g.chr18:60986069T>C, g.chr18:60986195T>A, g.chr18:60986323_60986324TT>GC, g.chr18:60986377T>G, g.chr18:60986509G>A, g.chr18:60986805T>C, g.chr18:60986960C>G, g.chr18:60986987T>A | Lymph |
| DO52668 | CCND3 | g.chr6:41903745_41903746insG | Lymph |
| DO52669 | BCL2, BCL2, BCL2, BCL2, BCL2, BCL2 | g.chr18:60985435C>T, g.chr18:60985444C>T, g.chr18:60986278G>C, g.chr18:60986420G>A, g.chr18:60986515G>A, g.chr18:60986651G>A | Lymph |
| DO52670 | MYD88, PIM1, PIM1, TMSB4X | g.chr3:38182641T>C, g.chr6:37138402C>T, g.chr6:37138423G>A, g.chrX:12993674C>T | Lymph |
| DO52671 | FOXO1, BCL2, BCL2, BCL2, BCL2, EZH2, ELK4 | g.chr13:41240288C>T, g.chr18:60985993G>C, g.chr18:60986331A>C, g.chr18:60986750T>G, g.chr18:60986935G>C, g.chr7:148508728A>T, g.chr1:205582792delC | Lymph |
| DO52672 | B2M, PIM1, CDKN2A, TMSB4X, B2M | g.chr15:45003764T>G, g.chr6:37138423G>C, g.chr9:21971208C>T, g.chrX:12993729T>C, g.chr15:45003781_45003784delCTCT | Lymph |
| DO52674 | NRAS, FOXO1, LRP1B, HLA-A, SGK1, TMSB4X, TMSB4X | g.chr1:115256528T>G, g.chr13:41240288C>T, g.chr2:141643809T>C, g.chr6:29911311C>T, g.chr6:134495654G>A, g.chrX:12993423G>A, g.chrX:12993848C>T | Lymph |
| DO52675 | BTG2, BTG1, SOCS1, SOCS1, ROBO2, EBF1, HLA-A, PIM1, TMSB4X, VTI1A | g.chr1:203276414G>A, g.chr12:92539204C>T, g.chr16:11348738G>A, g.chr16:11348808C>G, g.chr3:77147279C>T, g.chr5:158526445G>A, g.chr6:29911311C>T, g.chr6:37139063G>A, g.chrX:12993999G>A, g.chr10:114578212_114578213insT | Lymph |
| DO52677 | TP53, BCL2, BCL2, BCL2, BCL2, BCL2, BCL2, BCL2, BCL2, BCL2 | g.chr17:7577121G>A, g.chr18:60985877C>T, g.chr18:60985900C>T, g.chr18:60986069T>C, g.chr18:60986153T>G, g.chr18:60986243A>C, g.chr18:60986407T>C, g.chr18:60986420G>C, g.chr18:60986515G>A, g.chr18:60986917G>A | Lymph |
| DO52679 | BTG1, FOXO1, SOCS1, XPO1, TMSB4X, TMSB4X, TMSB4X, TMSB4X, TMSB4X | g.chr12:92539204C>T, g.chr13:41130185T>A, g.chr16:11348907G>A, g.chr2:61719472C>T, g.chrX:12993410G>A, g.chrX:12993554C>T, g.chrX:12993655C>T, g.chrX:12993735C>T, g.chrX:12993787G>C | Lymph |
| DO52681 | CREBBP, BCL2, BCL2, BCL2, BCL2, BCL2, BCL2, TMSB4X | g.chr16:3788618G>A, g.chr18:60985994C>T, g.chr18:60986300A>T, g.chr18:60986409G>A, g.chr18:60986423C>T, g.chr18:60986878T>C, g.chr18:60986980C>T, g.chrX:12993423G>A | Lymph |
| DO52682 | BCR, TMSB4X | g.chr22:23523353G>C, g.chrX:12993735C>T | Lymph |
| DO52683 | CCND3 | g.chr6:41903745_41903746insG | Lymph |
| DO52684 | SGK1, SGK1, P2RY8, TMSB4X, TMSB4X | g.chr6:134495648C>T, g.chr6:134495663G>C, g.chrX:1584907G>A, g.chrX:12993709C>G, g.chrX:12993910C>T | Lymph |
| DO52685 | BCL2, BCL2, BCL2, BCL2, BCL2, BCL2, BCL2, BCL2, BCL2, PIM1, PIM1, PIM1, EZH2, RALGDS, BCL11B | g.chr18:60985435C>T, g.chr18:60985834C>T, g.chr18:60985916G>A, g.chr18:60986144A>T, g.chr18:60986154A>G, g.chr18:60986163A>G, g.chr18:60986425G>A, g.chr18:60986516C>G, g.chr18:60986744G>A, g.chr6:37138950G>A, g.chr6:37139078C>T, g.chr6:37139236G>A, g.chr7:148508728A>T, g.chr9:135984123G>A, g.chr14:99638831delA | Lymph |
| DO52686 | PIM1, PIM1, PIM1, SGK1, SGK1, PAX5, TMSB4X, TMSB4X, TMSB4X, TMSB4X, TMSB4X, TMSB4X, CDH11 | g.chr6:37138423G>C, g.chr6:37139078C>T, g.chr6:37139210C>T, g.chr6:134495663G>C, g.chr6:134495724G>A, g.chr9:36834661C>T, g.chrX:12993554C>T, g.chrX:12993674C>T, g.chrX:12993677C>T, g.chrX:12993758C>G, g.chrX:12993825G>A, g.chrX:12994220C>T, g.chr16:64980684_64980685insA | Lymph |
| DO52687 | BCL2, TMSB4X, CDH10 | g.chr18:60986420G>C, g.chrX:12993825G>A, g.chr5:24487658_24487659insA | Lymph |
| DO52688 | BCL2, BCL2, EZH2 | g.chr18:60986252G>A, g.chr18:60986300A>T, g.chr7:148508728A>G | Lymph |
| DO52689 | BCL2, BCL2, BCL2, BCL2, BCL2, CARD11 | g.chr18:60985834C>T, g.chr18:60985917C>T, g.chr18:60986018T>G, g.chr18:60986082T>G, g.chr18:60986234T>A, g.chr7:2977614T>A | Lymph |
| DO52690 | CREBBP, BCL2, BCL2, BCL2, BCL2, BCL2, BCL2, BCL2, BCL2, LRP1B | g.chr16:3786704A>C, g.chr18:60985916G>C, g.chr18:60986144A>T, g.chr18:60986153T>G, g.chr18:60986299T>A, g.chr18:60986425G>A, g.chr18:60986652C>T, g.chr18:60986878T>C, g.chr18:60986888C>T, g.chr2:141027892G>C | Lymph |
| DO52691 | NRAS, FBXO11, FOXL2, MYC, MYC | g.chr1:115258747C>T, g.chr2:48040518T>C, g.chr3:138663667A>T, g.chr8:128748697G>A, g.chr8:128748832C>T | Lymph |
| DO52692 | BTG2, SOCS1, PIM1, PIM1, PIM1, PIM1, PIM1, PIM1, SGK1, SGK1, MYC, TMSB4X, TMSB4X, TMSB4X, TMSB4X, TMSB4X, TMSB4X, TMSB4X, TMSB4X, IGKJ3 | g.chr1:203274876G>A, g.chr16:11348962C>G, g.chr6:37138402C>T, g.chr6:37138423G>A, g.chr6:37139077G>A, g.chr6:37139146G>A, g.chr6:37139210C>T, g.chr6:37139236G>A, g.chr6:134495673G>A, g.chr6:134495724G>A, g.chr8:128750953C>G, g.chrX:12993589G>A, g.chrX:12993637C>T, g.chrX:12993677C>T, g.chrX:12993729T>C, g.chrX:12993735C>T, g.chrX:12993787G>C, g.chrX:12993848C>T, g.chrX:12994220C>T, g.chr2:89163232_89163252delCTTTTTTAAAGGGGACACAAA | Lymph |
| DO52693 | CREBBP, BCL2, BCL2, BCL2, BCL2, BCL2, CARD11, AKAP11 | g.chr16:3786703T>A, g.chr18:60985549G>A, g.chr18:60985916G>A, g.chr18:60985997T>C, g.chr18:60986163A>G, g.chr18:60986630T>C, g.chr7:2977614T>A, g.chr13:42877802_42877826delTCATGTTAATCTTGATAAGAAGGCA | Lymph |
| DO52694 | CREBBP, BCL2, BCL2, BCL2, BCL2, BCL2, BCL2, BCL2, BCL2, BCL2, BCL2, BCL2, BCL2 | g.chr16:3786748G>A, g.chr18:60985549G>A, g.chr18:60985876C>T, g.chr18:60985982G>A, g.chr18:60986069T>C, g.chr18:60986138G>A, g.chr18:60986252G>A, g.chr18:60986280A>G, g.chr18:60986423C>T, g.chr18:60986651G>C, g.chr18:60986935G>C, g.chr18:60986960C>G, g.chr18:60986987T>A | Lymph |
| DO52695 | CREBBP, BCL2, BCL2, BCL2, BCL2, BCL2, BCL2 | g.chr16:3786704A>G, g.chr18:60985508G>A, g.chr18:60985793C>T, g.chr18:60985834C>T, g.chr18:60986321A>G, g.chr18:60986515G>A, g.chr18:60986652C>T | Lymph |
| DO52696 | TP53 | g.chr17:7577560A>G | Lymph |
| DO52698 | NOTCH1 | g.chr9:139390152T>C | Lymph |
| DO52699 | TFEB | g.chr6:41655684G>A | Lymph |
| DO52703 | NFKBIE | g.chr6:44232739_44232742delGTAA | Lymph |
| DO52704 | BCL2, WWTR1 | g.chr18:60987040C>T, g.chr3:149237163delT | Lymph |
| DO52706 | XPO1 | g.chr2:61719472C>T | Lymph |
| DO52708 | SF3B1 | g.chr2:198266834T>C | Lymph |
| DO52710 | TP53 | g.chr17:7578190T>C | Lymph |
| DO52712 | NOTCH1 | g.chr9:139390649_139390650delAG | Lymph |
| DO52717 | TP53 | g.chr17:7577568C>A | Lymph |
| DO52718 | BCL2, BCL2, BCL2, TMSB4X | g.chr18:60985444C>T, g.chr18:60986906T>G, g.chr18:60987060T>A, g.chrX:12993655C>T | Lymph |
| DO52724 | EED | g.chr11:85956145_85956146insG | Lymph |
| DO52729 | BTG2, BTG2 | g.chr1:203274758C>T, g.chr1:203274758C>T | Myeloid |
| DO52732 | MITF, MITF | g.chr3:70014685G>A, g.chr3:70014685G>A | Myeloid |
| DO52733 | EPAS1, KDR, EPAS1, KDR | g.chr2:46613545G>T, g.chr4:55945722C>T, g.chr2:46613545G>T, g.chr4:55945722C>T | Myeloid |
| DO52736 | NOTCH2, SH2B3, TET2, NOTCH2, SH2B3, TET2, NOTCH2, SH2B3, TET2, AC004485.3, AC004862.6, AC097467.2, CRYM, CTB-57H20.1, CTD-2194D22.1, DNAJB7, GS1-256O22.5, HELQ, KCND1, LMCD1-AS1, MCHR2-AS1, MIR378D1, OTX2-AS1, PPP1R26-AS1, PROM2, PUM1, RAET1K, RFESD, RP11-154D6.1, RP11-184A2.3, RP11-310I9.1, RP11-32K4.1, RP11-492A10.1, RP11-631F7.1, RP11-731J8.2, RP4-756H11.3, RP5-921G16.1, SERPINB11, SGK223, SNORA31, SPTBN4, SULT1C2P1, SYT4, TG, TMEM132B, TNRC18, TPTE2P1, TRPC5, VWA3B, ZBTB7C, ZCCHC11, ZNF20, ZNF222, snoU13 | g.chr1:120462033G>A, g.chr12:111885596T>C, g.chr4:106164860A>G, g.chr1:120462033G>A, g.chr12:111885596T>C, g.chr4:106164860A>G, g.chr1:120462033G>A, g.chr12:111885596T>C, g.chr4:106164860A>G, g.chr7:24293280A>C, g.chr7:79973684C>T, g.chr4:156255744G>A, g.chr16:21289546G>T, g.chr5:143031632C>T, g.chr5:1852436C>G, g.chr22:41255694A>G, g.chrX:142562513G>A, g.chr4:84352921C>T, g.chrX:48827362G>A, g.chr3:8292118A>G, g.chr6:100457158G>A, g.chr4:5926633G>A, g.chr14:57590110G>A, g.chr9:138366942A>C, g.chr2:95950840C>T, g.chr1:31405551T>C, g.chr6:150327833C>T, g.chr5:94991892G>A, g.chr6:71951796G>A, g.chr10:3796957G>A, g.chr4:168990656C>T, g.chr8:65277637T>C, g.chr5:113949187G>A, g.chr6:148564361C>A, g.chr4:47440513T>C, g.chr7:66124697T>A, g.chr7:123809383A>C, g.chr18:61381918T>C, g.chr8:8234772C>T, g.chr10:78593831C>A, g.chr19:41073804G>A, g.chr2:108956360C>T, g.chr18:40850213C>T, g.chr8:133895139G>C, g.chr12:126145649G>C, g.chr7:5355647C>G, g.chr13:25529964T>C, g.chrX:111018133C>G, g.chr2:98828414C>A, g.chr18:45555354G>A, g.chr1:52981650A>T, g.chr19:12246374_12246375insC, g.chr19:44536240T>A, g.chr9:78290522C>A | Myeloid |
| DO52737 | AC093391.2, AL626787.1, AL672294.1, ALOX12P2, ARHGAP40, CACNA1C, CMAHP, COPB1, CTD-2058B24.2, CYLC2, DZIP1, FSIP2, ISM2, LINC00535, PPP1R12C, RNA5SP459, RP11-152L20.3, RP11-155G15.2, RP11-155G15.2, RP11-3B12.1, RP11-420N3.2, RP11-81K13.1, RP11-944L7.4, SNORD81, SUPT6H, TM4SF1-AS1, Y_RNA | g.chr2:136777710G>A, g.chr1:166027962G>T, g.chr1:249156513T>C, g.chr17:6792670G>A, g.chr20:37270390G>A, g.chr12:2717876C>G, g.chr6:25082700T>C, g.chr11:14521315C>A, g.chr14:38633395G>C, g.chr9:105767103C>T, g.chr13:96242650T>C, g.chr2:186665453G>C, g.chr14:77941705A>T, g.chr8:94341154C>T, g.chr19:55624914G>A, g.chr18:52813088C>A, g.chr15:92824837C>T, g.chr5:96654575G>A, g.chr5:96734949G>A, g.chr7:124626317G>T, g.chr16:5911956G>A, g.chr12:75413892C>T, g.chr3:44654842C>T, g.chr12:54183107C>T, g.chr17:27002476G>A, g.chr3:149103273A>G, g.chr20:42079004T>G | Myeloid |
| DO52738 | KLF6, RBM10, KLF6, TP53, RBM10 | g.chr10:3819915C>G, g.chrX:47044469G>T, g.chr10:3819915C>G, g.chr17:7577535C>A, g.chrX:47044469G>T | Myeloid |
| DO52739 | ASXL1, MACC1, SETBP1, ASXL1, MACC1 | g.chr20:31024339C>G, g.chr7:20179646T>C, g.chr18:42531907G>A, g.chr20:31024339C>G, g.chr7:20179646T>C | Myeloid |
| DO52740 | IDH2, FAT1, PHF6, IDH2, FAT1, PHF6 | g.chr15:90631934C>T, g.chr4:187518216G>A, g.chrX:133511742_133511743insA, g.chr15:90631934C>T, g.chr4:187518216G>A, g.chrX:133511742_133511743insA | Myeloid |
| DO52742 | ROS1, ROS1 | g.chr6:117714406C>T, g.chr6:117714406C>T | Myeloid |
| DO52743 | CTCF, CTCF, CTCF, AC004791.2, AC009236.1, AC072062.1, AC108142.1, AC136188.1, AC136932.2, AC144450.1, CTB-57H20.1, CTSL3P, F11-AS1, HLTF-AS1, HTR3B, IGHM, NFYB, NLGN4X, NR2F2-AS1, PRKY, RP1-272J12.1, RP11-122D10.1, RP11-135J2.4, RP11-17E2.2, RP11-17E2.2, RP11-482H16.1, RP11-542K23.7, SEC24B-AS1, STEAP2-AS1, TRGC2, UCHL1-AS1, snoU13 | g.chr16:67650710C>T, g.chr16:67650710C>T, g.chr16:67650710C>T, g.chr19:15972616G>A, g.chr2:45424442C>T, g.chr2:215743046G>A, g.chr4:182885907C>T, g.chr12:74291327A>G, g.chr16:33948737G>A, g.chr2:1552466G>A, g.chr5:143130167G>A, g.chr9:90390493C>T, g.chr4:187255606G>A, g.chr3:148811521delT, g.chr11:113816793C>T, g.chr14:106321605G>A, g.chr12:104510885T>C, g.chrX:5809876C>T, g.chr15:96853013G>A, g.chrY:7209728C>T, g.chr22:35409017C>T, g.chr15:37168924T>C, g.chr1:219280717G>A, g.chr4:22048305G>T, g.chr4:22219448C>T, g.chr2:56386929_56386930CC>GA, g.chr9:125163311A>G, g.chr4:110346403A>G, g.chr7:89652831G>A, g.chr7:38278352T>C, g.chr4:41247266C>A, g.chr2:115039649C>T | Myeloid |
| DO52744 | DNMT3A, TET2, DNMT3A, TET2 | g.chr2:25457176G>A, g.chr4:106190802delG, g.chr2:25457176G>A, g.chr4:106190802delG | Myeloid |
| DO52745 | FAT3, FAT3 | g.chr11:92534068G>C, g.chr11:92534068G>C | Myeloid |
| DO52747 | SPEN, IDH2, U2AF1, SPEN, IDH2, U2AF1 | g.chr1:16257858C>T, g.chr15:90631934C>T, g.chr21:44514777T>C, g.chr1:16257858C>T, g.chr15:90631934C>T, g.chr21:44514777T>C | Myeloid |
| DO52751 | ASXL1, EZH2, ASXL1, EZH2 | g.chr20:31022233A>G, g.chr7:148508788C>T, g.chr20:31022233A>G, g.chr7:148508788C>T | Myeloid |
| DO52752 | TP53, PRKAR1A, MECOM, PRKAR1A, MECOM | g.chr17:7577120C>T, g.chr17:66528481C>T, g.chr3:168845749G>T, g.chr17:66528481C>T, g.chr3:168845749G>T | Myeloid |
| DO52756 | MUC16, DNMT3A, MUC16, DNMT3A | g.chr19:9087213A>T, g.chr2:25457242C>T, g.chr19:9087213A>T, g.chr2:25457242C>T | Myeloid |
| DO52758 | MUC4, MUC4 | g.chr3:195498647T>A, g.chr3:195498647T>A | Myeloid |
| DO52760 | NF1, ITK, NF1, ITK | g.chr17:29559200T>G, g.chr5:156670701G>A, g.chr17:29559200T>G, g.chr5:156670701G>A | Myeloid |
| DO52762 | JAK2, JAK2 | g.chr9:5070022_5070027delTCACAA, g.chr9:5070022_5070027delTCACAA | Myeloid |
| DO52763 | SMARCA4, TSC1, SMARCA4, TSC1 | g.chr19:11114054G>A, g.chr9:135768908C>A, g.chr19:11114054G>A, g.chr9:135768908C>A | Myeloid |
| DO6352 | MYD88 | g.chr3:38182641T>C | Lymph |
| DO6360 | SF3B1 | g.chr2:198266834T>C | Lymph |
| DO6362 | SF3B1 | g.chr2:198266611C>T | Lymph |
| DO6364 | XPO1 | g.chr2:61719472C>T | Lymph |
| DO6398 | TP53, TP53 | g.chr17:7578526C>A, g.chr17:7579312C>T | Lymph |
| DO6428 | NFKBIE | g.chr6:44232739_44232742delGTAA | Lymph |
| DO6432 | BCL2, BCL2 | g.chr18:60985876C>T, g.chr18:60986154A>G | Lymph |
| DO6434 | NOTCH1 | g.chr9:139390649_139390650delAG | Lymph |
| DO6492 | MYD88 | g.chr3:38182641T>C | Lymph |
| DO6549 | NOTCH1 | g.chr9:139390152T>C | Lymph |
| DO6690 | SF3B1 | g.chr2:198266834T>C | Lymph |
| DO6742 | NOTCH1 | g.chr9:139390649_139390650delAG | Lymph |
| DO6754 | MYD88 | g.chr3:38182641T>C | Lymph |
| DO6934 | SETD2, NOTCH1 | g.chr3:47088090G>A, g.chr9:139390152T>C | Lymph |
| DO7084 | CARD11 | g.chr7:2985459T>C | Lymph |
| DO7112 | BCL2 | g.chr18:60986651G>C | Lymph |
| DO7124 | NOTCH1 | g.chr9:139390649_139390650delAG | Lymph |
| DO7166 | TP53 | g.chr17:7578190T>C | Lymph |

**Supplementary Table 2.** DiWANN network edge list with donor sample IDs as node labels.

| **Edge list** |
| --- |
| ['D0', 'DO1001'] |
| ['D0', 'DO1002'] |
| ['D0', 'DO1010'] |
| ['D0', 'DO1013'] |
| ['D0', 'DO1016'] |
| ['D0', 'DO1017'] |
| ['D0', 'DO10809'] |
| ['D0', 'DO10815'] |
| ['D0', 'DO10821'] |
| ['D0', 'DO10843'] |
| ['D0', 'DO10844'] |
| ['D0', 'DO217800'] |
| ['D0', 'DO217814'] |
| ['D0', 'DO217818'] |
| ['D0', 'DO217836'] |
| ['D0', 'DO217844'] |
| ['D0', 'DO217887'] |
| ['D0', 'DO217896'] |
| ['D0', 'DO217907'] |
| ['D0', 'DO217931'] |
| ['D0', 'DO217987'] |
| ['D0', 'DO218030'] |
| ['D0', 'DO218088'] |
| ['D0', 'DO218121'] |
| ['D0', 'DO218174'] |
| ['D0', 'DO218223'] |
| ['D0', 'DO218269'] |
| ['D0', 'DO218282'] |
| ['D0', 'DO218333'] |
| ['D0', 'DO218411'] |
| ['D0', 'DO218417'] |
| ['D0', 'DO218428'] |
| ['D0', 'DO218440'] |
| ['D0', 'DO218442'] |
| ['D0', 'DO218443'] |
| ['D0', 'DO218491'] |
| ['D0', 'DO218502'] |
| ['D0', 'DO218535'] |
| ['D0', 'DO218547'] |
| ['D0', 'DO218611'] |
| ['D0', 'DO218621'] |
| ['D0', 'DO218651'] |
| ['D0', 'DO218684'] |
| ['D0', 'DO218698'] |
| ['D0', 'DO218709'] |
| ['D0', 'DO218828'] |
| ['D0', 'DO220844'] |
| ['D0', 'DO220845'] |
| ['D0', 'DO220846'] |
| ['D0', 'DO220847'] |
| ['D0', 'DO220859'] |
| ['D0', 'DO220884'] |
| ['D0', 'DO220896'] |
| ['D0', 'DO220904'] |
| ['D0', 'DO221543'] |
| ['D0', 'DO221547'] |
| ['D0', 'DO221548'] |
| ['D0', 'DO23508'] |
| ['D0', 'DO23513'] |
| ['D0', 'DO23514'] |
| ['D0', 'DO23517'] |
| ['D0', 'DO23518'] |
| ['D0', 'DO23525'] |
| ['D0', 'DO23527'] |
| ['D0', 'DO23529'] |
| ['D0', 'DO23534'] |
| ['D0', 'DO23535'] |
| ['D0', 'DO23542'] |
| ['D0', 'DO23545'] |
| ['D0', 'DO23548'] |
| ['D0', 'DO27764'] |
| ['D0', 'DO27765'] |
| ['D0', 'DO27773'] |
| ['D0', 'DO27799'] |
| ['D0', 'DO27807'] |
| ['D0', 'DO27815'] |
| ['D0', 'DO32980'] |
| ['D0', 'DO33032'] |
| ['D0', 'DO33184'] |
| ['D0', 'DO33480'] |
| ['D0', 'DO35222'] |
| ['D0', 'DO35547'] |
| ['D0', 'DO35568'] |
| ['D0', 'DO35704'] |
| ['D0', 'DO35733'] |
| ['D0', 'DO35753'] |
| ['D0', 'DO35937'] |
| ['D0', 'DO35982'] |
| ['D0', 'DO44806'] |
| ['D0', 'DO44832'] |
| ['D0', 'DO45035'] |
| ['D0', 'DO45047'] |
| ['D0', 'DO45049'] |
| ['D0', 'DO45055'] |
| ['D0', 'DO45065'] |
| ['D0', 'DO45079'] |
| ['D0', 'DO45081'] |
| ['D0', 'DO45091'] |
| ['D0', 'DO45093'] |
| ['D0', 'DO45097'] |
| ['D0', 'DO45115'] |
| ['D0', 'DO45117'] |
| ['D0', 'DO45121'] |
| ['D0', 'DO45123'] |
| ['D0', 'DO45127'] |
| ['D0', 'DO45129'] |
| ['D0', 'DO45149'] |
| ['D0', 'DO45153'] |
| ['D0', 'DO45159'] |
| ['D0', 'DO45179'] |
| ['D0', 'DO45183'] |
| ['D0', 'DO45185'] |
| ['D0', 'DO45189'] |
| ['D0', 'DO45201'] |
| ['D0', 'DO45207'] |
| ['D0', 'DO45259'] |
| ['D0', 'DO45265'] |
| ['D0', 'DO45273'] |
| ['D0', 'DO45281'] |
| ['D0', 'DO45293'] |
| ['D0', 'DO45305'] |
| ['D0', 'DO46327'] |
| ['D0', 'DO46331'] |
| ['D0', 'DO46333'] |
| ['D0', 'DO46350'] |
| ['D0', 'DO46358'] |
| ['D0', 'DO46366'] |
| ['D0', 'DO46372'] |
| ['D0', 'DO46380'] |
| ['D0', 'DO46388'] |
| ['D0', 'DO46396'] |
| ['D0', 'DO46404'] |
| ['D0', 'DO46416'] |
| ['D0', 'DO46424'] |
| ['D0', 'DO46448'] |
| ['D0', 'DO46488'] |
| ['D0', 'DO46551'] |
| ['D0', 'DO46568'] |
| ['D0', 'DO46606'] |
| ['D0', 'DO46779'] |
| ['D0', 'DO46792'] |
| ['D0', 'DO46795'] |
| ['D0', 'DO46832'] |
| ['D0', 'DO46877'] |
| ['D0', 'DO46881'] |
| ['D0', 'DO46893'] |
| ['D0', 'DO46897'] |
| ['D0', 'DO46909'] |
| ['D0', 'DO46957'] |
| ['D0', 'DO46980'] |
| ['D0', 'DO47048'] |
| ['D0', 'DO47068'] |
| ['D0', 'DO48541'] |
| ['D0', 'DO48689'] |
| ['D0', 'DO48695'] |
| ['D0', 'DO48715'] |
| ['D0', 'DO48719'] |
| ['D0', 'DO48723'] |
| ['D0', 'DO48727'] |
| ['D0', 'DO48732'] |
| ['D0', 'DO48760'] |
| ['D0', 'DO48891'] |
| ['D0', 'DO48893'] |
| ['D0', 'DO48895'] |
| ['D0', 'DO48900'] |
| ['D0', 'DO48914'] |
| ['D0', 'DO48940'] |
| ['D0', 'DO49076'] |
| ['D0', 'DO49533'] |
| ['D0', 'DO49537'] |
| ['D0', 'DO50311'] |
| ['D0', 'DO50315'] |
| ['D0', 'DO50327'] |
| ['D0', 'DO50330'] |
| ['D0', 'DO50334'] |
| ['D0', 'DO50336'] |
| ['D0', 'DO50364'] |
| ['D0', 'DO50367'] |
| ['D0', 'DO50372'] |
| ['D0', 'DO50389'] |
| ['D0', 'DO50399'] |
| ['D0', 'DO50402'] |
| ['D0', 'DO50419'] |
| ['D0', 'DO50431'] |
| ['D0', 'DO50441'] |
| ['D0', 'DO50443'] |
| ['D0', 'DO50785'] |
| ['D0', 'DO50806'] |
| ['D0', 'DO50820'] |
| ['D0', 'DO50829'] |
| ['D0', 'DO50834'] |
| ['D0', 'DO50857'] |
| ['D0', 'DO51050'] |
| ['D0', 'DO51056'] |
| ['D0', 'DO51061'] |
| ['D0', 'DO51063'] |
| ['D0', 'DO51074'] |
| ['D0', 'DO51078'] |
| ['D0', 'DO51087'] |
| ['D0', 'DO51111'] |
| ['D0', 'DO51126'] |
| ['D0', 'DO51141'] |
| ['D0', 'DO51187'] |
| ['D0', 'DO51502'] |
| ['D0', 'DO51507'] |
| ['D0', 'DO51510'] |
| ['D0', 'DO51521'] |
| ['D0', 'DO52150'] |
| ['D0', 'DO52509'] |
| ['D0', 'DO52511'] |
| ['D0', 'DO52544'] |
| ['D0', 'DO52547'] |
| ['D0', 'DO52549'] |
| ['D0', 'DO52556'] |
| ['D0', 'DO52557'] |
| ['D0', 'DO52575'] |
| ['D0', 'DO52582'] |
| ['D0', 'DO52591'] |
| ['D0', 'DO52623'] |
| ['D0', 'DO52649'] |
| ['D0', 'DO52657'] |
| ['D0', 'DO52665'] |
| ['D0', 'DO52698'] |
| ['D0', 'DO52706'] |
| ['D0', 'DO52729'] |
| ['D0', 'DO52732'] |
| ['D0', 'DO52742'] |
| ['D0', 'DO52745'] |
| ['D0', 'DO52758'] |
| ['D0', 'DO6362'] |
| ['D0', 'DO7084'] |
| ['D0', 'DO7112'] |
| ['DO1001', 'D0'] |
| ['DO1001', 'DO51470'] |
| ['DO1001', 'DO51518'] |
| ['DO1002', 'D0'] |
| ['DO1002', 'DO218075'] |
| ['DO1005', 'DO218282'] |
| ['DO1007', 'DO217934'] |
| ['DO1007', 'DO218506'] |
| ['DO1010', 'D0'] |
| ['DO1010', 'DO33408'] |
| ['DO1010', 'DO34608'] |
| ['DO1010', 'DO45083'] |
| ['DO1010', 'DO46350'] |
| ['DO1010', 'DO51511'] |
| ['DO1013', 'D0'] |
| ['DO1013', 'DO217908'] |
| ['DO1013', 'DO218205'] |
| ['DO1013', 'DO218347'] |
| ['DO1013', 'DO220820'] |
| ['DO1013', 'DO220821'] |
| ['DO1013', 'DO45177'] |
| ['DO1013', 'DO51133'] |
| ['DO1016', 'D0'] |
| ['DO1017', 'D0'] |
| ['DO1017', 'DO218560'] |
| ['DO1076', 'DO217908'] |
| ['DO10809', 'D0'] |
| ['DO10815', 'D0'] |
| ['DO10821', 'D0'] |
| ['DO10840', 'DO218535'] |
| ['DO10841', 'DO50443'] |
| ['DO10843', 'D0'] |
| ['DO10843', 'DO218695'] |
| ['DO10843', 'DO32837'] |
| ['DO10843', 'DO46420'] |
| ['DO10843', 'DO49204'] |
| ['DO10843', 'DO50445'] |
| ['DO10844', 'D0'] |
| ['DO10858', 'DO50402'] |
| ['DO217786', 'DO217931'] |
| ['DO217786', 'DO218174'] |
| ['DO217800', 'D0'] |
| ['DO217814', 'D0'] |
| ['DO217818', 'D0'] |
| ['DO217818', 'DO46398'] |
| ['DO217826', 'DO218478'] |
| ['DO217836', 'D0'] |
| ['DO217844', 'D0'] |
| ['DO217850', 'DO221547'] |
| ['DO217887', 'D0'] |
| ['DO217887', 'DO48915'] |
| ['DO217896', 'D0'] |
| ['DO217896', 'DO50306'] |
| ['DO217907', 'D0'] |
| ['DO217907', 'DO52591'] |
| ['DO217908', 'DO1013'] |
| ['DO217908', 'DO1076'] |
| ['DO217931', 'D0'] |
| ['DO217931', 'DO217786'] |
| ['DO217931', 'DO218560'] |
| ['DO217931', 'DO221544'] |
| ['DO217931', 'DO35216'] |
| ['DO217931', 'DO49439'] |
| ['DO217931', 'DO50309'] |
| ['DO217934', 'DO1007'] |
| ['DO217934', 'DO217939'] |
| ['DO217939', 'DO217934'] |
| ['DO217950', 'DO218174'] |
| ['DO217950', 'DO23525'] |
| ['DO217962', 'DO217934'] |
| ['DO217987', 'D0'] |
| ['DO218030', 'D0'] |
| ['DO218030', 'DO33042'] |
| ['DO218030', 'DO50408'] |
| ['DO218031', 'D0'] |
| ['DO218075', 'DO1002'] |
| ['DO218075', 'DO50364'] |
| ['DO218088', 'D0'] |
| ['DO218088', 'DO33392'] |
| ['DO218088', 'DO50412'] |
| ['DO218088', 'DO50832'] |
| ['DO218088', 'DO51540'] |
| ['DO218121', 'D0'] |
| ['DO218139', 'DO218535'] |
| ['DO218173', 'DO218174'] |
| ['DO218174', 'D0'] |
| ['DO218174', 'DO217786'] |
| ['DO218174', 'DO217950'] |
| ['DO218174', 'DO45067'] |
| ['DO218174', 'DO50323'] |
| ['DO218180', 'DO221547'] |
| ['DO218205', 'DO1013'] |
| ['DO218223', 'D0'] |
| ['DO218227', 'D0'] |
| ['DO218227', 'DO218280'] |
| ['DO218269', 'D0'] |
| ['DO218280', 'DO48940'] |
| ['DO218282', 'D0'] |
| ['DO218282', 'DO1005'] |
| ['DO218282', 'DO218673'] |
| ['DO218282', 'DO33128'] |
| ['DO218282', 'DO35454'] |
| ['DO218306', 'DO1001'] |
| ['DO218333', 'D0'] |
| ['DO218333', 'DO34849'] |
| ['DO218347', 'DO1013'] |
| ['DO218411', 'D0'] |
| ['DO218417', 'D0'] |
| ['DO218428', 'D0'] |
| ['DO218428', 'DO51498'] |
| ['DO218440', 'D0'] |
| ['DO218440', 'DO50343'] |
| ['DO218442', 'D0'] |
| ['DO218443', 'D0'] |
| ['DO218478', 'DO217826'] |
| ['DO218491', 'D0'] |
| ['DO218502', 'D0'] |
| ['DO218506', 'DO1007'] |
| ['DO218535', 'D0'] |
| ['DO218535', 'DO221542'] |
| ['DO218535', 'DO27775'] |
| ['DO218535', 'DO33208'] |
| ['DO218535', 'DO45065'] |
| ['DO218535', 'DO49074'] |
| ['DO218535', 'DO49424'] |
| ['DO218535', 'DO50345'] |
| ['DO218535', 'DO51505'] |
| ['DO218547', 'D0'] |
| ['DO218550', 'DO23513'] |
| ['DO218560', 'DO1017'] |
| ['DO218560', 'DO217931'] |
| ['DO218611', 'D0'] |
| ['DO218621', 'D0'] |
| ['DO218621', 'DO50325'] |
| ['DO218651', 'D0'] |
| ['DO218651', 'DO34793'] |
| ['DO218651', 'DO49451'] |
| ['DO218651', 'DO52605'] |
| ['DO218673', 'DO218282'] |
| ['DO218673', 'DO23548'] |
| ['DO218684', 'D0'] |
| ['DO218684', 'DO32860'] |
| ['DO218693', 'DO1013'] |
| ['DO218693', 'DO221547'] |
| ['DO218693', 'DO50336'] |
| ['DO218693', 'DO51510'] |
| ['DO218695', 'DO10843'] |
| ['DO218697', 'DO23525'] |
| ['DO218698', 'D0'] |
| ['DO218698', 'DO51521'] |
| ['DO218709', 'D0'] |
| ['DO218736', 'DO1013'] |
| ['DO218736', 'DO217931'] |
| ['DO218769', 'DO221541'] |
| ['DO218828', 'D0'] |
| ['DO220820', 'DO1013'] |
| ['DO220821', 'DO1013'] |
| ['DO220828', 'DO217836'] |
| ['DO220828', 'DO220846'] |
| ['DO220828', 'DO45185'] |
| ['DO220844', 'D0'] |
| ['DO220844', 'DO45199'] |
| ['DO220844', 'DO48939'] |
| ['DO220845', 'D0'] |
| ['DO220845', 'DO220883'] |
| ['DO220845', 'DO220905'] |
| ['DO220846', 'D0'] |
| ['DO220846', 'DO51126'] |
| ['DO220847', 'D0'] |
| ['DO220849', 'DO48695'] |
| ['DO220851', 'DO45207'] |
| ['DO220851', 'DO49076'] |
| ['DO220852', 'DO45183'] |
| ['DO220853', 'DO45183'] |
| ['DO220855', 'DO220845'] |
| ['DO220855', 'DO220887'] |
| ['DO220857', 'DO45183'] |
| ['DO220857', 'DO50334'] |
| ['DO220859', 'D0'] |
| ['DO220860', 'DO45121'] |
| ['DO220861', 'DO49533'] |
| ['DO220862', 'DO45159'] |
| ['DO220862', 'DO49533'] |
| ['DO220866', 'D0'] |
| ['DO220872', 'DO220901'] |
| ['DO220873', 'DO218769'] |
| ['DO220873', 'DO221541'] |
| ['DO220873', 'DO45179'] |
| ['DO220873', 'DO45183'] |
| ['DO220873', 'DO45197'] |
| ['DO220874', 'DO220845'] |
| ['DO220874', 'DO46380'] |
| ['DO220874', 'DO51056'] |
| ['DO220875', 'D0'] |
| ['DO220875', 'DO51491'] |
| ['DO220877', 'DO218282'] |
| ['DO220877', 'DO220884'] |
| ['DO220877', 'DO35733'] |
| ['DO220877', 'DO50806'] |
| ['DO220878', 'DO45115'] |
| ['DO220878', 'DO45183'] |
| ['DO220878', 'DO51187'] |
| ['DO220879', 'DO220901'] |
| ['DO220880', 'DO49533'] |
| ['DO220881', 'DO220901'] |
| ['DO220882', 'DO45183'] |
| ['DO220883', 'DO220845'] |
| ['DO220884', 'D0'] |
| ['DO220885', 'DO49533'] |
| ['DO220886', 'DO220896'] |
| ['DO220886', 'DO50315'] |
| ['DO220887', 'DO220845'] |
| ['DO220887', 'DO45159'] |
| ['DO220889', 'D0'] |
| ['DO220889', 'DO35753'] |
| ['DO220890', 'DO45183'] |
| ['DO220891', 'DO10843'] |
| ['DO220892', 'DO220845'] |
| ['DO220893', 'DO45305'] |
| ['DO220893', 'DO49533'] |
| ['DO220894', 'DO220845'] |
| ['DO220895', 'DO220845'] |
| ['DO220896', 'D0'] |
| ['DO220898', 'DO220901'] |
| ['DO220899', 'D0'] |
| ['DO220900', 'DO218547'] |
| ['DO220900', 'DO220845'] |
| ['DO220901', 'DO220909'] |
| ['DO220902', 'DO220909'] |
| ['DO220903', 'DO220845'] |
| ['DO220903', 'DO23525'] |
| ['DO220903', 'DO46366'] |
| ['DO220903', 'DO48760'] |
| ['DO220904', 'D0'] |
| ['DO220904', 'DO51537'] |
| ['DO220905', 'DO220845'] |
| ['DO220906', 'DO51187'] |
| ['DO220907', 'DO220901'] |
| ['DO220908', 'D0'] |
| ['DO220909', 'DO220901'] |
| ['DO220910', 'DO220845'] |
| ['DO220911', 'DO220845'] |
| ['DO220911', 'DO23525'] |
| ['DO220912', 'DO220847'] |
| ['DO220913', 'DO49533'] |
| ['DO221123', 'DO220845'] |
| ['DO221123', 'DO47048'] |
| ['DO221124', 'D0'] |
| ['DO221124', 'DO27817'] |
| ['DO221124', 'DO52682'] |
| ['DO221129', 'DO52665'] |
| ['DO221539', 'DO221543'] |
| ['DO221539', 'DO46331'] |
| ['DO221540', 'DO221547'] |
| ['DO221540', 'DO46957'] |
| ['DO221541', 'DO218769'] |
| ['DO221542', 'DO218535'] |
| ['DO221542', 'DO221547'] |
| ['DO221542', 'DO32831'] |
| ['DO221543', 'D0'] |
| ['DO221543', 'DO221539'] |
| ['DO221543', 'DO32984'] |
| ['DO221543', 'DO33160'] |
| ['DO221543', 'DO33248'] |
| ['DO221543', 'DO33376'] |
| ['DO221543', 'DO33512'] |
| ['DO221543', 'DO34849'] |
| ['DO221543', 'DO35098'] |
| ['DO221543', 'DO35454'] |
| ['DO221543', 'DO49127'] |
| ['DO221543', 'DO49419'] |
| ['DO221543', 'DO49466'] |
| ['DO221543', 'DO49484'] |
| ['DO221543', 'DO51496'] |
| ['DO221543', 'DO51504'] |
| ['DO221543', 'DO51506'] |
| ['DO221543', 'DO51511'] |
| ['DO221543', 'DO51536'] |
| ['DO221544', 'DO217931'] |
| ['DO221545', 'DO221547'] |
| ['DO221546', 'DO50451'] |
| ['DO221547', 'D0'] |
| ['DO221547', 'DO218180'] |
| ['DO221547', 'DO221540'] |
| ['DO221547', 'DO221542'] |
| ['DO221547', 'DO221545'] |
| ['DO221547', 'DO32831'] |
| ['DO221547', 'DO32837'] |
| ['DO221547', 'DO32860'] |
| ['DO221547', 'DO32900'] |
| ['DO221547', 'DO33042'] |
| ['DO221547', 'DO33128'] |
| ['DO221547', 'DO33368'] |
| ['DO221547', 'DO33408'] |
| ['DO221547', 'DO34720'] |
| ['DO221547', 'DO34793'] |
| ['DO221547', 'DO34905'] |
| ['DO221547', 'DO35085'] |
| ['DO221547', 'DO35148'] |
| ['DO221547', 'DO35152'] |
| ['DO221547', 'DO35184'] |
| ['DO221547', 'DO35258'] |
| ['DO221547', 'DO35376'] |
| ['DO221547', 'DO35406'] |
| ['DO221547', 'DO49439'] |
| ['DO221547', 'DO49457'] |
| ['DO221547', 'DO51465'] |
| ['DO221547', 'DO51478'] |
| ['DO221547', 'DO51498'] |
| ['DO221547', 'DO51501'] |
| ['DO221547', 'DO51512'] |
| ['DO221547', 'DO51518'] |
| ['DO221547', 'DO51523'] |
| ['DO221547', 'DO51524'] |
| ['DO221547', 'DO51537'] |
| ['DO221547', 'DO51540'] |
| ['DO221547', 'DO51542'] |
| ['DO221548', 'D0'] |
| ['DO222299', 'D0'] |
| ['DO23508', 'D0'] |
| ['DO23508', 'DO33160'] |
| ['DO23509', 'DO50806'] |
| ['DO23509', 'DO51078'] |
| ['DO23513', 'D0'] |
| ['DO23514', 'D0'] |
| ['DO23517', 'D0'] |
| ['DO23518', 'D0'] |
| ['DO23518', 'DO45067'] |
| ['DO23518', 'DO50343'] |
| ['DO23522', 'D0'] |
| ['DO23525', 'D0'] |
| ['DO23525', 'DO217950'] |
| ['DO23525', 'DO218697'] |
| ['DO23525', 'DO32984'] |
| ['DO23527', 'D0'] |
| ['DO23527', 'DO45094'] |
| ['DO23529', 'D0'] |
| ['DO23534', 'D0'] |
| ['DO23534', 'DO45083'] |
| ['DO23534', 'DO45177'] |
| ['DO23534', 'DO45199'] |
| ['DO23534', 'DO48692'] |
| ['DO23535', 'D0'] |
| ['DO23535', 'DO51070'] |
| ['DO23542', 'D0'] |
| ['DO23542', 'DO44740'] |
| ['DO23542', 'DO49466'] |
| ['DO23543', 'DO23529'] |
| ['DO23543', 'DO45129'] |
| ['DO23545', 'D0'] |
| ['DO23545', 'DO49178'] |
| ['DO23548', 'D0'] |
| ['DO23548', 'DO218673'] |
| ['DO23548', 'DO50842'] |
| ['DO27763', 'D0'] |
| ['DO27764', 'D0'] |
| ['DO27765', 'D0'] |
| ['DO27767', 'DO27763'] |
| ['DO27767', 'DO27764'] |
| ['DO27769', 'DO218535'] |
| ['DO27769', 'DO27764'] |
| ['DO27773', 'D0'] |
| ['DO27775', 'DO218535'] |
| ['DO27779', 'D0'] |
| ['DO27779', 'DO52659'] |
| ['DO27783', 'D0'] |
| ['DO27785', 'D0'] |
| ['DO27787', 'D0'] |
| ['DO27789', 'D0'] |
| ['DO27791', 'DO7084'] |
| ['DO27793', 'D0'] |
| ['DO27793', 'DO27819'] |
| ['DO27795', 'DO50327'] |
| ['DO27797', 'D0'] |
| ['DO27799', 'D0'] |
| ['DO27801', 'D0'] |
| ['DO27803', 'D0'] |
| ['DO27803', 'DO27837'] |
| ['DO27805', 'D0'] |
| ['DO27805', 'DO27837'] |
| ['DO27805', 'DO52656'] |
| ['DO27807', 'D0'] |
| ['DO27807', 'DO27855'] |
| ['DO27809', 'D0'] |
| ['DO27811', 'D0'] |
| ['DO27815', 'D0'] |
| ['DO27817', 'D0'] |
| ['DO27819', 'DO46897'] |
| ['DO27821', 'DO218440'] |
| ['DO27825', 'DO218030'] |
| ['DO27827', 'D0'] |
| ['DO27829', 'DO50370'] |
| ['DO27833', 'DO218535'] |
| ['DO27833', 'DO52706'] |
| ['DO27835', 'D0'] |
| ['DO27835', 'DO52656'] |
| ['DO27837', 'D0'] |
| ['DO27849', 'DO45149'] |
| ['DO27851', 'DO51063'] |
| ['DO27851', 'DO51126'] |
| ['DO27853', 'DO52657'] |
| ['DO27855', 'DO27807'] |
| ['DO27857', 'DO52649'] |
| ['DO27857', 'DO52653'] |
| ['DO27859', 'D0'] |
| ['DO32831', 'DO221542'] |
| ['DO32831', 'DO221547'] |
| ['DO32831', 'DO45065'] |
| ['DO32837', 'DO10843'] |
| ['DO32837', 'DO221547'] |
| ['DO32860', 'DO218684'] |
| ['DO32860', 'DO221547'] |
| ['DO32878', 'DO33184'] |
| ['DO32893', 'DO33184'] |
| ['DO32893', 'DO46331'] |
| ['DO32900', 'DO221547'] |
| ['DO32980', 'D0'] |
| ['DO32980', 'DO33248'] |
| ['DO32984', 'DO221543'] |
| ['DO32984', 'DO23525'] |
| ['DO33000', 'DO33184'] |
| ['DO33000', 'DO35424'] |
| ['DO33008', 'DO33184'] |
| ['DO33032', 'D0'] |
| ['DO33042', 'DO218030'] |
| ['DO33042', 'DO221547'] |
| ['DO33042', 'DO49193'] |
| ['DO33128', 'DO218282'] |
| ['DO33128', 'DO221547'] |
| ['DO33152', 'DO221543'] |
| ['DO33160', 'DO221543'] |
| ['DO33160', 'DO23508'] |
| ['DO33168', 'DO218535'] |
| ['DO33168', 'DO221543'] |
| ['DO33184', 'D0'] |
| ['DO33184', 'DO32878'] |
| ['DO33184', 'DO32893'] |
| ['DO33184', 'DO33000'] |
| ['DO33184', 'DO33008'] |
| ['DO33184', 'DO34448'] |
| ['DO33184', 'DO34608'] |
| ['DO33184', 'DO35126'] |
| ['DO33184', 'DO35128'] |
| ['DO33184', 'DO35216'] |
| ['DO33184', 'DO35424'] |
| ['DO33184', 'DO46586'] |
| ['DO33184', 'DO49113'] |
| ['DO33184', 'DO49204'] |
| ['DO33184', 'DO49424'] |
| ['DO33184', 'DO49451'] |
| ['DO33184', 'DO51466'] |
| ['DO33184', 'DO51467'] |
| ['DO33184', 'DO51470'] |
| ['DO33184', 'DO51473'] |
| ['DO33184', 'DO51486'] |
| ['DO33184', 'DO51493'] |
| ['DO33184', 'DO51533'] |
| ['DO33184', 'DO51535'] |
| ['DO33200', 'DO51540'] |
| ['DO33208', 'DO218535'] |
| ['DO33208', 'DO33480'] |
| ['DO33248', 'DO221543'] |
| ['DO33248', 'DO32980'] |
| ['DO33264', 'DO1013'] |
| ['DO33264', 'DO221547'] |
| ['DO33368', 'DO221547'] |
| ['DO33376', 'DO221543'] |
| ['DO33376', 'DO46416'] |
| ['DO33392', 'DO218088'] |
| ['DO33392', 'DO33480'] |
| ['DO33408', 'DO1010'] |
| ['DO33408', 'DO221547'] |
| ['DO33480', 'D0'] |
| ['DO33480', 'DO33208'] |
| ['DO33480', 'DO33392'] |
| ['DO33480', 'DO33544'] |
| ['DO33480', 'DO48692'] |
| ['DO33480', 'DO49463'] |
| ['DO33512', 'DO221543'] |
| ['DO33544', 'DO33480'] |
| ['DO33544', 'DO45273'] |
| ['DO33600', 'DO221547'] |
| ['DO34312', 'DO32900'] |
| ['DO34432', 'DO34504'] |
| ['DO34448', 'DO33184'] |
| ['DO34504', 'DO34432'] |
| ['DO34608', 'DO1010'] |
| ['DO34608', 'DO33184'] |
| ['DO34656', 'DO50407'] |
| ['DO34720', 'DO221547'] |
| ['DO34728', 'DO32984'] |
| ['DO34736', 'DO33184'] |
| ['DO34736', 'DO45091'] |
| ['DO34785', 'DO221543'] |
| ['DO34793', 'DO218651'] |
| ['DO34793', 'DO221547'] |
| ['DO34849', 'DO218333'] |
| ['DO34849', 'DO221543'] |
| ['DO34905', 'DO221547'] |
| ['DO35083', 'DO23525'] |
| ['DO35083', 'DO33184'] |
| ['DO35083', 'DO48914'] |
| ['DO35083', 'DO50330'] |
| ['DO35083', 'DO50367'] |
| ['DO35085', 'DO221547'] |
| ['DO35085', 'DO46448'] |
| ['DO35098', 'DO221543'] |
| ['DO35098', 'DO51111'] |
| ['DO35116', 'DO33184'] |
| ['DO35126', 'DO33184'] |
| ['DO35126', 'DO46388'] |
| ['DO35128', 'DO33184'] |
| ['DO35128', 'DO52549'] |
| ['DO35136', 'DO51507'] |
| ['DO35144', 'DO51529'] |
| ['DO35148', 'DO221547'] |
| ['DO35148', 'DO46331'] |
| ['DO35152', 'DO221547'] |
| ['DO35152', 'DO51490'] |
| ['DO35184', 'DO221547'] |
| ['DO35216', 'DO217931'] |
| ['DO35216', 'DO33184'] |
| ['DO35222', 'D0'] |
| ['DO35222', 'DO49074'] |
| ['DO35222', 'DO49178'] |
| ['DO35228', 'DO221547'] |
| ['DO35228', 'DO49193'] |
| ['DO35228', 'DO52547'] |
| ['DO35236', 'DO221546'] |
| ['DO35236', 'DO50451'] |
| ['DO35258', 'DO221547'] |
| ['DO35258', 'DO52556'] |
| ['DO35350', 'DO221543'] |
| ['DO35376', 'DO221547'] |
| ['DO35376', 'DO46327'] |
| ['DO35406', 'DO221547'] |
| ['DO35406', 'DO45035'] |
| ['DO35424', 'DO33000'] |
| ['DO35424', 'DO33184'] |
| ['DO35424', 'DO50336'] |
| ['DO35442', 'D0'] |
| ['DO35442', 'DO35555'] |
| ['DO35442', 'DO49421'] |
| ['DO35454', 'DO218282'] |
| ['DO35454', 'DO221543'] |
| ['DO35547', 'D0'] |
| ['DO35547', 'DO48888'] |
| ['DO35547', 'DO48915'] |
| ['DO35555', 'D0'] |
| ['DO35555', 'DO45064'] |
| ['DO35568', 'D0'] |
| ['DO35568', 'DO48757'] |
| ['DO35704', 'D0'] |
| ['DO35733', 'D0'] |
| ['DO35753', 'D0'] |
| ['DO35937', 'D0'] |
| ['DO35982', 'D0'] |
| ['DO36221', 'DO217931'] |
| ['DO36223', 'DO51061'] |
| ['DO44740', 'DO23542'] |
| ['DO44806', 'D0'] |
| ['DO44806', 'DO48679'] |
| ['DO44832', 'D0'] |
| ['DO44832', 'DO45267'] |
| ['DO45035', 'D0'] |
| ['DO45035', 'DO35406'] |
| ['DO45035', 'DO50337'] |
| ['DO45035', 'DO51493'] |
| ['DO45039', 'DO45281'] |
| ['DO45039', 'DO46551'] |
| ['DO45041', 'DO220904'] |
| ['DO45041', 'DO23534'] |
| ['DO45047', 'D0'] |
| ['DO45047', 'DO50390'] |
| ['DO45049', 'D0'] |
| ['DO45055', 'D0'] |
| ['DO45064', 'D0'] |
| ['DO45064', 'DO35555'] |
| ['DO45065', 'D0'] |
| ['DO45065', 'DO218535'] |
| ['DO45065', 'DO32831'] |
| ['DO45065', 'DO50389'] |
| ['DO45067', 'DO218174'] |
| ['DO45067', 'DO23518'] |
| ['DO45071', 'DO218684'] |
| ['DO45071', 'DO23518'] |
| ['DO45071', 'DO45153'] |
| ['DO45075', 'DO46366'] |
| ['DO45079', 'D0'] |
| ['DO45081', 'D0'] |
| ['DO45081', 'DO49484'] |
| ['DO45083', 'DO1010'] |
| ['DO45083', 'DO23534'] |
| ['DO45091', 'D0'] |
| ['DO45091', 'DO48679'] |
| ['DO45093', 'D0'] |
| ['DO45093', 'DO51523'] |
| ['DO45094', 'DO23527'] |
| ['DO45096', 'D0'] |
| ['DO45097', 'D0'] |
| ['DO45115', 'D0'] |
| ['DO45117', 'D0'] |
| ['DO45121', 'D0'] |
| ['DO45121', 'DO220860'] |
| ['DO45123', 'D0'] |
| ['DO45127', 'D0'] |
| ['DO45129', 'D0'] |
| ['DO45141', 'DO44806'] |
| ['DO45149', 'D0'] |
| ['DO45153', 'D0'] |
| ['DO45159', 'D0'] |
| ['DO45161', 'DO218442'] |
| ['DO45161', 'DO23527'] |
| ['DO45161', 'DO48732'] |
| ['DO45177', 'DO1013'] |
| ['DO45177', 'DO23534'] |
| ['DO45179', 'D0'] |
| ['DO45183', 'D0'] |
| ['DO45183', 'DO220853'] |
| ['DO45185', 'D0'] |
| ['DO45189', 'D0'] |
| ['DO45197', 'D0'] |
| ['DO45199', 'DO220844'] |
| ['DO45199', 'DO23534'] |
| ['DO45201', 'D0'] |
| ['DO45207', 'D0'] |
| ['DO45255', 'DO50857'] |
| ['DO45259', 'D0'] |
| ['DO45265', 'D0'] |
| ['DO45265', 'DO50362'] |
| ['DO45267', 'DO44832'] |
| ['DO45267', 'DO46372'] |
| ['DO45273', 'D0'] |
| ['DO45273', 'DO33544'] |
| ['DO45273', 'DO50393'] |
| ['DO45281', 'D0'] |
| ['DO45281', 'DO45039'] |
| ['DO45287', 'D0'] |
| ['DO45287', 'DO48888'] |
| ['DO45293', 'D0'] |
| ['DO45299', 'DO51090'] |
| ['DO45305', 'D0'] |
| ['DO46327', 'D0'] |
| ['DO46327', 'DO35376'] |
| ['DO46327', 'DO51506'] |
| ['DO46330', 'DO217844'] |
| ['DO46330', 'DO218535'] |
| ['DO46331', 'D0'] |
| ['DO46331', 'DO221539'] |
| ['DO46331', 'DO32893'] |
| ['DO46331', 'DO35148'] |
| ['DO46331', 'DO50306'] |
| ['DO46331', 'DO50309'] |
| ['DO46331', 'DO50329'] |
| ['DO46333', 'D0'] |
| ['DO46333', 'DO51535'] |
| ['DO46350', 'D0'] |
| ['DO46350', 'DO1010'] |
| ['DO46358', 'D0'] |
| ['DO46358', 'DO50444'] |
| ['DO46358', 'DO52605'] |
| ['DO46366', 'D0'] |
| ['DO46366', 'DO45075'] |
| ['DO46372', 'D0'] |
| ['DO46372', 'DO45267'] |
| ['DO46378', 'DO50336'] |
| ['DO46380', 'D0'] |
| ['DO46380', 'DO51467'] |
| ['DO46388', 'D0'] |
| ['DO46388', 'DO35126'] |
| ['DO46396', 'D0'] |
| ['DO46398', 'DO217818'] |
| ['DO46402', 'D0'] |
| ['DO46402', 'DO51542'] |
| ['DO46404', 'D0'] |
| ['DO46404', 'DO49127'] |
| ['DO46412', 'DO1010'] |
| ['DO46416', 'D0'] |
| ['DO46416', 'DO33376'] |
| ['DO46420', 'DO10843'] |
| ['DO46424', 'D0'] |
| ['DO46448', 'D0'] |
| ['DO46448', 'DO35085'] |
| ['DO46448', 'DO46586'] |
| ['DO46488', 'D0'] |
| ['DO46551', 'D0'] |
| ['DO46551', 'DO45039'] |
| ['DO46568', 'D0'] |
| ['DO46568', 'DO50345'] |
| ['DO46568', 'DO6398'] |
| ['DO46586', 'DO33184'] |
| ['DO46586', 'DO46448'] |
| ['DO46591', 'D0'] |
| ['DO46606', 'D0'] |
| ['DO46779', 'D0'] |
| ['DO46783', 'DO50330'] |
| ['DO46783', 'DO52549'] |
| ['DO46792', 'D0'] |
| ['DO46795', 'D0'] |
| ['DO46832', 'D0'] |
| ['DO46832', 'DO46885'] |
| ['DO46834', 'D0'] |
| ['DO46838', 'D0'] |
| ['DO46877', 'D0'] |
| ['DO46877', 'DO49113'] |
| ['DO46881', 'D0'] |
| ['DO46885', 'DO46832'] |
| ['DO46893', 'D0'] |
| ['DO46897', 'D0'] |
| ['DO46897', 'DO27819'] |
| ['DO46909', 'D0'] |
| ['DO46957', 'D0'] |
| ['DO46957', 'DO221540'] |
| ['DO46980', 'D0'] |
| ['DO47016', 'DO47068'] |
| ['DO47048', 'D0'] |
| ['DO47068', 'D0'] |
| ['DO47068', 'DO47016'] |
| ['DO48541', 'D0'] |
| ['DO48541', 'DO50410'] |
| ['DO48577', 'D0'] |
| ['DO48578', 'DO51178'] |
| ['DO48679', 'DO44806'] |
| ['DO48679', 'DO45091'] |
| ['DO48682', 'DO52582'] |
| ['DO48689', 'D0'] |
| ['DO48692', 'DO23534'] |
| ['DO48692', 'DO33480'] |
| ['DO48695', 'D0'] |
| ['DO48695', 'DO220849'] |
| ['DO48715', 'D0'] |
| ['DO48719', 'D0'] |
| ['DO48723', 'D0'] |
| ['DO48727', 'D0'] |
| ['DO48732', 'D0'] |
| ['DO48757', 'DO35568'] |
| ['DO48760', 'D0'] |
| ['DO48888', 'DO35547'] |
| ['DO48891', 'D0'] |
| ['DO48891', 'DO48939'] |
| ['DO48893', 'D0'] |
| ['DO48895', 'D0'] |
| ['DO48900', 'D0'] |
| ['DO48914', 'D0'] |
| ['DO48915', 'DO217887'] |
| ['DO48915', 'DO35547'] |
| ['DO48939', 'DO220844'] |
| ['DO48939', 'DO48891'] |
| ['DO48940', 'D0'] |
| ['DO48940', 'DO218280'] |
| ['DO48945', 'DO45305'] |
| ['DO48964', 'D0'] |
| ['DO49074', 'DO218535'] |
| ['DO49074', 'DO35222'] |
| ['DO49076', 'D0'] |
| ['DO49079', 'DO10815'] |
| ['DO49079', 'DO33184'] |
| ['DO49080', 'D0'] |
| ['DO49087', 'DO218030'] |
| ['DO49087', 'DO218621'] |
| ['DO49113', 'DO33184'] |
| ['DO49113', 'DO46877'] |
| ['DO49127', 'DO221543'] |
| ['DO49127', 'DO46404'] |
| ['DO49135', 'DO221546'] |
| ['DO49135', 'DO50451'] |
| ['DO49138', 'DO217836'] |
| ['DO49138', 'DO218269'] |
| ['DO49138', 'DO33184'] |
| ['DO49168', 'DO51540'] |
| ['DO49178', 'DO23545'] |
| ['DO49178', 'DO35222'] |
| ['DO49193', 'DO33042'] |
| ['DO49204', 'DO10843'] |
| ['DO49204', 'DO33184'] |
| ['DO49418', 'DO33032'] |
| ['DO49418', 'DO50336'] |
| ['DO49419', 'DO221543'] |
| ['DO49420', 'DO33128'] |
| ['DO49421', 'D0'] |
| ['DO49421', 'DO48757'] |
| ['DO49424', 'DO218535'] |
| ['DO49424', 'DO33184'] |
| ['DO49436', 'DO221543'] |
| ['DO49436', 'DO50327'] |
| ['DO49439', 'DO217931'] |
| ['DO49439', 'DO221547'] |
| ['DO49442', 'D0'] |
| ['DO49442', 'DO27815'] |
| ['DO49442', 'DO46488'] |
| ['DO49442', 'DO51078'] |
| ['DO49442', 'DO52742'] |
| ['DO49448', 'DO50381'] |
| ['DO49448', 'DO50440'] |
| ['DO49451', 'DO218651'] |
| ['DO49451', 'DO33184'] |
| ['DO49454', 'DO33248'] |
| ['DO49457', 'DO221547'] |
| ['DO49457', 'DO50785'] |
| ['DO49457', 'DO51490'] |
| ['DO49463', 'DO33480'] |
| ['DO49463', 'DO50327'] |
| ['DO49466', 'DO221543'] |
| ['DO49466', 'DO23542'] |
| ['DO49484', 'DO221543'] |
| ['DO49484', 'DO45081'] |
| ['DO49533', 'D0'] |
| ['DO49537', 'D0'] |
| ['DO49537', 'DO50450'] |
| ['DO50306', 'DO217896'] |
| ['DO50306', 'DO46331'] |
| ['DO50309', 'DO217931'] |
| ['DO50309', 'DO46331'] |
| ['DO50311', 'D0'] |
| ['DO50314', 'D0'] |
| ['DO50314', 'DO51512'] |
| ['DO50314', 'DO51524'] |
| ['DO50315', 'D0'] |
| ['DO50316', 'DO50820'] |
| ['DO50318', 'DO50364'] |
| ['DO50323', 'DO218174'] |
| ['DO50325', 'DO218621'] |
| ['DO50325', 'DO50327'] |
| ['DO50326', 'DO217987'] |
| ['DO50327', 'D0'] |
| ['DO50327', 'DO49463'] |
| ['DO50327', 'DO50325'] |
| ['DO50327', 'DO50355'] |
| ['DO50328', 'D0'] |
| ['DO50329', 'DO46331'] |
| ['DO50330', 'D0'] |
| ['DO50330', 'DO46783'] |
| ['DO50330', 'DO50408'] |
| ['DO50331', 'DO10843'] |
| ['DO50331', 'DO50441'] |
| ['DO50332', 'D0'] |
| ['DO50332', 'DO50318'] |
| ['DO50332', 'DO50393'] |
| ['DO50332', 'DO50398'] |
| ['DO50334', 'D0'] |
| ['DO50336', 'D0'] |
| ['DO50336', 'DO35424'] |
| ['DO50336', 'DO46378'] |
| ['DO50337', 'DO45035'] |
| ['DO50338', 'DO46380'] |
| ['DO50340', 'D0'] |
| ['DO50340', 'DO45075'] |
| ['DO50342', 'DO45201'] |
| ['DO50343', 'DO218440'] |
| ['DO50343', 'DO23518'] |
| ['DO50345', 'DO218535'] |
| ['DO50345', 'DO46568'] |
| ['DO50346', 'DO218030'] |
| ['DO50346', 'DO23517'] |
| ['DO50348', 'DO217800'] |
| ['DO50350', 'DO217800'] |
| ['DO50355', 'DO50327'] |
| ['DO50362', 'DO45265'] |
| ['DO50364', 'D0'] |
| ['DO50364', 'DO218075'] |
| ['DO50364', 'DO50318'] |
| ['DO50364', 'DO50454'] |
| ['DO50364', 'DO51533'] |
| ['DO50367', 'D0'] |
| ['DO50370', 'D0'] |
| ['DO50370', 'DO50407'] |
| ['DO50370', 'DO51074'] |
| ['DO50372', 'D0'] |
| ['DO50381', 'DO49448'] |
| ['DO50381', 'DO50440'] |
| ['DO50382', 'DO45281'] |
| ['DO50384', 'D0'] |
| ['DO50384', 'DO35136'] |
| ['DO50384', 'DO50450'] |
| ['DO50387', 'D0'] |
| ['DO50387', 'DO46378'] |
| ['DO50387', 'DO52597'] |
| ['DO50389', 'D0'] |
| ['DO50389', 'DO45065'] |
| ['DO50390', 'DO45047'] |
| ['DO50393', 'DO45273'] |
| ['DO50398', 'DO50785'] |
| ['DO50399', 'D0'] |
| ['DO50399', 'DO51491'] |
| ['DO50402', 'D0'] |
| ['DO50406', 'DO218535'] |
| ['DO50407', 'D0'] |
| ['DO50407', 'DO34656'] |
| ['DO50407', 'DO50370'] |
| ['DO50408', 'DO218030'] |
| ['DO50408', 'DO50330'] |
| ['DO50410', 'DO48541'] |
| ['DO50411', 'DO23513'] |
| ['DO50412', 'DO218088'] |
| ['DO50419', 'D0'] |
| ['DO50430', 'D0'] |
| ['DO50430', 'DO50390'] |
| ['DO50430', 'DO51070'] |
| ['DO50431', 'D0'] |
| ['DO50440', 'DO49448'] |
| ['DO50440', 'DO50381'] |
| ['DO50441', 'D0'] |
| ['DO50443', 'D0'] |
| ['DO50443', 'DO10841'] |
| ['DO50444', 'DO46358'] |
| ['DO50445', 'DO10843'] |
| ['DO50446', 'DO218411'] |
| ['DO50446', 'DO50311'] |
| ['DO50447', 'DO50452'] |
| ['DO50449', 'DO218174'] |
| ['DO50449', 'DO50327'] |
| ['DO50449', 'DO50330'] |
| ['DO50450', 'DO49537'] |
| ['DO50451', 'DO221546'] |
| ['DO50452', 'D0'] |
| ['DO50452', 'DO50362'] |
| ['DO50452', 'DO50447'] |
| ['DO50453', 'DO217836'] |
| ['DO50453', 'DO45047'] |
| ['DO50454', 'DO50364'] |
| ['DO50785', 'D0'] |
| ['DO50785', 'DO49457'] |
| ['DO50785', 'DO50398'] |
| ['DO50793', 'DO1013'] |
| ['DO50793', 'DO23542'] |
| ['DO50806', 'D0'] |
| ['DO50806', 'DO23509'] |
| ['DO50818', 'DO23527'] |
| ['DO50818', 'DO6362'] |
| ['DO50820', 'D0'] |
| ['DO50829', 'D0'] |
| ['DO50829', 'DO51504'] |
| ['DO50832', 'DO218088'] |
| ['DO50832', 'DO51485'] |
| ['DO50834', 'D0'] |
| ['DO50834', 'DO51133'] |
| ['DO50842', 'DO23548'] |
| ['DO50844', 'DO1001'] |
| ['DO50857', 'D0'] |
| ['DO50857', 'DO45255'] |
| ['DO51050', 'D0'] |
| ['DO51056', 'D0'] |
| ['DO51061', 'D0'] |
| ['DO51063', 'D0'] |
| ['DO51070', 'DO23535'] |
| ['DO51074', 'D0'] |
| ['DO51074', 'DO51466'] |
| ['DO51078', 'D0'] |
| ['DO51078', 'DO23509'] |
| ['DO51087', 'D0'] |
| ['DO51090', 'D0'] |
| ['DO51111', 'D0'] |
| ['DO51111', 'DO35098'] |
| ['DO51126', 'D0'] |
| ['DO51126', 'DO220846'] |
| ['DO51133', 'DO1013'] |
| ['DO51133', 'DO50834'] |
| ['DO51141', 'D0'] |
| ['DO51178', 'DO48578'] |
| ['DO51187', 'D0'] |
| ['DO51465', 'DO221547'] |
| ['DO51466', 'DO33184'] |
| ['DO51466', 'DO51074'] |
| ['DO51467', 'DO33184'] |
| ['DO51467', 'DO46380'] |
| ['DO51470', 'DO1001'] |
| ['DO51470', 'DO33184'] |
| ['DO51470', 'DO51475'] |
| ['DO51473', 'DO33184'] |
| ['DO51474', 'DO221543'] |
| ['DO51475', 'DO51470'] |
| ['DO51478', 'DO221547'] |
| ['DO51481', 'DO221546'] |
| ['DO51481', 'DO50451'] |
| ['DO51484', 'DO33184'] |
| ['DO51484', 'DO51502'] |
| ['DO51485', 'DO50832'] |
| ['DO51485', 'DO51540'] |
| ['DO51486', 'DO33184'] |
| ['DO51486', 'DO51521'] |
| ['DO51490', 'DO35152'] |
| ['DO51490', 'DO49457'] |
| ['DO51491', 'DO50399'] |
| ['DO51493', 'DO33184'] |
| ['DO51493', 'DO45035'] |
| ['DO51496', 'DO221543'] |
| ['DO51497', 'DO10843'] |
| ['DO51497', 'DO221543'] |
| ['DO51498', 'DO218428'] |
| ['DO51498', 'DO221547'] |
| ['DO51500', 'DO218621'] |
| ['DO51500', 'DO48900'] |
| ['DO51501', 'DO221547'] |
| ['DO51501', 'DO52549'] |
| ['DO51502', 'D0'] |
| ['DO51504', 'DO221543'] |
| ['DO51504', 'DO50829'] |
| ['DO51505', 'DO218535'] |
| ['DO51506', 'DO221543'] |
| ['DO51506', 'DO46327'] |
| ['DO51507', 'D0'] |
| ['DO51507', 'DO35136'] |
| ['DO51510', 'D0'] |
| ['DO51511', 'DO1010'] |
| ['DO51511', 'DO221543'] |
| ['DO51512', 'DO221547'] |
| ['DO51515', 'DO33184'] |
| ['DO51515', 'DO50327'] |
| ['DO51518', 'DO1001'] |
| ['DO51518', 'DO221547'] |
| ['DO51521', 'D0'] |
| ['DO51521', 'DO218698'] |
| ['DO51521', 'DO51486'] |
| ['DO51523', 'DO221547'] |
| ['DO51523', 'DO45093'] |
| ['DO51524', 'DO221547'] |
| ['DO51528', 'DO218621'] |
| ['DO51528', 'DO33184'] |
| ['DO51529', 'DO35144'] |
| ['DO51533', 'DO33184'] |
| ['DO51533', 'DO50364'] |
| ['DO51534', 'DO23525'] |
| ['DO51534', 'DO33184'] |
| ['DO51534', 'DO35116'] |
| ['DO51535', 'DO33184'] |
| ['DO51535', 'DO46333'] |
| ['DO51536', 'DO221543'] |
| ['DO51537', 'DO220904'] |
| ['DO51537', 'DO221547'] |
| ['DO51538', 'DO34720'] |
| ['DO51540', 'DO218088'] |
| ['DO51540', 'DO221547'] |
| ['DO51540', 'DO33200'] |
| ['DO51540', 'DO49168'] |
| ['DO51540', 'DO51485'] |
| ['DO51542', 'DO221547'] |
| ['DO51543', 'DO217818'] |
| ['DO51543', 'DO221543'] |
| ['DO51548', 'DO33184'] |
| ['DO51548', 'DO45123'] |
| ['DO51549', 'DO10844'] |
| ['DO51549', 'DO33184'] |
| ['DO51953', 'DO217907'] |
| ['DO51954', 'D0'] |
| ['DO51954', 'DO218411'] |
| ['DO51954', 'DO46881'] |
| ['DO51955', 'D0'] |
| ['DO51956', 'D0'] |
| ['DO51958', 'DO44806'] |
| ['DO51958', 'DO46893'] |
| ['DO51959', 'D0'] |
| ['DO51959', 'DO46832'] |
| ['DO51959', 'DO46877'] |
| ['DO51959', 'DO51087'] |
| ['DO51959', 'DO51111'] |
| ['DO51960', 'DO218223'] |
| ['DO51962', 'D0'] |
| ['DO51962', 'DO218333'] |
| ['DO51962', 'DO48732'] |
| ['DO51964', 'D0'] |
| ['DO51964', 'DO10841'] |
| ['DO51964', 'DO218180'] |
| ['DO51964', 'DO45115'] |
| ['DO51964', 'DO46881'] |
| ['DO51964', 'DO48727'] |
| ['DO51964', 'DO50340'] |
| ['DO51964', 'DO51510'] |
| ['DO51964', 'DO52575'] |
| ['DO51965', 'D0'] |
| ['DO51965', 'DO220844'] |
| ['DO51965', 'DO23514'] |
| ['DO51965', 'DO23518'] |
| ['DO51965', 'DO23527'] |
| ['DO51965', 'DO23529'] |
| ['DO51965', 'DO23534'] |
| ['DO51965', 'DO23542'] |
| ['DO51965', 'DO23548'] |
| ['DO51965', 'DO35568'] |
| ['DO51965', 'DO44806'] |
| ['DO51965', 'DO44832'] |
| ['DO51965', 'DO45097'] |
| ['DO51965', 'DO45117'] |
| ['DO51965', 'DO45127'] |
| ['DO51965', 'DO45129'] |
| ['DO51965', 'DO45179'] |
| ['DO51965', 'DO45189'] |
| ['DO51965', 'DO45199'] |
| ['DO51965', 'DO45259'] |
| ['DO51965', 'DO45281'] |
| ['DO51965', 'DO48689'] |
| ['DO51965', 'DO50399'] |
| ['DO51965', 'DO50834'] |
| ['DO51965', 'DO50857'] |
| ['DO52150', 'D0'] |
| ['DO52150', 'DO6934'] |
| ['DO52509', 'D0'] |
| ['DO52510', 'D0'] |
| ['DO52510', 'DO52591'] |
| ['DO52511', 'D0'] |
| ['DO52512', 'D0'] |
| ['DO52512', 'DO27799'] |
| ['DO52513', 'D0'] |
| ['DO52543', 'DO52549'] |
| ['DO52544', 'D0'] |
| ['DO52547', 'D0'] |
| ['DO52549', 'D0'] |
| ['DO52549', 'DO35128'] |
| ['DO52549', 'DO46783'] |
| ['DO52549', 'DO51501'] |
| ['DO52549', 'DO52543'] |
| ['DO52556', 'D0'] |
| ['DO52556', 'DO35258'] |
| ['DO52557', 'D0'] |
| ['DO52558', 'DO1013'] |
| ['DO52558', 'DO52544'] |
| ['DO52561', 'D0'] |
| ['DO52561', 'DO33008'] |
| ['DO52575', 'D0'] |
| ['DO52582', 'D0'] |
| ['DO52591', 'D0'] |
| ['DO52591', 'DO217907'] |
| ['DO52597', 'D0'] |
| ['DO52605', 'DO218651'] |
| ['DO52605', 'DO46358'] |
| ['DO52623', 'D0'] |
| ['DO52647', 'D0'] |
| ['DO52647', 'DO27835'] |
| ['DO52648', 'D0'] |
| ['DO52649', 'D0'] |
| ['DO52650', 'D0'] |
| ['DO52651', 'DO221129'] |
| ['DO52652', 'D0'] |
| ['DO52653', 'DO52649'] |
| ['DO52654', 'D0'] |
| ['DO52654', 'DO52704'] |
| ['DO52655', 'D0'] |
| ['DO52656', 'D0'] |
| ['DO52657', 'D0'] |
| ['DO52659', 'D0'] |
| ['DO52659', 'DO27775'] |
| ['DO52661', 'DO46897'] |
| ['DO52662', 'D0'] |
| ['DO52663', 'D0'] |
| ['DO52663', 'DO52666'] |
| ['DO52664', 'DO27789'] |
| ['DO52665', 'D0'] |
| ['DO52665', 'DO221129'] |
| ['DO52666', 'D0'] |
| ['DO52667', 'D0'] |
| ['DO52669', 'D0'] |
| ['DO52670', 'DO52665'] |
| ['DO52671', 'D0'] |
| ['DO52672', 'DO218769'] |
| ['DO52672', 'DO221541'] |
| ['DO52674', 'DO221548'] |
| ['DO52675', 'DO52648'] |
| ['DO52677', 'DO218088'] |
| ['DO52679', 'DO52706'] |
| ['DO52681', 'D0'] |
| ['DO52682', 'D0'] |
| ['DO52684', 'DO27807'] |
| ['DO52685', 'D0'] |
| ['DO52685', 'DO6432'] |
| ['DO52686', 'DO35753'] |
| ['DO52686', 'DO45055'] |
| ['DO52687', 'D0'] |
| ['DO52688', 'D0'] |
| ['DO52688', 'DO27791'] |
| ['DO52689', 'DO27773'] |
| ['DO52690', 'D0'] |
| ['DO52690', 'DO27817'] |
| ['DO52691', 'DO51141'] |
| ['DO52692', 'D0'] |
| ['DO52692', 'DO27811'] |
| ['DO52692', 'DO27819'] |
| ['DO52692', 'DO52666'] |
| ['DO52692', 'DO52670'] |
| ['DO52692', 'DO52682'] |
| ['DO52693', 'D0'] |
| ['DO52694', 'DO7112'] |
| ['DO52695', 'D0'] |
| ['DO52695', 'DO27817'] |
| ['DO52698', 'D0'] |
| ['DO52698', 'DO6934'] |
| ['DO52703', 'D0'] |
| ['DO52704', 'D0'] |
| ['DO52706', 'D0'] |
| ['DO52712', 'D0'] |
| ['DO52718', 'D0'] |
| ['DO52729', 'D0'] |
| ['DO52732', 'D0'] |
| ['DO52733', 'D0'] |
| ['DO52736', 'D0'] |
| ['DO52737', 'D0'] |
| ['DO52737', 'DO46881'] |
| ['DO52738', 'D0'] |
| ['DO52738', 'DO33512'] |
| ['DO52739', 'DO48893'] |
| ['DO52740', 'D0'] |
| ['DO52742', 'D0'] |
| ['DO52743', 'D0'] |
| ['DO52743', 'DO48727'] |
| ['DO52744', 'D0'] |
| ['DO52745', 'D0'] |
| ['DO52747', 'D0'] |
| ['DO52751', 'D0'] |
| ['DO52752', 'DO218535'] |
| ['DO52756', 'D0'] |
| ['DO52758', 'D0'] |
| ['DO52760', 'D0'] |
| ['DO52763', 'D0'] |
| ['DO6362', 'D0'] |
| ['DO6398', 'DO46568'] |
| ['DO6432', 'D0'] |
| ['DO6934', 'DO52150'] |
| ['DO6934', 'DO52698'] |
| ['DO7084', 'D0'] |
| ['DO7084', 'DO27791'] |
| ['DO7112', 'D0'] |

**Supplementary Table 3.** Information of clusters formed by Louvain community detection (resolution = 1). The annotations (cancer types and genes) in these clusters are used as background for the Fischer exact test.

| **Cancer types** | **Genes** | **Avg mutation load** | **Num samples** |
| --- | --- | --- | --- |
| {'Lymph': 47, 'Liver': 30, 'Prost': 26, 'Eso': 19, 'Myeloid': 18, 'Breast': 14, 'Skin': 11, 'CNS': 10, 'Stomach': 7, 'Panc': 7, 'Kidney': 7, 'Biliary': 6, 'Ovary': 5, 'Bone': 4, 'None': 1} | {'BCL2': 160, 'TP53': 43, 'RP11-193H5.1': 33, 'TMSB4X': 25, 'AR': 18, 'PTEN': 12, 'SPOP': 12, 'RP11-32K4.1': 12, 'RP11-420N3.2': 12, 'Y_RNA': 12, 'MUC16': 11, 'TBL1XR1': 11, 'PIM1': 11, 'PDE4DIP': 11, 'MUC4': 11, 'RB1': 11, 'CTNNB1': 10, 'PRCC': 10, 'DDX6': 10, 'EZH2': 9, 'SGK1': 9, 'CTD-3006G17.2': 9, 'LINC00535': 9, 'ASXL2': 9, 'TRIM33': 9, 'LCP1': 9, 'KAT6A': 9, 'KCNJ5': 9, 'FOXA1': 8, 'CDK12': 8, 'RP11-17E2.2': 8, 'RPL23AP79': 8, 'snoU13': 8, 'NF1': 7, 'IKZF1': 7, 'ERBB4': 7, 'CNTNAP2': 7, 'AC079610.1': 7, 'MEF2C-AS1': 7, 'RP11-586K2.1': 7, 'BCR': 7, 'FAM135B': 6, 'CD209': 6, 'PTPRT': 6, 'CTNND2': 6, 'CREBBP': 6, 'STAT6': 6, 'DISC1FP1': 6, 'RNF219-AS1': 6, 'RP11-152L20.3': 6, 'FAT1': 6, 'LMNA': 6, 'PTK6': 6, 'WNK2': 6, 'CDH11': 5, 'CSMD3': 5, 'NOTCH2': 5, 'IL6ST': 5, 'RP11-541P9.3': 5, 'SETBP1': 5, 'BCL9': 5, 'GS1-256O22.5': 5, 'CTC-340A15.2': 5, 'TET2': 5, 'NRG1': 4, 'ANK1': 4, 'MACC1': 4, 'FAT3': 4, 'KMT2D': 4, 'ROBO2': 4, 'MED12': 4, 'ATM': 4, 'SMARCA4': 4, 'RP11-418J17.1': 4, 'RP11-624C23.1': 4, 'RP11-649A16.1': 4, 'APC': 4, 'CUL3': 4, 'CRNKL1': 4, 'SFRP4': 4, 'BAGE2': 4, 'RP11-3B12.1': 4, 'ZFHX4-AS1': 4, 'RMI2': 4, 'SPECC1': 4, 'EML4': 4, 'KIAA1549': 4, 'CDH10': 4, 'ZNF384': 4, 'ELL': 4, 'ASXL1': 4, 'IDH2': 4, 'DNMT3A': 4, 'GRIN2A': 3, 'AC108142.1': 3, 'RUNX1T1': 3, 'PAX5': 3, 'PREX2': 3, 'NRAS': 3, 'BCL11A': 3, 'BCL6': 3, 'BTG2': 3, 'H3F3A': 3, 'AC004053.1': 3, 'AC079613.1': 3, 'CTD-2307P3.1': 3, 'IL12A-AS1': 3, 'INHBA-AS1': 3, 'RP11-525K10.3': 3, 'GNA11': 3, 'AC007879.5': 3, 'AC097467.2': 3, 'LINC00395': 3, 'RP11-550P17.5': 3, 'RP11-624L4.1': 3, 'RP13-492C18.2': 3, 'RP3-399L15.3': 3, 'AF121898.3': 3, 'F11-AS1': 3, 'CARD11': 3, 'FOXO1': 3, 'SH2B3': 3, 'CTCF': 3, 'RNF43': 2, 'SMAD4': 2, 'PIK3CA': 2, 'BRAF': 2, 'PBX1': 2, 'SMAD2': 2, 'MALT1': 2, 'CTNNA2': 2, 'NFKBIE': 2, 'GRM3': 2, 'FAM47C': 2, 'ELK4': 2, 'CCND2': 2, 'BMP5': 2, 'MSH6': 2, 'CCND3': 2, 'IGHM': 2, 'FGFR1': 2, 'ZCCHC8': 2, 'CHD2': 2, 'NSD1': 2, 'PHOX2B': 2, 'DTX4': 2, 'RP11-804A23.2': 2, 'REL': 2, 'CREB1': 2, 'PDGFRB': 2, 'BCL11B': 2, 'LRP1B': 2, 'CDKN2A': 2, 'HOXC11': 2, 'ITGAV': 2, 'AACSP1': 2, 'AC012501.2': 2, 'AC108696.1': 2, 'AC144521.1': 2, 'AL133247.2': 2, 'BDNF-AS': 2, 'C1orf132': 2, 'CASC2': 2, 'CTBP1-AS2': 2, 'CTC-260E6.6': 2, 'GBA3': 2, 'GTPBP10': 2, 'GUCY1B2': 2, 'HCG18': 2, 'IFNG-AS1': 2, 'IGHV3OR16-13': 2, 'MKLN1-AS1': 2, 'PROX1-AS1': 2, 'RP11-115D19.1': 2, 'RP11-13J10.1': 2, 'RP11-342D14.1': 2, 'RP11-556E13.1': 2, 'RP11-665G4.1': 2, 'RP5-921G16.1': 2, 'FOXL2': 2, 'NPM1': 2, 'KDM6A': 2, 'HOXC13': 2, 'EPHA7': 2, 'AC004791.2': 2, 'AC007277.3': 2, 'AC016907.3': 2, 'AC027612.3': 2, 'AP000320.7': 2, 'CTD-2269F5.1': 2, 'EPHA1-AS1': 2, 'FLG-AS1': 2, 'LMCD1-AS1': 2, 'NPSR1-AS1': 2, 'RP11-431M7.2': 2, 'RP11-439L18.3': 2, 'RP11-446J8.1': 2, 'RP11-692D12.1': 2, 'RP11-707M1.1': 2, 'RP11-770E5.1': 2, 'RP11-820L6.1': 2, 'SCN4A': 2, 'SEC24B-AS1': 2, 'SNORA26': 2, 'TMEM200C': 2, 'TMPRSS4-AS1': 2, 'TPTE2P1': 2, 'TRAV27': 2, 'COL2A1': 2, 'AFF4': 2, 'GOPC': 2, 'CDH1': 2, 'AC004538.3': 2, 'AC007319.1': 2, 'AC009236.1': 2, 'CTC-525D6.1': 2, 'MCHR2-AS1': 2, 'RP11-154D6.1': 2, 'RP11-357C3.3': 2, 'RP11-679C8.2': 2, 'RP5-896L10.1': 2, 'AC091320.2': 2, 'FNDC3B': 2, 'AFF1': 2, 'EXT2': 2, 'BIRC3': 2, 'MAP3K13': 2, 'B2M': 2, 'XPO1': 2, 'MYC': 2, 'MITF': 2, 'EPAS1': 2, 'KDR': 2, 'CTB-57H20.1': 2, 'RP11-155G15.2': 2, 'PHF6': 2, 'ROS1': 2, 'SPEN': 2, 'U2AF1': 2, 'ITK': 2, 'TSC1': 2, 'None': 1, 'TFEB': 1, 'TGFBR2': 1, 'JAK3': 1, 'CAMTA1': 1, 'HMGN2P46': 1, 'MYD88': 1, 'PBRM1': 1, 'BRD3': 1, 'CBFB': 1, 'FBXW7': 1, 'FLI1': 1, 'PRKD1': 1, 'TCL1A': 1, 'AXIN1': 1, 'KEAP1': 1, 'ATF1': 1, 'SRGAP3': 1, 'JAZF1': 1, 'TRRAP': 1, 'KNSTRN': 1, 'PPP6C': 1, 'PPP2R2A': 1, 'WIF1': 1, 'EBF1': 1, 'EED': 1, 'CCNB1IP1': 1, 'FLT4': 1, 'HNF4A': 1, 'LINC00649': 1, 'TCF7L2': 1, 'KLK2': 1, 'STAG1': 1, 'ARID2': 1, 'LRRC4C': 1, 'TAL1': 1, 'BAX': 1, 'HOOK3': 1, 'DAXX': 1, 'SNORA63': 1, 'VHL': 1, 'PLEKHG6': 1, 'MTOR': 1, 'RPL22': 1, 'BMPR1A': 1, 'MYO5A': 1, 'LPP': 1, 'BCORL1': 1, 'KMT2A': 1, 'MAX': 1, 'KRAS': 1, 'AB015752.3': 1, 'AC002485.1': 1, 'AC009312.1': 1, 'AC012671.1': 1, 'AC013463.2': 1, 'AC018737.1': 1, 'AC018890.6': 1, 'AC024560.3': 1, 'AC079756.1': 1, 'AC091736.1': 1, 'AC104434.1': 1, 'AC108448.3': 1, 'AC129929.5': 1, 'AC131097.3': 1, 'ADD2': 1, 'AJ003147.9': 1, 'AJAP1': 1, 'AL117380.1': 1, 'AL138963.1': 1, 'AL590874.1': 1, 'ANKRD19P': 1, 'AP000640.2': 1, 'ARPC5': 1, 'B3GALTL': 1, 'BMS1P8': 1, 'BTAF1': 1, 'C18orf8': 1, 'CAMSAP3': 1, 'CCNL1': 1, 'CCR10': 1, 'CDPF1': 1, 'CLHC1': 1, 'CNGB3': 1, 'CNNM3': 1, 'COPA': 1, 'CST7': 1, 'CTD-2203K17.1': 1, 'CTH': 1, 'CYCS': 1, 'DCTN4': 1, 'DEAF1': 1, 'DGKI': 1, 'DNER': 1, 'DSTN': 1, 'EGF': 1, 'EMCN-IT3': 1, 'EOGT': 1, 'FAM210B': 1, 'FAM27E3': 1, 'FAM3B': 1, 'FAM86JP': 1, 'FZD3': 1, 'GCM1': 1, 'GGTA1P': 1, 'GLTSCR2': 1, 'GPR158-AS1': 1, 'GTF2A1': 1, 'HELZ2': 1, 'HS3ST1': 1, 'IGHD3OR15-3B': 1, 'IGLV1-51': 1, 'KCND2': 1, 'KCNRG': 1, 'LAMA2': 1, 'LIFR-AS1': 1, 'LRRIQ1': 1, 'MED15P9': 1, 'MEGF10': 1, 'MGAM': 1, 'MIR4472-1': 1, 'NABP1': 1, 'NCLN': 1, 'NDC80': 1, 'NFKBID': 1, 'NUFIP1': 1, 'OR2H1': 1, 'PARP4': 1, 'PCDHGA6': 1, 'PKD1L3': 1, 'PPP1R15A': 1, 'PRKCA': 1, 'RABGEF1': 1, 'RAPGEF4-AS1': 1, 'REXO1L1': 1, 'RHOU': 1, 'RN7SL139P': 1, 'RN7SL300P': 1, 'RNA5SP508': 1, 'RNU6-1163P': 1, 'RNU6-204P': 1, 'RNU6-439P': 1, 'RNU7-24P': 1, 'RP1-13P20.6': 1, 'RP1-167A14.2': 1, 'RP11-130F10.1': 1, 'RP11-167H9.4': 1, 'RP11-290F24.3': 1, 'RP11-356I2.4': 1, 'RP11-380P13.1': 1, 'RP11-438D8.2': 1, 'RP11-521M14.1': 1, 'RP11-57H12.5': 1, 'RP11-680B3.2': 1, 'RP11-693J15.4': 1, 'RP11-800A3.4': 1, 'RP11-846C15.2': 1, 'RP11-84A1.3': 1, 'RP11-933H2.4': 1, 'RP3-428L16.1': 1, 'RP4-605O3.4': 1, 'RP4-798P15.3': 1, 'SCARNA17': 1, 'SFXN1': 1, 'SIGLECL1': 1, 'SLC25A44': 1, 'SNHG11': 1, 'SNHG14': 1, 'SNX7': 1, 'SPATA3': 1, 'STXBP5-AS1': 1, 'SUSD2': 1, 'SVILP1': 1, 'THEMIS2': 1, 'TMEM161B-AS1': 1, 'TREML3P': 1, 'UCK1': 1, 'UGDH-AS1': 1, 'UHRF1': 1, 'UNC5B': 1, 'VPS37A': 1, 'WDR11-AS1': 1, 'Z93241.1': 1, 'ZC3H11A': 1, 'ZNF75D': 1, 'GNAS': 1, 'C16orf62': 1, 'ARID1A': 1, 'ZNF521': 1, 'KIT': 1, 'ERBB3': 1, 'KDM5A': 1, 'PRRX1': 1, 'IDH1': 1, 'AC004901.1': 1, 'AC009120.6': 1, 'AC015922.5': 1, 'AC024908.1': 1, 'AC061961.2': 1, 'AC091878.1': 1, 'AC097468.7': 1, 'AC102953.4': 1, 'ACE': 1, 'ADCY1': 1, 'ADPRHL2': 1, 'ADRBK2': 1, 'AF186996.1': 1, 'AL159977.1': 1, 'AL162511.1': 1, 'AL353791.1': 1, 'AL590726.1': 1, 'AL928742.2': 1, 'ANGPTL7': 1, 'ANKRD20A1': 1, 'ANKRD26P1': 1, 'ANKRD6': 1, 'AOC1': 1, 'AP000765.1': 1, 'AP001525.1': 1, 'AP002954.4': 1, 'ARHGEF26-AS1': 1, 'ARHGEF37': 1, 'BCORP1': 1, 'BRINP2': 1, 'C11orf84': 1, 'C2orf16': 1, 'C6': 1, 'CAB39L': 1, 'CASC8': 1, 'CCT6P3': 1, 'CDHR5': 1, 'CLRN1-AS1': 1, 'COL26A1': 1, 'CPNE4': 1, 'CSTF3-AS1': 1, 'CTB-186H2.2': 1, 'CTC-297N7.5': 1, 'CTD-2001E22.1': 1, 'CTD-2015H6.3': 1, 'CTD-2176I21.2': 1, 'CTD-2215L10.1': 1, 'CTNNA3': 1, 'CXXC1P1': 1, 'CXXC4': 1, 'DARC': 1, 'DLG2': 1, 'ELOVL2': 1, 'ENTPD3-AS1': 1, 'ERMP1': 1, 'FAM120AOS': 1, 'FAM153B': 1, 'FAM181A-AS1': 1, 'FAM184B': 1, 'FAM230B': 1, 'FETUB': 1, 'FNDC1': 1, 'FOLH1B': 1, 'FREM2': 1, 'GATAD2A': 1, 'GPR156': 1, 'GPR4': 1, 'GTF2H5': 1, 'HEATR6': 1, 'HEG1': 1, 'HHIPL1': 1, 'HMX3': 1, 'HTR5BP': 1, 'IGKV1D-37': 1, 'INTS4': 1, 'INTS4L1': 1, 'KCNMB3P1': 1, 'KIAA1257': 1, 'KIAA1683': 1, 'KIF25': 1, 'KIRREL3-AS3': 1, 'KLHL3': 1, 'LINC00467': 1, 'LINC00963': 1, 'LINC01088': 1, 'MIR31HG': 1, 'MIR548H4': 1, 'MIR596': 1, 'MMP8': 1, 'MSLN': 1, 'MTHFD2P1': 1, 'MTRNR2L5': 1, 'MTSS1L': 1, 'NELFA': 1, 'NRGN': 1, 'NT5DC4': 1, 'OR5D18': 1, 'OR8B8': 1, 'PASD1': 1, 'PCBP1-AS1': 1, 'PCDHB3': 1, 'PDE10A': 1, 'PEG10': 1, 'PEX7': 1, 'PI3': 1, 'PPFIA1': 1, 'PPP1R35': 1, 'PSTK': 1, 'RFTN1P1': 1, 'RHBDL3': 1, 'RN7SKP227': 1, 'RN7SL564P': 1, 'RN7SL7P': 1, 'RN7SL89P': 1, 'RNA5SP358': 1, 'RNA5SP403': 1, 'RNU1-59P': 1, 'RNU4-57P': 1, 'RNU6-102P': 1, 'RNU6-1047P': 1, 'RNU6-442P': 1, 'RNU6-52P': 1, 'RNU6-543P': 1, 'RNU6-656P': 1, 'RP1-240B8.3': 1, 'RP1-28O10.1': 1, 'RP1-65P5.1': 1, 'RP1-93I3.1': 1, 'RP11-100L22.2': 1, 'RP11-1017G21.4': 1, 'RP11-1084E5.1': 1, 'RP11-1103G16.1': 1, 'RP11-1129I3.1': 1, 'RP11-125B21.2': 1, 'RP11-1277A3.2': 1, 'RP11-13G14.4': 1, 'RP11-142C4.6': 1, 'RP11-144L1.4': 1, 'RP11-145A3.1': 1, 'RP11-158J3.2': 1, 'RP11-159L20.2': 1, 'RP11-166B2.8': 1, 'RP11-17A1.3': 1, 'RP11-231I13.2': 1, 'RP11-231P20.2': 1, 'RP11-23D24.2': 1, 'RP11-274B21.1': 1, 'RP11-280O1.2': 1, 'RP11-285J16.1': 1, 'RP11-324H6.5': 1, 'RP11-326E22.1': 1, 'RP11-343D2.11': 1, 'RP11-379F4.4': 1, 'RP11-431M7.3': 1, 'RP11-445F12.1': 1, 'RP11-454C18.2': 1, 'RP11-513G19.1': 1, 'RP11-526D8.7': 1, 'RP11-538P18.2': 1, 'RP11-551L14.1': 1, 'RP11-58B2.1': 1, 'RP11-702H23.4': 1, 'RP11-706C16.8': 1, 'RP11-742B18.1': 1, 'RP11-744N12.3': 1, 'RP11-752G15.9': 1, 'RP11-75N4.2': 1, 'RP11-788M5.3': 1, 'RP11-86L19.2': 1, 'RP11-978I15.10': 1, 'RP3-323P13.2': 1, 'RP4-630C24.3': 1, 'RP5-1121H13.4': 1, 'RP5-905H7.3': 1, 'SEC14L4': 1, 'SLAMF6': 1, 'SLC18A2': 1, 'SLC26A2': 1, 'SLC31A1': 1, 'SLC46A1': 1, 'SLC5A3': 1, 'SLC8A1': 1, 'SLC8A1-AS1': 1, 'SNAPC1': 1, 'SNORA51': 1, 'SNORD29': 1, 'SNRPD2': 1, 'SORL1': 1, 'SRD5A3-AS1': 1, 'SRGAP2B': 1, 'SSPO': 1, 'SUPT20H': 1, 'TCHH': 1, 'TFAP2B': 1, 'TGS1': 1, 'THSD1': 1, 'TMEM254-AS1': 1, 'TNS3': 1, 'TTN': 1, 'TYW1B': 1, 'USP15': 1, 'WDR74': 1, 'XRCC5': 1, 'ZFYVE21': 1, 'ZNF207': 1, 'ZNF527': 1, 'ZNF665': 1, 'ZNF844': 1, 'ZNRD1-AS1': 1, 'ZRANB2-AS1': 1, 'ZRANB2-AS2': 1, 'hsa-mir-490': 1, 'ABCC4': 1, 'AC004041.2': 1, 'AC004458.1': 1, 'AC005062.2': 1, 'AC005592.2': 1, 'AC006322.1': 1, 'AC008836.1': 1, 'AC009677.1': 1, 'AC012363.4': 1, 'AC016725.4': 1, 'AC073479.1': 1, 'AC079135.1': 1, 'AC079630.4': 1, 'AC083906.1': 1, 'AC092846.2': 1, 'AC093874.1': 1, 'AC096579.13': 1, 'AC098617.1': 1, 'AC138761.1': 1, 'ADAL': 1, 'AL139815.1': 1, 'AL359709.2': 1, 'ALDH9A1': 1, 'ANKRD12': 1, 'AP001597.1': 1, 'ARHGAP11B': 1, 'ARHGEF40': 1, 'ASNA1': 1, 'ATG5': 1, 'BPIFA4P': 1, 'C10orf12': 1, 'C17orf80': 1, 'CD2AP': 1, 'CDC42SE2': 1, 'CDH23': 1, 'CDKN2B-AS1': 1, 'CHIC1': 1, 'CNGA1': 1, 'COL24A1': 1, 'COL5A3': 1, 'CTB-49A3.2': 1, 'CTC-457E21.9': 1, 'CTD-2547L16.1': 1, 'CTD-3088G3.8': 1, 'CTTNBP2': 1, 'CYP4B1': 1, 'DCAF4L2': 1, 'DDX11-AS1': 1, 'DDX50': 1, 'DDX60': 1, 'DIO2-AS1': 1, 'DIRC3': 1, 'DOCK5': 1, 'DUOX2': 1, 'EFCAB1': 1, 'EGFEM1P': 1, 'EHHADH-AS1': 1, 'EIF4E': 1, 'EMILIN1': 1, 'EPSTI1': 1, 'ERICH1-AS1': 1, 'ESPNP': 1, 'ESRRAP2': 1, 'FAIM2': 1, 'FAM177A1': 1, 'FBN1': 1, 'FBXL5': 1, 'FBXO28': 1, 'FZD10-AS1': 1, 'GJD2': 1, 'GLUD1P2': 1, 'GPD2': 1, 'GPR133': 1, 'GPT': 1, 'GRID1': 1, 'GRIPAP1': 1, 'GUSBP1': 1, 'HCN4': 1, 'HEBP2': 1, 'HIST1H1A': 1, 'HMGCS1': 1, 'HNRNPA1P48': 1, 'IGFBP7-AS1': 1, 'IGHD3-10': 1, 'IGHV1-24': 1, 'IGKV1-27': 1, 'IGLV7-46': 1, 'ITGB2': 1, 'KB-1562D12.1': 1, 'KCNB2': 1, 'KCNV1': 1, 'KHDRBS3': 1, 'KIAA2022': 1, 'KLF11': 1, 'KNTC1': 1, 'LEF1-AS1': 1, 'LINC00189': 1, 'LMTK2': 1, 'LRP3': 1, 'LRRC49': 1, 'MAGI3': 1, 'MAMDC2': 1, 'MCC': 1, 'MIR184': 1, 'MIR3622A': 1, 'MIR496': 1, 'MLK7-AS1': 1, 'MRPL45P2': 1, 'MYO18A': 1, 'NALCN-AS1': 1, 'NOVA1-AS1': 1, 'OIT3': 1, 'PDE3A': 1, 'PICK1': 1, 'PIK3CG': 1, 'PPIP5K2': 1, 'PVRL4': 1, 'RAD1': 1, 'RAPGEFL1': 1, 'RGS18': 1, 'RN7SL170P': 1, 'RN7SL251P': 1, 'RN7SL357P': 1, 'RN7SL572P': 1, 'RN7SL600P': 1, 'RN7SL637P': 1, 'RN7SL649P': 1, 'RNA5SP116': 1, 'RNA5SP504': 1, 'RNA5SP62': 1, 'RNF217': 1, 'RNMT': 1, 'RNPC3': 1, 'RNU6-1070P': 1, 'RNU6-243P': 1, 'RNU6-741P': 1, 'RNU7-136P': 1, 'RNY4P27': 1, 'RP1-167F1.2': 1, 'RP11-1069G10.1': 1, 'RP11-150C16.1': 1, 'RP11-163M18.1': 1, 'RP11-175P19.2': 1, 'RP11-196E1.3': 1, 'RP11-33N16.3': 1, 'RP11-366F6.2': 1, 'RP11-368M16.3': 1, 'RP11-370I10.6': 1, 'RP11-385J1.2': 1, 'RP11-422J8.1': 1, 'RP11-423H2.3': 1, 'RP11-429B14.1': 1, 'RP11-435M3.2': 1, 'RP11-505P4.7': 1, 'RP11-519G16.3': 1, 'RP11-532F12.5': 1, 'RP11-54D18.3': 1, 'RP11-562L8.1': 1, 'RP11-572M11.4': 1, 'RP11-611E13.2': 1, 'RP11-634B7.4': 1, 'RP11-66D17.5': 1, 'RP11-678G14.2': 1, 'RP11-76N22.2': 1, 'RP11-804N13.1': 1, 'RP11-826N14.1': 1, 'RP11-89K10.1': 1, 'RP11-92C4.3': 1, 'RP11-93K22.6': 1, 'RP11-98D18.15': 1, 'RP3-340N1.5': 1, 'RP4-651E10.4': 1, 'RP4-694A7.4': 1, 'RP5-991G20.1': 1, 'RUNDC1': 1, 'SAGE1': 1, 'SCRN1': 1, 'SEPT14': 1, 'SHANK2': 1, 'SIGLEC6': 1, 'SLC10A7': 1, 'SLC16A10': 1, 'SLC25A32': 1, 'SLC25A38': 1, 'SLC30A2': 1, 'SLC7A14': 1, 'SLFN5': 1, 'SRD5A2': 1, 'STARD13': 1, 'SUCO': 1, 'TAOK3': 1, 'TCF21': 1, 'TENC1': 1, 'TEX26-AS1': 1, 'TMEM154': 1, 'TMEM38B': 1, 'TRAPPC3L': 1, 'TRBV6-8': 1, 'TRIM69': 1, 'U3': 1, 'U8': 1, 'UBE2Q2': 1, 'UFL1-AS1': 1, 'UMODL1': 1, 'UNG': 1, 'URB1': 1, 'UVRAG': 1, 'VNN2': 1, 'VSTM4': 1, 'VTI1B': 1, 'WDR5B': 1, 'Z95704.4': 1, 'ZBED3-AS1': 1, 'ZBED5-AS1': 1, 'ZBTB38': 1, 'ZNF106': 1, 'ZNF341': 1, 'ZNF815P': 1, 'ZNF827': 1, 'ABCA3': 1, 'AC092048.1': 1, 'ADAMTS9-AS2': 1, 'ATP2C1': 1, 'C19orf35': 1, 'CTC-297N7.11': 1, 'CTD-2014E2.5': 1, 'FARSB': 1, 'FCRL3': 1, 'INHBA': 1, 'KIAA0513': 1, 'KIF9-AS1': 1, 'LL09NC01-254D11.1': 1, 'MIR518D': 1, 'MIR526B': 1, 'NREP-AS1': 1, 'OR14A2': 1, 'PMS2CL': 1, 'RASA2': 1, 'RBFOX2': 1, 'RN7SL575P': 1, 'RN7SL659P': 1, 'RNA5SP36': 1, 'RNU4-65P': 1, 'RP1-91G5.3': 1, 'RP11-167N24.3': 1, 'RP11-550I24.2': 1, 'RP11-644F5.16': 1, 'RP11-657O9.1': 1, 'RP11-708B6.2': 1, 'RP4-735C1.4': 1, 'RP4-777D9.2': 1, 'RPA3-AS1': 1, 'SERPINB9': 1, 'SLC15A1': 1, 'SP100': 1, 'STARD4-AS1': 1, 'TPT1-AS1': 1, 'TRAPPC3': 1, 'TRBV6-5': 1, 'TSSC2': 1, 'ZNF37BP': 1, 'AC027612.2': 1, 'AC079305.8': 1, 'AC110086.1': 1, 'CTD-2021J15.1': 1, 'CTSS': 1, 'FAM154A': 1, 'FZD1': 1, 'KIF1A': 1, 'NHLH1': 1, 'P2RY2': 1, 'PNMA1': 1, 'RP11-508N22.8': 1, 'RP13-578N3.3': 1, 'SNORD2': 1, 'SPANXA2-OT1': 1, 'ZCCHC4': 1, 'H3F3B': 1, 'IGKJ5': 1, 'HLA-A': 1, 'BTG1': 1, 'SOCS1': 1, 'RALGDS': 1, 'FBXO11': 1, 'AKAP11': 1, 'WWTR1': 1, 'NOTCH1': 1, 'AC004485.3': 1, 'AC004862.6': 1, 'CRYM': 1, 'CTD-2194D22.1': 1, 'DNAJB7': 1, 'HELQ': 1, 'KCND1': 1, 'MIR378D1': 1, 'OTX2-AS1': 1, 'PPP1R26-AS1': 1, 'PROM2': 1, 'PUM1': 1, 'RAET1K': 1, 'RFESD': 1, 'RP11-184A2.3': 1, 'RP11-310I9.1': 1, 'RP11-492A10.1': 1, 'RP11-631F7.1': 1, 'RP11-731J8.2': 1, 'RP4-756H11.3': 1, 'SERPINB11': 1, 'SGK223': 1, 'SNORA31': 1, 'SPTBN4': 1, 'SULT1C2P1': 1, 'SYT4': 1, 'TG': 1, 'TMEM132B': 1, 'TNRC18': 1, 'TRPC5': 1, 'VWA3B': 1, 'ZBTB7C': 1, 'ZCCHC11': 1, 'ZNF20': 1, 'ZNF222': 1, 'AC093391.2': 1, 'AL626787.1': 1, 'AL672294.1': 1, 'ALOX12P2': 1, 'ARHGAP40': 1, 'CACNA1C': 1, 'CMAHP': 1, 'COPB1': 1, 'CTD-2058B24.2': 1, 'CYLC2': 1, 'DZIP1': 1, 'FSIP2': 1, 'ISM2': 1, 'PPP1R12C': 1, 'RNA5SP459': 1, 'RP11-81K13.1': 1, 'RP11-944L7.4': 1, 'SNORD81': 1, 'SUPT6H': 1, 'TM4SF1-AS1': 1, 'AC072062.1': 1, 'AC136188.1': 1, 'AC136932.2': 1, 'AC144450.1': 1, 'CTSL3P': 1, 'HLTF-AS1': 1, 'HTR3B': 1, 'NFYB': 1, 'NLGN4X': 1, 'NR2F2-AS1': 1, 'PRKY': 1, 'RP1-272J12.1': 1, 'RP11-122D10.1': 1, 'RP11-135J2.4': 1, 'RP11-482H16.1': 1, 'RP11-542K23.7': 1, 'STEAP2-AS1': 1, 'TRGC2': 1, 'UCHL1-AS1': 1, 'SF3B1': 1} | 9.19811321 | 212 |
| {'Panc': 27, 'Ovary': 5, 'Eso': 4, 'Breast': 3, 'Liver': 2, 'Head': 1, 'Skin': 1, 'Lymph': 1, 'Kidney': 1, 'Prost': 1} | {'TP53': 33, 'KRAS': 27, 'SMAD4': 6, 'CDKN2A': 4, 'ZFHX3': 3, 'SMARCA4': 3, 'PTPRT': 2, 'PRPF40B': 2, 'CSMD3': 1, 'TAL1': 1, 'PRRC2C': 1, 'PRRX1': 1, 'BRAF': 1, 'MEPCE': 1, 'PDE4B': 1, 'ID3': 1, 'CCND3': 1, 'P2RY8': 1, 'ZNF521': 1, 'HLA-A': 1, 'PABPC1': 1, 'PTEN': 1, 'HMCN1': 1, 'NANOS2': 1, 'RBM10': 1, 'PICALM': 1, 'KLK2': 1, 'HMGN2P46': 1, 'TGFBR2': 1, 'DCAF12L2': 1} | 2.2173913 | 46 |
| {'Panc': 27, 'Liver': 4, 'Breast': 3, 'Eso': 3, 'Biliary': 1, 'Head': 1, 'Skin': 1, 'Ovary': 1, 'Kidney': 1} | {'TP53': 30, 'KRAS': 29, 'CDKN2A': 6, 'SMAD4': 4, 'PBRM1': 2, 'ARID1A': 2, 'CTNNB1': 2, 'RBM10': 1, 'USP45': 1, 'PIK3CA': 1, 'AFF3': 1, 'MUC16': 1, 'PTPRT': 1, 'MED12': 1, 'SND1': 1, 'ANK1': 1, 'ZPLD1': 1, 'RUNX1T1': 1, 'ERBB2': 1, 'APC': 1, 'FLI1': 1, 'PRPF40B': 1, 'ZFHX3': 1} | 2.16666667 | 42 |
| {'Skin': 18, 'Liver': 5, 'Breast': 3, 'Eso': 3, 'Head': 2, 'Biliary': 2, 'Lymph': 2, 'Panc': 2, 'Ovary': 1, 'CNS': 1, 'Myeloid': 1} | {'BRAF': 13, 'MUC16': 11, 'CDKN2A': 9, 'TP53': 8, 'NRAS': 7, 'PIK3CA': 6, 'ERBB4': 4, 'PTPRT': 4, 'PBX1': 3, 'RUNX1T1': 3, 'GRIN2A': 3, 'IDH1': 3, 'TMSB4X': 3, 'CTNNB1': 3, 'TERT': 2, 'PREX2': 2, 'EPHA7': 2, 'CNTNAP2': 2, 'KRAS': 2, 'MAF': 2, 'KMT2D': 2, 'SF3B1': 1, 'GATA3': 1, 'TGFBR2': 1, 'CUL3': 1, 'PGM5P2': 1, 'CCND2': 1, 'CD28': 1, 'KLK2': 1, 'TRPM1': 1, 'PTPRB': 1, 'MECOM': 1, 'AMER1': 1, 'KNSTRN': 1, 'ERCC4': 1, 'SETBP1': 1, 'SMAD2': 1, 'GRM3': 1, 'POT1': 1, 'BCL10': 1, 'POU2AF1': 1, 'ATF1': 1, 'TCL1A': 1, 'AXIN1': 1, 'USP6': 1, 'PTPRD': 1, 'ZFHX3': 1, 'RNF43': 1, 'LIFR': 1, 'MACC1': 1, 'DDX46': 1, 'CDH10': 1, 'MTOR': 1, 'BTG1': 1, 'BCL6': 1, 'SGK1': 1, 'SMARCA4': 1, 'FBXO11': 1, 'CCR7': 1, 'AR': 1} | 3.325 | 40 |
| {'Panc': 23, 'Liver': 6, 'Ovary': 4, 'Kidney': 2, 'Prost': 2, 'Stomach': 1, 'Breast': 1, 'Myeloid': 1} | {'TP53': 24, 'KRAS': 23, 'KMT2C': 5, 'CTNNB1': 4, 'CTC-340A15.2': 4, 'AF121898.3': 3, 'RNF219-AS1': 3, 'RP11-586K2.1': 3, 'RP11-679C8.2': 3, 'hsa-mir-490': 3, 'VAV1': 3, 'DGCR8': 3, 'NF2': 3, 'STAG2': 3, 'CDH11': 3, 'ZFHX3': 3, 'LEF1': 3, 'CHST11': 2, 'ACVR2A': 2, 'PREX2': 2, 'VHL': 2, 'GATA3': 2, 'ARHGAP35': 2, 'EIF4A2': 2, 'KAT6A': 2, 'AC144521.1': 2, 'DISC1FP1': 2, 'RP11-274B21.1': 2, 'RP11-382A20.4': 2, 'RP11-420N3.2': 2, 'SNHG14': 2, 'ROBO2': 2, 'KLF6': 2, 'RBM10': 2, 'CDKN2A': 1, 'DDR2': 1, 'FAT4': 1, 'RP11-1129I3.1': 1, 'NONO': 1, 'FAM135B': 1, 'RN7SKP104': 1, 'KIT': 1, 'A2ML1-AS1': 1, 'AC000370.2': 1, 'AC004460.1': 1, 'AC004538.3': 1, 'AC004673.1': 1, 'AC005592.2': 1, 'AC007128.1': 1, 'AC007970.1': 1, 'AC009950.2': 1, 'AC016723.4': 1, 'AC092965.1': 1, 'AC105245.1': 1, 'AC108142.1': 1, 'AC126177.1': 1, 'ACO1': 1, 'ADAM5': 1, 'AE000661.37': 1, 'AL354806.1': 1, 'AL512640.1': 1, 'AP001596.6': 1, 'ARSK': 1, 'ATG16L1': 1, 'BAI3': 1, 'BEND4': 1, 'CASKIN1': 1, 'CCT6P3': 1, 'CDC20B': 1, 'COL6A1': 1, 'COL6A5': 1, 'CRYGEP': 1, 'CSDE1': 1, 'CSMD1': 1, 'CTB-118P15.2': 1, 'CTBS': 1, 'CTC-457E21.6': 1, 'CTD-2288O8.1': 1, 'CTD-2307P3.1': 1, 'CTD-3006G17.2': 1, 'DCST2': 1, 'DGKE': 1, 'DNAJB9': 1, 'DUT': 1, 'EMCN-IT3': 1, 'EMR4P': 1, 'FAM179A': 1, 'FAM84B': 1, 'FLG-AS1': 1, 'FNIP2': 1, 'FOLH1B': 1, 'FREM2': 1, 'GABRB1': 1, 'GBA3': 1, 'GDF7': 1, 'GFRAL': 1, 'GRM6': 1, 'GRPEL1': 1, 'HLA-AS1': 1, 'HPN-AS1': 1, 'HTR5BP': 1, 'IGHV2-5': 1, 'IL1RAP': 1, 'IL21R-AS1': 1, 'ILDR2': 1, 'JAG2': 1, 'KCNC1': 1, 'LINC00189': 1, 'LINC00475': 1, 'LINC00634': 1, 'LLNLF-65H9.1': 1, 'LRRC37A16P': 1, 'MEF2C-AS1': 1, 'MIR1268A': 1, 'MIR4431': 1, 'MIR4454': 1, 'MTMR9LP': 1, 'MTRNR2L7': 1, 'Metazoa_SRP': 1, 'NALCN-AS1': 1, 'NFATC3': 1, 'NPAS2': 1, 'NPSR1-AS1': 1, 'NTF4': 1, 'NUP188': 1, 'OR52M1': 1, 'OTX2-AS1': 1, 'PAIP2B': 1, 'PCDHA5': 1, 'PKD1L1': 1, 'PLEKHA8P1': 1, 'PNMA5': 1, 'RASGRP1': 1, 'RN7SKP149': 1, 'RN7SL278P': 1, 'RN7SL33P': 1, 'RN7SL644P': 1, 'RN7SL865P': 1, 'RNA5SP24': 1, 'RNA5SP474': 1, 'RNF215': 1, 'RNU2-54P': 1, 'RNU6-133P': 1, 'RNU6-227P': 1, 'RNU6-533P': 1, 'RNU7-174P': 1, 'RP1-23K20.2': 1, 'RP1-261D10.2': 1, 'RP1-272J12.1': 1, 'RP11-103J8.1': 1, 'RP11-114H24.5': 1, 'RP11-13J10.1': 1, 'RP11-152P17.2': 1, 'RP11-15B17.1': 1, 'RP11-1L12.3': 1, 'RP11-230B22.1': 1, 'RP11-231I13.2': 1, 'RP11-284M14.1': 1, 'RP11-286H14.4': 1, 'RP11-313J2.1': 1, 'RP11-333E1.1': 1, 'RP11-33N16.3': 1, 'RP11-368J21.3': 1, 'RP11-402C9.1': 1, 'RP11-428C19.4': 1, 'RP11-562L8.1': 1, 'RP11-572M11.4': 1, 'RP11-624C23.1': 1, 'RP11-634B7.4': 1, 'RP11-680F20.9': 1, 'RP11-707M1.1': 1, 'RP11-708B6.2': 1, 'RP11-742B18.1': 1, 'RP11-770E5.1': 1, 'RP11-77K12.5': 1, 'RP11-804A23.1': 1, 'RP11-867G2.8': 1, 'RP11-87M18.2': 1, 'RP11-89K10.1': 1, 'RP4-724E13.2': 1, 'RP4-755D9.1': 1, 'RP5-1091N2.9': 1, 'SCN10A': 1, 'SCRG1': 1, 'SEC63': 1, 'SEMA5A': 1, 'SHANK3': 1, 'SIGLEC9': 1, 'SLAIN2': 1, 'SLC46A1': 1, 'SMARCA2': 1, 'SPHK1': 1, 'SRGAP2-AS1': 1, 'SSPO': 1, 'STEAP2-AS1': 1, 'SUGT1P3': 1, 'SWT1': 1, 'TECTA': 1, 'TFAP2C': 1, 'TLE3': 1, 'TLR8-AS1': 1, 'TMEM230': 1, 'TOP3B': 1, 'TRBV28': 1, 'TRIM3': 1, 'TRPM6': 1, 'TSPAN32': 1, 'TYW1B': 1, 'U3': 1, 'USP2-AS1': 1, 'WDR83OS': 1, 'WSCD1': 1, 'XKR8': 1, 'Y_RNA': 1, 'ZFHX4': 1, 'ZFHX4-AS1': 1, 'snoU13': 1, 'PIK3CA': 1, 'DCAF12L2': 1, 'SDHA': 1, 'STAT5B': 1, 'BRAF': 1, 'PTPRT': 1, 'SMAD4': 1, 'CD209': 1} | 8.025 | 40 |
| {'Lymph': 9, 'Panc': 8, 'Eso': 4, 'Biliary': 2, 'Liver': 2, 'Ovary': 2, 'Myeloid': 1} | {'TP53': 24, 'KRAS': 9, 'MYC': 6, 'RHOA': 3, 'PIM1': 3, 'ID3': 2, 'BCL6': 2, 'SGK1': 2, 'PRKAR1A': 2, 'MECOM': 2, 'ARID1A': 1, 'PBX1': 1, 'U2AF1': 1, 'SMARCA4': 1, 'FBXO11': 1, 'ZFHX3': 1, 'PHOX2B': 1, 'SOCS1': 1, 'KIAA1549': 1, 'TMSB4X': 1, 'CDH10': 1, 'XPO1': 1, 'PABPC1': 1, 'JAK3': 1, 'LRP1B': 1, 'FAT4': 1, 'ATM': 1, 'CSMD3': 1} | 2.60714286 | 28 |
| {'Liver': 11, 'Panc': 4, 'Ovary': 4, 'Skin': 2, 'CNS': 2, 'Eso': 2, 'Breast': 1, 'Prost': 1} | {'CTNNB1': 17, 'TP53': 11, 'ARID2': 8, 'CTNNA2': 7, 'ELF3': 5, 'KDM5A': 5, 'ALDH2': 5, 'HSP90AA1': 5, 'USP8': 5, 'COL1A1': 5, 'PBRM1': 5, 'TET2': 5, 'CDH10': 5, 'SGK1': 5, 'KIAA1549': 5, 'EIF1AX': 5, 'STAG2': 5, 'TCF12': 5, 'TMPRSS2': 5, 'KRAS': 4, 'MUC16': 2, 'PREX2': 2, 'DDX3X': 2, 'PBX1': 1, 'CCND2': 1, 'PTPRB': 1, 'CRNKL1': 1, 'MLH1': 1, 'EZH2': 1, 'PIK3CA': 1, 'ROBO2': 1, 'RP11-1103G16.1': 1, 'SMARCA4': 1, 'AFF3': 1, 'ARID1A': 1} | 5.18518519 | 27 |
| {'Panc': 9, 'Eso': 7, 'Breast': 3, 'Lymph': 2, 'Stomach': 1} | {'TP53': 18, 'GNAS': 7, 'KRAS': 7, 'CDKN2A': 6, 'SMARCA4': 2, 'MYC': 2, 'RPL10': 1, 'CARD11': 1, 'BCL9': 1, 'HMGN2P46': 1, 'CD209': 1, 'MUC16': 1, 'PTPN13': 1, 'RPL22': 1, 'LARP4B': 1, 'BCL11B': 1, 'MYO5A': 1, 'LYL1': 1, 'ASXL2': 1, 'ACVR2A': 1, 'N4BP2': 1, 'MLLT3': 1, 'DDX3X': 1, 'NONO': 1, 'NRAS': 1, 'DCAF12L2': 1, 'CDH11': 1, 'BCL11A': 1, 'SMAD4': 1, 'PIK3CA': 1, 'PREX2': 1, 'MAX': 1, 'H3F3A': 1} | 3.13636364 | 22 |
| {'Breast': 8, 'Panc': 2, 'Eso': 2, 'Stomach': 1, 'Ovary': 1, 'Liver': 1, 'Prost': 1, 'Bone': 1} | {'PIK3CA': 11, 'TP53': 8, 'GNAS': 3, 'LEPROTL1': 2, 'KRAS': 2, 'PTEN': 2, 'CTNNB1': 2, 'GATA3': 1, 'ERBB3': 1, 'CSMD3': 1, 'RPL22': 1, 'LARP4B': 1, 'VTI1A': 1, 'ATM': 1, 'KDM5A': 1, 'SIX1': 1, 'BCL11B': 1, 'CIITA': 1, 'SPECC1': 1, 'PPP2R1A': 1, 'ACVR2A': 1, 'N4BP2': 1, 'PHOX2B': 1, 'IL6ST': 1, 'HNRNPA2B1': 1, 'BRAF': 1, 'HOOK3': 1, 'EXT1': 1, 'AR': 1, 'MLLT3': 1, 'CTNNA2': 1, 'APC': 1, 'BAX': 1, 'PTPRT': 1, 'RB1': 1, 'CD209': 1} | 3.47058824 | 17 |
| {'Lymph': 16, 'Kidney': 1} | {'BCL2': 36, 'PIM1': 12, 'TMSB4X': 12, 'MYC': 6, 'CREBBP': 4, 'MYD88': 4, 'MUC16': 3, 'SOCS1': 3, 'BCR': 2, 'CD79B': 2, 'BTG2': 2, 'BCL6': 2, 'SGK1': 2, 'ID3': 1, 'RPL10': 1, 'B2M': 1, 'LRP1B': 1, 'IGKJ3': 1} | 5.58823529 | 17 |
| {'Panc': 6, 'Breast': 4, 'Eso': 3, 'Stomach': 1, 'Prost': 1, 'Ovary': 1} | {'SF3B1': 9, 'TP53': 8, 'KRAS': 6, 'AKT1': 2, 'NRG1': 2, 'KLK2': 2, 'MB21D2': 2, 'KIT': 2, 'PDGFRB': 2, 'PIK3CA': 1, 'SDHA': 1, 'ROBO2': 1} | 2.375 | 16 |
| {'Panc': 9, 'Liver': 2, 'Eso': 2, 'Stomach': 1, 'Lymph': 1} | {'TP53': 14, 'KRAS': 9, 'BCL2': 9, 'U2AF1': 2, 'PDE4DIP': 1, 'FLT4': 1, 'APC': 1, 'PTEN': 1} | 2.53333333 | 15 |
| {'Skin': 8, 'Liver': 3, 'Panc': 2, 'Stomach': 1, 'Lymph': 1} | {'NRAS': 8, 'B2M': 5, 'MUC16': 5, 'CTNNB1': 3, 'ATM': 3, 'MECOM': 3, 'PTPRT': 3, 'RGS7': 2, 'ARHGEF12': 2, 'TP53': 2, 'CDKN2A': 2, 'DCC': 2, 'ERBB4': 2, 'CUL3': 2, 'RBM15': 1, 'LIFR': 1, 'AC016725.4': 1, 'BCL10': 1, 'TCL1A': 1, 'FAM135B': 1, 'PPP6C': 1, 'NBEA': 1, 'TCF7L2': 1, 'HMGN2P46': 1, 'SMARCA4': 1, 'DDR2': 1, 'ELK4': 1, 'SIX1': 1, 'SMAD2': 1, 'NONO': 1, 'DIAPH2-AS1': 1, 'PDE4DIP': 1, 'POU2AF1': 1, 'SRGAP3': 1, 'PTEN': 1, 'A1CF': 1, 'ERCC4': 1, 'CSMD3': 1, 'GRIN2A': 1, 'SETBP1': 1, 'KEAP1': 1, 'CTNNA2': 1, 'LRP1B': 1, 'CDH10': 1, 'CSF1R': 1, 'JAZF1': 1, 'PTPRD': 1, 'IBTK': 1, 'KRAS': 1, 'ATRX': 1, 'PIM1': 1, 'TMSB4X': 1} | 5.46666667 | 15 |
| {'Prost': 3, 'Liver': 2, 'Eso': 2, 'Head': 1, 'Breast': 1, 'Skin': 1, 'Lymph': 1, 'Panc': 1} | {'TP53': 10, 'PIK3CA': 4, 'RUNX1T1': 4, 'KRAS': 2, 'FAT4': 2, 'SOCS1': 1, 'SMARCA4': 1, 'IDH1': 1, 'TGFBR2': 1, 'TMSB4X': 1, 'B2M': 1, 'LYL1': 1} | 2.41666667 | 12 |
| {'Liver': 4, 'Panc': 3, 'Stomach': 2, 'Breast': 1, 'Skin': 1, 'Prost': 1} | {'TP53': 10, 'CTNNB1': 3, 'MUC16': 3, 'KRAS': 3, 'CNTNAP2': 2, 'NUP214': 2, 'TBL1XR1': 1, 'PTPRC': 1, 'RGS7': 1, 'FAT3': 1, 'PAFAH1B2': 1, 'NBEA': 1, 'PRKD1': 1, 'GRIN2A': 1, 'PTPRT': 1, 'MLH1': 1, 'ROBO2': 1, 'MECOM': 1, 'GRM3': 1, 'KIAA1549': 1, 'PREX2': 1, 'CSMD3': 1, 'SLC24A5': 1, 'PTPN13': 1, 'FAT4': 1, 'HLA-A': 1} | 3.58333333 | 12 |
| {'Eso': 3, 'Panc': 3, 'Biliary': 1, 'Skin': 1, 'Ovary': 1} | {'TP53': 9, 'KRAS': 3, 'MUC16': 2, 'ARID1A': 1, 'PBX1': 1, 'PTPRC': 1, 'KMT2D': 1, 'TCL1A': 1, 'CTNNA2': 1, 'CD28': 1, 'CRNKL1': 1, 'PTPRT': 1, 'CDH10': 1, 'CSF1R': 1, 'POT1': 1, 'CNTNAP2': 1, 'CDKN2A': 1, 'RP11-231I13.2': 1, 'REL': 1, 'ERBB2': 1, 'SETBP1': 1, 'APC': 1, 'PTEN': 1, 'BAX': 1} | 3.88888889 | 9 |
| {'Skin': 8} | {'BRAF': 8, 'MUC16': 8, 'PTPRT': 5, 'PTPRB': 4, 'ERBB4': 3, 'CSMD3': 1, 'TMEM121': 1, 'RBM15': 1, 'RGS7': 1, 'GRIN2A': 1, 'MACC1': 1, 'SND1': 1, 'CDKN2A': 1, 'TP53': 1, 'TM9SF4': 1, 'PAFAH1B2': 1, 'ROBO2': 1, 'ELK4': 1, 'A1CF': 1, 'LRP1B': 1, 'NFKBIE': 1, 'CNTNAP2': 1, 'PREX2': 1, 'PAX5': 1, 'AMER1': 1, 'SMARCD2': 1, 'KMT2D': 1, 'KLK2': 1, 'CUL3': 1, 'MECOM': 1, 'TRRAP': 1, 'PTEN': 1} | 6.875 | 8 |
| {'Panc': 2, 'CNS': 2, 'Liver': 2} | {'CTNNB1': 4, 'SMARCA4': 3, 'GNAS': 2, 'TP53': 2, 'CCR7': 1, 'CSMD3': 1, 'BMPR1A': 1, 'LEF1': 1, 'EXT1': 1, 'CTA-85E5.10': 1} | 2.83333333 | 6 |
| {'Eso': 3, 'Breast': 1, 'Head': 1, 'Panc': 1} | {'TP53': 5, 'USP6': 2, 'ERBB2': 1, 'LRP1B': 1, 'KRAS': 1} | 1.66666667 | 6 |
| {'Panc': 2, 'Breast': 1, 'Ovary': 1, 'Eso': 1, 'Bone': 1} | {'TP53': 7, 'KRAS': 2, 'CTNNA2': 1} | 1.66666667 | 6 |
| {'Liver': 4, 'Panc': 1, 'Prost': 1} | {'CSMD3': 9, 'TP53': 8, 'CTNNB1': 7, 'NBEA': 5, 'CTNNA2': 5, 'TBL1XR1': 5, 'MLLT3': 5, 'SNX29': 5, 'RABEP1': 5, 'SGK1': 4, 'PMS2': 2, 'KRAS': 1, 'CEP170P1': 1, 'RUSC2': 1} | 10.5 | 6 |
| {'Liver': 5, 'Prost': 1} | {'CEBPA': 7, 'SNX29': 5, 'CTNNB1': 4, 'ARID1A': 3, 'RB1': 3, 'SOX21': 3, 'KTN1': 3, 'AKT1': 3, 'MAF': 3, 'ASXL2': 3, 'CTNNA2': 3, 'ERBB4': 3, 'TGFBR2': 3, 'FAT4': 3, 'NRG1': 3, 'CSMD3': 3, 'MLLT3': 3, 'STIL': 3, 'PWWP2A': 2, 'MACC1': 2, 'QKI': 2, 'PICALM': 1, 'TP53': 1, 'SF3B1': 1, 'MEF2C-AS1': 1} | 11.8333333 | 6 |
| {'CNS': 3, 'Breast': 1, 'Biliary': 1} | {'SMO': 3, 'PIK3CA': 2, 'TRRAP': 2, 'RPL22': 1} | 1.6 | 5 |
| {'Breast': 5} | {'GATA3': 5, 'PIK3CA': 4, 'CBFB': 1, 'SMARCA4': 1} | 2.2 | 5 |
| {'Panc': 2, 'Eso': 2, 'Myeloid': 1} | {'KRAS': 2, 'SMAD4': 2, 'ERBB4': 2, 'MUC16': 2} | 1.6 | 5 |
| {'Panc': 4, 'Eso': 1} | {'KRAS': 5, 'CDKN2A': 5, 'TP53': 3, 'U2AF1': 1} | 2.8 | 5 |
| {'Eso': 3, 'Liver': 1} | {'CDH11': 3, 'TP53': 2, 'BMP5': 2, 'MECOM': 1, 'ANK1': 1, 'FOXO1': 1} | 2.5 | 4 |
| {'Lymph': 3} | {'CARD11': 2, 'EZH2': 2, 'BCL2': 2} | 2 | 3 |
| {'Lymph': 3} | {'TMSB4X': 4, 'SGK1': 2, 'BTG1': 1, 'P2RY8': 1} | 2.66666667 | 3 |
| {'Lymph': 3} | {'BCL2': 7, 'MYD88': 3, 'EZH2': 2, 'ZFHX3': 1} | 4.33333333 | 3 |
| {'Biliary': 1, 'Head': 1, 'CNS': 1} | {'TP53': 2, 'IKZF1': 2, 'ARID1A': 1} | 1.66666667 | 3 |
| {'Eso': 2, 'Panc': 1} | {'TP53': 4, 'ARID1A': 2, 'KRAS': 1} | 2.33333333 | 3 |
| {'Lymph': 2, 'Panc': 1} | {'SETD2': 2, 'NOTCH1': 2} | 1.33333333 | 3 |
| {'Breast': 2} | {'PTEN': 2, 'SF3B1': 1, 'PIK3CA': 1, 'DCAF12L2': 1} | 2.5 | 2 |
| {'Skin': 1, 'Liver': 1} | {'CD209': 2, 'CNTNAP2': 1} | 1.5 | 2 |
| {'Skin': 1, 'Liver': 1} | {'DDR2': 2, 'PTPRT': 1} | 1.5 | 2 |
| {'Panc': 2} | {'BRAF': 2, 'TP53': 1, 'FAT4': 1} | 2 | 2 |
| {'Panc': 2} | {'KRAS': 2, 'CDKN2A': 2, 'TP53': 1} | 2.5 | 2 |
| {'Kidney': 2} | {'VHL': 2, 'H3F3A': 1} | 1.5 | 2 |
| {'Panc': 1, 'Eso': 1} | {'GNAS': 2, 'USP6': 1} | 1.5 | 2 |
| {'Panc': 2} | {'MEN1': 2, 'PTEN': 1} | 1.5 | 2 |
| {'Biliary': 1, 'Prost': 1} | {'SMARCA4': 2, 'ACVR2A': 2, 'EXT1': 2, 'TP53': 1, 'GNAS': 1, 'RUNX1T1': 1, 'RALGDS': 1, 'LARP4B': 1, 'PTEN': 1, 'KDM5A': 1, 'MYO5A': 1, 'CIITA': 1, 'SPECC1': 1, 'STAT5B': 1, 'PPP2R1A': 1, 'ASXL2': 1, 'REL': 1, 'STAG1': 1, 'WWTR1': 1, 'LPP': 1, 'LEF1': 1, 'HNRNPA2B1': 1, 'CSMD3': 1, 'DDX3X': 1, 'BCORL1': 1} | 14 | 2 |
| {'Lymph': 2} | {'SOCS1': 4, 'BTG2': 1, 'BTG1': 1, 'ROBO2': 1, 'EBF1': 1, 'HLA-A': 1, 'PIM1': 1, 'TMSB4X': 1, 'VTI1A': 1} | 6 | 2 |
| {'Lymph': 2} | {'BCL2': 9, 'TMSB4X': 3, 'STAT6': 1, 'HMGN2P46': 1, 'ZFHX3': 1, 'TP53': 1, 'SGK1': 1, 'FAM135B': 1} | 9 | 2 |

**Supplementary Table 4.** Information of clusters formed by Louvain community detection (resolution = 1.5).

| **Cancer types** | **Genes** | **Avg mutation load** | **Num samples** |
| --- | --- | --- | --- |
| {'Lymph': 38, 'Prost': 19, 'Myeloid': 16, 'Liver': 13, 'Skin': 10, 'Breast': 8, 'Eso': 8, 'CNS': 7, 'Stomach': 5, 'Ovary': 5, 'Panc': 4, 'Kidney': 4, 'Biliary': 3, 'Bone': 2, 'None': 1} | {'BCL2': 126, 'RP11-193H5.1': 33, 'TP53': 24, 'TMSB4X': 20, 'MUC16': 10, 'PIM1': 10, 'SGK1': 9, 'ASXL2': 9, 'NF1': 7, 'CNTNAP2': 7, 'EZH2': 7, 'ERBB4': 6, 'PTPRT': 6, 'PTEN': 6, 'CREBBP': 6, 'AR': 6, 'FAT1': 6, 'WNK2': 6, 'FAM135B': 5, 'CD209': 5, 'CSMD3': 5, 'NOTCH2': 5, 'TET2': 5, 'CDH11': 4, 'CTNND2': 4, 'MACC1': 4, 'FAT3': 4, 'KMT2D': 4, 'STAT6': 4, 'MED12': 4, 'ATM': 4, 'SMARCA4': 4, 'RP11-418J17.1': 4, 'RMI2': 4, 'SPECC1': 4, 'EML4': 4, 'KIAA1549': 4, 'CDH10': 4, 'ZNF384': 4, 'ELL': 4, 'ASXL1': 4, 'IDH2': 4, 'DNMT3A': 4, 'GRIN2A': 3, 'NRG1': 3, 'PAX5': 3, 'NRAS': 3, 'BCL11A': 3, 'BCL6': 3, 'SPOP': 3, 'BTG2': 3, 'Y_RNA': 3, 'ROBO2': 3, 'CARD11': 3, 'SH2B3': 3, 'AC108142.1': 2, 'RNF43': 2, 'BRAF': 2, 'PBX1': 2, 'SMAD2': 2, 'MALT1': 2, 'CTNNA2': 2, 'NFKBIE': 2, 'GRM3': 2, 'RUNX1T1': 2, 'FAM47C': 2, 'ELK4': 2, 'CCND2': 2, 'BMP5': 2, 'PREX2': 2, 'MSH6': 2, 'CCND3': 2, 'FGFR1': 2, 'ZCCHC8': 2, 'CHD2': 2, 'NSD1': 2, 'PHOX2B': 2, 'DTX4': 2, 'REL': 2, 'CREB1': 2, 'PDGFRB': 2, 'BCL11B': 2, 'LRP1B': 2, 'SETBP1': 2, 'PDE4DIP': 2, 'H3F3A': 2, 'HOXC11': 2, 'ITGAV': 2, 'AC079610.1': 2, 'C1orf132': 2, 'CASC2': 2, 'DISC1FP1': 2, 'GS1-256O22.5': 2, 'LINC00535': 2, 'MEF2C-AS1': 2, 'RNF219-AS1': 2, 'RP11-32K4.1': 2, 'RP11-420N3.2': 2, 'RP11-624C23.1': 2, 'RP5-921G16.1': 2, 'APC': 2, 'FOXL2': 2, 'COL2A1': 2, 'AFF4': 2, 'GOPC': 2, 'CDH1': 2, 'AFF1': 2, 'EXT2': 2, 'KCNJ5': 2, 'BIRC3': 2, 'MAP3K13': 2, 'B2M': 2, 'FOXO1': 2, 'MYC': 2, 'MITF': 2, 'EPAS1': 2, 'KDR': 2, 'PHF6': 2, 'ROS1': 2, 'SPEN': 2, 'U2AF1': 2, 'MUC4': 2, 'ITK': 2, 'TSC1': 2, 'None': 1, 'TFEB': 1, 'CAMTA1': 1, 'HMGN2P46': 1, 'MYD88': 1, 'PBRM1': 1, 'BRD3': 1, 'CBFB': 1, 'FBXW7': 1, 'IKZF1': 1, 'PIK3CA': 1, 'PRKD1': 1, 'TCL1A': 1, 'AXIN1': 1, 'KEAP1': 1, 'ATF1': 1, 'SRGAP3': 1, 'JAZF1': 1, 'ANK1': 1, 'TRRAP': 1, 'KNSTRN': 1, 'PPP6C': 1, 'PPP2R2A': 1, 'WIF1': 1, 'IGHM': 1, 'EBF1': 1, 'EED': 1, 'CCNB1IP1': 1, 'FLT4': 1, 'HNF4A': 1, 'LINC00649': 1, 'LRRC4C': 1, 'TAL1': 1, 'BAX': 1, 'HOOK3': 1, 'DAXX': 1, 'SNORA63': 1, 'VHL': 1, 'PLEKHG6': 1, 'RPL22': 1, 'BMPR1A': 1, 'MYO5A': 1, 'LPP': 1, 'IL6ST': 1, 'BCORL1': 1, 'KMT2A': 1, 'KRAS': 1, 'CDKN2A': 1, 'AACSP1': 1, 'AB015752.3': 1, 'AC002485.1': 1, 'AC004053.1': 1, 'AC009312.1': 1, 'AC012501.2': 1, 'AC012671.1': 1, 'AC013463.2': 1, 'AC018737.1': 1, 'AC018890.6': 1, 'AC024560.3': 1, 'AC079613.1': 1, 'AC079756.1': 1, 'AC091736.1': 1, 'AC104434.1': 1, 'AC108448.3': 1, 'AC108696.1': 1, 'AC129929.5': 1, 'AC131097.3': 1, 'AC144521.1': 1, 'ADD2': 1, 'AJ003147.9': 1, 'AJAP1': 1, 'AL117380.1': 1, 'AL133247.2': 1, 'AL138963.1': 1, 'AL590874.1': 1, 'ANKRD19P': 1, 'AP000640.2': 1, 'ARPC5': 1, 'B3GALTL': 1, 'BDNF-AS': 1, 'BMS1P8': 1, 'BTAF1': 1, 'C18orf8': 1, 'CAMSAP3': 1, 'CCNL1': 1, 'CCR10': 1, 'CDPF1': 1, 'CLHC1': 1, 'CNGB3': 1, 'CNNM3': 1, 'COPA': 1, 'CST7': 1, 'CTBP1-AS2': 1, 'CTC-260E6.6': 1, 'CTD-2203K17.1': 1, 'CTD-2307P3.1': 1, 'CTD-3006G17.2': 1, 'CTH': 1, 'CYCS': 1, 'DCTN4': 1, 'DEAF1': 1, 'DGKI': 1, 'DNER': 1, 'DSTN': 1, 'EGF': 1, 'EMCN-IT3': 1, 'EOGT': 1, 'FAM210B': 1, 'FAM27E3': 1, 'FAM3B': 1, 'FAM86JP': 1, 'FZD3': 1, 'GBA3': 1, 'GCM1': 1, 'GGTA1P': 1, 'GLTSCR2': 1, 'GPR158-AS1': 1, 'GTF2A1': 1, 'GTPBP10': 1, 'GUCY1B2': 1, 'HCG18': 1, 'HELZ2': 1, 'HS3ST1': 1, 'IFNG-AS1': 1, 'IGHD3OR15-3B': 1, 'IGHV3OR16-13': 1, 'IGLV1-51': 1, 'IL12A-AS1': 1, 'INHBA-AS1': 1, 'KCND2': 1, 'KCNRG': 1, 'LAMA2': 1, 'LIFR-AS1': 1, 'LRRIQ1': 1, 'MED15P9': 1, 'MEGF10': 1, 'MGAM': 1, 'MIR4472-1': 1, 'MKLN1-AS1': 1, 'NABP1': 1, 'NCLN': 1, 'NDC80': 1, 'NFKBID': 1, 'NUFIP1': 1, 'OR2H1': 1, 'PARP4': 1, 'PCDHGA6': 1, 'PKD1L3': 1, 'PPP1R15A': 1, 'PRKCA': 1, 'PROX1-AS1': 1, 'RABGEF1': 1, 'RAPGEF4-AS1': 1, 'REXO1L1': 1, 'RHOU': 1, 'RN7SL139P': 1, 'RN7SL300P': 1, 'RNA5SP508': 1, 'RNU6-1163P': 1, 'RNU6-204P': 1, 'RNU6-439P': 1, 'RNU7-24P': 1, 'RP1-13P20.6': 1, 'RP1-167A14.2': 1, 'RP11-115D19.1': 1, 'RP11-130F10.1': 1, 'RP11-13J10.1': 1, 'RP11-152L20.3': 1, 'RP11-167H9.4': 1, 'RP11-290F24.3': 1, 'RP11-342D14.1': 1, 'RP11-356I2.4': 1, 'RP11-380P13.1': 1, 'RP11-438D8.2': 1, 'RP11-521M14.1': 1, 'RP11-525K10.3': 1, 'RP11-556E13.1': 1, 'RP11-57H12.5': 1, 'RP11-649A16.1': 1, 'RP11-665G4.1': 1, 'RP11-680B3.2': 1, 'RP11-693J15.4': 1, 'RP11-800A3.4': 1, 'RP11-846C15.2': 1, 'RP11-84A1.3': 1, 'RP11-933H2.4': 1, 'RP3-428L16.1': 1, 'RP4-605O3.4': 1, 'RP4-798P15.3': 1, 'SCARNA17': 1, 'SFXN1': 1, 'SIGLECL1': 1, 'SLC25A44': 1, 'SNHG11': 1, 'SNHG14': 1, 'SNX7': 1, 'SPATA3': 1, 'STXBP5-AS1': 1, 'SUSD2': 1, 'SVILP1': 1, 'THEMIS2': 1, 'TMEM161B-AS1': 1, 'TREML3P': 1, 'UCK1': 1, 'UGDH-AS1': 1, 'UHRF1': 1, 'UNC5B': 1, 'VPS37A': 1, 'WDR11-AS1': 1, 'Z93241.1': 1, 'ZC3H11A': 1, 'ZNF75D': 1, 'GNAS': 1, 'CTNNB1': 1, 'C16orf62': 1, 'ARID1A': 1, 'ZNF521': 1, 'KIT': 1, 'KDM5A': 1, 'AC027612.2': 1, 'AC079305.8': 1, 'AC110086.1': 1, 'CTD-2021J15.1': 1, 'CTSS': 1, 'FAM154A': 1, 'FZD1': 1, 'KIF1A': 1, 'NHLH1': 1, 'P2RY2': 1, 'PNMA1': 1, 'RP11-508N22.8': 1, 'RP13-578N3.3': 1, 'SNORD2': 1, 'SPANXA2-OT1': 1, 'ZCCHC4': 1, 'H3F3B': 1, 'IGKJ5': 1, 'HLA-A': 1, 'RALGDS': 1, 'FBXO11': 1, 'AKAP11': 1, 'WWTR1': 1, 'NOTCH1': 1, 'AC004485.3': 1, 'AC004862.6': 1, 'AC097467.2': 1, 'CRYM': 1, 'CTB-57H20.1': 1, 'CTD-2194D22.1': 1, 'DNAJB7': 1, 'HELQ': 1, 'KCND1': 1, 'LMCD1-AS1': 1, 'MCHR2-AS1': 1, 'MIR378D1': 1, 'OTX2-AS1': 1, 'PPP1R26-AS1': 1, 'PROM2': 1, 'PUM1': 1, 'RAET1K': 1, 'RFESD': 1, 'RP11-154D6.1': 1, 'RP11-184A2.3': 1, 'RP11-310I9.1': 1, 'RP11-492A10.1': 1, 'RP11-631F7.1': 1, 'RP11-731J8.2': 1, 'RP4-756H11.3': 1, 'SERPINB11': 1, 'SGK223': 1, 'SNORA31': 1, 'SPTBN4': 1, 'SULT1C2P1': 1, 'SYT4': 1, 'TG': 1, 'TMEM132B': 1, 'TNRC18': 1, 'TPTE2P1': 1, 'TRPC5': 1, 'VWA3B': 1, 'ZBTB7C': 1, 'ZCCHC11': 1, 'ZNF20': 1, 'ZNF222': 1, 'snoU13': 1} | 6.06993007 | 143 |
| {'Panc': 25, 'Eso': 5, 'Ovary': 4, 'Breast': 2, 'Liver': 2, 'Kidney': 1, 'Prost': 1, 'Bone': 1} | {'TP53': 26, 'KRAS': 21, 'SMAD4': 5, 'CDKN2A': 4, 'SMARCA4': 3, 'TGFBR2': 2, 'KLK2': 2, 'GNAS': 2, 'ZFHX3': 2, 'PTPRT': 2, 'PRPF40B': 2, 'PRRC2C': 1, 'ZNF521': 1, 'HLA-A': 1, 'PABPC1': 1, 'APC': 1, 'BAX': 1, 'PTEN': 1, 'RBM10': 1, 'PICALM': 1, 'HMGN2P46': 1, 'DCAF12L2': 1, 'CD209': 1} | 2.02439024 | 41 |
| {'Panc': 23, 'Liver': 6, 'Ovary': 4, 'Stomach': 1, 'Breast': 1, 'Prost': 1, 'Myeloid': 1} | {'KRAS': 23, 'TP53': 21, 'CTNNB1': 4, 'CTC-340A15.2': 4, 'AF121898.3': 3, 'RNF219-AS1': 3, 'RP11-586K2.1': 3, 'RP11-679C8.2': 3, 'hsa-mir-490': 3, 'CHST11': 2, 'ACVR2A': 2, 'KMT2C': 2, 'PREX2': 2, 'GATA3': 2, 'ARHGAP35': 2, 'EIF4A2': 2, 'KAT6A': 2, 'AC144521.1': 2, 'DISC1FP1': 2, 'RP11-274B21.1': 2, 'RP11-382A20.4': 2, 'RP11-420N3.2': 2, 'SNHG14': 2, 'KLF6': 2, 'RBM10': 2, 'CDKN2A': 1, 'DDR2': 1, 'FAT4': 1, 'RP11-1129I3.1': 1, 'NONO': 1, 'FAM135B': 1, 'KIT': 1, 'A2ML1-AS1': 1, 'AC000370.2': 1, 'AC004460.1': 1, 'AC004538.3': 1, 'AC004673.1': 1, 'AC005592.2': 1, 'AC007128.1': 1, 'AC007970.1': 1, 'AC009950.2': 1, 'AC016723.4': 1, 'AC092965.1': 1, 'AC105245.1': 1, 'AC108142.1': 1, 'AC126177.1': 1, 'ACO1': 1, 'ADAM5': 1, 'AE000661.37': 1, 'AL354806.1': 1, 'AL512640.1': 1, 'AP001596.6': 1, 'ARSK': 1, 'ATG16L1': 1, 'BAI3': 1, 'BEND4': 1, 'CASKIN1': 1, 'CCT6P3': 1, 'CDC20B': 1, 'COL6A1': 1, 'COL6A5': 1, 'CRYGEP': 1, 'CSDE1': 1, 'CSMD1': 1, 'CTB-118P15.2': 1, 'CTBS': 1, 'CTC-457E21.6': 1, 'CTD-2288O8.1': 1, 'CTD-2307P3.1': 1, 'CTD-3006G17.2': 1, 'DCST2': 1, 'DGKE': 1, 'DNAJB9': 1, 'DUT': 1, 'EMCN-IT3': 1, 'EMR4P': 1, 'FAM179A': 1, 'FAM84B': 1, 'FLG-AS1': 1, 'FNIP2': 1, 'FOLH1B': 1, 'FREM2': 1, 'GABRB1': 1, 'GBA3': 1, 'GDF7': 1, 'GFRAL': 1, 'GRM6': 1, 'GRPEL1': 1, 'HLA-AS1': 1, 'HPN-AS1': 1, 'HTR5BP': 1, 'IGHV2-5': 1, 'IL1RAP': 1, 'IL21R-AS1': 1, 'ILDR2': 1, 'JAG2': 1, 'KCNC1': 1, 'LINC00189': 1, 'LINC00475': 1, 'LINC00634': 1, 'LLNLF-65H9.1': 1, 'LRRC37A16P': 1, 'MEF2C-AS1': 1, 'MIR1268A': 1, 'MIR4431': 1, 'MIR4454': 1, 'MTMR9LP': 1, 'MTRNR2L7': 1, 'Metazoa_SRP': 1, 'NALCN-AS1': 1, 'NFATC3': 1, 'NPAS2': 1, 'NPSR1-AS1': 1, 'NTF4': 1, 'NUP188': 1, 'OR52M1': 1, 'OTX2-AS1': 1, 'PAIP2B': 1, 'PCDHA5': 1, 'PKD1L1': 1, 'PLEKHA8P1': 1, 'PNMA5': 1, 'RASGRP1': 1, 'RN7SKP149': 1, 'RN7SL278P': 1, 'RN7SL33P': 1, 'RN7SL644P': 1, 'RN7SL865P': 1, 'RNA5SP24': 1, 'RNA5SP474': 1, 'RNF215': 1, 'RNU2-54P': 1, 'RNU6-133P': 1, 'RNU6-227P': 1, 'RNU6-533P': 1, 'RNU7-174P': 1, 'RP1-23K20.2': 1, 'RP1-261D10.2': 1, 'RP1-272J12.1': 1, 'RP11-103J8.1': 1, 'RP11-114H24.5': 1, 'RP11-13J10.1': 1, 'RP11-152P17.2': 1, 'RP11-15B17.1': 1, 'RP11-1L12.3': 1, 'RP11-230B22.1': 1, 'RP11-231I13.2': 1, 'RP11-284M14.1': 1, 'RP11-286H14.4': 1, 'RP11-313J2.1': 1, 'RP11-333E1.1': 1, 'RP11-33N16.3': 1, 'RP11-368J21.3': 1, 'RP11-402C9.1': 1, 'RP11-428C19.4': 1, 'RP11-562L8.1': 1, 'RP11-572M11.4': 1, 'RP11-624C23.1': 1, 'RP11-634B7.4': 1, 'RP11-680F20.9': 1, 'RP11-707M1.1': 1, 'RP11-708B6.2': 1, 'RP11-742B18.1': 1, 'RP11-770E5.1': 1, 'RP11-77K12.5': 1, 'RP11-804A23.1': 1, 'RP11-867G2.8': 1, 'RP11-87M18.2': 1, 'RP11-89K10.1': 1, 'RP4-724E13.2': 1, 'RP4-755D9.1': 1, 'RP5-1091N2.9': 1, 'SCN10A': 1, 'SCRG1': 1, 'SEC63': 1, 'SEMA5A': 1, 'SHANK3': 1, 'SIGLEC9': 1, 'SLAIN2': 1, 'SLC46A1': 1, 'SMARCA2': 1, 'SPHK1': 1, 'SRGAP2-AS1': 1, 'SSPO': 1, 'STEAP2-AS1': 1, 'SUGT1P3': 1, 'SWT1': 1, 'TECTA': 1, 'TFAP2C': 1, 'TLE3': 1, 'TLR8-AS1': 1, 'TMEM230': 1, 'TOP3B': 1, 'TRBV28': 1, 'TRIM3': 1, 'TRPM6': 1, 'TSPAN32': 1, 'TYW1B': 1, 'U3': 1, 'USP2-AS1': 1, 'WDR83OS': 1, 'WSCD1': 1, 'XKR8': 1, 'Y_RNA': 1, 'ZFHX4': 1, 'ZFHX4-AS1': 1, 'snoU13': 1, 'PIK3CA': 1, 'DCAF12L2': 1, 'SDHA': 1, 'STAT5B': 1, 'BRAF': 1, 'PTPRT': 1, 'SMAD4': 1, 'CD209': 1} | 7.81081081 | 37 |
| {'Panc': 24, 'Ovary': 3, 'Breast': 2, 'Liver': 2, 'Biliary': 1, 'Head': 1, 'Skin': 1, 'Kidney': 1, 'Eso': 1} | {'KRAS': 27, 'TP53': 25, 'CDKN2A': 4, 'PBRM1': 2, 'ARID1A': 2, 'SMAD4': 2, 'RBM10': 1, 'USP45': 1, 'PIK3CA': 1, 'AFF3': 1, 'MUC16': 1, 'PTPRT': 1, 'MED12': 1, 'CTNNB1': 1, 'SND1': 1, 'ZPLD1': 1, 'RUNX1T1': 1, 'FLI1': 1, 'PRPF40B': 1, 'ZFHX3': 1} | 2.11111111 | 36 |
| {'Lymph': 11, 'Panc': 8, 'Eso': 4, 'Biliary': 2, 'Liver': 2, 'Ovary': 2, 'Breast': 1, 'Myeloid': 1} | {'TP53': 24, 'KRAS': 9, 'MYC': 6, 'TMSB4X': 6, 'RHOA': 3, 'PIM1': 3, 'XPO1': 3, 'JAK3': 2, 'ID3': 2, 'BCL6': 2, 'SOCS1': 2, 'SGK1': 2, 'PRKAR1A': 2, 'MECOM': 2, 'ARID1A': 1, 'PBX1': 1, 'U2AF1': 1, 'SMARCA4': 1, 'FBXO11': 1, 'ZFHX3': 1, 'PHOX2B': 1, 'KIAA1549': 1, 'CDH10': 1, 'PABPC1': 1, 'LRP1B': 1, 'FAT4': 1, 'ATM': 1, 'CSMD3': 1, 'BTG1': 1, 'FOXO1': 1} | 2.70967742 | 31 |
| {'Panc': 14, 'Eso': 5, 'Liver': 3, 'Lymph': 2, 'Stomach': 1, 'Breast': 1, 'CNS': 1} | {'TP53': 21, 'KRAS': 14, 'BCL2': 9, 'GNAS': 7, 'MAX': 2, 'U2AF1': 2, 'SMARCA4': 1, 'RPL10': 1, 'PDE4DIP': 1, 'CTNNB1': 1, 'NRAS': 1, 'FLT4': 1, 'DCAF12L2': 1, 'SMAD4': 1, 'APC': 1, 'PTEN': 1} | 2.40740741 | 27 |
| {'Breast': 10, 'Liver': 5, 'Panc': 4, 'Ovary': 2, 'CNS': 2, 'Skin': 1, 'Prost': 1} | {'PIK3CA': 11, 'TP53': 8, 'CTNNB1': 6, 'KRAS': 4, 'ARID2': 3, 'LEPROTL1': 2, 'PTEN': 2, 'DDX3X': 2, 'RB1': 2, 'GATA3': 1, 'ERBB3': 1, 'MLLT3': 1, 'CTNNA2': 1, 'GNAS': 1, 'RP11-1103G16.1': 1} | 1.84 | 25 |
| {'Skin': 17, 'Stomach': 1, 'Lymph': 1, 'Liver': 1, 'Biliary': 1, 'Kidney': 1, 'CNS': 1, 'Myeloid': 1, 'Prost': 1} | {'BRAF': 12, 'NRAS': 7, 'ERBB4': 5, 'PTPRT': 4, 'MUC16': 4, 'RUNX1T1': 3, 'IDH1': 3, 'TMSB4X': 3, 'PBX1': 2, 'PRRX1': 2, 'GRIN2A': 2, 'PREX2': 2, 'CDKN2A': 2, 'CNTNAP2': 2, 'MTOR': 2, 'KMT2D': 2, 'CUL3': 1, 'PGM5P2': 1, 'CCND2': 1, 'TP53': 1, 'MEPCE': 1, 'PDE4B': 1, 'CD28': 1, 'KLK2': 1, 'TRPM1': 1, 'PTPRB': 1, 'EPHA7': 1, 'MECOM': 1, 'AMER1': 1, 'KNSTRN': 1, 'ERCC4': 1, 'SETBP1': 1, 'SMAD2': 1, 'GRM3': 1, 'POT1': 1, 'TERT': 1, 'ZFHX3': 1, 'CDH10': 1, 'BTG1': 1, 'BCL6': 1, 'SGK1': 1} | 3.28 | 25 |
| {'Liver': 15, 'Ovary': 2, 'Eso': 2, 'Skin': 1, 'Biliary': 1, 'Panc': 1, 'Prost': 1} | {'CTNNB1': 21, 'CTNNA2': 7, 'ARID2': 6, 'ELF3': 5, 'KDM5A': 5, 'ALDH2': 5, 'HSP90AA1': 5, 'USP8': 5, 'COL1A1': 5, 'PBRM1': 5, 'TET2': 5, 'CDH10': 5, 'SGK1': 5, 'KIAA1549': 5, 'EIF1AX': 5, 'STAG2': 5, 'TCF12': 5, 'TMPRSS2': 5, 'TP53': 4, 'MUC16': 2, 'PREX2': 2, 'PBX1': 1, 'CCND2': 1, 'PTPRB': 1, 'CRNKL1': 1, 'MLH1': 1, 'EZH2': 1, 'TBL1XR1': 1, 'STAG1': 1, 'ROBO2': 1, 'SMARCA4': 1, 'AFF3': 1, 'ARID1A': 1} | 5.60869565 | 23 |
| {'Eso': 5, 'Liver': 3, 'Prost': 2, 'Myeloid': 2, 'Biliary': 1, 'Stomach': 1, 'Skin': 1, 'Ovary': 1, 'Kidney': 1, 'Panc': 1, 'Bone': 1} | {'AR': 14, 'PRCC': 10, 'DDX6': 10, 'RB1': 10, 'TBL1XR1': 10, 'RP11-420N3.2': 10, 'TRIM33': 9, 'PDE4DIP': 9, 'LCP1': 9, 'SPOP': 9, 'KAT6A': 9, 'TP53': 8, 'RP11-32K4.1': 8, 'RPL23AP79': 8, 'Y_RNA': 8, 'CTD-3006G17.2': 7, 'LINC00535': 7, 'RP11-17E2.2': 7, 'snoU13': 7, 'BCR': 7, 'MUC16': 6, 'LMNA': 6, 'RP11-541P9.3': 5, 'MUC4': 5, 'CTC-340A15.2': 5, 'RP11-586K2.1': 5, 'RP11-152L20.3': 5, 'SFRP4': 4, 'AC079610.1': 4, 'BAGE2': 4, 'DISC1FP1': 4, 'MEF2C-AS1': 4, 'RNF219-AS1': 4, 'RP11-3B12.1': 4, 'ZFHX4-AS1': 4, 'EPHA7': 3, 'AC007879.5': 3, 'GS1-256O22.5': 3, 'LINC00395': 3, 'RP11-550P17.5': 3, 'RP11-649A16.1': 3, 'RP13-492C18.2': 3, 'RP3-399L15.3': 3, 'AF121898.3': 3, 'CTCF': 3, 'ANK1': 2, 'PIK3CA': 2, 'BRAF': 2, 'MAF': 2, 'RP11-804A23.2': 2, 'HOXC13': 2, 'AC004053.1': 2, 'AC004791.2': 2, 'AC007277.3': 2, 'AC027612.3': 2, 'AC097467.2': 2, 'AP000320.7': 2, 'CTD-2269F5.1': 2, 'EPHA1-AS1': 2, 'FLG-AS1': 2, 'IL12A-AS1': 2, 'INHBA-AS1': 2, 'NPSR1-AS1': 2, 'RP11-439L18.3': 2, 'RP11-525K10.3': 2, 'RP11-624L4.1': 2, 'RP11-692D12.1': 2, 'RP11-707M1.1': 2, 'RP11-770E5.1': 2, 'RP11-820L6.1': 2, 'SCN4A': 2, 'SEC24B-AS1': 2, 'SNORA26': 2, 'TMEM200C': 2, 'TMPRSS4-AS1': 2, 'TRAV27': 2, 'AC004538.3': 2, 'AC007319.1': 2, 'AC009236.1': 2, 'AC079613.1': 2, 'CTC-525D6.1': 2, 'F11-AS1': 2, 'RP11-357C3.3': 2, 'RP11-624C23.1': 2, 'RP11-679C8.2': 2, 'RP5-896L10.1': 2, 'RP11-155G15.2': 2, 'KRAS': 1, 'CSMD3': 1, 'RPL22': 1, 'LARP4B': 1, 'VTI1A': 1, 'ATM': 1, 'KDM5A': 1, 'SIX1': 1, 'BCL11B': 1, 'CIITA': 1, 'SPECC1': 1, 'PPP2R1A': 1, 'ACVR2A': 1, 'N4BP2': 1, 'PHOX2B': 1, 'IL6ST': 1, 'HNRNPA2B1': 1, 'HOOK3': 1, 'EXT1': 1, 'BCL10': 1, 'PBX1': 1, 'POU2AF1': 1, 'ATF1': 1, 'TCL1A': 1, 'AXIN1': 1, 'GRIN2A': 1, 'USP6': 1, 'PTPRT': 1, 'PTPRD': 1, 'CDKN2A': 1, 'APC': 1, 'AACSP1': 1, 'AC004901.1': 1, 'AC009120.6': 1, 'AC012501.2': 1, 'AC015922.5': 1, 'AC016907.3': 1, 'AC024908.1': 1, 'AC061961.2': 1, 'AC091878.1': 1, 'AC097468.7': 1, 'AC102953.4': 1, 'AC144521.1': 1, 'ACE': 1, 'ADCY1': 1, 'ADPRHL2': 1, 'ADRBK2': 1, 'AF186996.1': 1, 'AL133247.2': 1, 'AL159977.1': 1, 'AL162511.1': 1, 'AL353791.1': 1, 'AL590726.1': 1, 'AL928742.2': 1, 'ANGPTL7': 1, 'ANKRD20A1': 1, 'ANKRD26P1': 1, 'ANKRD6': 1, 'AOC1': 1, 'AP000765.1': 1, 'AP001525.1': 1, 'AP002954.4': 1, 'ARHGEF26-AS1': 1, 'ARHGEF37': 1, 'BCORP1': 1, 'BRINP2': 1, 'C11orf84': 1, 'C2orf16': 1, 'C6': 1, 'CAB39L': 1, 'CASC8': 1, 'CCT6P3': 1, 'CDHR5': 1, 'CLRN1-AS1': 1, 'COL26A1': 1, 'CPNE4': 1, 'CSTF3-AS1': 1, 'CTB-186H2.2': 1, 'CTBP1-AS2': 1, 'CTC-260E6.6': 1, 'CTC-297N7.5': 1, 'CTD-2001E22.1': 1, 'CTD-2015H6.3': 1, 'CTD-2176I21.2': 1, 'CTD-2215L10.1': 1, 'CTD-2307P3.1': 1, 'CTNNA3': 1, 'CXXC1P1': 1, 'CXXC4': 1, 'DARC': 1, 'DLG2': 1, 'ELOVL2': 1, 'ENTPD3-AS1': 1, 'ERMP1': 1, 'FAM120AOS': 1, 'FAM153B': 1, 'FAM181A-AS1': 1, 'FAM184B': 1, 'FAM230B': 1, 'FETUB': 1, 'FNDC1': 1, 'FOLH1B': 1, 'FREM2': 1, 'GATAD2A': 1, 'GBA3': 1, 'GPR156': 1, 'GPR4': 1, 'GTF2H5': 1, 'GTPBP10': 1, 'GUCY1B2': 1, 'HEATR6': 1, 'HEG1': 1, 'HHIPL1': 1, 'HMX3': 1, 'HTR5BP': 1, 'IGHV3OR16-13': 1, 'IGKV1D-37': 1, 'INTS4': 1, 'INTS4L1': 1, 'KCNMB3P1': 1, 'KIAA1257': 1, 'KIAA1683': 1, 'KIF25': 1, 'KIRREL3-AS3': 1, 'KLHL3': 1, 'LINC00467': 1, 'LINC00963': 1, 'LINC01088': 1, 'LMCD1-AS1': 1, 'MIR31HG': 1, 'MIR548H4': 1, 'MIR596': 1, 'MKLN1-AS1': 1, 'MMP8': 1, 'MSLN': 1, 'MTHFD2P1': 1, 'MTRNR2L5': 1, 'MTSS1L': 1, 'NELFA': 1, 'NRGN': 1, 'NT5DC4': 1, 'OR5D18': 1, 'OR8B8': 1, 'PASD1': 1, 'PCBP1-AS1': 1, 'PCDHB3': 1, 'PDE10A': 1, 'PEG10': 1, 'PEX7': 1, 'PI3': 1, 'PPFIA1': 1, 'PPP1R35': 1, 'PSTK': 1, 'RFTN1P1': 1, 'RHBDL3': 1, 'RN7SKP227': 1, 'RN7SL564P': 1, 'RN7SL7P': 1, 'RN7SL89P': 1, 'RNA5SP358': 1, 'RNA5SP403': 1, 'RNU1-59P': 1, 'RNU4-57P': 1, 'RNU6-102P': 1, 'RNU6-1047P': 1, 'RNU6-442P': 1, 'RNU6-52P': 1, 'RNU6-543P': 1, 'RNU6-656P': 1, 'RP1-240B8.3': 1, 'RP1-28O10.1': 1, 'RP1-65P5.1': 1, 'RP1-93I3.1': 1, 'RP11-100L22.2': 1, 'RP11-1017G21.4': 1, 'RP11-1084E5.1': 1, 'RP11-1103G16.1': 1, 'RP11-1129I3.1': 1, 'RP11-125B21.2': 1, 'RP11-1277A3.2': 1, 'RP11-13G14.4': 1, 'RP11-13J10.1': 1, 'RP11-142C4.6': 1, 'RP11-144L1.4': 1, 'RP11-145A3.1': 1, 'RP11-158J3.2': 1, 'RP11-159L20.2': 1, 'RP11-166B2.8': 1, 'RP11-17A1.3': 1, 'RP11-231I13.2': 1, 'RP11-231P20.2': 1, 'RP11-23D24.2': 1, 'RP11-274B21.1': 1, 'RP11-280O1.2': 1, 'RP11-285J16.1': 1, 'RP11-324H6.5': 1, 'RP11-326E22.1': 1, 'RP11-343D2.11': 1, 'RP11-379F4.4': 1, 'RP11-431M7.2': 1, 'RP11-431M7.3': 1, 'RP11-445F12.1': 1, 'RP11-446J8.1': 1, 'RP11-454C18.2': 1, 'RP11-513G19.1': 1, 'RP11-526D8.7': 1, 'RP11-538P18.2': 1, 'RP11-551L14.1': 1, 'RP11-556E13.1': 1, 'RP11-58B2.1': 1, 'RP11-702H23.4': 1, 'RP11-706C16.8': 1, 'RP11-742B18.1': 1, 'RP11-744N12.3': 1, 'RP11-752G15.9': 1, 'RP11-75N4.2': 1, 'RP11-788M5.3': 1, 'RP11-86L19.2': 1, 'RP11-978I15.10': 1, 'RP3-323P13.2': 1, 'RP4-630C24.3': 1, 'RP5-1121H13.4': 1, 'RP5-905H7.3': 1, 'SEC14L4': 1, 'SLAMF6': 1, 'SLC18A2': 1, 'SLC26A2': 1, 'SLC31A1': 1, 'SLC46A1': 1, 'SLC5A3': 1, 'SLC8A1': 1, 'SLC8A1-AS1': 1, 'SNAPC1': 1, 'SNORA51': 1, 'SNORD29': 1, 'SNRPD2': 1, 'SORL1': 1, 'SRD5A3-AS1': 1, 'SRGAP2B': 1, 'SSPO': 1, 'SUPT20H': 1, 'TCHH': 1, 'TFAP2B': 1, 'TGS1': 1, 'THSD1': 1, 'TMEM254-AS1': 1, 'TNS3': 1, 'TPTE2P1': 1, 'TTN': 1, 'TYW1B': 1, 'USP15': 1, 'WDR74': 1, 'XRCC5': 1, 'ZFYVE21': 1, 'ZNF207': 1, 'ZNF527': 1, 'ZNF665': 1, 'ZNF844': 1, 'ZNRD1-AS1': 1, 'ZRANB2-AS1': 1, 'ZRANB2-AS2': 1, 'hsa-mir-490': 1, 'ABCC4': 1, 'AC004041.2': 1, 'AC004458.1': 1, 'AC005062.2': 1, 'AC005592.2': 1, 'AC006322.1': 1, 'AC008836.1': 1, 'AC009677.1': 1, 'AC012363.4': 1, 'AC016725.4': 1, 'AC073479.1': 1, 'AC079135.1': 1, 'AC079630.4': 1, 'AC083906.1': 1, 'AC092846.2': 1, 'AC093874.1': 1, 'AC096579.13': 1, 'AC098617.1': 1, 'AC108696.1': 1, 'AC138761.1': 1, 'ADAL': 1, 'AL139815.1': 1, 'AL359709.2': 1, 'ALDH9A1': 1, 'ANKRD12': 1, 'AP001597.1': 1, 'ARHGAP11B': 1, 'ARHGEF40': 1, 'ASNA1': 1, 'ATG5': 1, 'BPIFA4P': 1, 'C10orf12': 1, 'C17orf80': 1, 'CD2AP': 1, 'CDC42SE2': 1, 'CDH23': 1, 'CDKN2B-AS1': 1, 'CHIC1': 1, 'CNGA1': 1, 'COL24A1': 1, 'COL5A3': 1, 'CTB-49A3.2': 1, 'CTC-457E21.9': 1, 'CTD-2547L16.1': 1, 'CTD-3088G3.8': 1, 'CTTNBP2': 1, 'CYP4B1': 1, 'DCAF4L2': 1, 'DDX11-AS1': 1, 'DDX50': 1, 'DDX60': 1, 'DIO2-AS1': 1, 'DIRC3': 1, 'DOCK5': 1, 'DUOX2': 1, 'EFCAB1': 1, 'EGFEM1P': 1, 'EHHADH-AS1': 1, 'EIF4E': 1, 'EMILIN1': 1, 'EPSTI1': 1, 'ERICH1-AS1': 1, 'ESPNP': 1, 'ESRRAP2': 1, 'FAIM2': 1, 'FAM177A1': 1, 'FBN1': 1, 'FBXL5': 1, 'FBXO28': 1, 'FZD10-AS1': 1, 'GJD2': 1, 'GLUD1P2': 1, 'GPD2': 1, 'GPR133': 1, 'GPT': 1, 'GRID1': 1, 'GRIPAP1': 1, 'GUSBP1': 1, 'HCG18': 1, 'HCN4': 1, 'HEBP2': 1, 'HIST1H1A': 1, 'HMGCS1': 1, 'HNRNPA1P48': 1, 'IGFBP7-AS1': 1, 'IGHD3-10': 1, 'IGHV1-24': 1, 'IGKV1-27': 1, 'IGLV7-46': 1, 'ITGB2': 1, 'KB-1562D12.1': 1, 'KCNB2': 1, 'KCNV1': 1, 'KHDRBS3': 1, 'KIAA2022': 1, 'KLF11': 1, 'KNTC1': 1, 'LEF1-AS1': 1, 'LINC00189': 1, 'LMTK2': 1, 'LRP3': 1, 'LRRC49': 1, 'MAGI3': 1, 'MAMDC2': 1, 'MCC': 1, 'MCHR2-AS1': 1, 'MIR184': 1, 'MIR3622A': 1, 'MIR496': 1, 'MLK7-AS1': 1, 'MRPL45P2': 1, 'MYO18A': 1, 'NALCN-AS1': 1, 'NOVA1-AS1': 1, 'OIT3': 1, 'PDE3A': 1, 'PICK1': 1, 'PIK3CG': 1, 'PPIP5K2': 1, 'PROX1-AS1': 1, 'PVRL4': 1, 'RAD1': 1, 'RAPGEFL1': 1, 'RGS18': 1, 'RN7SL170P': 1, 'RN7SL251P': 1, 'RN7SL357P': 1, 'RN7SL572P': 1, 'RN7SL600P': 1, 'RN7SL637P': 1, 'RN7SL649P': 1, 'RNA5SP116': 1, 'RNA5SP504': 1, 'RNA5SP62': 1, 'RNF217': 1, 'RNMT': 1, 'RNPC3': 1, 'RNU6-1070P': 1, 'RNU6-243P': 1, 'RNU6-741P': 1, 'RNU7-136P': 1, 'RNY4P27': 1, 'RP1-167F1.2': 1, 'RP11-1069G10.1': 1, 'RP11-115D19.1': 1, 'RP11-150C16.1': 1, 'RP11-154D6.1': 1, 'RP11-163M18.1': 1, 'RP11-175P19.2': 1, 'RP11-196E1.3': 1, 'RP11-33N16.3': 1, 'RP11-342D14.1': 1, 'RP11-366F6.2': 1, 'RP11-368M16.3': 1, 'RP11-370I10.6': 1, 'RP11-385J1.2': 1, 'RP11-422J8.1': 1, 'RP11-423H2.3': 1, 'RP11-429B14.1': 1, 'RP11-435M3.2': 1, 'RP11-505P4.7': 1, 'RP11-519G16.3': 1, 'RP11-532F12.5': 1, 'RP11-54D18.3': 1, 'RP11-562L8.1': 1, 'RP11-572M11.4': 1, 'RP11-611E13.2': 1, 'RP11-634B7.4': 1, 'RP11-665G4.1': 1, 'RP11-66D17.5': 1, 'RP11-678G14.2': 1, 'RP11-76N22.2': 1, 'RP11-804N13.1': 1, 'RP11-826N14.1': 1, 'RP11-89K10.1': 1, 'RP11-92C4.3': 1, 'RP11-93K22.6': 1, 'RP11-98D18.15': 1, 'RP3-340N1.5': 1, 'RP4-651E10.4': 1, 'RP4-694A7.4': 1, 'RP5-991G20.1': 1, 'RUNDC1': 1, 'SAGE1': 1, 'SCRN1': 1, 'SEPT14': 1, 'SHANK2': 1, 'SIGLEC6': 1, 'SLC10A7': 1, 'SLC16A10': 1, 'SLC25A32': 1, 'SLC25A38': 1, 'SLC30A2': 1, 'SLC7A14': 1, 'SLFN5': 1, 'SRD5A2': 1, 'STARD13': 1, 'SUCO': 1, 'TAOK3': 1, 'TCF21': 1, 'TENC1': 1, 'TEX26-AS1': 1, 'TMEM154': 1, 'TMEM38B': 1, 'TRAPPC3L': 1, 'TRBV6-8': 1, 'TRIM69': 1, 'U3': 1, 'U8': 1, 'UBE2Q2': 1, 'UFL1-AS1': 1, 'UMODL1': 1, 'UNG': 1, 'URB1': 1, 'UVRAG': 1, 'VNN2': 1, 'VSTM4': 1, 'VTI1B': 1, 'WDR5B': 1, 'Z95704.4': 1, 'ZBED3-AS1': 1, 'ZBED5-AS1': 1, 'ZBTB38': 1, 'ZNF106': 1, 'ZNF341': 1, 'ZNF815P': 1, 'ZNF827': 1, 'H3F3A': 1, 'AC093391.2': 1, 'AL626787.1': 1, 'AL672294.1': 1, 'ALOX12P2': 1, 'ARHGAP40': 1, 'CACNA1C': 1, 'CMAHP': 1, 'COPB1': 1, 'CTD-2058B24.2': 1, 'CYLC2': 1, 'DZIP1': 1, 'FSIP2': 1, 'ISM2': 1, 'PPP1R12C': 1, 'RNA5SP459': 1, 'RP11-81K13.1': 1, 'RP11-944L7.4': 1, 'SNORD81': 1, 'SUPT6H': 1, 'TM4SF1-AS1': 1, 'AC072062.1': 1, 'AC108142.1': 1, 'AC136188.1': 1, 'AC136932.2': 1, 'AC144450.1': 1, 'CTB-57H20.1': 1, 'CTSL3P': 1, 'HLTF-AS1': 1, 'HTR3B': 1, 'IGHM': 1, 'NFYB': 1, 'NLGN4X': 1, 'NR2F2-AS1': 1, 'PRKY': 1, 'RP1-272J12.1': 1, 'RP11-122D10.1': 1, 'RP11-135J2.4': 1, 'RP11-482H16.1': 1, 'RP11-542K23.7': 1, 'STEAP2-AS1': 1, 'TRGC2': 1, 'UCHL1-AS1': 1} | 46.8947368 | 19 |
| {'Panc': 7, 'Eso': 5, 'Breast': 3, 'Stomach': 1, 'Liver': 1, 'Lymph': 1, 'CNS': 1} | {'TP53': 15, 'KRAS': 6, 'CDKN2A': 6, 'CDH11': 2, 'MYC': 2, 'BCL9': 2, 'CD209': 2, 'SMAD4': 2, 'SMARCA4': 1, 'CARD11': 1, 'HMGN2P46': 1, 'MUC16': 1, 'PTPN13': 1, 'RPL22': 1, 'LARP4B': 1, 'BCL11B': 1, 'MYO5A': 1, 'LYL1': 1, 'ASXL2': 1, 'ACVR2A': 1, 'N4BP2': 1, 'MLLT3': 1, 'DDX3X': 1, 'NONO': 1, 'BCL11A': 1, 'PIK3CA': 1, 'PREX2': 1, 'H3F3A': 1} | 3 | 19 |
| {'Liver': 5, 'Breast': 4, 'Panc': 3, 'Head': 2, 'Eso': 2, 'Biliary': 1, 'Skin': 1, 'Lymph': 1} | {'TP53': 8, 'PIK3CA': 6, 'CDKN2A': 6, 'CTNNB1': 4, 'KRAS': 3, 'MUC16': 2, 'ANK1': 2, 'SF3B1': 1, 'GATA3': 1, 'TGFBR2': 1, 'TERT': 1, 'RNF43': 1, 'LIFR': 1, 'MACC1': 1, 'BRAF': 1, 'DDX46': 1, 'SMARCA4': 1, 'FBXO11': 1, 'CCR7': 1} | 2.26315789 | 19 |
| {'Skin': 8, 'Liver': 4, 'Panc': 2, 'Stomach': 1, 'Eso': 1, 'Lymph': 1} | {'NRAS': 8, 'B2M': 5, 'MUC16': 5, 'CTNNB1': 3, 'TP53': 3, 'ATM': 3, 'MECOM': 3, 'PTPRT': 3, 'RGS7': 2, 'ARHGEF12': 2, 'CDKN2A': 2, 'TCF7L2': 2, 'DCC': 2, 'ERBB4': 2, 'CUL3': 2, 'RBM15': 1, 'LIFR': 1, 'AC016725.4': 1, 'BCL10': 1, 'TCL1A': 1, 'FAM135B': 1, 'PPP6C': 1, 'NBEA': 1, 'HMGN2P46': 1, 'SMARCA4': 1, 'DDR2': 1, 'ELK4': 1, 'SIX1': 1, 'SMAD2': 1, 'NONO': 1, 'DIAPH2-AS1': 1, 'PDE4DIP': 1, 'POU2AF1': 1, 'SRGAP3': 1, 'PTEN': 1, 'A1CF': 1, 'ERCC4': 1, 'CSMD3': 1, 'GRIN2A': 1, 'SETBP1': 1, 'KEAP1': 1, 'CTNNA2': 1, 'LRP1B': 1, 'CDH10': 1, 'CSF1R': 1, 'JAZF1': 1, 'PTPRD': 1, 'IBTK': 1, 'KRAS': 1, 'ATRX': 1, 'PIM1': 1, 'TMSB4X': 1} | 4.94117647 | 17 |
| {'Lymph': 16, 'Kidney': 1} | {'BCL2': 36, 'PIM1': 12, 'TMSB4X': 12, 'MYC': 6, 'CREBBP': 4, 'MYD88': 4, 'MUC16': 3, 'SOCS1': 3, 'BCR': 2, 'CD79B': 2, 'BTG2': 2, 'BCL6': 2, 'SGK1': 2, 'ID3': 1, 'RPL10': 1, 'B2M': 1, 'LRP1B': 1, 'IGKJ3': 1} | 5.58823529 | 17 |
| {'Panc': 6, 'Breast': 4, 'Eso': 3, 'Stomach': 1, 'Prost': 1, 'Ovary': 1} | {'SF3B1': 9, 'TP53': 8, 'KRAS': 6, 'AKT1': 2, 'NRG1': 2, 'KLK2': 2, 'MB21D2': 2, 'KIT': 2, 'PDGFRB': 2, 'PIK3CA': 1, 'SDHA': 1, 'ROBO2': 1} | 2.375 | 16 |
| {'Prost': 4, 'Liver': 3, 'Breast': 2, 'Eso': 2, 'Head': 1, 'Skin': 1, 'Lymph': 1, 'Panc': 1} | {'TP53': 12, 'PIK3CA': 4, 'RUNX1T1': 4, 'IDH1': 2, 'KRAS': 2, 'FAT4': 2, 'SOCS1': 1, 'SMARCA4': 1, 'TGFBR2': 1, 'TMSB4X': 1, 'B2M': 1, 'LYL1': 1} | 2.13333333 | 15 |
| {'Liver': 4, 'Panc': 3, 'Stomach': 2, 'Skin': 2, 'Breast': 1, 'CNS': 1, 'Prost': 1} | {'TP53': 10, 'CTNNB1': 3, 'MUC16': 3, 'KRAS': 3, 'ROBO2': 2, 'CNTNAP2': 2, 'PREX2': 2, 'NUP214': 2, 'TBL1XR1': 1, 'PTPRC': 1, 'RGS7': 1, 'FAT3': 1, 'PAFAH1B2': 1, 'NBEA': 1, 'PRKD1': 1, 'GRIN2A': 1, 'PTPRT': 1, 'MLH1': 1, 'MECOM': 1, 'GRM3': 1, 'KIAA1549': 1, 'CSMD3': 1, 'SLC24A5': 1, 'PTPN13': 1, 'FAT4': 1, 'HLA-A': 1} | 3.21428571 | 14 |
| {'Eso': 4, 'Panc': 3, 'Biliary': 1, 'Skin': 1, 'Ovary': 1} | {'TP53': 9, 'KRAS': 3, 'MUC16': 2, 'SETBP1': 2, 'ARID1A': 1, 'PBX1': 1, 'PTPRC': 1, 'KMT2D': 1, 'TCL1A': 1, 'CTNNA2': 1, 'CD28': 1, 'CRNKL1': 1, 'PTPRT': 1, 'CDH10': 1, 'CSF1R': 1, 'POT1': 1, 'CNTNAP2': 1, 'CDKN2A': 1, 'RP11-231I13.2': 1, 'REL': 1, 'ERBB2': 1, 'APC': 1, 'PTEN': 1, 'BAX': 1} | 3.6 | 10 |
| {'Eso': 5, 'Breast': 1, 'Head': 1, 'Liver': 1, 'Panc': 1} | {'TP53': 7, 'USP6': 2, 'ERBB2': 2, 'CDKN2A': 2, 'APC': 1, 'LRP1B': 1, 'KRAS': 1} | 1.77777778 | 9 |
| {'Liver': 5, 'Stomach': 1, 'Prost': 1, 'Lymph': 1} | {'CEBPA': 7, 'SNX29': 5, 'CTNNB1': 4, 'ARID1A': 3, 'RB1': 3, 'SOX21': 3, 'KTN1': 3, 'AKT1': 3, 'MAF': 3, 'ASXL2': 3, 'CTNNA2': 3, 'ERBB4': 3, 'TGFBR2': 3, 'FAT4': 3, 'NRG1': 3, 'CSMD3': 3, 'MLLT3': 3, 'STIL': 3, 'TP53': 2, 'PWWP2A': 2, 'SF3B1': 2, 'MACC1': 2, 'QKI': 2, 'PICALM': 1, 'MEF2C-AS1': 1} | 9.125 | 8 |
| {'Skin': 8} | {'BRAF': 8, 'MUC16': 8, 'PTPRT': 5, 'PTPRB': 4, 'ERBB4': 3, 'CSMD3': 1, 'TMEM121': 1, 'RBM15': 1, 'RGS7': 1, 'GRIN2A': 1, 'MACC1': 1, 'SND1': 1, 'CDKN2A': 1, 'TP53': 1, 'TM9SF4': 1, 'PAFAH1B2': 1, 'ROBO2': 1, 'ELK4': 1, 'A1CF': 1, 'LRP1B': 1, 'NFKBIE': 1, 'CNTNAP2': 1, 'PREX2': 1, 'PAX5': 1, 'AMER1': 1, 'SMARCD2': 1, 'KMT2D': 1, 'KLK2': 1, 'CUL3': 1, 'MECOM': 1, 'TRRAP': 1, 'PTEN': 1} | 6.875 | 8 |
| {'Liver': 4, 'Panc': 1, 'Kidney': 1, 'Prost': 1} | {'TP53': 9, 'CSMD3': 9, 'CTNNB1': 7, 'NBEA': 5, 'CTNNA2': 5, 'TBL1XR1': 5, 'MLLT3': 5, 'SNX29': 5, 'RABEP1': 5, 'SGK1': 4, 'PMS2': 2, 'KRAS': 1, 'CEP170P1': 1, 'RUSC2': 1} | 9.14285714 | 7 |
| {'Panc': 2, 'CNS': 2, 'Liver': 2} | {'CTNNB1': 4, 'SMARCA4': 3, 'GNAS': 2, 'TP53': 2, 'CCR7': 1, 'CSMD3': 1, 'BMPR1A': 1, 'LEF1': 1, 'EXT1': 1, 'CTA-85E5.10': 1} | 2.83333333 | 6 |
| {'Lymph': 6} | {'BCL2': 34, 'EZH2': 2, 'STAT6': 2, 'FAM135B': 1, 'PIM1': 1} | 6.66666667 | 6 |
| {'Panc': 2, 'Breast': 1, 'Ovary': 1, 'Eso': 1, 'Bone': 1} | {'TP53': 7, 'KRAS': 2, 'CTNNA2': 1} | 1.66666667 | 6 |
| {'Panc': 3, 'Breast': 1, 'Head': 1, 'Liver': 1} | {'TP53': 6, 'KRAS': 3, 'CSMD3': 1, 'TAL1': 1, 'CDKN2A': 1, 'HMCN1': 1, 'NANOS2': 1, 'ZFHX3': 1} | 2.5 | 6 |
| {'CNS': 3, 'Breast': 1, 'Biliary': 1} | {'SMO': 3, 'PIK3CA': 2, 'TRRAP': 2, 'RPL22': 1} | 1.6 | 5 |
| {'Breast': 5} | {'GATA3': 5, 'PIK3CA': 4, 'CBFB': 1, 'SMARCA4': 1} | 2.2 | 5 |
| {'Panc': 2, 'Eso': 2, 'Myeloid': 1} | {'KRAS': 2, 'SMAD4': 2, 'ERBB4': 2, 'MUC16': 2} | 1.6 | 5 |
| {'Panc': 4, 'Eso': 1} | {'KRAS': 5, 'CDKN2A': 5, 'TP53': 3, 'U2AF1': 1} | 2.8 | 5 |
| {'Kidney': 2, 'Prost': 2} | {'TP53': 4, 'VAV1': 3, 'DGCR8': 3, 'NF2': 3, 'STAG2': 3, 'CDH11': 3, 'ZFHX3': 3, 'LEF1': 3, 'KMT2C': 3, 'VHL': 2, 'ROBO2': 2, 'RN7SKP104': 1} | 8.25 | 4 |
| {'Eso': 3, 'Liver': 1} | {'CDH11': 3, 'TP53': 2, 'BMP5': 2, 'MECOM': 1, 'ANK1': 1, 'FOXO1': 1} | 2.5 | 4 |
| {'Prost': 2, 'Breast': 1, 'Bone': 1} | {'FOXA1': 8, 'CDK12': 8, 'KCNJ5': 7, 'PTEN': 6, 'PTK6': 6, 'IKZF1': 6, 'BCL9': 4, 'CUL3': 4, 'CRNKL1': 4, 'MUC4': 4, 'IL6ST': 4, 'GNA11': 3, 'SETBP1': 2, 'CTNND2': 2, 'NPM1': 2, 'KDM6A': 2, 'AC091320.2': 2, 'FNDC3B': 2, 'RP11-32K4.1': 2, 'RP11-586K2.1': 2, 'ABCA3': 1, 'AC016907.3': 1, 'AC079610.1': 1, 'AC092048.1': 1, 'ADAMTS9-AS2': 1, 'ATP2C1': 1, 'BDNF-AS': 1, 'C19orf35': 1, 'CTC-297N7.11': 1, 'CTD-2014E2.5': 1, 'CTD-2307P3.1': 1, 'CTD-3006G17.2': 1, 'F11-AS1': 1, 'FARSB': 1, 'FCRL3': 1, 'IFNG-AS1': 1, 'INHBA': 1, 'KIAA0513': 1, 'KIF9-AS1': 1, 'LL09NC01-254D11.1': 1, 'MEF2C-AS1': 1, 'MIR518D': 1, 'MIR526B': 1, 'NREP-AS1': 1, 'OR14A2': 1, 'PMS2CL': 1, 'RASA2': 1, 'RBFOX2': 1, 'RN7SL575P': 1, 'RN7SL659P': 1, 'RNA5SP36': 1, 'RNU4-65P': 1, 'RP1-91G5.3': 1, 'RP11-167N24.3': 1, 'RP11-17E2.2': 1, 'RP11-431M7.2': 1, 'RP11-446J8.1': 1, 'RP11-550I24.2': 1, 'RP11-624L4.1': 1, 'RP11-644F5.16': 1, 'RP11-657O9.1': 1, 'RP11-708B6.2': 1, 'RP4-735C1.4': 1, 'RP4-777D9.2': 1, 'RPA3-AS1': 1, 'SERPINB9': 1, 'SLC15A1': 1, 'SP100': 1, 'STARD4-AS1': 1, 'TPT1-AS1': 1, 'TRAPPC3': 1, 'TRBV6-5': 1, 'TSSC2': 1, 'Y_RNA': 1, 'ZNF37BP': 1} | 33.75 | 4 |
| {'Eso': 2, 'Lymph': 1, 'Panc': 1} | {'TP53': 4, 'ID3': 1, 'CCND3': 1, 'P2RY8': 1, 'KRAS': 1, 'SMAD4': 1} | 2.25 | 4 |
| {'Eso': 2, 'Breast': 1} | {'TP53': 4, 'ERBB3': 1, 'RUNX1T1': 1, 'NRG1': 1} | 2.33333333 | 3 |
| {'Biliary': 1, 'Head': 1, 'CNS': 1} | {'TP53': 2, 'IKZF1': 2, 'ARID1A': 1} | 1.66666667 | 3 |
| {'Biliary': 1, 'Liver': 1, 'Eso': 1} | {'TP53': 3, 'SMAD4': 2, 'FLI1': 1, 'APC': 1} | 2.33333333 | 3 |
| {'Lymph': 3} | {'TMSB4X': 4, 'SGK1': 2, 'BTG1': 1, 'P2RY8': 1} | 2.66666667 | 3 |
| {'Lymph': 3} | {'BCL2': 7, 'MYD88': 3, 'EZH2': 2, 'ZFHX3': 1} | 4.33333333 | 3 |
| {'Eso': 2, 'Panc': 1} | {'TP53': 4, 'ARID1A': 2, 'KRAS': 1} | 2.33333333 | 3 |
| {'Lymph': 2, 'Panc': 1} | {'SETD2': 2, 'NOTCH1': 2} | 1.33333333 | 3 |
| {'Lymph': 3} | {'CARD11': 2, 'EZH2': 2, 'BCL2': 2} | 2 | 3 |
| {'Breast': 2} | {'PTEN': 2, 'SF3B1': 1, 'PIK3CA': 1, 'DCAF12L2': 1} | 2.5 | 2 |
| {'Skin': 1, 'Liver': 1} | {'CD209': 2, 'CNTNAP2': 1} | 1.5 | 2 |
| {'Skin': 1, 'Liver': 1} | {'DDR2': 2, 'PTPRT': 1} | 1.5 | 2 |
| {'Panc': 2} | {'BRAF': 2, 'TP53': 1, 'FAT4': 1} | 2 | 2 |
| {'Kidney': 2} | {'VHL': 2, 'H3F3A': 1} | 1.5 | 2 |
| {'Panc': 1, 'Eso': 1} | {'GNAS': 2, 'USP6': 1} | 1.5 | 2 |
| {'Biliary': 1, 'Prost': 1} | {'SMARCA4': 2, 'ACVR2A': 2, 'EXT1': 2, 'TP53': 1, 'GNAS': 1, 'RUNX1T1': 1, 'RALGDS': 1, 'LARP4B': 1, 'PTEN': 1, 'KDM5A': 1, 'MYO5A': 1, 'CIITA': 1, 'SPECC1': 1, 'STAT5B': 1, 'PPP2R1A': 1, 'ASXL2': 1, 'REL': 1, 'STAG1': 1, 'WWTR1': 1, 'LPP': 1, 'LEF1': 1, 'HNRNPA2B1': 1, 'CSMD3': 1, 'DDX3X': 1, 'BCORL1': 1} | 14 | 2 |
| {'Panc': 2} | {'MEN1': 2, 'PTEN': 1} | 1.5 | 2 |
| {'Panc': 2} | {'KRAS': 2, 'CDKN2A': 2, 'TP53': 1} | 2.5 | 2 |
| {'Lymph': 2} | {'BCL2': 9, 'TMSB4X': 3, 'STAT6': 1, 'HMGN2P46': 1, 'ZFHX3': 1, 'TP53': 1, 'SGK1': 1, 'FAM135B': 1} | 9 | 2 |
| {'Lymph': 2} | {'SOCS1': 4, 'BTG2': 1, 'BTG1': 1, 'ROBO2': 1, 'EBF1': 1, 'HLA-A': 1, 'PIM1': 1, 'TMSB4X': 1, 'VTI1A': 1} | 6 | 2 |

**Supplementary Table 5.** Edge list of Tissue – Gene bipartite network.

| **Edge list** |
| --- |
| ('A1CF', 'Skin') |
| ('A2ML1-AS1', 'Panc') |
| ('AACSP1', 'Panc') |
| ('AACSP1', 'Prost') |
| ('AB015752.3', 'Panc') |
| ('ABCA3', 'Prost') |
| ('ABCC4', 'Prost') |
| ('AC000370.2', 'Panc') |
| ('AC002485.1', 'Panc') |
| ('AC004041.2', 'Prost') |
| ('AC004053.1', 'Panc') |
| ('AC004053.1', 'Prost') |
| ('AC004458.1', 'Prost') |
| ('AC004460.1', 'Panc') |
| ('AC004485.3', 'Myeloid') |
| ('AC004538.3', 'Panc') |
| ('AC004538.3', 'Prost') |
| ('AC004673.1', 'Panc') |
| ('AC004791.2', 'Prost') |
| ('AC004791.2', 'Myeloid') |
| ('AC004862.6', 'Myeloid') |
| ('AC004901.1', 'Prost') |
| ('AC005062.2', 'Prost') |
| ('AC005592.2', 'Panc') |
| ('AC005592.2', 'Prost') |
| ('AC006322.1', 'Prost') |
| ('AC007040.8', 'CNS') |
| ('AC007128.1', 'Panc') |
| ('AC007277.3', 'Prost') |
| ('AC007319.1', 'Prost') |
| ('AC007879.5', 'Prost') |
| ('AC007970.1', 'Panc') |
| ('AC008836.1', 'Prost') |
| ('AC009120.6', 'Prost') |
| ('AC009236.1', 'Prost') |
| ('AC009236.1', 'Myeloid') |
| ('AC009312.1', 'Panc') |
| ('AC009677.1', 'Prost') |
| ('AC009950.2', 'Panc') |
| ('AC012363.4', 'Prost') |
| ('AC012501.2', 'Panc') |
| ('AC012501.2', 'Prost') |
| ('AC012671.1', 'Panc') |
| ('AC013463.2', 'Panc') |
| ('AC015922.5', 'Prost') |
| ('AC016723.4', 'Panc') |
| ('AC016725.4', 'Skin') |
| ('AC016725.4', 'Prost') |
| ('AC016907.3', 'Prost') |
| ('AC018737.1', 'Panc') |
| ('AC018890.6', 'Panc') |
| ('AC024560.3', 'Panc') |
| ('AC024908.1', 'Prost') |
| ('AC027612.2', 'Prost') |
| ('AC027612.3', 'Prost') |
| ('AC061961.2', 'Prost') |
| ('AC072062.1', 'Myeloid') |
| ('AC073479.1', 'Prost') |
| ('AC079135.1', 'Prost') |
| ('AC079305.8', 'Prost') |
| ('AC079610.1', 'Panc') |
| ('AC079610.1', 'Prost') |
| ('AC079613.1', 'Panc') |
| ('AC079613.1', 'Prost') |
| ('AC079630.4', 'Prost') |
| ('AC079756.1', 'Panc') |
| ('AC083906.1', 'Prost') |
| ('AC091320.2', 'Prost') |
| ('AC091736.1', 'Panc') |
| ('AC091878.1', 'Prost') |
| ('AC092048.1', 'Prost') |
| ('AC092846.2', 'Prost') |
| ('AC092965.1', 'Panc') |
| ('AC093391.2', 'Myeloid') |
| ('AC093874.1', 'Prost') |
| ('AC096579.13', 'Prost') |
| ('AC097467.2', 'Prost') |
| ('AC097467.2', 'Myeloid') |
| ('AC097468.7', 'Prost') |
| ('AC098617.1', 'Prost') |
| ('AC102953.4', 'Prost') |
| ('AC104434.1', 'Panc') |
| ('AC105245.1', 'Panc') |
| ('AC108142.1', 'Stomach') |
| ('AC108142.1', 'Panc') |
| ('AC108142.1', 'Myeloid') |
| ('AC108448.3', 'Panc') |
| ('AC108696.1', 'Panc') |
| ('AC108696.1', 'Prost') |
| ('AC110086.1', 'Prost') |
| ('AC126177.1', 'Panc') |
| ('AC129929.5', 'Panc') |
| ('AC131097.3', 'Panc') |
| ('AC136188.1', 'Myeloid') |
| ('AC136932.2', 'Myeloid') |
| ('AC138761.1', 'Prost') |
| ('AC144450.1', 'Myeloid') |
| ('AC144521.1', 'Panc') |
| ('AC144521.1', 'Prost') |
| ('ACE', 'Prost') |
| ('ACO1', 'Panc') |
| ('ACVR2A', 'Stomach') |
| ('ACVR2A', 'Panc') |
| ('ACVR2A', 'Biliary') |
| ('ADAL', 'Prost') |
| ('ADAM5', 'Panc') |
| ('ADAMTS9-AS2', 'Prost') |
| ('ADCY1', 'Prost') |
| ('ADD2', 'Panc') |
| ('ADPRHL2', 'Prost') |
| ('ADRBK2', 'Prost') |
| ('AE000661.37', 'Panc') |
| ('AF121898.3', 'Panc') |
| ('AF121898.3', 'Prost') |
| ('AF186996.1', 'Prost') |
| ('AFF1', 'Prost') |
| ('AFF3', 'Panc') |
| ('AFF3', 'Eso') |
| ('AFF4', 'Prost') |
| ('AJ003147.9', 'Panc') |
| ('AJAP1', 'Panc') |
| ('AKAP11', 'Lymph') |
| ('AKT1', 'Breast') |
| ('AKT1', 'Prost') |
| ('AL117380.1', 'Panc') |
| ('AL133247.2', 'Panc') |
| ('AL133247.2', 'Prost') |
| ('AL138963.1', 'Panc') |
| ('AL139815.1', 'Prost') |
| ('AL159977.1', 'Prost') |
| ('AL162511.1', 'Prost') |
| ('AL353791.1', 'Prost') |
| ('AL354806.1', 'Panc') |
| ('AL359709.2', 'Prost') |
| ('AL512640.1', 'Panc') |
| ('AL590726.1', 'Prost') |
| ('AL590874.1', 'Panc') |
| ('AL626787.1', 'Myeloid') |
| ('AL672294.1', 'Myeloid') |
| ('AL928742.2', 'Prost') |
| ('ALDH2', 'Prost') |
| ('ALDH9A1', 'Prost') |
| ('ALOX12P2', 'Myeloid') |
| ('AMER1', 'Skin') |
| ('ANGPTL7', 'Prost') |
| ('ANK1', 'Biliary') |
| ('ANK1', 'Skin') |
| ('ANK1', 'Liver') |
| ('ANK1', 'Eso') |
| ('ANKRD12', 'Prost') |
| ('ANKRD19P', 'Panc') |
| ('ANKRD20A1', 'Prost') |
| ('ANKRD26P1', 'Prost') |
| ('ANKRD6', 'Prost') |
| ('AOC1', 'Prost') |
| ('AP000320.7', 'Prost') |
| ('AP000640.2', 'Panc') |
| ('AP000765.1', 'Prost') |
| ('AP001525.1', 'Prost') |
| ('AP001596.6', 'Panc') |
| ('AP001597.1', 'Prost') |
| ('AP002954.4', 'Prost') |
| ('APC', 'Panc') |
| ('APC', 'Eso') |
| ('APC', 'Prost') |
| ('APOB', 'Liver') |
| ('AR', 'Eso') |
| ('AR', 'Stomach') |
| ('AR', 'Panc') |
| ('AR', 'Prost') |
| ('ARHGAP11B', 'Prost') |
| ('ARHGAP35', 'Panc') |
| ('ARHGAP40', 'Myeloid') |
| ('ARHGEF12', 'Skin') |
| ('ARHGEF26-AS1', 'Prost') |
| ('ARHGEF37', 'Prost') |
| ('ARHGEF40', 'Prost') |
| ('ARID1A', 'Eso') |
| ('ARID1A', 'Biliary') |
| ('ARID1A', 'Panc') |
| ('ARID1A', 'Prost') |
| ('ARID2', 'Skin') |
| ('ARID2', 'Liver') |
| ('ARID2', 'Biliary') |
| ('ARID2', 'CNS') |
| ('ARID2', 'Prost') |
| ('ARPC5', 'Panc') |
| ('ARSK', 'Panc') |
| ('ASNA1', 'Prost') |
| ('ASXL1', 'Myeloid') |
| ('ASXL2', 'Panc') |
| ('ASXL2', 'Biliary') |
| ('ASXL2', 'Prost') |
| ('ATF1', 'Skin') |
| ('ATG16L1', 'Panc') |
| ('ATG5', 'Prost') |
| ('ATM', 'Stomach') |
| ('ATM', 'Skin') |
| ('ATM', 'Prost') |
| ('ATM', 'Liver') |
| ('ATM', 'Eso') |
| ('ATM', 'Panc') |
| ('ATP2C1', 'Prost') |
| ('ATRX', 'Liver') |
| ('AXIN1', 'Skin') |
| ('B2M', 'Stomach') |
| ('B2M', 'Skin') |
| ('B2M', 'Panc') |
| ('B2M', 'Lymph') |
| ('B3GALTL', 'Panc') |
| ('BAGE2', 'Prost') |
| ('BAI3', 'Panc') |
| ('BAX', 'Ovary') |
| ('BAX', 'Panc') |
| ('BCL10', 'Skin') |
| ('BCL11A', 'Stomach') |
| ('BCL11A', 'Eso') |
| ('BCL11A', 'Prost') |
| ('BCL11B', 'Stomach') |
| ('BCL11B', 'Panc') |
| ('BCL11B', 'Liver') |
| ('BCL11B', 'Lymph') |
| ('BCL2', 'Lymph') |
| ('BCL6', 'Lymph') |
| ('BCL9', 'Panc') |
| ('BCL9', 'CNS') |
| ('BCL9', 'Prost') |
| ('BCORL1', 'Biliary') |
| ('BCORL1', 'Liver') |
| ('BCORP1', 'Prost') |
| ('BCR', 'Lymph') |
| ('BCR', 'Prost') |
| ('BDNF-AS', 'Panc') |
| ('BDNF-AS', 'Prost') |
| ('BEND4', 'Panc') |
| ('BIRC3', 'Prost') |
| ('BMP5', 'Skin') |
| ('BMP5', 'Eso') |
| ('BMPR1A', 'Panc') |
| ('BMPR1A', 'Liver') |
| ('BMS1P8', 'Panc') |
| ('BPIFA4P', 'Prost') |
| ('BRAF', 'Stomach') |
| ('BRAF', 'Skin') |
| ('BRAF', 'Lymph') |
| ('BRAF', 'Panc') |
| ('BRAF', 'CNS') |
| ('BRD3', 'Stomach') |
| ('BRINP2', 'Prost') |
| ('BTAF1', 'Panc') |
| ('BTG1', 'Lymph') |
| ('BTG2', 'Lymph') |
| ('BTG2', 'Liver') |
| ('BTG2', 'Myeloid') |
| ('C10orf12', 'Prost') |
| ('C11orf84', 'Prost') |
| ('C16orf62', 'Eso') |
| ('C17orf80', 'Prost') |
| ('C18orf8', 'Panc') |
| ('C19orf35', 'Prost') |
| ('C1orf132', 'Panc') |
| ('C2orf16', 'Prost') |
| ('C6', 'Prost') |
| ('CAB39L', 'Prost') |
| ('CACNA1C', 'Myeloid') |
| ('CAMSAP3', 'Panc') |
| ('CAMTA1', 'Stomach') |
| ('CARD11', 'Lymph') |
| ('CASC2', 'Panc') |
| ('CASC8', 'Prost') |
| ('CASKIN1', 'Panc') |
| ('CBFB', 'Breast') |
| ('CCNB1IP1', 'Liver') |
| ('CCNB1IP1', 'Prost') |
| ('CCND2', 'Skin') |
| ('CCND3', 'Lymph') |
| ('CCNL1', 'Panc') |
| ('CCR10', 'Panc') |
| ('CCR7', 'Panc') |
| ('CCT6P3', 'Panc') |
| ('CCT6P3', 'Prost') |
| ('CD209', 'Stomach') |
| ('CD209', 'Skin') |
| ('CD209', 'Panc') |
| ('CD209', 'Liver') |
| ('CD209', 'Eso') |
| ('CD209', 'Prost') |
| ('CD209', 'Bone') |
| ('CD28', 'Skin') |
| ('CD2AP', 'Prost') |
| ('CD79B', 'Lymph') |
| ('CDC20B', 'Panc') |
| ('CDC42SE2', 'Prost') |
| ('CDH1', 'Prost') |
| ('CDH10', 'Skin') |
| ('CDH10', 'Lymph') |
| ('CDH10', 'Prost') |
| ('CDH11', 'Skin') |
| ('CDH11', 'Liver') |
| ('CDH11', 'CNS') |
| ('CDH11', 'Eso') |
| ('CDH11', 'Prost') |
| ('CDH11', 'Lymph') |
| ('CDH23', 'Prost') |
| ('CDHR5', 'Prost') |
| ('CDK12', 'Prost') |
| ('CDKN2A', 'Head') |
| ('CDKN2A', 'Skin') |
| ('CDKN2A', 'Panc') |
| ('CDKN2A', 'Liver') |
| ('CDKN2A', 'Eso') |
| ('CDKN2A', 'Lymph') |
| ('CDKN2B-AS1', 'Prost') |
| ('CDPF1', 'Panc') |
| ('CEBPA', 'Prost') |
| ('CEP170P1', 'Panc') |
| ('CHD2', 'Prost') |
| ('CHIC1', 'Prost') |
| ('CHST11', 'Breast') |
| ('CHST11', 'Panc') |
| ('CIITA', 'Stomach') |
| ('CIITA', 'Biliary') |
| ('CLHC1', 'Panc') |
| ('CLRN1-AS1', 'Prost') |
| ('CMAHP', 'Myeloid') |
| ('CNGA1', 'Prost') |
| ('CNGB3', 'Panc') |
| ('CNNM3', 'Panc') |
| ('CNTNAP2', 'Skin') |
| ('CNTNAP2', 'Lymph') |
| ('CNTNAP2', 'Prost') |
| ('COL1A1', 'Prost') |
| ('COL24A1', 'Prost') |
| ('COL26A1', 'Prost') |
| ('COL2A1', 'Prost') |
| ('COL5A3', 'Prost') |
| ('COL6A1', 'Panc') |
| ('COL6A5', 'Panc') |
| ('COPA', 'Panc') |
| ('COPB1', 'Myeloid') |
| ('CPNE4', 'Prost') |
| ('CREB1', 'Prost') |
| ('CREBBP', 'Lymph') |
| ('CRNKL1', 'Skin') |
| ('CRNKL1', 'Prost') |
| ('CRYGEP', 'Panc') |
| ('CRYM', 'Myeloid') |
| ('CSDE1', 'Panc') |
| ('CSF1R', 'Skin') |
| ('CSMD1', 'Panc') |
| ('CSMD3', 'Head') |
| ('CSMD3', 'Stomach') |
| ('CSMD3', 'Skin') |
| ('CSMD3', 'Lymph') |
| ('CSMD3', 'Panc') |
| ('CSMD3', 'Biliary') |
| ('CSMD3', 'Eso') |
| ('CSMD3', 'Prost') |
| ('CST7', 'Panc') |
| ('CSTF3-AS1', 'Prost') |
| ('CTA-85E5.10', 'Liver') |
| ('CTB-118P15.2', 'Panc') |
| ('CTB-186H2.2', 'Prost') |
| ('CTB-49A3.2', 'Prost') |
| ('CTB-57H20.1', 'Myeloid') |
| ('CTBP1-AS2', 'Panc') |
| ('CTBP1-AS2', 'Prost') |
| ('CTBS', 'Panc') |
| ('CTC-260E6.6', 'Panc') |
| ('CTC-260E6.6', 'Prost') |
| ('CTC-297N7.11', 'Prost') |
| ('CTC-297N7.5', 'Prost') |
| ('CTC-340A15.2', 'Panc') |
| ('CTC-340A15.2', 'Prost') |
| ('CTC-457E21.6', 'Panc') |
| ('CTC-457E21.9', 'Prost') |
| ('CTC-525D6.1', 'Prost') |
| ('CTCF', 'Myeloid') |
| ('CTD-2001E22.1', 'Prost') |
| ('CTD-2014E2.5', 'Prost') |
| ('CTD-2015H6.3', 'Prost') |
| ('CTD-2021J15.1', 'Prost') |
| ('CTD-2058B24.2', 'Myeloid') |
| ('CTD-2176I21.2', 'Prost') |
| ('CTD-2194D22.1', 'Myeloid') |
| ('CTD-2203K17.1', 'Panc') |
| ('CTD-2215L10.1', 'Prost') |
| ('CTD-2269F5.1', 'Prost') |
| ('CTD-2288O8.1', 'Panc') |
| ('CTD-2307P3.1', 'Panc') |
| ('CTD-2307P3.1', 'Prost') |
| ('CTD-2547L16.1', 'Prost') |
| ('CTD-3006G17.2', 'Panc') |
| ('CTD-3006G17.2', 'Prost') |
| ('CTD-3088G3.8', 'Prost') |
| ('CTH', 'Panc') |
| ('CTNNA2', 'Skin') |
| ('CTNNA2', 'Panc') |
| ('CTNNA2', 'Eso') |
| ('CTNNA2', 'Prost') |
| ('CTNNA3', 'Prost') |
| ('CTNNB1', 'Stomach') |
| ('CTNNB1', 'Skin') |
| ('CTNNB1', 'Liver') |
| ('CTNNB1', 'CNS') |
| ('CTNNB1', 'Panc') |
| ('CTNNB1', 'Eso') |
| ('CTNNB1', 'Prost') |
| ('CTNND2', 'Skin') |
| ('CTNND2', 'Prost') |
| ('CTSL3P', 'Myeloid') |
| ('CTSS', 'Prost') |
| ('CTTNBP2', 'Prost') |
| ('CUL3', 'Skin') |
| ('CUL3', 'Prost') |
| ('CXXC1P1', 'Prost') |
| ('CXXC4', 'Prost') |
| ('CYCS', 'Panc') |
| ('CYLC2', 'Myeloid') |
| ('CYP4B1', 'Prost') |
| ('DARC', 'Prost') |
| ('DAXX', 'Panc') |
| ('DCAF12L2', 'Breast') |
| ('DCAF12L2', 'Panc') |
| ('DCAF12L2', 'Liver') |
| ('DCAF4L2', 'Prost') |
| ('DCC', 'Skin') |
| ('DCST2', 'Panc') |
| ('DCTN4', 'Panc') |
| ('DDR2', 'Skin') |
| ('DDR2', 'Panc') |
| ('DDR2', 'Liver') |
| ('DDX11-AS1', 'Prost') |
| ('DDX3X', 'Panc') |
| ('DDX3X', 'Biliary') |
| ('DDX3X', 'CNS') |
| ('DDX46', 'Skin') |
| ('DDX50', 'Prost') |
| ('DDX6', 'Prost') |
| ('DDX60', 'Prost') |
| ('DEAF1', 'Panc') |
| ('DGCR8', 'Prost') |
| ('DGKE', 'Panc') |
| ('DGKI', 'Panc') |
| ('DIAPH2-AS1', 'Skin') |
| ('DIO2-AS1', 'Prost') |
| ('DIRC3', 'Prost') |
| ('DISC1FP1', 'Panc') |
| ('DISC1FP1', 'Prost') |
| ('DLG2', 'Prost') |
| ('DNAJB7', 'Myeloid') |
| ('DNAJB9', 'Panc') |
| ('DNER', 'Panc') |
| ('DNMT3A', 'Myeloid') |
| ('DOCK5', 'Prost') |
| ('DSTN', 'Panc') |
| ('DTX4', 'Kidney') |
| ('DTX4', 'Panc') |
| ('DUOX2', 'Prost') |
| ('DUT', 'Panc') |
| ('DZIP1', 'Myeloid') |
| ('EBF1', 'Lymph') |
| ('EED', 'CNS') |
| ('EED', 'Lymph') |
| ('EFCAB1', 'Prost') |
| ('EGF', 'Panc') |
| ('EGFEM1P', 'Prost') |
| ('EHHADH-AS1', 'Prost') |
| ('EIF1AX', 'Prost') |
| ('EIF4A2', 'Panc') |
| ('EIF4E', 'Prost') |
| ('ELF3', 'Prost') |
| ('ELK4', 'Skin') |
| ('ELK4', 'Lymph') |
| ('ELL', 'Prost') |
| ('ELOVL2', 'Prost') |
| ('EMCN-IT3', 'Panc') |
| ('EMILIN1', 'Prost') |
| ('EML4', 'Prost') |
| ('EMR4P', 'Panc') |
| ('ENTPD3-AS1', 'Prost') |
| ('EOGT', 'Panc') |
| ('EPAS1', 'Myeloid') |
| ('EPHA1-AS1', 'Prost') |
| ('EPHA7', 'Skin') |
| ('EPHA7', 'Prost') |
| ('EPSTI1', 'Prost') |
| ('ERBB2', 'Eso') |
| ('ERBB3', 'Breast') |
| ('ERBB3', 'Eso') |
| ('ERBB4', 'Stomach') |
| ('ERBB4', 'Skin') |
| ('ERBB4', 'Liver') |
| ('ERBB4', 'Myeloid') |
| ('ERBB4', 'Eso') |
| ('ERBB4', 'Prost') |
| ('ERBB4', 'Lymph') |
| ('ERCC4', 'Skin') |
| ('ERICH1-AS1', 'Prost') |
| ('ERMP1', 'Prost') |
| ('ESPNP', 'Prost') |
| ('ESRRAP2', 'Prost') |
| ('EXT1', 'Stomach') |
| ('EXT1', 'Panc') |
| ('EXT1', 'Biliary') |
| ('EXT2', 'Prost') |
| ('EZH2', 'Skin') |
| ('EZH2', 'Lymph') |
| ('EZH2', 'Myeloid') |
| ('F11-AS1', 'Prost') |
| ('F11-AS1', 'Myeloid') |
| ('FAIM2', 'Prost') |
| ('FAM120AOS', 'Prost') |
| ('FAM135B', 'Eso') |
| ('FAM135B', 'Stomach') |
| ('FAM135B', 'Skin') |
| ('FAM135B', 'Lymph') |
| ('FAM135B', 'Ovary') |
| ('FAM135B', 'Prost') |
| ('FAM153B', 'Prost') |
| ('FAM154A', 'Prost') |
| ('FAM177A1', 'Prost') |
| ('FAM179A', 'Panc') |
| ('FAM181A-AS1', 'Prost') |
| ('FAM184B', 'Prost') |
| ('FAM210B', 'Panc') |
| ('FAM230B', 'Prost') |
| ('FAM27E3', 'Panc') |
| ('FAM3B', 'Panc') |
| ('FAM47C', 'Skin') |
| ('FAM47C', 'Panc') |
| ('FAM84B', 'Panc') |
| ('FAM86JP', 'Panc') |
| ('FARSB', 'Prost') |
| ('FAT1', 'Prost') |
| ('FAT1', 'Myeloid') |
| ('FAT3', 'Skin') |
| ('FAT3', 'Ovary') |
| ('FAT3', 'Prost') |
| ('FAT3', 'Myeloid') |
| ('FAT4', 'Panc') |
| ('FAT4', 'Ovary') |
| ('FAT4', 'Prost') |
| ('FBN1', 'Prost') |
| ('FBXL5', 'Prost') |
| ('FBXO11', 'Lymph') |
| ('FBXO28', 'Prost') |
| ('FBXW7', 'Stomach') |
| ('FBXW7', 'CNS') |
| ('FCRL3', 'Prost') |
| ('FETUB', 'Prost') |
| ('FGFR1', 'CNS') |
| ('FLG-AS1', 'Panc') |
| ('FLG-AS1', 'Prost') |
| ('FLI1', 'Biliary') |
| ('FLI1', 'Panc') |
| ('FLT4', 'Liver') |
| ('FLT4', 'Panc') |
| ('FNDC1', 'Prost') |
| ('FNDC3B', 'Prost') |
| ('FNIP2', 'Panc') |
| ('FOLH1B', 'Panc') |
| ('FOLH1B', 'Prost') |
| ('FOXA1', 'Prost') |
| ('FOXL2', 'Prost') |
| ('FOXL2', 'Lymph') |
| ('FOXO1', 'Eso') |
| ('FOXO1', 'Lymph') |
| ('FREM2', 'Panc') |
| ('FREM2', 'Prost') |
| ('FSIP2', 'Myeloid') |
| ('FZD1', 'Prost') |
| ('FZD10-AS1', 'Prost') |
| ('FZD3', 'Panc') |
| ('GABRB1', 'Panc') |
| ('GATA3', 'Breast') |
| ('GATA3', 'Panc') |
| ('GATAD2A', 'Prost') |
| ('GBA3', 'Panc') |
| ('GBA3', 'Prost') |
| ('GCM1', 'Panc') |
| ('GDF7', 'Panc') |
| ('GFRAL', 'Panc') |
| ('GGTA1P', 'Panc') |
| ('GJD2', 'Prost') |
| ('GLTSCR2', 'Panc') |
| ('GLUD1P2', 'Prost') |
| ('GNA11', 'Prost') |
| ('GNAS', 'Breast') |
| ('GNAS', 'Panc') |
| ('GNAS', 'Biliary') |
| ('GNAS', 'Ovary') |
| ('GNAS', 'Eso') |
| ('GOPC', 'Prost') |
| ('GPD2', 'Prost') |
| ('GPR133', 'Prost') |
| ('GPR156', 'Prost') |
| ('GPR158-AS1', 'Panc') |
| ('GPR4', 'Prost') |
| ('GPT', 'Prost') |
| ('GRID1', 'Prost') |
| ('GRIN2A', 'Prost') |
| ('GRIN2A', 'Skin') |
| ('GRIPAP1', 'Prost') |
| ('GRM3', 'Skin') |
| ('GRM6', 'Panc') |
| ('GRPEL1', 'Panc') |
| ('GS1-256O22.5', 'Panc') |
| ('GS1-256O22.5', 'Prost') |
| ('GS1-256O22.5', 'Myeloid') |
| ('GTF2A1', 'Panc') |
| ('GTF2H5', 'Prost') |
| ('GTPBP10', 'Panc') |
| ('GTPBP10', 'Prost') |
| ('GUCY1B2', 'Panc') |
| ('GUCY1B2', 'Prost') |
| ('GUSBP1', 'Prost') |
| ('H3F3A', 'Kidney') |
| ('H3F3A', 'Panc') |
| ('H3F3A', 'Breast') |
| ('H3F3A', 'Bone') |
| ('H3F3B', 'Bone') |
| ('HCG18', 'Panc') |
| ('HCG18', 'Prost') |
| ('HCN4', 'Prost') |
| ('HEATR6', 'Prost') |
| ('HEBP2', 'Prost') |
| ('HEG1', 'Prost') |
| ('HELQ', 'Myeloid') |
| ('HELZ2', 'Panc') |
| ('HHIPL1', 'Prost') |
| ('HIST1H1A', 'Prost') |
| ('HLA-A', 'Panc') |
| ('HLA-A', 'Liver') |
| ('HLA-A', 'Lymph') |
| ('HLA-AS1', 'Panc') |
| ('HLTF-AS1', 'Myeloid') |
| ('HMCN1', 'Liver') |
| ('HMGCS1', 'Prost') |
| ('HMGN2P46', 'Stomach') |
| ('HMGN2P46', 'Skin') |
| ('HMGN2P46', 'Panc') |
| ('HMGN2P46', 'Lymph') |
| ('HMX3', 'Prost') |
| ('HNF4A', 'Liver') |
| ('HNRNPA1P48', 'Prost') |
| ('HNRNPA2B1', 'Stomach') |
| ('HNRNPA2B1', 'Biliary') |
| ('HOOK3', 'Stomach') |
| ('HOOK3', 'Ovary') |
| ('HOXC11', 'Panc') |
| ('HOXC13', 'Prost') |
| ('HPN-AS1', 'Panc') |
| ('HS3ST1', 'Panc') |
| ('HSP90AA1', 'Prost') |
| ('HTR3B', 'Myeloid') |
| ('HTR5BP', 'Panc') |
| ('HTR5BP', 'Prost') |
| ('IBTK', 'Skin') |
| ('ID3', 'Lymph') |
| ('IDH1', 'Skin') |
| ('IDH1', 'Lymph') |
| ('IDH1', 'Biliary') |
| ('IDH1', 'CNS') |
| ('IDH1', 'Prost') |
| ('IDH2', 'Myeloid') |
| ('IFNG-AS1', 'Panc') |
| ('IFNG-AS1', 'Prost') |
| ('IGFBP7-AS1', 'Prost') |
| ('IGHD3-10', 'Prost') |
| ('IGHD3OR15-3B', 'Panc') |
| ('IGHJ6', 'Lymph') |
| ('IGHM', 'Lymph') |
| ('IGHM', 'Myeloid') |
| ('IGHV1-24', 'Prost') |
| ('IGHV2-5', 'Panc') |
| ('IGHV3OR16-13', 'Panc') |
| ('IGHV3OR16-13', 'Prost') |
| ('IGKJ3', 'Lymph') |
| ('IGKJ5', 'Lymph') |
| ('IGKV1-27', 'Prost') |
| ('IGKV1D-37', 'Prost') |
| ('IGLV1-51', 'Panc') |
| ('IGLV7-46', 'Prost') |
| ('IKZF1', 'Head') |
| ('IKZF1', 'Biliary') |
| ('IKZF1', 'Ovary') |
| ('IKZF1', 'CNS') |
| ('IKZF1', 'Prost') |
| ('IL12A-AS1', 'Panc') |
| ('IL12A-AS1', 'Prost') |
| ('IL1RAP', 'Panc') |
| ('IL21R-AS1', 'Panc') |
| ('IL6ST', 'Stomach') |
| ('IL6ST', 'Liver') |
| ('IL6ST', 'Prost') |
| ('ILDR2', 'Panc') |
| ('INHBA', 'Prost') |
| ('INHBA-AS1', 'Panc') |
| ('INHBA-AS1', 'Prost') |
| ('INTS4', 'Prost') |
| ('INTS4L1', 'Prost') |
| ('ISM2', 'Myeloid') |
| ('ITGAV', 'Panc') |
| ('ITGB2', 'Prost') |
| ('ITK', 'Myeloid') |
| ('JAG2', 'Panc') |
| ('JAK2', 'Myeloid') |
| ('JAK3', 'Breast') |
| ('JAK3', 'Ovary') |
| ('JAZF1', 'Skin') |
| ('KAT6A', 'Panc') |
| ('KAT6A', 'Prost') |
| ('KB-1562D12.1', 'Prost') |
| ('KCNB2', 'Prost') |
| ('KCNC1', 'Panc') |
| ('KCND1', 'Myeloid') |
| ('KCND2', 'Panc') |
| ('KCNJ5', 'Prost') |
| ('KCNMB3P1', 'Prost') |
| ('KCNRG', 'Panc') |
| ('KCNV1', 'Prost') |
| ('KDM5A', 'Stomach') |
| ('KDM5A', 'Biliary') |
| ('KDM5A', 'Prost') |
| ('KDM6A', 'Prost') |
| ('KDR', 'Myeloid') |
| ('KEAP1', 'Skin') |
| ('KHDRBS3', 'Prost') |
| ('KIAA0513', 'Prost') |
| ('KIAA1257', 'Prost') |
| ('KIAA1549', 'Skin') |
| ('KIAA1549', 'Lymph') |
| ('KIAA1549', 'Prost') |
| ('KIAA1683', 'Prost') |
| ('KIAA2022', 'Prost') |
| ('KIF1A', 'Prost') |
| ('KIF25', 'Prost') |
| ('KIF9-AS1', 'Prost') |
| ('KIRREL3-AS3', 'Prost') |
| ('KIT', 'Prost') |
| ('KIT', 'Panc') |
| ('KIT', 'Eso') |
| ('KLF11', 'Prost') |
| ('KLF6', 'Myeloid') |
| ('KLHL3', 'Prost') |
| ('KLK2', 'Skin') |
| ('KLK2', 'Prost') |
| ('KLK2', 'Liver') |
| ('KLK2', 'Panc') |
| ('KMT2A', 'CNS') |
| ('KMT2A', 'Prost') |
| ('KMT2C', 'Panc') |
| ('KMT2C', 'Prost') |
| ('KMT2D', 'Skin') |
| ('KMT2D', 'Lymph') |
| ('KMT2D', 'Liver') |
| ('KMT2D', 'CNS') |
| ('KNSTRN', 'Skin') |
| ('KNTC1', 'Prost') |
| ('KRAS', 'Biliary') |
| ('KRAS', 'Head') |
| ('KRAS', 'Stomach') |
| ('KRAS', 'Panc') |
| ('KRAS', 'Ovary') |
| ('KRAS', 'Liver') |
| ('KRAS', 'Eso') |
| ('KTN1', 'Prost') |
| ('LAMA2', 'Panc') |
| ('LARP4B', 'Stomach') |
| ('LARP4B', 'Panc') |
| ('LARP4B', 'Biliary') |
| ('LCP1', 'Prost') |
| ('LEF1', 'Panc') |
| ('LEF1', 'Biliary') |
| ('LEF1', 'Prost') |
| ('LEF1-AS1', 'Prost') |
| ('LEPROTL1', 'Breast') |
| ('LIFR', 'Skin') |
| ('LIFR-AS1', 'Panc') |
| ('LINC00189', 'Panc') |
| ('LINC00189', 'Prost') |
| ('LINC00395', 'Prost') |
| ('LINC00467', 'Prost') |
| ('LINC00475', 'Panc') |
| ('LINC00535', 'Panc') |
| ('LINC00535', 'Prost') |
| ('LINC00535', 'Myeloid') |
| ('LINC00634', 'Panc') |
| ('LINC00649', 'Liver') |
| ('LINC00963', 'Prost') |
| ('LINC01088', 'Prost') |
| ('LL09NC01-254D11.1', 'Prost') |
| ('LLNLF-65H9.1', 'Panc') |
| ('LMCD1-AS1', 'Prost') |
| ('LMCD1-AS1', 'Myeloid') |
| ('LMNA', 'Prost') |
| ('LMTK2', 'Prost') |
| ('LPP', 'Biliary') |
| ('LPP', 'Liver') |
| ('LRP1B', 'Skin') |
| ('LRP1B', 'Ovary') |
| ('LRP1B', 'Liver') |
| ('LRP1B', 'Eso') |
| ('LRP1B', 'Lymph') |
| ('LRP3', 'Prost') |
| ('LRRC37A16P', 'Panc') |
| ('LRRC49', 'Prost') |
| ('LRRC4C', 'Ovary') |
| ('LRRIQ1', 'Panc') |
| ('LYL1', 'Panc') |
| ('LYL1', 'Eso') |
| ('MACC1', 'Skin') |
| ('MACC1', 'Prost') |
| ('MACC1', 'Myeloid') |
| ('MAF', 'Liver') |
| ('MAF', 'Eso') |
| ('MAF', 'Prost') |
| ('MAGI3', 'Prost') |
| ('MALT1', 'Skin') |
| ('MAMDC2', 'Prost') |
| ('MAP3K13', 'Prost') |
| ('MAX', 'CNS') |
| ('MAX', 'Panc') |
| ('MB21D2', 'Prost') |
| ('MCC', 'Prost') |
| ('MCHR2-AS1', 'Prost') |
| ('MCHR2-AS1', 'Myeloid') |
| ('MECOM', 'Skin') |
| ('MECOM', 'Eso') |
| ('MECOM', 'Myeloid') |
| ('MED12', 'Panc') |
| ('MED12', 'Prost') |
| ('MED15P9', 'Panc') |
| ('MEF2C-AS1', 'Panc') |
| ('MEF2C-AS1', 'Liver') |
| ('MEF2C-AS1', 'Prost') |
| ('MEGF10', 'Panc') |
| ('MEN1', 'Panc') |
| ('MEPCE', 'Skin') |
| ('MGAM', 'Panc') |
| ('MIR1268A', 'Panc') |
| ('MIR184', 'Prost') |
| ('MIR31HG', 'Prost') |
| ('MIR3622A', 'Prost') |
| ('MIR378D1', 'Myeloid') |
| ('MIR4431', 'Panc') |
| ('MIR4454', 'Panc') |
| ('MIR4472-1', 'Panc') |
| ('MIR496', 'Prost') |
| ('MIR518D', 'Prost') |
| ('MIR526B', 'Prost') |
| ('MIR548H4', 'Prost') |
| ('MIR596', 'Prost') |
| ('MITF', 'Myeloid') |
| ('MKLN1-AS1', 'Panc') |
| ('MKLN1-AS1', 'Prost') |
| ('MLH1', 'Skin') |
| ('MLK7-AS1', 'Prost') |
| ('MLLT3', 'Breast') |
| ('MLLT3', 'Panc') |
| ('MLLT3', 'Prost') |
| ('MMP8', 'Prost') |
| ('MRPL45P2', 'Prost') |
| ('MSH6', 'Skin') |
| ('MSLN', 'Prost') |
| ('MTHFD2P1', 'Prost') |
| ('MTMR9LP', 'Panc') |
| ('MTOR', 'Lymph') |
| ('MTOR', 'Kidney') |
| ('MTRNR2L5', 'Prost') |
| ('MTRNR2L7', 'Panc') |
| ('MTSS1L', 'Prost') |
| ('MUC16', 'Skin') |
| ('MUC16', 'Lymph') |
| ('MUC16', 'Panc') |
| ('MUC16', 'Prost') |
| ('MUC16', 'Kidney') |
| ('MUC16', 'Liver') |
| ('MUC16', 'Eso') |
| ('MUC16', 'Myeloid') |
| ('MUC4', 'Prost') |
| ('MUC4', 'Myeloid') |
| ('MYC', 'Lymph') |
| ('MYD88', 'Stomach') |
| ('MYD88', 'Lymph') |
| ('MYO18A', 'Prost') |
| ('MYO5A', 'Panc') |
| ('MYO5A', 'Biliary') |
| ('MYO5A', 'Liver') |
| ('Metazoa_SRP', 'Panc') |
| ('N4BP2', 'Stomach') |
| ('N4BP2', 'Panc') |
| ('NABP1', 'Panc') |
| ('NALCN-AS1', 'Panc') |
| ('NALCN-AS1', 'Prost') |
| ('NANOS2', 'Liver') |
| ('NBEA', 'Skin') |
| ('NBEA', 'Prost') |
| ('NCLN', 'Panc') |
| ('NDC80', 'Panc') |
| ('NELFA', 'Prost') |
| ('NF1', 'Stomach') |
| ('NF1', 'Prost') |
| ('NF1', 'Myeloid') |
| ('NF2', 'Prost') |
| ('NFATC3', 'Panc') |
| ('NFKBID', 'Panc') |
| ('NFKBIE', 'Skin') |
| ('NFKBIE', 'Lymph') |
| ('NFYB', 'Myeloid') |
| ('NHLH1', 'Prost') |
| ('NLGN4X', 'Myeloid') |
| ('NONO', 'Skin') |
| ('NONO', 'Panc') |
| ('NONO', 'Liver') |
| ('NOTCH1', 'Lymph') |
| ('NOTCH2', 'Lymph') |
| ('NOTCH2', 'Myeloid') |
| ('NOVA1-AS1', 'Prost') |
| ('NPAS2', 'Panc') |
| ('NPM1', 'Prost') |
| ('NPSR1-AS1', 'Panc') |
| ('NPSR1-AS1', 'Prost') |
| ('NR2F2-AS1', 'Myeloid') |
| ('NRAS', 'Skin') |
| ('NRAS', 'Myeloid') |
| ('NRAS', 'Liver') |
| ('NRAS', 'Panc') |
| ('NRAS', 'Lymph') |
| ('NREP-AS1', 'Prost') |
| ('NRG1', 'Eso') |
| ('NRG1', 'Stomach') |
| ('NRG1', 'Prost') |
| ('NRGN', 'Prost') |
| ('NSD1', 'Prost') |
| ('NT5DC4', 'Prost') |
| ('NTF4', 'Panc') |
| ('NUFIP1', 'Panc') |
| ('NUP188', 'Panc') |
| ('NUP214', 'Liver') |
| ('NUP214', 'Prost') |
| ('OIT3', 'Prost') |
| ('OR14A2', 'Prost') |
| ('OR2H1', 'Panc') |
| ('OR52M1', 'Panc') |
| ('OR5D18', 'Prost') |
| ('OR8B8', 'Prost') |
| ('OTX2-AS1', 'Panc') |
| ('OTX2-AS1', 'Myeloid') |
| ('P2RY2', 'Prost') |
| ('P2RY8', 'Lymph') |
| ('PABPC1', 'Panc') |
| ('PAFAH1B2', 'Skin') |
| ('PAIP2B', 'Panc') |
| ('PARP4', 'Panc') |
| ('PASD1', 'Prost') |
| ('PAX5', 'Skin') |
| ('PAX5', 'Liver') |
| ('PAX5', 'Lymph') |
| ('PBRM1', 'Stomach') |
| ('PBRM1', 'Panc') |
| ('PBRM1', 'Kidney') |
| ('PBRM1', 'Prost') |
| ('PBX1', 'Eso') |
| ('PBX1', 'Skin') |
| ('PBX1', 'Liver') |
| ('PCBP1-AS1', 'Prost') |
| ('PCDHA5', 'Panc') |
| ('PCDHB3', 'Prost') |
| ('PCDHGA6', 'Panc') |
| ('PDE10A', 'Prost') |
| ('PDE3A', 'Prost') |
| ('PDE4B', 'Skin') |
| ('PDE4DIP', 'Skin') |
| ('PDE4DIP', 'Panc') |
| ('PDE4DIP', 'Prost') |
| ('PDGFRB', 'Prost') |
| ('PEG10', 'Prost') |
| ('PEX7', 'Prost') |
| ('PGM5P2', 'Skin') |
| ('PHF6', 'Myeloid') |
| ('PHOX2B', 'Stomach') |
| ('PHOX2B', 'Lymph') |
| ('PHOX2B', 'Liver') |
| ('PHOX2B', 'Eso') |
| ('PI3', 'Prost') |
| ('PICALM', 'Liver') |
| ('PICALM', 'Panc') |
| ('PICK1', 'Prost') |
| ('PIK3CA', 'Breast') |
| ('PIK3CA', 'Head') |
| ('PIK3CA', 'Stomach') |
| ('PIK3CA', 'Panc') |
| ('PIK3CA', 'CNS') |
| ('PIK3CA', 'Liver') |
| ('PIK3CA', 'Biliary') |
| ('PIK3CA', 'Eso') |
| ('PIK3CA', 'Prost') |
| ('PIK3CG', 'Prost') |
| ('PIM1', 'Lymph') |
| ('PKD1L1', 'Panc') |
| ('PKD1L3', 'Panc') |
| ('PLEKHA8P1', 'Panc') |
| ('PLEKHG6', 'Kidney') |
| ('PMS2', 'Prost') |
| ('PMS2CL', 'Prost') |
| ('PNMA1', 'Prost') |
| ('PNMA5', 'Panc') |
| ('POT1', 'Skin') |
| ('POU2AF1', 'Skin') |
| ('PPFIA1', 'Prost') |
| ('PPIP5K2', 'Prost') |
| ('PPP1R12C', 'Myeloid') |
| ('PPP1R15A', 'Panc') |
| ('PPP1R26-AS1', 'Myeloid') |
| ('PPP1R35', 'Prost') |
| ('PPP2R1A', 'Stomach') |
| ('PPP2R1A', 'Biliary') |
| ('PPP2R2A', 'Liver') |
| ('PPP6C', 'Skin') |
| ('PRCC', 'Prost') |
| ('PREX2', 'Skin') |
| ('PREX2', 'Liver') |
| ('PREX2', 'Panc') |
| ('PREX2', 'Eso') |
| ('PRKAR1A', 'Myeloid') |
| ('PRKCA', 'Panc') |
| ('PRKD1', 'Skin') |
| ('PRKY', 'Myeloid') |
| ('PROM2', 'Myeloid') |
| ('PROX1-AS1', 'Panc') |
| ('PROX1-AS1', 'Prost') |
| ('PRPF40B', 'Panc') |
| ('PRRC2C', 'Breast') |
| ('PRRX1', 'Skin') |
| ('PRRX1', 'Prost') |
| ('PSTK', 'Prost') |
| ('PTEN', 'Breast') |
| ('PTEN', 'Skin') |
| ('PTEN', 'Biliary') |
| ('PTEN', 'Panc') |
| ('PTEN', 'CNS') |
| ('PTEN', 'Eso') |
| ('PTEN', 'Prost') |
| ('PTEN', 'Bone') |
| ('PTK6', 'Prost') |
| ('PTPN13', 'Panc') |
| ('PTPRB', 'Skin') |
| ('PTPRC', 'Skin') |
| ('PTPRD', 'Skin') |
| ('PTPRT', 'Skin') |
| ('PTPRT', 'Panc') |
| ('PTPRT', 'Eso') |
| ('PTPRT', 'Prost') |
| ('PUM1', 'Myeloid') |
| ('PVRL4', 'Prost') |
| ('PWWP2A', 'Liver') |
| ('QKI', 'Prost') |
| ('RABEP1', 'Prost') |
| ('RABGEF1', 'Panc') |
| ('RAD1', 'Prost') |
| ('RAET1K', 'Myeloid') |
| ('RALGDS', 'Biliary') |
| ('RALGDS', 'Lymph') |
| ('RAPGEF4-AS1', 'Panc') |
| ('RAPGEFL1', 'Prost') |
| ('RASA2', 'Prost') |
| ('RASGRP1', 'Panc') |
| ('RB1', 'Prost') |
| ('RB1', 'Breast') |
| ('RBFOX2', 'Prost') |
| ('RBM10', 'Biliary') |
| ('RBM10', 'Panc') |
| ('RBM10', 'Myeloid') |
| ('RBM15', 'Skin') |
| ('REL', 'Biliary') |
| ('REL', 'Ovary') |
| ('REL', 'Prost') |
| ('REXO1L1', 'Panc') |
| ('RFESD', 'Myeloid') |
| ('RFTN1P1', 'Prost') |
| ('RGS18', 'Prost') |
| ('RGS7', 'Skin') |
| ('RHBDL3', 'Prost') |
| ('RHOA', 'Lymph') |
| ('RHOU', 'Panc') |
| ('RMI2', 'Prost') |
| ('RN7SKP104', 'Kidney') |
| ('RN7SKP149', 'Panc') |
| ('RN7SKP227', 'Prost') |
| ('RN7SL139P', 'Panc') |
| ('RN7SL170P', 'Prost') |
| ('RN7SL251P', 'Prost') |
| ('RN7SL278P', 'Panc') |
| ('RN7SL300P', 'Panc') |
| ('RN7SL33P', 'Panc') |
| ('RN7SL357P', 'Prost') |
| ('RN7SL564P', 'Prost') |
| ('RN7SL572P', 'Prost') |
| ('RN7SL575P', 'Prost') |
| ('RN7SL600P', 'Prost') |
| ('RN7SL637P', 'Prost') |
| ('RN7SL644P', 'Panc') |
| ('RN7SL649P', 'Prost') |
| ('RN7SL659P', 'Prost') |
| ('RN7SL7P', 'Prost') |
| ('RN7SL865P', 'Panc') |
| ('RN7SL89P', 'Prost') |
| ('RNA5SP116', 'Prost') |
| ('RNA5SP24', 'Panc') |
| ('RNA5SP358', 'Prost') |
| ('RNA5SP36', 'Prost') |
| ('RNA5SP403', 'Prost') |
| ('RNA5SP459', 'Myeloid') |
| ('RNA5SP474', 'Panc') |
| ('RNA5SP504', 'Prost') |
| ('RNA5SP508', 'Panc') |
| ('RNA5SP62', 'Prost') |
| ('RNF215', 'Panc') |
| ('RNF217', 'Prost') |
| ('RNF219-AS1', 'Panc') |
| ('RNF219-AS1', 'Prost') |
| ('RNF43', 'Stomach') |
| ('RNF43', 'Skin') |
| ('RNMT', 'Prost') |
| ('RNPC3', 'Prost') |
| ('RNU1-59P', 'Prost') |
| ('RNU2-54P', 'Panc') |
| ('RNU4-57P', 'Prost') |
| ('RNU4-65P', 'Prost') |
| ('RNU6-102P', 'Prost') |
| ('RNU6-1047P', 'Prost') |
| ('RNU6-1070P', 'Prost') |
| ('RNU6-1163P', 'Panc') |
| ('RNU6-133P', 'Panc') |
| ('RNU6-204P', 'Panc') |
| ('RNU6-227P', 'Panc') |
| ('RNU6-243P', 'Prost') |
| ('RNU6-439P', 'Panc') |
| ('RNU6-442P', 'Prost') |
| ('RNU6-52P', 'Prost') |
| ('RNU6-533P', 'Panc') |
| ('RNU6-543P', 'Prost') |
| ('RNU6-656P', 'Prost') |
| ('RNU6-741P', 'Prost') |
| ('RNU7-136P', 'Prost') |
| ('RNU7-174P', 'Panc') |
| ('RNU7-24P', 'Panc') |
| ('RNY4P27', 'Prost') |
| ('ROBO2', 'Skin') |
| ('ROBO2', 'CNS') |
| ('ROBO2', 'Liver') |
| ('ROBO2', 'Eso') |
| ('ROBO2', 'Prost') |
| ('ROBO2', 'Lymph') |
| ('ROS1', 'Myeloid') |
| ('RP1-13P20.6', 'Panc') |
| ('RP1-167A14.2', 'Panc') |
| ('RP1-167F1.2', 'Prost') |
| ('RP1-23K20.2', 'Panc') |
| ('RP1-240B8.3', 'Prost') |
| ('RP1-261D10.2', 'Panc') |
| ('RP1-272J12.1', 'Panc') |
| ('RP1-272J12.1', 'Myeloid') |
| ('RP1-28O10.1', 'Prost') |
| ('RP1-65P5.1', 'Prost') |
| ('RP1-91G5.3', 'Prost') |
| ('RP1-93I3.1', 'Prost') |
| ('RP11-100L22.2', 'Prost') |
| ('RP11-1017G21.4', 'Prost') |
| ('RP11-103J8.1', 'Panc') |
| ('RP11-1069G10.1', 'Prost') |
| ('RP11-1084E5.1', 'Prost') |
| ('RP11-1103G16.1', 'Ovary') |
| ('RP11-1103G16.1', 'Prost') |
| ('RP11-1129I3.1', 'Panc') |
| ('RP11-1129I3.1', 'Prost') |
| ('RP11-114H24.5', 'Panc') |
| ('RP11-115D19.1', 'Panc') |
| ('RP11-115D19.1', 'Prost') |
| ('RP11-122D10.1', 'Myeloid') |
| ('RP11-125B21.2', 'Prost') |
| ('RP11-1277A3.2', 'Prost') |
| ('RP11-130F10.1', 'Panc') |
| ('RP11-135J2.4', 'Myeloid') |
| ('RP11-13G14.4', 'Prost') |
| ('RP11-13J10.1', 'Panc') |
| ('RP11-13J10.1', 'Prost') |
| ('RP11-142C4.6', 'Prost') |
| ('RP11-144L1.4', 'Prost') |
| ('RP11-145A3.1', 'Prost') |
| ('RP11-150C16.1', 'Prost') |
| ('RP11-152L20.3', 'Panc') |
| ('RP11-152L20.3', 'Prost') |
| ('RP11-152L20.3', 'Myeloid') |
| ('RP11-152P17.2', 'Panc') |
| ('RP11-154D6.1', 'Prost') |
| ('RP11-154D6.1', 'Myeloid') |
| ('RP11-155G15.2', 'Myeloid') |
| ('RP11-158J3.2', 'Prost') |
| ('RP11-159L20.2', 'Prost') |
| ('RP11-15B17.1', 'Panc') |
| ('RP11-163M18.1', 'Prost') |
| ('RP11-166B2.8', 'Prost') |
| ('RP11-167H9.4', 'Panc') |
| ('RP11-167N24.3', 'Prost') |
| ('RP11-175P19.2', 'Prost') |
| ('RP11-17A1.3', 'Prost') |
| ('RP11-17E2.2', 'Prost') |
| ('RP11-17E2.2', 'Myeloid') |
| ('RP11-184A2.3', 'Myeloid') |
| ('RP11-193H5.1', 'Panc') |
| ('RP11-196E1.3', 'Prost') |
| ('RP11-1L12.3', 'Panc') |
| ('RP11-230B22.1', 'Panc') |
| ('RP11-231I13.2', 'Skin') |
| ('RP11-231I13.2', 'Panc') |
| ('RP11-231I13.2', 'Prost') |
| ('RP11-231P20.2', 'Prost') |
| ('RP11-23D24.2', 'Prost') |
| ('RP11-274B21.1', 'Panc') |
| ('RP11-274B21.1', 'Prost') |
| ('RP11-280O1.2', 'Prost') |
| ('RP11-284M14.1', 'Panc') |
| ('RP11-285J16.1', 'Prost') |
| ('RP11-286H14.4', 'Panc') |
| ('RP11-290F24.3', 'Panc') |
| ('RP11-310I9.1', 'Myeloid') |
| ('RP11-313J2.1', 'Panc') |
| ('RP11-324H6.5', 'Prost') |
| ('RP11-326E22.1', 'Prost') |
| ('RP11-32K4.1', 'Panc') |
| ('RP11-32K4.1', 'Prost') |
| ('RP11-32K4.1', 'Myeloid') |
| ('RP11-333E1.1', 'Panc') |
| ('RP11-33N16.3', 'Panc') |
| ('RP11-33N16.3', 'Prost') |
| ('RP11-342D14.1', 'Panc') |
| ('RP11-342D14.1', 'Prost') |
| ('RP11-343D2.11', 'Prost') |
| ('RP11-356I2.4', 'Panc') |
| ('RP11-357C3.3', 'Prost') |
| ('RP11-366F6.2', 'Prost') |
| ('RP11-368J21.3', 'Panc') |
| ('RP11-368M16.3', 'Prost') |
| ('RP11-370I10.6', 'Prost') |
| ('RP11-379F4.4', 'Prost') |
| ('RP11-380P13.1', 'Panc') |
| ('RP11-382A20.4', 'Panc') |
| ('RP11-385J1.2', 'Prost') |
| ('RP11-3B12.1', 'Prost') |
| ('RP11-3B12.1', 'Myeloid') |
| ('RP11-402C9.1', 'Panc') |
| ('RP11-418J17.1', 'Panc') |
| ('RP11-420N3.2', 'Panc') |
| ('RP11-420N3.2', 'Prost') |
| ('RP11-420N3.2', 'Myeloid') |
| ('RP11-422J8.1', 'Prost') |
| ('RP11-423H2.3', 'Prost') |
| ('RP11-428C19.4', 'Panc') |
| ('RP11-429B14.1', 'Prost') |
| ('RP11-431M7.2', 'Prost') |
| ('RP11-431M7.3', 'Prost') |
| ('RP11-435M3.2', 'Prost') |
| ('RP11-438D8.2', 'Panc') |
| ('RP11-439L18.3', 'Prost') |
| ('RP11-445F12.1', 'Prost') |
| ('RP11-446J8.1', 'Prost') |
| ('RP11-454C18.2', 'Prost') |
| ('RP11-482H16.1', 'Myeloid') |
| ('RP11-492A10.1', 'Myeloid') |
| ('RP11-505P4.7', 'Prost') |
| ('RP11-508N22.8', 'Prost') |
| ('RP11-513G19.1', 'Prost') |
| ('RP11-519G16.3', 'Prost') |
| ('RP11-521M14.1', 'Panc') |
| ('RP11-525K10.3', 'Panc') |
| ('RP11-525K10.3', 'Prost') |
| ('RP11-526D8.7', 'Prost') |
| ('RP11-532F12.5', 'Prost') |
| ('RP11-538P18.2', 'Prost') |
| ('RP11-541P9.3', 'Liver') |
| ('RP11-541P9.3', 'Prost') |
| ('RP11-542K23.7', 'Myeloid') |
| ('RP11-54D18.3', 'Prost') |
| ('RP11-550I24.2', 'Prost') |
| ('RP11-550P17.5', 'Prost') |
| ('RP11-551L14.1', 'Prost') |
| ('RP11-556E13.1', 'Panc') |
| ('RP11-556E13.1', 'Prost') |
| ('RP11-562L8.1', 'Panc') |
| ('RP11-562L8.1', 'Prost') |
| ('RP11-572M11.4', 'Panc') |
| ('RP11-572M11.4', 'Prost') |
| ('RP11-57H12.5', 'Panc') |
| ('RP11-586K2.1', 'Panc') |
| ('RP11-586K2.1', 'Prost') |
| ('RP11-58B2.1', 'Prost') |
| ('RP11-611E13.2', 'Prost') |
| ('RP11-624C23.1', 'Panc') |
| ('RP11-624C23.1', 'Prost') |
| ('RP11-624L4.1', 'Prost') |
| ('RP11-631F7.1', 'Myeloid') |
| ('RP11-634B7.4', 'Panc') |
| ('RP11-634B7.4', 'Prost') |
| ('RP11-644F5.16', 'Prost') |
| ('RP11-649A16.1', 'Panc') |
| ('RP11-649A16.1', 'Prost') |
| ('RP11-657O9.1', 'Prost') |
| ('RP11-665G4.1', 'Panc') |
| ('RP11-665G4.1', 'Prost') |
| ('RP11-66D17.5', 'Prost') |
| ('RP11-678G14.2', 'Prost') |
| ('RP11-679C8.2', 'Panc') |
| ('RP11-679C8.2', 'Prost') |
| ('RP11-680B3.2', 'Panc') |
| ('RP11-680F20.9', 'Panc') |
| ('RP11-692D12.1', 'Prost') |
| ('RP11-693J15.4', 'Panc') |
| ('RP11-702H23.4', 'Prost') |
| ('RP11-706C16.8', 'Prost') |
| ('RP11-707M1.1', 'Panc') |
| ('RP11-707M1.1', 'Prost') |
| ('RP11-708B6.2', 'Panc') |
| ('RP11-708B6.2', 'Prost') |
| ('RP11-731J8.2', 'Myeloid') |
| ('RP11-742B18.1', 'Panc') |
| ('RP11-742B18.1', 'Prost') |
| ('RP11-744N12.3', 'Prost') |
| ('RP11-752G15.9', 'Prost') |
| ('RP11-75N4.2', 'Prost') |
| ('RP11-76N22.2', 'Prost') |
| ('RP11-770E5.1', 'Panc') |
| ('RP11-770E5.1', 'Prost') |
| ('RP11-77K12.5', 'Panc') |
| ('RP11-788M5.3', 'Prost') |
| ('RP11-800A3.4', 'Panc') |
| ('RP11-804A23.1', 'Panc') |
| ('RP11-804A23.2', 'Kidney') |
| ('RP11-804A23.2', 'Prost') |
| ('RP11-804N13.1', 'Prost') |
| ('RP11-81K13.1', 'Myeloid') |
| ('RP11-820L6.1', 'Prost') |
| ('RP11-826N14.1', 'Prost') |
| ('RP11-846C15.2', 'Panc') |
| ('RP11-84A1.3', 'Panc') |
| ('RP11-867G2.8', 'Panc') |
| ('RP11-86L19.2', 'Prost') |
| ('RP11-87M18.2', 'Panc') |
| ('RP11-89K10.1', 'Panc') |
| ('RP11-89K10.1', 'Prost') |
| ('RP11-92C4.3', 'Prost') |
| ('RP11-933H2.4', 'Panc') |
| ('RP11-93K22.6', 'Prost') |
| ('RP11-944L7.4', 'Myeloid') |
| ('RP11-978I15.10', 'Prost') |
| ('RP11-98D18.15', 'Prost') |
| ('RP13-492C18.2', 'Prost') |
| ('RP13-578N3.3', 'Prost') |
| ('RP3-323P13.2', 'Prost') |
| ('RP3-340N1.5', 'Prost') |
| ('RP3-399L15.3', 'Prost') |
| ('RP3-428L16.1', 'Panc') |
| ('RP4-605O3.4', 'Panc') |
| ('RP4-630C24.3', 'Prost') |
| ('RP4-651E10.4', 'Prost') |
| ('RP4-694A7.4', 'Prost') |
| ('RP4-724E13.2', 'Panc') |
| ('RP4-735C1.4', 'Prost') |
| ('RP4-755D9.1', 'Panc') |
| ('RP4-756H11.3', 'Myeloid') |
| ('RP4-777D9.2', 'Prost') |
| ('RP4-798P15.3', 'Panc') |
| ('RP5-1091N2.9', 'Panc') |
| ('RP5-1121H13.4', 'Prost') |
| ('RP5-896L10.1', 'Prost') |
| ('RP5-905H7.3', 'Prost') |
| ('RP5-921G16.1', 'Panc') |
| ('RP5-921G16.1', 'Myeloid') |
| ('RP5-991G20.1', 'Prost') |
| ('RPA3-AS1', 'Prost') |
| ('RPL10', 'Lymph') |
| ('RPL22', 'Stomach') |
| ('RPL22', 'Panc') |
| ('RPL22', 'Biliary') |
| ('RPL22', 'Liver') |
| ('RPL23AP79', 'Prost') |
| ('RUNDC1', 'Prost') |
| ('RUNX1T1', 'Breast') |
| ('RUNX1T1', 'Skin') |
| ('RUNX1T1', 'Lymph') |
| ('RUNX1T1', 'Liver') |
| ('RUNX1T1', 'Biliary') |
| ('RUNX1T1', 'Eso') |
| ('RUNX1T1', 'Prost') |
| ('RUSC2', 'Liver') |
| ('SAGE1', 'Prost') |
| ('SCARNA17', 'Panc') |
| ('SCN10A', 'Panc') |
| ('SCN4A', 'Prost') |
| ('SCRG1', 'Panc') |
| ('SCRN1', 'Prost') |
| ('SDHA', 'Prost') |
| ('SDHA', 'Panc') |
| ('SEC14L4', 'Prost') |
| ('SEC24B-AS1', 'Prost') |
| ('SEC24B-AS1', 'Myeloid') |
| ('SEC63', 'Panc') |
| ('SEMA5A', 'Panc') |
| ('SEPT14', 'Prost') |
| ('SERPINB11', 'Myeloid') |
| ('SERPINB9', 'Prost') |
| ('SETBP1', 'Skin') |
| ('SETBP1', 'CNS') |
| ('SETBP1', 'Eso') |
| ('SETBP1', 'Prost') |
| ('SETBP1', 'Myeloid') |
| ('SETD2', 'Panc') |
| ('SETD2', 'Lymph') |
| ('SF3B1', 'Breast') |
| ('SF3B1', 'Panc') |
| ('SF3B1', 'Prost') |
| ('SF3B1', 'Biliary') |
| ('SF3B1', 'Eso') |
| ('SF3B1', 'Liver') |
| ('SF3B1', 'Lymph') |
| ('SFRP4', 'Prost') |
| ('SFXN1', 'Panc') |
| ('SGK1', 'Lymph') |
| ('SGK1', 'Prost') |
| ('SGK223', 'Myeloid') |
| ('SH2B3', 'Myeloid') |
| ('SHANK2', 'Prost') |
| ('SHANK3', 'Panc') |
| ('SIGLEC6', 'Prost') |
| ('SIGLEC9', 'Panc') |
| ('SIGLECL1', 'Panc') |
| ('SIX1', 'Stomach') |
| ('SIX1', 'Skin') |
| ('SLAIN2', 'Panc') |
| ('SLAMF6', 'Prost') |
| ('SLC10A7', 'Prost') |
| ('SLC15A1', 'Prost') |
| ('SLC16A10', 'Prost') |
| ('SLC18A2', 'Prost') |
| ('SLC24A5', 'Skin') |
| ('SLC25A32', 'Prost') |
| ('SLC25A38', 'Prost') |
| ('SLC25A44', 'Panc') |
| ('SLC26A2', 'Prost') |
| ('SLC30A2', 'Prost') |
| ('SLC31A1', 'Prost') |
| ('SLC46A1', 'Panc') |
| ('SLC46A1', 'Prost') |
| ('SLC5A3', 'Prost') |
| ('SLC7A14', 'Prost') |
| ('SLC8A1', 'Prost') |
| ('SLC8A1-AS1', 'Prost') |
| ('SLFN5', 'Prost') |
| ('SMAD2', 'Skin') |
| ('SMAD2', 'Liver') |
| ('SMAD4', 'Biliary') |
| ('SMAD4', 'Panc') |
| ('SMAD4', 'Eso') |
| ('SMARCA2', 'Panc') |
| ('SMARCA4', 'Breast') |
| ('SMARCA4', 'Skin') |
| ('SMARCA4', 'Lymph') |
| ('SMARCA4', 'Panc') |
| ('SMARCA4', 'CNS') |
| ('SMARCA4', 'Biliary') |
| ('SMARCA4', 'Eso') |
| ('SMARCA4', 'Prost') |
| ('SMARCA4', 'Myeloid') |
| ('SMARCD2', 'Skin') |
| ('SMO', 'CNS') |
| ('SNAPC1', 'Prost') |
| ('SND1', 'Skin') |
| ('SND1', 'Liver') |
| ('SNHG11', 'Panc') |
| ('SNHG14', 'Panc') |
| ('SNORA26', 'Prost') |
| ('SNORA31', 'Myeloid') |
| ('SNORA51', 'Prost') |
| ('SNORA63', 'Kidney') |
| ('SNORD2', 'Prost') |
| ('SNORD29', 'Prost') |
| ('SNORD81', 'Myeloid') |
| ('SNRPD2', 'Prost') |
| ('SNX29', 'Prost') |
| ('SNX7', 'Panc') |
| ('SOCS1', 'Lymph') |
| ('SORL1', 'Prost') |
| ('SOX21', 'Prost') |
| ('SP100', 'Prost') |
| ('SPANXA2-OT1', 'Prost') |
| ('SPATA3', 'Panc') |
| ('SPECC1', 'Stomach') |
| ('SPECC1', 'Biliary') |
| ('SPECC1', 'Prost') |
| ('SPEN', 'Myeloid') |
| ('SPHK1', 'Panc') |
| ('SPOP', 'Prost') |
| ('SPTBN4', 'Myeloid') |
| ('SRD5A2', 'Prost') |
| ('SRD5A3-AS1', 'Prost') |
| ('SRGAP2-AS1', 'Panc') |
| ('SRGAP2B', 'Prost') |
| ('SRGAP3', 'Skin') |
| ('SSPO', 'Panc') |
| ('SSPO', 'Prost') |
| ('STAG1', 'Liver') |
| ('STAG1', 'Biliary') |
| ('STAG2', 'Prost') |
| ('STARD13', 'Prost') |
| ('STARD4-AS1', 'Prost') |
| ('STAT5B', 'Biliary') |
| ('STAT5B', 'Panc') |
| ('STAT6', 'Lymph') |
| ('STAT6', 'Prost') |
| ('STEAP2-AS1', 'Panc') |
| ('STEAP2-AS1', 'Myeloid') |
| ('STIL', 'Prost') |
| ('STXBP5-AS1', 'Panc') |
| ('SUCO', 'Prost') |
| ('SUGT1P3', 'Panc') |
| ('SULT1C2P1', 'Myeloid') |
| ('SUPT20H', 'Prost') |
| ('SUPT6H', 'Myeloid') |
| ('SUSD2', 'Panc') |
| ('SVILP1', 'Panc') |
| ('SWT1', 'Panc') |
| ('SYT4', 'Myeloid') |
| ('TAL1', 'Head') |
| ('TAL1', 'Ovary') |
| ('TAOK3', 'Prost') |
| ('TBL1XR1', 'Breast') |
| ('TBL1XR1', 'Liver') |
| ('TBL1XR1', 'Prost') |
| ('TCF12', 'Prost') |
| ('TCF21', 'Prost') |
| ('TCF7L2', 'Skin') |
| ('TCF7L2', 'Liver') |
| ('TCHH', 'Prost') |
| ('TCL1A', 'Skin') |
| ('TECTA', 'Panc') |
| ('TENC1', 'Prost') |
| ('TERT', 'Head') |
| ('TERT', 'Skin') |
| ('TET2', 'Prost') |
| ('TET2', 'Myeloid') |
| ('TEX26-AS1', 'Prost') |
| ('TFAP2B', 'Prost') |
| ('TFAP2C', 'Panc') |
| ('TFEB', 'Prost') |
| ('TFEB', 'Lymph') |
| ('TG', 'Myeloid') |
| ('TGFBR2', 'Eso') |
| ('TGFBR2', 'Breast') |
| ('TGFBR2', 'Lymph') |
| ('TGFBR2', 'Panc') |
| ('TGFBR2', 'Prost') |
| ('TGS1', 'Prost') |
| ('THEMIS2', 'Panc') |
| ('THSD1', 'Prost') |
| ('TLE3', 'Panc') |
| ('TLR8-AS1', 'Panc') |
| ('TM4SF1-AS1', 'Myeloid') |
| ('TM9SF4', 'Skin') |
| ('TMEM121', 'Skin') |
| ('TMEM132B', 'Myeloid') |
| ('TMEM154', 'Prost') |
| ('TMEM161B-AS1', 'Panc') |
| ('TMEM200C', 'Prost') |
| ('TMEM230', 'Panc') |
| ('TMEM254-AS1', 'Prost') |
| ('TMEM38B', 'Prost') |
| ('TMPRSS2', 'Prost') |
| ('TMPRSS4-AS1', 'Prost') |
| ('TMSB4X', 'Lymph') |
| ('TNRC18', 'Myeloid') |
| ('TNS3', 'Prost') |
| ('TOP3B', 'Panc') |
| ('TP53', 'Breast') |
| ('TP53', 'Prost') |
| ('TP53', 'Eso') |
| ('TP53', 'Stomach') |
| ('TP53', 'Biliary') |
| ('TP53', 'Head') |
| ('TP53', 'Skin') |
| ('TP53', 'Panc') |
| ('TP53', 'Liver') |
| ('TP53', 'Lymph') |
| ('TP53', 'Ovary') |
| ('TP53', 'Kidney') |
| ('TP53', 'Bone') |
| ('TP53', 'Myeloid') |
| ('TPT1-AS1', 'Prost') |
| ('TPTE2P1', 'Prost') |
| ('TPTE2P1', 'Myeloid') |
| ('TRAPPC3', 'Prost') |
| ('TRAPPC3L', 'Prost') |
| ('TRAV27', 'Prost') |
| ('TRBV28', 'Panc') |
| ('TRBV6-5', 'Prost') |
| ('TRBV6-8', 'Prost') |
| ('TREML3P', 'Panc') |
| ('TRGC2', 'Myeloid') |
| ('TRIM3', 'Panc') |
| ('TRIM33', 'Prost') |
| ('TRIM69', 'Prost') |
| ('TRPC5', 'Myeloid') |
| ('TRPM1', 'Skin') |
| ('TRPM6', 'Panc') |
| ('TRRAP', 'Skin') |
| ('TRRAP', 'Biliary') |
| ('TRRAP', 'CNS') |
| ('TSC1', 'Myeloid') |
| ('TSPAN32', 'Panc') |
| ('TSSC2', 'Prost') |
| ('TTN', 'Prost') |
| ('TYW1B', 'Panc') |
| ('TYW1B', 'Prost') |
| ('U2AF1', 'Biliary') |
| ('U2AF1', 'Liver') |
| ('U2AF1', 'Panc') |
| ('U2AF1', 'Myeloid') |
| ('U3', 'Panc') |
| ('U3', 'Prost') |
| ('U8', 'Prost') |
| ('UBE2Q2', 'Prost') |
| ('UCHL1-AS1', 'Myeloid') |
| ('UCK1', 'Panc') |
| ('UFL1-AS1', 'Prost') |
| ('UGDH-AS1', 'Panc') |
| ('UHRF1', 'Panc') |
| ('UMODL1', 'Prost') |
| ('UNC5B', 'Panc') |
| ('UNG', 'Prost') |
| ('URB1', 'Prost') |
| ('USP15', 'Prost') |
| ('USP2-AS1', 'Panc') |
| ('USP45', 'Biliary') |
| ('USP6', 'Breast') |
| ('USP6', 'Head') |
| ('USP6', 'Skin') |
| ('USP6', 'Eso') |
| ('USP8', 'Prost') |
| ('UVRAG', 'Prost') |
| ('VAV1', 'Prost') |
| ('VHL', 'Kidney') |
| ('VNN2', 'Prost') |
| ('VPS37A', 'Panc') |
| ('VSTM4', 'Prost') |
| ('VTI1A', 'Stomach') |
| ('VTI1A', 'Lymph') |
| ('VTI1B', 'Prost') |
| ('VWA3B', 'Myeloid') |
| ('WDR11-AS1', 'Panc') |
| ('WDR5B', 'Prost') |
| ('WDR74', 'Prost') |
| ('WDR83OS', 'Panc') |
| ('WIF1', 'Lymph') |
| ('WIF1', 'Eso') |
| ('WNK2', 'Prost') |
| ('WSCD1', 'Panc') |
| ('WWTR1', 'Biliary') |
| ('WWTR1', 'Lymph') |
| ('XKR8', 'Panc') |
| ('XPO1', 'Lymph') |
| ('XRCC5', 'Prost') |
| ('Y_RNA', 'Panc') |
| ('Y_RNA', 'Prost') |
| ('Y_RNA', 'Myeloid') |
| ('Z93241.1', 'Panc') |
| ('Z95704.4', 'Prost') |
| ('ZBED3-AS1', 'Prost') |
| ('ZBED5-AS1', 'Prost') |
| ('ZBTB38', 'Prost') |
| ('ZBTB7C', 'Myeloid') |
| ('ZC3H11A', 'Panc') |
| ('ZCCHC11', 'Myeloid') |
| ('ZCCHC4', 'Prost') |
| ('ZCCHC8', 'Prost') |
| ('ZFHX3', 'Skin') |
| ('ZFHX3', 'Lymph') |
| ('ZFHX3', 'Kidney') |
| ('ZFHX3', 'Panc') |
| ('ZFHX3', 'Prost') |
| ('ZFHX4', 'Panc') |
| ('ZFHX4-AS1', 'Panc') |
| ('ZFHX4-AS1', 'Prost') |
| ('ZFYVE21', 'Prost') |
| ('ZNF106', 'Prost') |
| ('ZNF20', 'Myeloid') |
| ('ZNF207', 'Prost') |
| ('ZNF222', 'Myeloid') |
| ('ZNF341', 'Prost') |
| ('ZNF37BP', 'Prost') |
| ('ZNF384', 'Prost') |
| ('ZNF521', 'Panc') |
| ('ZNF521', 'Eso') |
| ('ZNF527', 'Prost') |
| ('ZNF665', 'Prost') |
| ('ZNF75D', 'Panc') |
| ('ZNF815P', 'Prost') |
| ('ZNF827', 'Prost') |
| ('ZNF844', 'Prost') |
| ('ZNRD1-AS1', 'Prost') |
| ('ZPLD1', 'Ovary') |
| ('ZRANB2-AS1', 'Prost') |
| ('ZRANB2-AS2', 'Prost') |
| ('hsa-mir-490', 'Panc') |
| ('hsa-mir-490', 'Prost') |
| ('snoU13', 'Panc') |
| ('snoU13', 'Prost') |
| ('snoU13', 'Myeloid') |

**Supplementary Table 6.** Edge list of Sample – Gene bipartite network.

| **Edge list** |
| --- |
| ('A1CF', 'DO220890') |
| ('A1CF', 'DO220902') |
| ('A2ML1-AS1', 'DO49454') |
| ('AACSP1', 'DO49442') |
| ('AACSP1', 'DO51954') |
| ('AB015752.3', 'DO49442') |
| ('ABCA3', 'DO52510') |
| ('ABCC4', 'DO51964') |
| ('AC000370.2', 'DO49454') |
| ('AC002485.1', 'DO49442') |
| ('AC004041.2', 'DO51964') |
| ('AC004053.1', 'DO49442') |
| ('AC004053.1', 'DO51954') |
| ('AC004458.1', 'DO51964') |
| ('AC004460.1', 'DO49454') |
| ('AC004485.3', 'DO52736') |
| ('AC004538.3', 'DO49454') |
| ('AC004538.3', 'DO51964') |
| ('AC004673.1', 'DO49454') |
| ('AC004791.2', 'DO51954') |
| ('AC004791.2', 'DO52743') |
| ('AC004862.6', 'DO52736') |
| ('AC004901.1', 'DO51954') |
| ('AC005062.2', 'DO51964') |
| ('AC005592.2', 'DO49454') |
| ('AC005592.2', 'DO51964') |
| ('AC006322.1', 'DO51964') |
| ('AC007040.8', 'DO35544') |
| ('AC007128.1', 'DO49454') |
| ('AC007277.3', 'DO51954') |
| ('AC007277.3', 'DO51964') |
| ('AC007319.1', 'DO51964') |
| ('AC007879.5', 'DO51954') |
| ('AC007879.5', 'DO51964') |
| ('AC007970.1', 'DO49454') |
| ('AC008836.1', 'DO51964') |
| ('AC009120.6', 'DO51954') |
| ('AC009236.1', 'DO51964') |
| ('AC009236.1', 'DO52743') |
| ('AC009312.1', 'DO49442') |
| ('AC009677.1', 'DO51964') |
| ('AC009950.2', 'DO49454') |
| ('AC012363.4', 'DO51964') |
| ('AC012501.2', 'DO49442') |
| ('AC012501.2', 'DO51954') |
| ('AC012671.1', 'DO49442') |
| ('AC013463.2', 'DO49442') |
| ('AC015922.5', 'DO51954') |
| ('AC016723.4', 'DO49454') |
| ('AC016725.4', 'DO220852') |
| ('AC016725.4', 'DO51964') |
| ('AC016907.3', 'DO51954') |
| ('AC016907.3', 'DO52510') |
| ('AC018737.1', 'DO49442') |
| ('AC018890.6', 'DO49442') |
| ('AC024560.3', 'DO49442') |
| ('AC024908.1', 'DO51954') |
| ('AC027612.2', 'DO52512') |
| ('AC027612.3', 'DO51954') |
| ('AC061961.2', 'DO51954') |
| ('AC072062.1', 'DO52743') |
| ('AC073479.1', 'DO51964') |
| ('AC079135.1', 'DO51964') |
| ('AC079305.8', 'DO52512') |
| ('AC079610.1', 'DO49442') |
| ('AC079610.1', 'DO51954') |
| ('AC079610.1', 'DO51964') |
| ('AC079610.1', 'DO52510') |
| ('AC079613.1', 'DO49442') |
| ('AC079613.1', 'DO51964') |
| ('AC079630.4', 'DO51964') |
| ('AC079756.1', 'DO49442') |
| ('AC083906.1', 'DO51964') |
| ('AC091320.2', 'DO52510') |
| ('AC091736.1', 'DO49442') |
| ('AC091878.1', 'DO51954') |
| ('AC092048.1', 'DO52510') |
| ('AC092846.2', 'DO51964') |
| ('AC092965.1', 'DO49454') |
| ('AC093391.2', 'DO52737') |
| ('AC093874.1', 'DO51964') |
| ('AC096579.13', 'DO51964') |
| ('AC097467.2', 'DO51954') |
| ('AC097467.2', 'DO51964') |
| ('AC097467.2', 'DO52736') |
| ('AC097468.7', 'DO51954') |
| ('AC098617.1', 'DO51964') |
| ('AC102953.4', 'DO51954') |
| ('AC104434.1', 'DO49442') |
| ('AC105245.1', 'DO49454') |
| ('AC108142.1', 'DO217814') |
| ('AC108142.1', 'DO49442') |
| ('AC108142.1', 'DO49454') |
| ('AC108142.1', 'DO52743') |
| ('AC108448.3', 'DO49442') |
| ('AC108696.1', 'DO49442') |
| ('AC108696.1', 'DO51964') |
| ('AC110086.1', 'DO52512') |
| ('AC126177.1', 'DO49454') |
| ('AC129929.5', 'DO49442') |
| ('AC131097.3', 'DO49442') |
| ('AC136188.1', 'DO52743') |
| ('AC136932.2', 'DO52743') |
| ('AC138761.1', 'DO51964') |
| ('AC144450.1', 'DO52743') |
| ('AC144521.1', 'DO49442') |
| ('AC144521.1', 'DO49454') |
| ('AC144521.1', 'DO51954') |
| ('ACE', 'DO51954') |
| ('ACO1', 'DO49454') |
| ('ACVR2A', 'DO218693') |
| ('ACVR2A', 'DO33152') |
| ('ACVR2A', 'DO35083') |
| ('ACVR2A', 'DO45299') |
| ('ADAL', 'DO51964') |
| ('ADAM5', 'DO49454') |
| ('ADAMTS9-AS2', 'DO52510') |
| ('ADCY1', 'DO51954') |
| ('ADD2', 'DO49442') |
| ('ADPRHL2', 'DO51954') |
| ('ADRBK2', 'DO51954') |
| ('AE000661.37', 'DO49454') |
| ('AF121898.3', 'DO49454') |
| ('AF121898.3', 'DO51964') |
| ('AF186996.1', 'DO51954') |
| ('AFF1', 'DO52511') |
| ('AFF3', 'DO221545') |
| ('AFF3', 'DO50382') |
| ('AFF4', 'DO51955') |
| ('AJ003147.9', 'DO49442') |
| ('AJAP1', 'DO49442') |
| ('AKAP11', 'DO52693') |
| ('AKT1', 'DO1017') |
| ('AKT1', 'DO218065') |
| ('AKT1', 'DO218560') |
| ('AKT1', 'DO220823') |
| ('AKT1', 'DO51962') |
| ('AL117380.1', 'DO49442') |
| ('AL133247.2', 'DO49442') |
| ('AL133247.2', 'DO51954') |
| ('AL138963.1', 'DO49442') |
| ('AL139815.1', 'DO51964') |
| ('AL159977.1', 'DO51954') |
| ('AL162511.1', 'DO51954') |
| ('AL353791.1', 'DO51954') |
| ('AL354806.1', 'DO49454') |
| ('AL359709.2', 'DO51964') |
| ('AL512640.1', 'DO49454') |
| ('AL590726.1', 'DO51954') |
| ('AL590874.1', 'DO49442') |
| ('AL626787.1', 'DO52737') |
| ('AL672294.1', 'DO52737') |
| ('AL928742.2', 'DO51954') |
| ('ALDH2', 'DO51965') |
| ('ALDH9A1', 'DO51964') |
| ('ALOX12P2', 'DO52737') |
| ('AMER1', 'DO220894') |
| ('AMER1', 'DO220902') |
| ('ANGPTL7', 'DO51954') |
| ('ANK1', 'DO218411') |
| ('ANK1', 'DO220899') |
| ('ANK1', 'DO45071') |
| ('ANK1', 'DO45153') |
| ('ANK1', 'DO50446') |
| ('ANK1', 'DO50447') |
| ('ANKRD12', 'DO51964') |
| ('ANKRD19P', 'DO49442') |
| ('ANKRD20A1', 'DO51954') |
| ('ANKRD26P1', 'DO51954') |
| ('ANKRD6', 'DO51954') |
| ('AOC1', 'DO51954') |
| ('AP000320.7', 'DO51954') |
| ('AP000320.7', 'DO51964') |
| ('AP000640.2', 'DO49442') |
| ('AP000765.1', 'DO51954') |
| ('AP001525.1', 'DO51954') |
| ('AP001596.6', 'DO49454') |
| ('AP001597.1', 'DO51964') |
| ('AP002954.4', 'DO51954') |
| ('APC', 'DO49418') |
| ('APC', 'DO50332') |
| ('APC', 'DO50342') |
| ('APC', 'DO50393') |
| ('APC', 'DO50411') |
| ('APC', 'DO50419') |
| ('APC', 'DO50445') |
| ('APC', 'DO50446') |
| ('APC', 'DO52131') |
| ('APOB', 'DO50851') |
| ('AR', 'DO10841') |
| ('AR', 'DO218693') |
| ('AR', 'DO50326') |
| ('AR', 'DO50340') |
| ('AR', 'DO50342') |
| ('AR', 'DO51510') |
| ('AR', 'DO51954') |
| ('AR', 'DO51960') |
| ('AR', 'DO51964') |
| ('ARHGAP11B', 'DO51964') |
| ('ARHGAP35', 'DO49454') |
| ('ARHGAP40', 'DO52737') |
| ('ARHGEF12', 'DO220857') |
| ('ARHGEF12', 'DO220890') |
| ('ARHGEF26-AS1', 'DO51954') |
| ('ARHGEF37', 'DO51954') |
| ('ARHGEF40', 'DO51964') |
| ('ARID1A', 'DO10840') |
| ('ARID1A', 'DO218227') |
| ('ARID1A', 'DO218695') |
| ('ARID1A', 'DO34905') |
| ('ARID1A', 'DO50326') |
| ('ARID1A', 'DO50381') |
| ('ARID1A', 'DO50382') |
| ('ARID1A', 'DO50440') |
| ('ARID1A', 'DO51538') |
| ('ARID1A', 'DO51962') |
| ('ARID2', 'DO220844') |
| ('ARID2', 'DO45199') |
| ('ARID2', 'DO45259') |
| ('ARID2', 'DO45277') |
| ('ARID2', 'DO48939') |
| ('ARID2', 'DO51965') |
| ('ARPC5', 'DO49442') |
| ('ARSK', 'DO49454') |
| ('ASNA1', 'DO51964') |
| ('ASXL1', 'DO52739') |
| ('ASXL1', 'DO52751') |
| ('ASXL2', 'DO35083') |
| ('ASXL2', 'DO45299') |
| ('ASXL2', 'DO51956') |
| ('ASXL2', 'DO51960') |
| ('ASXL2', 'DO51962') |
| ('ATF1', 'DO220889') |
| ('ATF1', 'DO220903') |
| ('ATG16L1', 'DO49454') |
| ('ATG5', 'DO51964') |
| ('ATM', 'DO218693') |
| ('ATM', 'DO220878') |
| ('ATM', 'DO220906') |
| ('ATM', 'DO36223') |
| ('ATM', 'DO45096') |
| ('ATM', 'DO50406') |
| ('ATM', 'DO51187') |
| ('ATP2C1', 'DO52510') |
| ('ATRX', 'DO45197') |
| ('ATRX', 'DO50809') |
| ('AXIN1', 'DO220886') |
| ('AXIN1', 'DO220903') |
| ('B2M', 'DO218769') |
| ('B2M', 'DO220873') |
| ('B2M', 'DO221541') |
| ('B2M', 'DO27851') |
| ('B2M', 'DO52652') |
| ('B2M', 'DO52655') |
| ('B2M', 'DO52663') |
| ('B2M', 'DO52672') |
| ('B3GALTL', 'DO49442') |
| ('BAGE2', 'DO51954') |
| ('BAGE2', 'DO51964') |
| ('BAI3', 'DO49454') |
| ('BAX', 'DO46591') |
| ('BAX', 'DO49418') |
| ('BAX', 'DO51497') |
| ('BCL10', 'DO220857') |
| ('BCL10', 'DO220903') |
| ('BCL11A', 'DO222299') |
| ('BCL11A', 'DO50346') |
| ('BCL11A', 'DO52512') |
| ('BCL11B', 'DO218693') |
| ('BCL11B', 'DO35083') |
| ('BCL11B', 'DO48682') |
| ('BCL11B', 'DO52685') |
| ('BCL2', 'DO221124') |
| ('BCL2', 'DO27773') |
| ('BCL2', 'DO27785') |
| ('BCL2', 'DO27787') |
| ('BCL2', 'DO27797') |
| ('BCL2', 'DO27801') |
| ('BCL2', 'DO27803') |
| ('BCL2', 'DO27805') |
| ('BCL2', 'DO27809') |
| ('BCL2', 'DO27817') |
| ('BCL2', 'DO27835') |
| ('BCL2', 'DO27837') |
| ('BCL2', 'DO27857') |
| ('BCL2', 'DO27859') |
| ('BCL2', 'DO52647') |
| ('BCL2', 'DO52650') |
| ('BCL2', 'DO52651') |
| ('BCL2', 'DO52652') |
| ('BCL2', 'DO52653') |
| ('BCL2', 'DO52654') |
| ('BCL2', 'DO52655') |
| ('BCL2', 'DO52656') |
| ('BCL2', 'DO52662') |
| ('BCL2', 'DO52663') |
| ('BCL2', 'DO52664') |
| ('BCL2', 'DO52667') |
| ('BCL2', 'DO52669') |
| ('BCL2', 'DO52671') |
| ('BCL2', 'DO52677') |
| ('BCL2', 'DO52681') |
| ('BCL2', 'DO52685') |
| ('BCL2', 'DO52687') |
| ('BCL2', 'DO52688') |
| ('BCL2', 'DO52689') |
| ('BCL2', 'DO52690') |
| ('BCL2', 'DO52693') |
| ('BCL2', 'DO52694') |
| ('BCL2', 'DO52695') |
| ('BCL2', 'DO52704') |
| ('BCL2', 'DO52718') |
| ('BCL2', 'DO6432') |
| ('BCL2', 'DO7112') |
| ('BCL6', 'DO221123') |
| ('BCL6', 'DO27767') |
| ('BCL6', 'DO27779') |
| ('BCL6', 'DO27783') |
| ('BCL6', 'DO27811') |
| ('BCL6', 'DO27853') |
| ('BCL6', 'DO52650') |
| ('BCL6', 'DO52663') |
| ('BCL9', 'DO35083') |
| ('BCL9', 'DO48914') |
| ('BCL9', 'DO51953') |
| ('BCORL1', 'DO45299') |
| ('BCORL1', 'DO48682') |
| ('BCORP1', 'DO51954') |
| ('BCR', 'DO221124') |
| ('BCR', 'DO51964') |
| ('BCR', 'DO52682') |
| ('BDNF-AS', 'DO49442') |
| ('BDNF-AS', 'DO52510') |
| ('BEND4', 'DO49454') |
| ('BIRC3', 'DO52512') |
| ('BMP5', 'DO220886') |
| ('BMP5', 'DO50315') |
| ('BMP5', 'DO50447') |
| ('BMP5', 'DO50452') |
| ('BMPR1A', 'DO35442') |
| ('BMPR1A', 'DO48682') |
| ('BMS1P8', 'DO49442') |
| ('BPIFA4P', 'DO51964') |
| ('BRAF', 'DO218693') |
| ('BRAF', 'DO220845') |
| ('BRAF', 'DO220847') |
| ('BRAF', 'DO220848') |
| ('BRAF', 'DO220855') |
| ('BRAF', 'DO220858') |
| ('BRAF', 'DO220863') |
| ('BRAF', 'DO220868') |
| ('BRAF', 'DO220869') |
| ('BRAF', 'DO220872') |
| ('BRAF', 'DO220874') |
| ('BRAF', 'DO220879') |
| ('BRAF', 'DO220881') |
| ('BRAF', 'DO220883') |
| ('BRAF', 'DO220887') |
| ('BRAF', 'DO220892') |
| ('BRAF', 'DO220894') |
| ('BRAF', 'DO220895') |
| ('BRAF', 'DO220897') |
| ('BRAF', 'DO220898') |
| ('BRAF', 'DO220900') |
| ('BRAF', 'DO220901') |
| ('BRAF', 'DO220902') |
| ('BRAF', 'DO220903') |
| ('BRAF', 'DO220905') |
| ('BRAF', 'DO220907') |
| ('BRAF', 'DO220909') |
| ('BRAF', 'DO220910') |
| ('BRAF', 'DO220911') |
| ('BRAF', 'DO220912') |
| ('BRAF', 'DO221123') |
| ('BRAF', 'DO34432') |
| ('BRAF', 'DO34504') |
| ('BRAF', 'DO35138') |
| ('BRAF', 'DO35994') |
| ('BRAF', 'DO36147') |
| ('BRAF', 'DO36163') |
| ('BRAF', 'DO51474') |
| ('BRD3', 'DO218031') |
| ('BRD3', 'DO218243') |
| ('BRINP2', 'DO51954') |
| ('BTAF1', 'DO49442') |
| ('BTG1', 'DO221123') |
| ('BTG1', 'DO27855') |
| ('BTG1', 'DO52675') |
| ('BTG1', 'DO52679') |
| ('BTG2', 'DO27811') |
| ('BTG2', 'DO48723') |
| ('BTG2', 'DO52675') |
| ('BTG2', 'DO52692') |
| ('BTG2', 'DO52729') |
| ('C10orf12', 'DO51964') |
| ('C11orf84', 'DO51954') |
| ('C16orf62', 'DO50316') |
| ('C17orf80', 'DO51964') |
| ('C18orf8', 'DO49442') |
| ('C19orf35', 'DO52510') |
| ('C1orf132', 'DO49442') |
| ('C2orf16', 'DO51954') |
| ('C6', 'DO51954') |
| ('CAB39L', 'DO51954') |
| ('CACNA1C', 'DO52737') |
| ('CAMSAP3', 'DO49442') |
| ('CAMTA1', 'DO218031') |
| ('CAMTA1', 'DO218243') |
| ('CARD11', 'DO27791') |
| ('CARD11', 'DO27825') |
| ('CARD11', 'DO52652') |
| ('CARD11', 'DO52689') |
| ('CARD11', 'DO52693') |
| ('CARD11', 'DO7084') |
| ('CASC2', 'DO49442') |
| ('CASC8', 'DO51954') |
| ('CASKIN1', 'DO49454') |
| ('CBFB', 'DO217962') |
| ('CBFB', 'DO218121') |
| ('CCNB1IP1', 'DO45049') |
| ('CCNB1IP1', 'DO51150') |
| ('CCND2', 'DO220862') |
| ('CCND2', 'DO220875') |
| ('CCND2', 'DO220886') |
| ('CCND2', 'DO220896') |
| ('CCND3', 'DO27765') |
| ('CCND3', 'DO27815') |
| ('CCND3', 'DO27829') |
| ('CCND3', 'DO52668') |
| ('CCND3', 'DO52683') |
| ('CCNL1', 'DO49442') |
| ('CCR10', 'DO49442') |
| ('CCR7', 'DO34728') |
| ('CCR7', 'DO35442') |
| ('CCT6P3', 'DO49454') |
| ('CCT6P3', 'DO51954') |
| ('CD209', 'DO218223') |
| ('CD209', 'DO220849') |
| ('CD209', 'DO35083') |
| ('CD209', 'DO48695') |
| ('CD209', 'DO50367') |
| ('CD209', 'DO51543') |
| ('CD209', 'DO51960') |
| ('CD209', 'DO52597') |
| ('CD28', 'DO220880') |
| ('CD28', 'DO220891') |
| ('CD2AP', 'DO51964') |
| ('CD79B', 'DO221129') |
| ('CD79B', 'DO52651') |
| ('CDC20B', 'DO49454') |
| ('CDC42SE2', 'DO51964') |
| ('CDH1', 'DO51955') |
| ('CDH10', 'DO220891') |
| ('CDH10', 'DO220906') |
| ('CDH10', 'DO220913') |
| ('CDH10', 'DO27779') |
| ('CDH10', 'DO51965') |
| ('CDH10', 'DO52512') |
| ('CDH10', 'DO52687') |
| ('CDH11', 'DO220851') |
| ('CDH11', 'DO220889') |
| ('CDH11', 'DO23517') |
| ('CDH11', 'DO35753') |
| ('CDH11', 'DO50346') |
| ('CDH11', 'DO50362') |
| ('CDH11', 'DO50447') |
| ('CDH11', 'DO50452') |
| ('CDH11', 'DO51959') |
| ('CDH11', 'DO52686') |
| ('CDH23', 'DO51964') |
| ('CDHR5', 'DO51954') |
| ('CDK12', 'DO51953') |
| ('CDKN2A', 'DO217950') |
| ('CDKN2A', 'DO218697') |
| ('CDKN2A', 'DO220857') |
| ('CDKN2A', 'DO220879') |
| ('CDKN2A', 'DO220885') |
| ('CDKN2A', 'DO220891') |
| ('CDKN2A', 'DO220900') |
| ('CDKN2A', 'DO220903') |
| ('CDKN2A', 'DO220911') |
| ('CDKN2A', 'DO221546') |
| ('CDKN2A', 'DO23525') |
| ('CDKN2A', 'DO32878') |
| ('CDKN2A', 'DO32984') |
| ('CDKN2A', 'DO33152') |
| ('CDKN2A', 'DO34312') |
| ('CDKN2A', 'DO34728') |
| ('CDKN2A', 'DO35083') |
| ('CDKN2A', 'DO35144') |
| ('CDKN2A', 'DO35184') |
| ('CDKN2A', 'DO35236') |
| ('CDKN2A', 'DO46783') |
| ('CDKN2A', 'DO49080') |
| ('CDKN2A', 'DO49135') |
| ('CDKN2A', 'DO49457') |
| ('CDKN2A', 'DO50330') |
| ('CDKN2A', 'DO50398') |
| ('CDKN2A', 'DO50408') |
| ('CDKN2A', 'DO50449') |
| ('CDKN2A', 'DO50451') |
| ('CDKN2A', 'DO50785') |
| ('CDKN2A', 'DO50844') |
| ('CDKN2A', 'DO51481') |
| ('CDKN2A', 'DO51484') |
| ('CDKN2A', 'DO51490') |
| ('CDKN2A', 'DO51502') |
| ('CDKN2A', 'DO51529') |
| ('CDKN2A', 'DO51534') |
| ('CDKN2A', 'DO52672') |
| ('CDKN2B-AS1', 'DO51964') |
| ('CDPF1', 'DO49442') |
| ('CEBPA', 'DO51962') |
| ('CEP170P1', 'DO34736') |
| ('CHD2', 'DO36223') |
| ('CHIC1', 'DO51964') |
| ('CHST11', 'DO218333') |
| ('CHST11', 'DO34849') |
| ('CIITA', 'DO218693') |
| ('CIITA', 'DO45299') |
| ('CLHC1', 'DO49442') |
| ('CLRN1-AS1', 'DO51954') |
| ('CMAHP', 'DO52737') |
| ('CNGA1', 'DO51964') |
| ('CNGB3', 'DO49442') |
| ('CNNM3', 'DO49442') |
| ('CNTNAP2', 'DO220849') |
| ('CNTNAP2', 'DO220877') |
| ('CNTNAP2', 'DO220889') |
| ('CNTNAP2', 'DO220891') |
| ('CNTNAP2', 'DO220899') |
| ('CNTNAP2', 'DO220900') |
| ('CNTNAP2', 'DO220902') |
| ('CNTNAP2', 'DO220910') |
| ('CNTNAP2', 'DO220912') |
| ('CNTNAP2', 'DO27827') |
| ('CNTNAP2', 'DO51955') |
| ('COL1A1', 'DO51965') |
| ('COL24A1', 'DO51964') |
| ('COL26A1', 'DO51954') |
| ('COL2A1', 'DO51955') |
| ('COL5A3', 'DO51964') |
| ('COL6A1', 'DO49454') |
| ('COL6A5', 'DO49454') |
| ('COPA', 'DO49442') |
| ('COPB1', 'DO52737') |
| ('CPNE4', 'DO51954') |
| ('CREB1', 'DO48577') |
| ('CREBBP', 'DO221124') |
| ('CREBBP', 'DO27785') |
| ('CREBBP', 'DO27787') |
| ('CREBBP', 'DO27817') |
| ('CREBBP', 'DO52662') |
| ('CREBBP', 'DO52681') |
| ('CREBBP', 'DO52690') |
| ('CREBBP', 'DO52693') |
| ('CREBBP', 'DO52694') |
| ('CREBBP', 'DO52695') |
| ('CRNKL1', 'DO220875') |
| ('CRNKL1', 'DO220891') |
| ('CRNKL1', 'DO51953') |
| ('CRYGEP', 'DO49454') |
| ('CRYM', 'DO52736') |
| ('CSDE1', 'DO49454') |
| ('CSF1R', 'DO220891') |
| ('CSF1R', 'DO220906') |
| ('CSMD1', 'DO49454') |
| ('CSMD3', 'DO218306') |
| ('CSMD3', 'DO218693') |
| ('CSMD3', 'DO220851') |
| ('CSMD3', 'DO220872') |
| ('CSMD3', 'DO220877') |
| ('CSMD3', 'DO220890') |
| ('CSMD3', 'DO220912') |
| ('CSMD3', 'DO27809') |
| ('CSMD3', 'DO27853') |
| ('CSMD3', 'DO35442') |
| ('CSMD3', 'DO45299') |
| ('CSMD3', 'DO50406') |
| ('CSMD3', 'DO51958') |
| ('CSMD3', 'DO51962') |
| ('CST7', 'DO49442') |
| ('CSTF3-AS1', 'DO51954') |
| ('CTA-85E5.10', 'DO45064') |
| ('CTB-118P15.2', 'DO49454') |
| ('CTB-186H2.2', 'DO51954') |
| ('CTB-49A3.2', 'DO51964') |
| ('CTB-57H20.1', 'DO52736') |
| ('CTB-57H20.1', 'DO52743') |
| ('CTBP1-AS2', 'DO49442') |
| ('CTBP1-AS2', 'DO51954') |
| ('CTBS', 'DO49454') |
| ('CTC-260E6.6', 'DO49442') |
| ('CTC-260E6.6', 'DO51954') |
| ('CTC-297N7.11', 'DO52510') |
| ('CTC-297N7.5', 'DO51954') |
| ('CTC-340A15.2', 'DO49454') |
| ('CTC-340A15.2', 'DO51954') |
| ('CTC-340A15.2', 'DO51964') |
| ('CTC-457E21.6', 'DO49454') |
| ('CTC-457E21.9', 'DO51964') |
| ('CTC-525D6.1', 'DO51964') |
| ('CTCF', 'DO52743') |
| ('CTD-2001E22.1', 'DO51954') |
| ('CTD-2014E2.5', 'DO52510') |
| ('CTD-2015H6.3', 'DO51954') |
| ('CTD-2021J15.1', 'DO52512') |
| ('CTD-2058B24.2', 'DO52737') |
| ('CTD-2176I21.2', 'DO51954') |
| ('CTD-2194D22.1', 'DO52736') |
| ('CTD-2203K17.1', 'DO49442') |
| ('CTD-2215L10.1', 'DO51954') |
| ('CTD-2269F5.1', 'DO51954') |
| ('CTD-2269F5.1', 'DO51964') |
| ('CTD-2288O8.1', 'DO49454') |
| ('CTD-2307P3.1', 'DO49442') |
| ('CTD-2307P3.1', 'DO49454') |
| ('CTD-2307P3.1', 'DO51954') |
| ('CTD-2307P3.1', 'DO52510') |
| ('CTD-2547L16.1', 'DO51964') |
| ('CTD-3006G17.2', 'DO49442') |
| ('CTD-3006G17.2', 'DO49454') |
| ('CTD-3006G17.2', 'DO51954') |
| ('CTD-3006G17.2', 'DO51964') |
| ('CTD-3006G17.2', 'DO52510') |
| ('CTD-3088G3.8', 'DO51964') |
| ('CTH', 'DO49442') |
| ('CTNNA2', 'DO220851') |
| ('CTNNA2', 'DO220891') |
| ('CTNNA2', 'DO220906') |
| ('CTNNA2', 'DO33264') |
| ('CTNNA2', 'DO50399') |
| ('CTNNA2', 'DO50444') |
| ('CTNNA2', 'DO51491') |
| ('CTNNA2', 'DO51958') |
| ('CTNNA2', 'DO51962') |
| ('CTNNA2', 'DO51965') |
| ('CTNNA3', 'DO51954') |
| ('CTNNB1', 'DO218673') |
| ('CTNNB1', 'DO218769') |
| ('CTNNB1', 'DO220873') |
| ('CTNNB1', 'DO23514') |
| ('CTNNB1', 'DO23518') |
| ('CTNNB1', 'DO23527') |
| ('CTNNB1', 'DO23529') |
| ('CTNNB1', 'DO23534') |
| ('CTNNB1', 'DO23542') |
| ('CTNNB1', 'DO23543') |
| ('CTNNB1', 'DO23548') |
| ('CTNNB1', 'DO23549') |
| ('CTNNB1', 'DO35555') |
| ('CTNNB1', 'DO35568') |
| ('CTNNB1', 'DO35582') |
| ('CTNNB1', 'DO44740') |
| ('CTNNB1', 'DO44806') |
| ('CTNNB1', 'DO44828') |
| ('CTNNB1', 'DO44832') |
| ('CTNNB1', 'DO45039') |
| ('CTNNB1', 'DO45041') |
| ('CTNNB1', 'DO45064') |
| ('CTNNB1', 'DO45067') |
| ('CTNNB1', 'DO45071') |
| ('CTNNB1', 'DO45073') |
| ('CTNNB1', 'DO45077') |
| ('CTNNB1', 'DO45083') |
| ('CTNNB1', 'DO45094') |
| ('CTNNB1', 'DO45097') |
| ('CTNNB1', 'DO45117') |
| ('CTNNB1', 'DO45127') |
| ('CTNNB1', 'DO45129') |
| ('CTNNB1', 'DO45133') |
| ('CTNNB1', 'DO45141') |
| ('CTNNB1', 'DO45161') |
| ('CTNNB1', 'DO45171') |
| ('CTNNB1', 'DO45177') |
| ('CTNNB1', 'DO45179') |
| ('CTNNB1', 'DO45199') |
| ('CTNNB1', 'DO45255') |
| ('CTNNB1', 'DO45267') |
| ('CTNNB1', 'DO45281') |
| ('CTNNB1', 'DO48557') |
| ('CTNNB1', 'DO48672') |
| ('CTNNB1', 'DO48679') |
| ('CTNNB1', 'DO48686') |
| ('CTNNB1', 'DO48689') |
| ('CTNNB1', 'DO48692') |
| ('CTNNB1', 'DO48703') |
| ('CTNNB1', 'DO48721') |
| ('CTNNB1', 'DO48742') |
| ('CTNNB1', 'DO48751') |
| ('CTNNB1', 'DO48757') |
| ('CTNNB1', 'DO48759') |
| ('CTNNB1', 'DO49466') |
| ('CTNNB1', 'DO50316') |
| ('CTNNB1', 'DO50343') |
| ('CTNNB1', 'DO50382') |
| ('CTNNB1', 'DO50780') |
| ('CTNNB1', 'DO50787') |
| ('CTNNB1', 'DO50793') |
| ('CTNNB1', 'DO50802') |
| ('CTNNB1', 'DO50804') |
| ('CTNNB1', 'DO50817') |
| ('CTNNB1', 'DO50818') |
| ('CTNNB1', 'DO50822') |
| ('CTNNB1', 'DO50834') |
| ('CTNNB1', 'DO50842') |
| ('CTNNB1', 'DO50851') |
| ('CTNNB1', 'DO50857') |
| ('CTNNB1', 'DO51057') |
| ('CTNNB1', 'DO51133') |
| ('CTNNB1', 'DO51958') |
| ('CTNNB1', 'DO51965') |
| ('CTNND2', 'DO220851') |
| ('CTNND2', 'DO220866') |
| ('CTNND2', 'DO51953') |
| ('CTNND2', 'DO51955') |
| ('CTSL3P', 'DO52743') |
| ('CTSS', 'DO52512') |
| ('CTTNBP2', 'DO51964') |
| ('CUL3', 'DO220861') |
| ('CUL3', 'DO220878') |
| ('CUL3', 'DO220906') |
| ('CUL3', 'DO220907') |
| ('CUL3', 'DO51953') |
| ('CXXC1P1', 'DO51954') |
| ('CXXC4', 'DO51954') |
| ('CYCS', 'DO49442') |
| ('CYLC2', 'DO52737') |
| ('CYP4B1', 'DO51964') |
| ('DARC', 'DO51954') |
| ('DAXX', 'DO46779') |
| ('DAXX', 'DO52129') |
| ('DCAF12L2', 'DO218478') |
| ('DCAF12L2', 'DO49436') |
| ('DCAF12L2', 'DO50793') |
| ('DCAF12L2', 'DO52561') |
| ('DCAF4L2', 'DO51964') |
| ('DCC', 'DO220878') |
| ('DCC', 'DO220906') |
| ('DCST2', 'DO49454') |
| ('DCTN4', 'DO49442') |
| ('DDR2', 'DO220860') |
| ('DDR2', 'DO220878') |
| ('DDR2', 'DO33152') |
| ('DDR2', 'DO45121') |
| ('DDX11-AS1', 'DO51964') |
| ('DDX3X', 'DO35083') |
| ('DDX3X', 'DO45299') |
| ('DDX3X', 'DO48891') |
| ('DDX3X', 'DO48939') |
| ('DDX46', 'DO220911') |
| ('DDX50', 'DO51964') |
| ('DDX6', 'DO51954') |
| ('DDX60', 'DO51964') |
| ('DEAF1', 'DO49442') |
| ('DGCR8', 'DO51959') |
| ('DGKE', 'DO49454') |
| ('DGKI', 'DO49442') |
| ('DIAPH2-AS1', 'DO220878') |
| ('DIO2-AS1', 'DO51964') |
| ('DIRC3', 'DO51964') |
| ('DISC1FP1', 'DO49442') |
| ('DISC1FP1', 'DO49454') |
| ('DISC1FP1', 'DO51954') |
| ('DISC1FP1', 'DO51964') |
| ('DLG2', 'DO51954') |
| ('DNAJB7', 'DO52736') |
| ('DNAJB9', 'DO49454') |
| ('DNER', 'DO49442') |
| ('DNMT3A', 'DO52744') |
| ('DNMT3A', 'DO52756') |
| ('DOCK5', 'DO51964') |
| ('DSTN', 'DO49442') |
| ('DTX4', 'DO46834') |
| ('DTX4', 'DO49442') |
| ('DUOX2', 'DO51964') |
| ('DUT', 'DO49454') |
| ('DZIP1', 'DO52737') |
| ('EBF1', 'DO27859') |
| ('EBF1', 'DO52675') |
| ('EED', 'DO35704') |
| ('EED', 'DO52724') |
| ('EFCAB1', 'DO51964') |
| ('EGF', 'DO49442') |
| ('EGFEM1P', 'DO51964') |
| ('EHHADH-AS1', 'DO51964') |
| ('EIF1AX', 'DO51965') |
| ('EIF4A2', 'DO49454') |
| ('EIF4E', 'DO51964') |
| ('ELF3', 'DO51965') |
| ('ELK4', 'DO220866') |
| ('ELK4', 'DO220878') |
| ('ELK4', 'DO220902') |
| ('ELK4', 'DO52671') |
| ('ELL', 'DO52513') |
| ('ELOVL2', 'DO51954') |
| ('EMCN-IT3', 'DO49442') |
| ('EMCN-IT3', 'DO49454') |
| ('EMILIN1', 'DO51964') |
| ('EML4', 'DO51960') |
| ('EMR4P', 'DO49454') |
| ('ENTPD3-AS1', 'DO51954') |
| ('EOGT', 'DO49442') |
| ('EPAS1', 'DO52733') |
| ('EPHA1-AS1', 'DO51954') |
| ('EPHA1-AS1', 'DO51964') |
| ('EPHA7', 'DO220893') |
| ('EPHA7', 'DO220903') |
| ('EPHA7', 'DO51954') |
| ('EPSTI1', 'DO51964') |
| ('ERBB2', 'DO50318') |
| ('ERBB2', 'DO50331') |
| ('ERBB2', 'DO50332') |
| ('ERBB3', 'DO218347') |
| ('ERBB3', 'DO50348') |
| ('ERBB4', 'DO218547') |
| ('ERBB4', 'DO220851') |
| ('ERBB4', 'DO220855') |
| ('ERBB4', 'DO220872') |
| ('ERBB4', 'DO220878') |
| ('ERBB4', 'DO220885') |
| ('ERBB4', 'DO220887') |
| ('ERBB4', 'DO220890') |
| ('ERBB4', 'DO220899') |
| ('ERBB4', 'DO220900') |
| ('ERBB4', 'DO220907') |
| ('ERBB4', 'DO220912') |
| ('ERBB4', 'DO45207') |
| ('ERBB4', 'DO49537') |
| ('ERBB4', 'DO50450') |
| ('ERBB4', 'DO51962') |
| ('ERBB4', 'DO51992') |
| ('ERCC4', 'DO220890') |
| ('ERCC4', 'DO220895') |
| ('ERICH1-AS1', 'DO51964') |
| ('ERMP1', 'DO51954') |
| ('ESPNP', 'DO51964') |
| ('ESRRAP2', 'DO51964') |
| ('EXT1', 'DO218693') |
| ('EXT1', 'DO35442') |
| ('EXT1', 'DO45299') |
| ('EXT2', 'DO52512') |
| ('EZH2', 'DO220875') |
| ('EZH2', 'DO27791') |
| ('EZH2', 'DO27803') |
| ('EZH2', 'DO27837') |
| ('EZH2', 'DO27849') |
| ('EZH2', 'DO27857') |
| ('EZH2', 'DO52652') |
| ('EZH2', 'DO52653') |
| ('EZH2', 'DO52655') |
| ('EZH2', 'DO52671') |
| ('EZH2', 'DO52685') |
| ('EZH2', 'DO52688') |
| ('EZH2', 'DO52751') |
| ('F11-AS1', 'DO51964') |
| ('F11-AS1', 'DO52510') |
| ('F11-AS1', 'DO52743') |
| ('FAIM2', 'DO51964') |
| ('FAM120AOS', 'DO51954') |
| ('FAM135B', 'DO10858') |
| ('FAM135B', 'DO218031') |
| ('FAM135B', 'DO218243') |
| ('FAM135B', 'DO220857') |
| ('FAM135B', 'DO220886') |
| ('FAM135B', 'DO27805') |
| ('FAM135B', 'DO46398') |
| ('FAM135B', 'DO51955') |
| ('FAM135B', 'DO52664') |
| ('FAM153B', 'DO51954') |
| ('FAM154A', 'DO52512') |
| ('FAM177A1', 'DO51964') |
| ('FAM179A', 'DO49454') |
| ('FAM181A-AS1', 'DO51954') |
| ('FAM184B', 'DO51954') |
| ('FAM210B', 'DO49442') |
| ('FAM230B', 'DO51954') |
| ('FAM27E3', 'DO49442') |
| ('FAM3B', 'DO49442') |
| ('FAM47C', 'DO220851') |
| ('FAM47C', 'DO49076') |
| ('FAM84B', 'DO49454') |
| ('FAM86JP', 'DO49442') |
| ('FARSB', 'DO52510') |
| ('FAT1', 'DO51956') |
| ('FAT1', 'DO52740') |
| ('FAT3', 'DO220877') |
| ('FAT3', 'DO220889') |
| ('FAT3', 'DO46424') |
| ('FAT3', 'DO51049') |
| ('FAT3', 'DO52745') |
| ('FAT4', 'DO34504') |
| ('FAT4', 'DO34785') |
| ('FAT4', 'DO46330') |
| ('FAT4', 'DO49420') |
| ('FAT4', 'DO50430') |
| ('FAT4', 'DO51070') |
| ('FAT4', 'DO51962') |
| ('FBN1', 'DO51964') |
| ('FBXL5', 'DO51964') |
| ('FBXO11', 'DO27767') |
| ('FBXO11', 'DO27821') |
| ('FBXO11', 'DO52691') |
| ('FBXO28', 'DO51964') |
| ('FBXW7', 'DO218443') |
| ('FBXW7', 'DO48899') |
| ('FCRL3', 'DO52510') |
| ('FETUB', 'DO51954') |
| ('FGFR1', 'DO35937') |
| ('FGFR1', 'DO35982') |
| ('FGFR1', 'DO36009') |
| ('FGFR1', 'DO36030') |
| ('FGFR1', 'DO36171') |
| ('FLG-AS1', 'DO49454') |
| ('FLG-AS1', 'DO51954') |
| ('FLG-AS1', 'DO51964') |
| ('FLI1', 'DO218550') |
| ('FLI1', 'DO51465') |
| ('FLT4', 'DO45079') |
| ('FLT4', 'DO49168') |
| ('FNDC1', 'DO51954') |
| ('FNDC3B', 'DO52510') |
| ('FNIP2', 'DO49454') |
| ('FOLH1B', 'DO49454') |
| ('FOLH1B', 'DO51954') |
| ('FOXA1', 'DO51953') |
| ('FOXL2', 'DO51141') |
| ('FOXL2', 'DO52691') |
| ('FOXO1', 'DO50452') |
| ('FOXO1', 'DO52671') |
| ('FOXO1', 'DO52674') |
| ('FOXO1', 'DO52679') |
| ('FREM2', 'DO49454') |
| ('FREM2', 'DO51954') |
| ('FSIP2', 'DO52737') |
| ('FZD1', 'DO52512') |
| ('FZD10-AS1', 'DO51964') |
| ('FZD3', 'DO49442') |
| ('GABRB1', 'DO49454') |
| ('GATA3', 'DO1007') |
| ('GATA3', 'DO1076') |
| ('GATA3', 'DO217934') |
| ('GATA3', 'DO217939') |
| ('GATA3', 'DO217953') |
| ('GATA3', 'DO217962') |
| ('GATA3', 'DO218173') |
| ('GATA3', 'DO218408') |
| ('GATA3', 'DO218506') |
| ('GATA3', 'DO218553') |
| ('GATA3', 'DO49454') |
| ('GATAD2A', 'DO51954') |
| ('GBA3', 'DO49442') |
| ('GBA3', 'DO49454') |
| ('GBA3', 'DO51954') |
| ('GCM1', 'DO49442') |
| ('GDF7', 'DO49454') |
| ('GFRAL', 'DO49454') |
| ('GGTA1P', 'DO49442') |
| ('GJD2', 'DO51964') |
| ('GLTSCR2', 'DO49442') |
| ('GLUD1P2', 'DO51964') |
| ('GNA11', 'DO51953') |
| ('GNAS', 'DO218621') |
| ('GNAS', 'DO33016') |
| ('GNAS', 'DO33264') |
| ('GNAS', 'DO33488') |
| ('GNAS', 'DO35442') |
| ('GNAS', 'DO45299') |
| ('GNAS', 'DO46378') |
| ('GNAS', 'DO48541') |
| ('GNAS', 'DO49087') |
| ('GNAS', 'DO49421') |
| ('GNAS', 'DO50316') |
| ('GNAS', 'DO50325') |
| ('GNAS', 'DO50387') |
| ('GNAS', 'DO50410') |
| ('GNAS', 'DO51500') |
| ('GNAS', 'DO51515') |
| ('GNAS', 'DO51528') |
| ('GOPC', 'DO51955') |
| ('GPD2', 'DO51964') |
| ('GPR133', 'DO51964') |
| ('GPR156', 'DO51954') |
| ('GPR158-AS1', 'DO49442') |
| ('GPR4', 'DO51954') |
| ('GPT', 'DO51964') |
| ('GRID1', 'DO51964') |
| ('GRIN2A', 'DO10821') |
| ('GRIN2A', 'DO220877') |
| ('GRIN2A', 'DO220879') |
| ('GRIN2A', 'DO220880') |
| ('GRIN2A', 'DO220885') |
| ('GRIN2A', 'DO220886') |
| ('GRIN2A', 'DO220889') |
| ('GRIN2A', 'DO220903') |
| ('GRIN2A', 'DO220906') |
| ('GRIN2A', 'DO51148') |
| ('GRIPAP1', 'DO51964') |
| ('GRM3', 'DO220851') |
| ('GRM3', 'DO220877') |
| ('GRM3', 'DO220886') |
| ('GRM3', 'DO220900') |
| ('GRM6', 'DO49454') |
| ('GRPEL1', 'DO49454') |
| ('GS1-256O22.5', 'DO49442') |
| ('GS1-256O22.5', 'DO51954') |
| ('GS1-256O22.5', 'DO51964') |
| ('GS1-256O22.5', 'DO52736') |
| ('GTF2A1', 'DO49442') |
| ('GTF2H5', 'DO51954') |
| ('GTPBP10', 'DO49442') |
| ('GTPBP10', 'DO51954') |
| ('GUCY1B2', 'DO49442') |
| ('GUCY1B2', 'DO51954') |
| ('GUSBP1', 'DO51964') |
| ('H3F3A', 'DO47016') |
| ('H3F3A', 'DO49442') |
| ('H3F3A', 'DO52543') |
| ('H3F3A', 'DO52575') |
| ('H3F3A', 'DO52633') |
| ('H3F3B', 'DO52623') |
| ('H3F3B', 'DO52625') |
| ('H3F3B', 'DO52629') |
| ('H3F3B', 'DO52631') |
| ('HCG18', 'DO49442') |
| ('HCG18', 'DO51964') |
| ('HCN4', 'DO51964') |
| ('HEATR6', 'DO51954') |
| ('HEBP2', 'DO51964') |
| ('HEG1', 'DO51954') |
| ('HELQ', 'DO52736') |
| ('HELZ2', 'DO49442') |
| ('HHIPL1', 'DO51954') |
| ('HIST1H1A', 'DO51964') |
| ('HLA-A', 'DO35116') |
| ('HLA-A', 'DO50842') |
| ('HLA-A', 'DO52674') |
| ('HLA-A', 'DO52675') |
| ('HLA-AS1', 'DO49454') |
| ('HLTF-AS1', 'DO52743') |
| ('HMCN1', 'DO50844') |
| ('HMGCS1', 'DO51964') |
| ('HMGN2P46', 'DO218031') |
| ('HMGN2P46', 'DO218243') |
| ('HMGN2P46', 'DO220878') |
| ('HMGN2P46', 'DO35083') |
| ('HMGN2P46', 'DO51549') |
| ('HMGN2P46', 'DO52664') |
| ('HMX3', 'DO51954') |
| ('HNF4A', 'DO45096') |
| ('HNRNPA1P48', 'DO51964') |
| ('HNRNPA2B1', 'DO218693') |
| ('HNRNPA2B1', 'DO45299') |
| ('HOOK3', 'DO218693') |
| ('HOOK3', 'DO46591') |
| ('HOXC11', 'DO49442') |
| ('HOXC13', 'DO51954') |
| ('HPN-AS1', 'DO49454') |
| ('HS3ST1', 'DO49442') |
| ('HSP90AA1', 'DO51965') |
| ('HTR3B', 'DO52743') |
| ('HTR5BP', 'DO49454') |
| ('HTR5BP', 'DO51954') |
| ('IBTK', 'DO220906') |
| ('ID3', 'DO27763') |
| ('ID3', 'DO27767') |
| ('ID3', 'DO27793') |
| ('ID3', 'DO27829') |
| ('IDH1', 'DO220893') |
| ('IDH1', 'DO27851') |
| ('IDH1', 'DO45305') |
| ('IDH1', 'DO48911') |
| ('IDH1', 'DO48945') |
| ('IDH1', 'DO51063') |
| ('IDH2', 'DO52740') |
| ('IDH2', 'DO52747') |
| ('IFNG-AS1', 'DO49442') |
| ('IFNG-AS1', 'DO52510') |
| ('IGFBP7-AS1', 'DO51964') |
| ('IGHD3-10', 'DO51964') |
| ('IGHD3OR15-3B', 'DO49442') |
| ('IGHJ6', 'DO27771') |
| ('IGHM', 'DO27853') |
| ('IGHM', 'DO52743') |
| ('IGHV1-24', 'DO51964') |
| ('IGHV2-5', 'DO49454') |
| ('IGHV3OR16-13', 'DO49442') |
| ('IGHV3OR16-13', 'DO51954') |
| ('IGKJ3', 'DO52692') |
| ('IGKJ5', 'DO52650') |
| ('IGKV1-27', 'DO51964') |
| ('IGKV1D-37', 'DO51954') |
| ('IGLV1-51', 'DO49442') |
| ('IGLV7-46', 'DO51964') |
| ('IKZF1', 'DO218280') |
| ('IKZF1', 'DO218491') |
| ('IKZF1', 'DO46400') |
| ('IKZF1', 'DO48940') |
| ('IKZF1', 'DO52510') |
| ('IL12A-AS1', 'DO49442') |
| ('IL12A-AS1', 'DO51954') |
| ('IL12A-AS1', 'DO51964') |
| ('IL1RAP', 'DO49454') |
| ('IL21R-AS1', 'DO49454') |
| ('IL6ST', 'DO218693') |
| ('IL6ST', 'DO48682') |
| ('IL6ST', 'DO51953') |
| ('ILDR2', 'DO49454') |
| ('INHBA', 'DO52510') |
| ('INHBA-AS1', 'DO49442') |
| ('INHBA-AS1', 'DO51954') |
| ('INHBA-AS1', 'DO51964') |
| ('INTS4', 'DO51954') |
| ('INTS4L1', 'DO51954') |
| ('ISM2', 'DO52737') |
| ('ITGAV', 'DO49442') |
| ('ITGB2', 'DO51964') |
| ('ITK', 'DO52760') |
| ('JAG2', 'DO49454') |
| ('JAK2', 'DO52762') |
| ('JAK3', 'DO217844') |
| ('JAK3', 'DO46330') |
| ('JAZF1', 'DO220899') |
| ('JAZF1', 'DO220906') |
| ('KAT6A', 'DO49454') |
| ('KAT6A', 'DO51964') |
| ('KB-1562D12.1', 'DO51964') |
| ('KCNB2', 'DO51964') |
| ('KCNC1', 'DO49454') |
| ('KCND1', 'DO52736') |
| ('KCND2', 'DO49442') |
| ('KCNJ5', 'DO52510') |
| ('KCNJ5', 'DO52512') |
| ('KCNMB3P1', 'DO51954') |
| ('KCNRG', 'DO49442') |
| ('KCNV1', 'DO51964') |
| ('KDM5A', 'DO218693') |
| ('KDM5A', 'DO45299') |
| ('KDM5A', 'DO51050') |
| ('KDM5A', 'DO51114') |
| ('KDM5A', 'DO51965') |
| ('KDM6A', 'DO51953') |
| ('KDR', 'DO52733') |
| ('KEAP1', 'DO220886') |
| ('KEAP1', 'DO220906') |
| ('KHDRBS3', 'DO51964') |
| ('KIAA0513', 'DO52510') |
| ('KIAA1257', 'DO51954') |
| ('KIAA1549', 'DO220877') |
| ('KIAA1549', 'DO27779') |
| ('KIAA1549', 'DO51960') |
| ('KIAA1549', 'DO51965') |
| ('KIAA1683', 'DO51954') |
| ('KIAA2022', 'DO51964') |
| ('KIF1A', 'DO52512') |
| ('KIF25', 'DO51954') |
| ('KIF9-AS1', 'DO52510') |
| ('KIRREL3-AS3', 'DO51954') |
| ('KIT', 'DO36221') |
| ('KIT', 'DO49419') |
| ('KIT', 'DO50342') |
| ('KLF11', 'DO51964') |
| ('KLF6', 'DO52738') |
| ('KLHL3', 'DO51954') |
| ('KLK2', 'DO220885') |
| ('KLK2', 'DO220907') |
| ('KLK2', 'DO36221') |
| ('KLK2', 'DO45123') |
| ('KLK2', 'DO51548') |
| ('KMT2A', 'DO48895') |
| ('KMT2A', 'DO51062') |
| ('KMT2C', 'DO35098') |
| ('KMT2C', 'DO51111') |
| ('KMT2C', 'DO51959') |
| ('KMT2D', 'DO220891') |
| ('KMT2D', 'DO220907') |
| ('KMT2D', 'DO27783') |
| ('KMT2D', 'DO27849') |
| ('KMT2D', 'DO27859') |
| ('KMT2D', 'DO45149') |
| ('KMT2D', 'DO48945') |
| ('KNSTRN', 'DO220895') |
| ('KNSTRN', 'DO220912') |
| ('KNTC1', 'DO51964') |
| ('KRAS', 'DO217850') |
| ('KRAS', 'DO218139') |
| ('KRAS', 'DO218180') |
| ('KRAS', 'DO218693') |
| ('KRAS', 'DO221539') |
| ('KRAS', 'DO221540') |
| ('KRAS', 'DO221541') |
| ('KRAS', 'DO221542') |
| ('KRAS', 'DO221543') |
| ('KRAS', 'DO221544') |
| ('KRAS', 'DO221545') |
| ('KRAS', 'DO221546') |
| ('KRAS', 'DO221547') |
| ('KRAS', 'DO32831') |
| ('KRAS', 'DO32833') |
| ('KRAS', 'DO32837') |
| ('KRAS', 'DO32860') |
| ('KRAS', 'DO32863') |
| ('KRAS', 'DO32875') |
| ('KRAS', 'DO32878') |
| ('KRAS', 'DO32893') |
| ('KRAS', 'DO32900') |
| ('KRAS', 'DO32916') |
| ('KRAS', 'DO32972') |
| ('KRAS', 'DO32984') |
| ('KRAS', 'DO33000') |
| ('KRAS', 'DO33008') |
| ('KRAS', 'DO33028') |
| ('KRAS', 'DO33042') |
| ('KRAS', 'DO33128') |
| ('KRAS', 'DO33152') |
| ('KRAS', 'DO33160') |
| ('KRAS', 'DO33168') |
| ('KRAS', 'DO33184') |
| ('KRAS', 'DO33200') |
| ('KRAS', 'DO33208') |
| ('KRAS', 'DO33248') |
| ('KRAS', 'DO33264') |
| ('KRAS', 'DO33288') |
| ('KRAS', 'DO33344') |
| ('KRAS', 'DO33368') |
| ('KRAS', 'DO33376') |
| ('KRAS', 'DO33392') |
| ('KRAS', 'DO33400') |
| ('KRAS', 'DO33408') |
| ('KRAS', 'DO33472') |
| ('KRAS', 'DO33480') |
| ('KRAS', 'DO33512') |
| ('KRAS', 'DO33544') |
| ('KRAS', 'DO33552') |
| ('KRAS', 'DO33600') |
| ('KRAS', 'DO33960') |
| ('KRAS', 'DO33984') |
| ('KRAS', 'DO34264') |
| ('KRAS', 'DO34288') |
| ('KRAS', 'DO34312') |
| ('KRAS', 'DO34336') |
| ('KRAS', 'DO34448') |
| ('KRAS', 'DO34600') |
| ('KRAS', 'DO34608') |
| ('KRAS', 'DO34616') |
| ('KRAS', 'DO34640') |
| ('KRAS', 'DO34656') |
| ('KRAS', 'DO34680') |
| ('KRAS', 'DO34696') |
| ('KRAS', 'DO34720') |
| ('KRAS', 'DO34728') |
| ('KRAS', 'DO34736') |
| ('KRAS', 'DO34785') |
| ('KRAS', 'DO34793') |
| ('KRAS', 'DO34801') |
| ('KRAS', 'DO34809') |
| ('KRAS', 'DO34817') |
| ('KRAS', 'DO34849') |
| ('KRAS', 'DO34905') |
| ('KRAS', 'DO35082') |
| ('KRAS', 'DO35083') |
| ('KRAS', 'DO35085') |
| ('KRAS', 'DO35098') |
| ('KRAS', 'DO35116') |
| ('KRAS', 'DO35118') |
| ('KRAS', 'DO35126') |
| ('KRAS', 'DO35128') |
| ('KRAS', 'DO35132') |
| ('KRAS', 'DO35136') |
| ('KRAS', 'DO35144') |
| ('KRAS', 'DO35148') |
| ('KRAS', 'DO35152') |
| ('KRAS', 'DO35184') |
| ('KRAS', 'DO35186') |
| ('KRAS', 'DO35198') |
| ('KRAS', 'DO35200') |
| ('KRAS', 'DO35216') |
| ('KRAS', 'DO35222') |
| ('KRAS', 'DO35228') |
| ('KRAS', 'DO35230') |
| ('KRAS', 'DO35236') |
| ('KRAS', 'DO35258') |
| ('KRAS', 'DO35290') |
| ('KRAS', 'DO35305') |
| ('KRAS', 'DO35350') |
| ('KRAS', 'DO35360') |
| ('KRAS', 'DO35376') |
| ('KRAS', 'DO35406') |
| ('KRAS', 'DO35424') |
| ('KRAS', 'DO35454') |
| ('KRAS', 'DO35496') |
| ('KRAS', 'DO45251') |
| ('KRAS', 'DO45297') |
| ('KRAS', 'DO46586') |
| ('KRAS', 'DO48692') |
| ('KRAS', 'DO49074') |
| ('KRAS', 'DO49078') |
| ('KRAS', 'DO49079') |
| ('KRAS', 'DO49080') |
| ('KRAS', 'DO49113') |
| ('KRAS', 'DO49127') |
| ('KRAS', 'DO49129') |
| ('KRAS', 'DO49135') |
| ('KRAS', 'DO49137') |
| ('KRAS', 'DO49138') |
| ('KRAS', 'DO49166') |
| ('KRAS', 'DO49168') |
| ('KRAS', 'DO49172') |
| ('KRAS', 'DO49178') |
| ('KRAS', 'DO49181') |
| ('KRAS', 'DO49183') |
| ('KRAS', 'DO49184') |
| ('KRAS', 'DO49193') |
| ('KRAS', 'DO49204') |
| ('KRAS', 'DO49419') |
| ('KRAS', 'DO49420') |
| ('KRAS', 'DO49422') |
| ('KRAS', 'DO49424') |
| ('KRAS', 'DO49427') |
| ('KRAS', 'DO49433') |
| ('KRAS', 'DO49436') |
| ('KRAS', 'DO49439') |
| ('KRAS', 'DO49445') |
| ('KRAS', 'DO49448') |
| ('KRAS', 'DO49451') |
| ('KRAS', 'DO49454') |
| ('KRAS', 'DO49457') |
| ('KRAS', 'DO49460') |
| ('KRAS', 'DO49463') |
| ('KRAS', 'DO49466') |
| ('KRAS', 'DO49481') |
| ('KRAS', 'DO49484') |
| ('KRAS', 'DO50307') |
| ('KRAS', 'DO50396') |
| ('KRAS', 'DO50451') |
| ('KRAS', 'DO50453') |
| ('KRAS', 'DO51465') |
| ('KRAS', 'DO51466') |
| ('KRAS', 'DO51467') |
| ('KRAS', 'DO51468') |
| ('KRAS', 'DO51469') |
| ('KRAS', 'DO51470') |
| ('KRAS', 'DO51472') |
| ('KRAS', 'DO51473') |
| ('KRAS', 'DO51474') |
| ('KRAS', 'DO51475') |
| ('KRAS', 'DO51477') |
| ('KRAS', 'DO51478') |
| ('KRAS', 'DO51480') |
| ('KRAS', 'DO51481') |
| ('KRAS', 'DO51483') |
| ('KRAS', 'DO51484') |
| ('KRAS', 'DO51485') |
| ('KRAS', 'DO51486') |
| ('KRAS', 'DO51488') |
| ('KRAS', 'DO51490') |
| ('KRAS', 'DO51492') |
| ('KRAS', 'DO51493') |
| ('KRAS', 'DO51494') |
| ('KRAS', 'DO51495') |
| ('KRAS', 'DO51496') |
| ('KRAS', 'DO51497') |
| ('KRAS', 'DO51498') |
| ('KRAS', 'DO51499') |
| ('KRAS', 'DO51500') |
| ('KRAS', 'DO51501') |
| ('KRAS', 'DO51504') |
| ('KRAS', 'DO51505') |
| ('KRAS', 'DO51506') |
| ('KRAS', 'DO51507') |
| ('KRAS', 'DO51509') |
| ('KRAS', 'DO51511') |
| ('KRAS', 'DO51512') |
| ('KRAS', 'DO51513') |
| ('KRAS', 'DO51514') |
| ('KRAS', 'DO51515') |
| ('KRAS', 'DO51518') |
| ('KRAS', 'DO51519') |
| ('KRAS', 'DO51520') |
| ('KRAS', 'DO51522') |
| ('KRAS', 'DO51523') |
| ('KRAS', 'DO51524') |
| ('KRAS', 'DO51527') |
| ('KRAS', 'DO51528') |
| ('KRAS', 'DO51529') |
| ('KRAS', 'DO51530') |
| ('KRAS', 'DO51531') |
| ('KRAS', 'DO51532') |
| ('KRAS', 'DO51533') |
| ('KRAS', 'DO51534') |
| ('KRAS', 'DO51535') |
| ('KRAS', 'DO51536') |
| ('KRAS', 'DO51537') |
| ('KRAS', 'DO51538') |
| ('KRAS', 'DO51540') |
| ('KRAS', 'DO51542') |
| ('KRAS', 'DO51543') |
| ('KRAS', 'DO51544') |
| ('KRAS', 'DO51546') |
| ('KRAS', 'DO51548') |
| ('KRAS', 'DO51549') |
| ('KTN1', 'DO51962') |
| ('LAMA2', 'DO49442') |
| ('LARP4B', 'DO218693') |
| ('LARP4B', 'DO35083') |
| ('LARP4B', 'DO45299') |
| ('LCP1', 'DO51964') |
| ('LEF1', 'DO35442') |
| ('LEF1', 'DO45299') |
| ('LEF1', 'DO51959') |
| ('LEF1-AS1', 'DO51964') |
| ('LEPROTL1', 'DO1076') |
| ('LEPROTL1', 'DO217908') |
| ('LIFR', 'DO220852') |
| ('LIFR', 'DO220911') |
| ('LIFR-AS1', 'DO49442') |
| ('LINC00189', 'DO49454') |
| ('LINC00189', 'DO51964') |
| ('LINC00395', 'DO51954') |
| ('LINC00395', 'DO51964') |
| ('LINC00467', 'DO51954') |
| ('LINC00475', 'DO49454') |
| ('LINC00535', 'DO49442') |
| ('LINC00535', 'DO51954') |
| ('LINC00535', 'DO51964') |
| ('LINC00535', 'DO52737') |
| ('LINC00634', 'DO49454') |
| ('LINC00649', 'DO45096') |
| ('LINC00963', 'DO51954') |
| ('LINC01088', 'DO51954') |
| ('LL09NC01-254D11.1', 'DO52510') |
| ('LLNLF-65H9.1', 'DO49454') |
| ('LMCD1-AS1', 'DO51954') |
| ('LMCD1-AS1', 'DO52736') |
| ('LMNA', 'DO51964') |
| ('LMTK2', 'DO51964') |
| ('LPP', 'DO45299') |
| ('LPP', 'DO48682') |
| ('LRP1B', 'DO220902') |
| ('LRP1B', 'DO220906') |
| ('LRP1B', 'DO46330') |
| ('LRP1B', 'DO48719') |
| ('LRP1B', 'DO50454') |
| ('LRP1B', 'DO50819') |
| ('LRP1B', 'DO52674') |
| ('LRP1B', 'DO52690') |
| ('LRP3', 'DO51964') |
| ('LRRC37A16P', 'DO49454') |
| ('LRRC49', 'DO51964') |
| ('LRRC4C', 'DO46488') |
| ('LRRIQ1', 'DO49442') |
| ('LYL1', 'DO35083') |
| ('LYL1', 'DO50453') |
| ('MACC1', 'DO220851') |
| ('MACC1', 'DO220879') |
| ('MACC1', 'DO220886') |
| ('MACC1', 'DO220911') |
| ('MACC1', 'DO51962') |
| ('MACC1', 'DO52739') |
| ('MAF', 'DO45075') |
| ('MAF', 'DO50340') |
| ('MAF', 'DO51962') |
| ('MAGI3', 'DO51964') |
| ('MALT1', 'DO220851') |
| ('MALT1', 'DO220886') |
| ('MAMDC2', 'DO51964') |
| ('MAP3K13', 'DO52512') |
| ('MAX', 'DO48900') |
| ('MAX', 'DO51500') |
| ('MB21D2', 'DO36221') |
| ('MCC', 'DO51964') |
| ('MCHR2-AS1', 'DO51964') |
| ('MCHR2-AS1', 'DO52736') |
| ('MECOM', 'DO220877') |
| ('MECOM', 'DO220878') |
| ('MECOM', 'DO220890') |
| ('MECOM', 'DO220894') |
| ('MECOM', 'DO220907') |
| ('MECOM', 'DO50447') |
| ('MECOM', 'DO52752') |
| ('MED12', 'DO34312') |
| ('MED12', 'DO36223') |
| ('MED12', 'DO51061') |
| ('MED12', 'DO51064') |
| ('MED12', 'DO51084') |
| ('MED12', 'DO52509') |
| ('MED15P9', 'DO49442') |
| ('MEF2C-AS1', 'DO49442') |
| ('MEF2C-AS1', 'DO49454') |
| ('MEF2C-AS1', 'DO50818') |
| ('MEF2C-AS1', 'DO51954') |
| ('MEF2C-AS1', 'DO51964') |
| ('MEF2C-AS1', 'DO52510') |
| ('MEGF10', 'DO49442') |
| ('MEN1', 'DO48578') |
| ('MEN1', 'DO51178') |
| ('MEPCE', 'DO220874') |
| ('MGAM', 'DO49442') |
| ('MIR1268A', 'DO49454') |
| ('MIR184', 'DO51964') |
| ('MIR31HG', 'DO51954') |
| ('MIR3622A', 'DO51964') |
| ('MIR378D1', 'DO52736') |
| ('MIR4431', 'DO49454') |
| ('MIR4454', 'DO49454') |
| ('MIR4472-1', 'DO49442') |
| ('MIR496', 'DO51964') |
| ('MIR518D', 'DO52510') |
| ('MIR526B', 'DO52510') |
| ('MIR548H4', 'DO51954') |
| ('MIR596', 'DO51954') |
| ('MITF', 'DO52732') |
| ('MKLN1-AS1', 'DO49442') |
| ('MKLN1-AS1', 'DO51954') |
| ('MLH1', 'DO220875') |
| ('MLH1', 'DO220877') |
| ('MLK7-AS1', 'DO51964') |
| ('MLLT3', 'DO220820') |
| ('MLLT3', 'DO35083') |
| ('MLLT3', 'DO51958') |
| ('MLLT3', 'DO51962') |
| ('MMP8', 'DO51954') |
| ('MRPL45P2', 'DO51964') |
| ('MSH6', 'DO220889') |
| ('MSH6', 'DO220899') |
| ('MSLN', 'DO51954') |
| ('MTHFD2P1', 'DO51954') |
| ('MTMR9LP', 'DO49454') |
| ('MTOR', 'DO221123') |
| ('MTOR', 'DO47048') |
| ('MTOR', 'DO47174') |
| ('MTRNR2L5', 'DO51954') |
| ('MTRNR2L7', 'DO49454') |
| ('MTSS1L', 'DO51954') |
| ('MUC16', 'DO220853') |
| ('MUC16', 'DO220857') |
| ('MUC16', 'DO220862') |
| ('MUC16', 'DO220875') |
| ('MUC16', 'DO220877') |
| ('MUC16', 'DO220881') |
| ('MUC16', 'DO220882') |
| ('MUC16', 'DO220886') |
| ('MUC16', 'DO220889') |
| ('MUC16', 'DO220891') |
| ('MUC16', 'DO220892') |
| ('MUC16', 'DO220893') |
| ('MUC16', 'DO220899') |
| ('MUC16', 'DO220902') |
| ('MUC16', 'DO220903') |
| ('MUC16', 'DO220906') |
| ('MUC16', 'DO220907') |
| ('MUC16', 'DO220911') |
| ('MUC16', 'DO220912') |
| ('MUC16', 'DO220913') |
| ('MUC16', 'DO27819') |
| ('MUC16', 'DO33368') |
| ('MUC16', 'DO35083') |
| ('MUC16', 'DO36223') |
| ('MUC16', 'DO46897') |
| ('MUC16', 'DO48760') |
| ('MUC16', 'DO50384') |
| ('MUC16', 'DO50450') |
| ('MUC16', 'DO52661') |
| ('MUC16', 'DO52756') |
| ('MUC4', 'DO51953') |
| ('MUC4', 'DO51954') |
| ('MUC4', 'DO52758') |
| ('MYC', 'DO27763') |
| ('MYC', 'DO27767') |
| ('MYC', 'DO27769') |
| ('MYC', 'DO27775') |
| ('MYC', 'DO27793') |
| ('MYC', 'DO27819') |
| ('MYC', 'DO27825') |
| ('MYC', 'DO52659') |
| ('MYC', 'DO52661') |
| ('MYC', 'DO52691') |
| ('MYC', 'DO52692') |
| ('MYD88', 'DO218031') |
| ('MYD88', 'DO218243') |
| ('MYD88', 'DO221129') |
| ('MYD88', 'DO27857') |
| ('MYD88', 'DO52649') |
| ('MYD88', 'DO52651') |
| ('MYD88', 'DO52653') |
| ('MYD88', 'DO52665') |
| ('MYD88', 'DO52670') |
| ('MYD88', 'DO6352') |
| ('MYD88', 'DO6492') |
| ('MYD88', 'DO6754') |
| ('MYO18A', 'DO51964') |
| ('MYO5A', 'DO35083') |
| ('MYO5A', 'DO45299') |
| ('MYO5A', 'DO48682') |
| ('Metazoa_SRP', 'DO49454') |
| ('N4BP2', 'DO218693') |
| ('N4BP2', 'DO35083') |
| ('NABP1', 'DO49442') |
| ('NALCN-AS1', 'DO49454') |
| ('NALCN-AS1', 'DO51964') |
| ('NANOS2', 'DO50844') |
| ('NBEA', 'DO220873') |
| ('NBEA', 'DO220877') |
| ('NBEA', 'DO51958') |
| ('NCLN', 'DO49442') |
| ('NDC80', 'DO49442') |
| ('NELFA', 'DO51954') |
| ('NF1', 'DO218031') |
| ('NF1', 'DO218243') |
| ('NF1', 'DO51960') |
| ('NF1', 'DO52760') |
| ('NF2', 'DO51959') |
| ('NFATC3', 'DO49454') |
| ('NFKBID', 'DO49442') |
| ('NFKBIE', 'DO220851') |
| ('NFKBIE', 'DO220902') |
| ('NFKBIE', 'DO52703') |
| ('NFKBIE', 'DO6428') |
| ('NFYB', 'DO52743') |
| ('NHLH1', 'DO52512') |
| ('NLGN4X', 'DO52743') |
| ('NONO', 'DO220878') |
| ('NONO', 'DO35083') |
| ('NONO', 'DO44740') |
| ('NOTCH1', 'DO52698') |
| ('NOTCH1', 'DO52712') |
| ('NOTCH1', 'DO6434') |
| ('NOTCH1', 'DO6549') |
| ('NOTCH1', 'DO6742') |
| ('NOTCH1', 'DO6934') |
| ('NOTCH1', 'DO7124') |
| ('NOTCH2', 'DO27783') |
| ('NOTCH2', 'DO27853') |
| ('NOTCH2', 'DO52736') |
| ('NOVA1-AS1', 'DO51964') |
| ('NPAS2', 'DO49454') |
| ('NPM1', 'DO51953') |
| ('NPSR1-AS1', 'DO49454') |
| ('NPSR1-AS1', 'DO51954') |
| ('NR2F2-AS1', 'DO52743') |
| ('NRAS', 'DO220852') |
| ('NRAS', 'DO220853') |
| ('NRAS', 'DO220857') |
| ('NRAS', 'DO220861') |
| ('NRAS', 'DO220862') |
| ('NRAS', 'DO220873') |
| ('NRAS', 'DO220878') |
| ('NRAS', 'DO220880') |
| ('NRAS', 'DO220882') |
| ('NRAS', 'DO220885') |
| ('NRAS', 'DO220890') |
| ('NRAS', 'DO220893') |
| ('NRAS', 'DO220913') |
| ('NRAS', 'DO221548') |
| ('NRAS', 'DO45183') |
| ('NRAS', 'DO49087') |
| ('NRAS', 'DO49533') |
| ('NRAS', 'DO52674') |
| ('NRAS', 'DO52691') |
| ('NREP-AS1', 'DO52510') |
| ('NRG1', 'DO10858') |
| ('NRG1', 'DO217896') |
| ('NRG1', 'DO50306') |
| ('NRG1', 'DO50328') |
| ('NRG1', 'DO50350') |
| ('NRG1', 'DO50402') |
| ('NRG1', 'DO51962') |
| ('NRGN', 'DO51954') |
| ('NSD1', 'DO36223') |
| ('NT5DC4', 'DO51954') |
| ('NTF4', 'DO49454') |
| ('NUFIP1', 'DO49442') |
| ('NUP188', 'DO49454') |
| ('NUP214', 'DO23509') |
| ('NUP214', 'DO51078') |
| ('OIT3', 'DO51964') |
| ('OR14A2', 'DO52510') |
| ('OR2H1', 'DO49442') |
| ('OR52M1', 'DO49454') |
| ('OR5D18', 'DO51954') |
| ('OR8B8', 'DO51954') |
| ('OTX2-AS1', 'DO49454') |
| ('OTX2-AS1', 'DO52736') |
| ('P2RY2', 'DO52512') |
| ('P2RY8', 'DO27829') |
| ('P2RY8', 'DO52684') |
| ('PABPC1', 'DO33168') |
| ('PABPC1', 'DO49079') |
| ('PAFAH1B2', 'DO220877') |
| ('PAFAH1B2', 'DO220898') |
| ('PAIP2B', 'DO49454') |
| ('PARP4', 'DO49442') |
| ('PASD1', 'DO51954') |
| ('PAX5', 'DO220859') |
| ('PAX5', 'DO220902') |
| ('PAX5', 'DO45055') |
| ('PAX5', 'DO52686') |
| ('PBRM1', 'DO218031') |
| ('PBRM1', 'DO218243') |
| ('PBRM1', 'DO221540') |
| ('PBRM1', 'DO46957') |
| ('PBRM1', 'DO47072') |
| ('PBRM1', 'DO51965') |
| ('PBX1', 'DO10840') |
| ('PBX1', 'DO220851') |
| ('PBX1', 'DO220862') |
| ('PBX1', 'DO220875') |
| ('PBX1', 'DO220885') |
| ('PBX1', 'DO220891') |
| ('PBX1', 'DO220903') |
| ('PBX1', 'DO48682') |
| ('PCBP1-AS1', 'DO51954') |
| ('PCDHA5', 'DO49454') |
| ('PCDHB3', 'DO51954') |
| ('PCDHGA6', 'DO49442') |
| ('PDE10A', 'DO51954') |
| ('PDE3A', 'DO51964') |
| ('PDE4B', 'DO220874') |
| ('PDE4DIP', 'DO220882') |
| ('PDE4DIP', 'DO33200') |
| ('PDE4DIP', 'DO49442') |
| ('PDE4DIP', 'DO51964') |
| ('PDGFRB', 'DO36221') |
| ('PDGFRB', 'DO48577') |
| ('PEG10', 'DO51954') |
| ('PEX7', 'DO51954') |
| ('PGM5P2', 'DO220861') |
| ('PHF6', 'DO52740') |
| ('PHOX2B', 'DO218693') |
| ('PHOX2B', 'DO27767') |
| ('PHOX2B', 'DO45201') |
| ('PHOX2B', 'DO50342') |
| ('PI3', 'DO51954') |
| ('PICALM', 'DO45094') |
| ('PICALM', 'DO51548') |
| ('PICK1', 'DO51964') |
| ('PIK3CA', 'DO1007') |
| ('PIK3CA', 'DO1013') |
| ('PIK3CA', 'DO1076') |
| ('PIK3CA', 'DO217786') |
| ('PIK3CA', 'DO217826') |
| ('PIK3CA', 'DO217836') |
| ('PIK3CA', 'DO217887') |
| ('PIK3CA', 'DO217908') |
| ('PIK3CA', 'DO217939') |
| ('PIK3CA', 'DO217950') |
| ('PIK3CA', 'DO217962') |
| ('PIK3CA', 'DO218060') |
| ('PIK3CA', 'DO218072') |
| ('PIK3CA', 'DO218173') |
| ('PIK3CA', 'DO218174') |
| ('PIK3CA', 'DO218180') |
| ('PIK3CA', 'DO218205') |
| ('PIK3CA', 'DO218347') |
| ('PIK3CA', 'DO218489') |
| ('PIK3CA', 'DO218506') |
| ('PIK3CA', 'DO218611') |
| ('PIK3CA', 'DO218656') |
| ('PIK3CA', 'DO218693') |
| ('PIK3CA', 'DO218736') |
| ('PIK3CA', 'DO218742') |
| ('PIK3CA', 'DO218770') |
| ('PIK3CA', 'DO220820') |
| ('PIK3CA', 'DO220821') |
| ('PIK3CA', 'DO220822') |
| ('PIK3CA', 'DO220825') |
| ('PIK3CA', 'DO220828') |
| ('PIK3CA', 'DO33264') |
| ('PIK3CA', 'DO35620') |
| ('PIK3CA', 'DO36080') |
| ('PIK3CA', 'DO45067') |
| ('PIK3CA', 'DO45177') |
| ('PIK3CA', 'DO45303') |
| ('PIK3CA', 'DO48915') |
| ('PIK3CA', 'DO49138') |
| ('PIK3CA', 'DO50323') |
| ('PIK3CA', 'DO50365') |
| ('PIK3CA', 'DO50392') |
| ('PIK3CA', 'DO50449') |
| ('PIK3CA', 'DO50453') |
| ('PIK3CA', 'DO50793') |
| ('PIK3CA', 'DO51133') |
| ('PIK3CA', 'DO51964') |
| ('PIK3CA', 'DO52558') |
| ('PIK3CG', 'DO51964') |
| ('PIM1', 'DO27779') |
| ('PIM1', 'DO27833') |
| ('PIM1', 'DO27853') |
| ('PIM1', 'DO52647') |
| ('PIM1', 'DO52650') |
| ('PIM1', 'DO52651') |
| ('PIM1', 'DO52659') |
| ('PIM1', 'DO52662') |
| ('PIM1', 'DO52666') |
| ('PIM1', 'DO52670') |
| ('PIM1', 'DO52672') |
| ('PIM1', 'DO52675') |
| ('PIM1', 'DO52685') |
| ('PIM1', 'DO52686') |
| ('PIM1', 'DO52692') |
| ('PKD1L1', 'DO49454') |
| ('PKD1L3', 'DO49442') |
| ('PLEKHA8P1', 'DO49454') |
| ('PLEKHG6', 'DO46980') |
| ('PMS2', 'DO51958') |
| ('PMS2CL', 'DO52510') |
| ('PNMA1', 'DO52512') |
| ('PNMA5', 'DO49454') |
| ('POT1', 'DO220891') |
| ('POT1', 'DO220900') |
| ('POU2AF1', 'DO220882') |
| ('POU2AF1', 'DO220903') |
| ('PPFIA1', 'DO51954') |
| ('PPIP5K2', 'DO51964') |
| ('PPP1R12C', 'DO52737') |
| ('PPP1R15A', 'DO49442') |
| ('PPP1R26-AS1', 'DO52736') |
| ('PPP1R35', 'DO51954') |
| ('PPP2R1A', 'DO218693') |
| ('PPP2R1A', 'DO45299') |
| ('PPP2R2A', 'DO23522') |
| ('PPP6C', 'DO220857') |
| ('PPP6C', 'DO220912') |
| ('PRCC', 'DO51954') |
| ('PREX2', 'DO220875') |
| ('PREX2', 'DO220877') |
| ('PREX2', 'DO220883') |
| ('PREX2', 'DO220884') |
| ('PREX2', 'DO220886') |
| ('PREX2', 'DO220895') |
| ('PREX2', 'DO220902') |
| ('PREX2', 'DO220908') |
| ('PREX2', 'DO45081') |
| ('PREX2', 'DO49484') |
| ('PREX2', 'DO50449') |
| ('PREX2', 'DO51491') |
| ('PRKAR1A', 'DO52752') |
| ('PRKCA', 'DO49442') |
| ('PRKD1', 'DO220877') |
| ('PRKD1', 'DO220886') |
| ('PRKY', 'DO52743') |
| ('PROM2', 'DO52736') |
| ('PROX1-AS1', 'DO49442') |
| ('PROX1-AS1', 'DO51964') |
| ('PRPF40B', 'DO51486') |
| ('PRPF40B', 'DO51521') |
| ('PRPF40B', 'DO51538') |
| ('PRRC2C', 'DO218698') |
| ('PRRX1', 'DO220874') |
| ('PRRX1', 'DO51056') |
| ('PSTK', 'DO51954') |
| ('PTEN', 'DO217826') |
| ('PTEN', 'DO217907') |
| ('PTEN', 'DO218478') |
| ('PTEN', 'DO220821') |
| ('PTEN', 'DO220882') |
| ('PTEN', 'DO220907') |
| ('PTEN', 'DO220912') |
| ('PTEN', 'DO45293') |
| ('PTEN', 'DO45299') |
| ('PTEN', 'DO48578') |
| ('PTEN', 'DO48889') |
| ('PTEN', 'DO50337') |
| ('PTEN', 'DO50412') |
| ('PTEN', 'DO51497') |
| ('PTEN', 'DO51953') |
| ('PTEN', 'DO51960') |
| ('PTEN', 'DO52558') |
| ('PTEN', 'DO52591') |
| ('PTK6', 'DO52510') |
| ('PTPN13', 'DO35083') |
| ('PTPN13', 'DO49420') |
| ('PTPRB', 'DO220875') |
| ('PTPRB', 'DO220879') |
| ('PTPRB', 'DO220893') |
| ('PTPRB', 'DO220902') |
| ('PTPRB', 'DO220907') |
| ('PTPRC', 'DO220877') |
| ('PTPRC', 'DO220891') |
| ('PTPRD', 'DO220903') |
| ('PTPRD', 'DO220906') |
| ('PTPRT', 'DO220851') |
| ('PTPRT', 'DO220855') |
| ('PTPRT', 'DO220860') |
| ('PTPRT', 'DO220861') |
| ('PTPRT', 'DO220872') |
| ('PTPRT', 'DO220874') |
| ('PTPRT', 'DO220877') |
| ('PTPRT', 'DO220882') |
| ('PTPRT', 'DO220889') |
| ('PTPRT', 'DO220891') |
| ('PTPRT', 'DO220899') |
| ('PTPRT', 'DO220902') |
| ('PTPRT', 'DO220903') |
| ('PTPRT', 'DO220906') |
| ('PTPRT', 'DO220909') |
| ('PTPRT', 'DO220912') |
| ('PTPRT', 'DO33600') |
| ('PTPRT', 'DO50338') |
| ('PTPRT', 'DO50387') |
| ('PTPRT', 'DO51496') |
| ('PTPRT', 'DO52513') |
| ('PUM1', 'DO52736') |
| ('PVRL4', 'DO51964') |
| ('PWWP2A', 'DO45161') |
| ('PWWP2A', 'DO48732') |
| ('QKI', 'DO51962') |
| ('RABEP1', 'DO51958') |
| ('RABGEF1', 'DO49442') |
| ('RAD1', 'DO51964') |
| ('RAET1K', 'DO52736') |
| ('RALGDS', 'DO45299') |
| ('RALGDS', 'DO52685') |
| ('RAPGEF4-AS1', 'DO49442') |
| ('RAPGEFL1', 'DO51964') |
| ('RASA2', 'DO52510') |
| ('RASGRP1', 'DO49454') |
| ('RB1', 'DO51954') |
| ('RB1', 'DO51962') |
| ('RB1', 'DO52544') |
| ('RB1', 'DO52558') |
| ('RBFOX2', 'DO52510') |
| ('RBM10', 'DO217850') |
| ('RBM10', 'DO51473') |
| ('RBM10', 'DO52738') |
| ('RBM15', 'DO220852') |
| ('RBM15', 'DO220879') |
| ('REL', 'DO45299') |
| ('REL', 'DO46420') |
| ('REL', 'DO48577') |
| ('REXO1L1', 'DO49442') |
| ('RFESD', 'DO52736') |
| ('RFTN1P1', 'DO51954') |
| ('RGS18', 'DO51964') |
| ('RGS7', 'DO220857') |
| ('RGS7', 'DO220877') |
| ('RGS7', 'DO220878') |
| ('RGS7', 'DO220879') |
| ('RHBDL3', 'DO51954') |
| ('RHOA', 'DO27764') |
| ('RHOA', 'DO27767') |
| ('RHOA', 'DO27769') |
| ('RHOU', 'DO49442') |
| ('RMI2', 'DO51960') |
| ('RN7SKP104', 'DO46885') |
| ('RN7SKP149', 'DO49454') |
| ('RN7SKP227', 'DO51954') |
| ('RN7SL139P', 'DO49442') |
| ('RN7SL170P', 'DO51964') |
| ('RN7SL251P', 'DO51964') |
| ('RN7SL278P', 'DO49454') |
| ('RN7SL300P', 'DO49442') |
| ('RN7SL33P', 'DO49454') |
| ('RN7SL357P', 'DO51964') |
| ('RN7SL564P', 'DO51954') |
| ('RN7SL572P', 'DO51964') |
| ('RN7SL575P', 'DO52510') |
| ('RN7SL600P', 'DO51964') |
| ('RN7SL637P', 'DO51964') |
| ('RN7SL644P', 'DO49454') |
| ('RN7SL649P', 'DO51964') |
| ('RN7SL659P', 'DO52510') |
| ('RN7SL7P', 'DO51954') |
| ('RN7SL865P', 'DO49454') |
| ('RN7SL89P', 'DO51954') |
| ('RNA5SP116', 'DO51964') |
| ('RNA5SP24', 'DO49454') |
| ('RNA5SP358', 'DO51954') |
| ('RNA5SP36', 'DO52510') |
| ('RNA5SP403', 'DO51954') |
| ('RNA5SP459', 'DO52737') |
| ('RNA5SP474', 'DO49454') |
| ('RNA5SP504', 'DO51964') |
| ('RNA5SP508', 'DO49442') |
| ('RNA5SP62', 'DO51964') |
| ('RNF215', 'DO49454') |
| ('RNF217', 'DO51964') |
| ('RNF219-AS1', 'DO49442') |
| ('RNF219-AS1', 'DO49454') |
| ('RNF219-AS1', 'DO51954') |
| ('RNF219-AS1', 'DO51964') |
| ('RNF43', 'DO218031') |
| ('RNF43', 'DO218243') |
| ('RNF43', 'DO220886') |
| ('RNF43', 'DO220911') |
| ('RNMT', 'DO51964') |
| ('RNPC3', 'DO51964') |
| ('RNU1-59P', 'DO51954') |
| ('RNU2-54P', 'DO49454') |
| ('RNU4-57P', 'DO51954') |
| ('RNU4-65P', 'DO52510') |
| ('RNU6-102P', 'DO51954') |
| ('RNU6-1047P', 'DO51954') |
| ('RNU6-1070P', 'DO51964') |
| ('RNU6-1163P', 'DO49442') |
| ('RNU6-133P', 'DO49454') |
| ('RNU6-204P', 'DO49442') |
| ('RNU6-227P', 'DO49454') |
| ('RNU6-243P', 'DO51964') |
| ('RNU6-439P', 'DO49442') |
| ('RNU6-442P', 'DO51954') |
| ('RNU6-52P', 'DO51954') |
| ('RNU6-533P', 'DO49454') |
| ('RNU6-543P', 'DO51954') |
| ('RNU6-656P', 'DO51954') |
| ('RNU6-741P', 'DO51964') |
| ('RNU7-136P', 'DO51964') |
| ('RNU7-174P', 'DO49454') |
| ('RNU7-24P', 'DO49442') |
| ('RNY4P27', 'DO51964') |
| ('ROBO2', 'DO220877') |
| ('ROBO2', 'DO220898') |
| ('ROBO2', 'DO35733') |
| ('ROBO2', 'DO45255') |
| ('ROBO2', 'DO50329') |
| ('ROBO2', 'DO51959') |
| ('ROBO2', 'DO52512') |
| ('ROBO2', 'DO52675') |
| ('ROS1', 'DO52742') |
| ('RP1-13P20.6', 'DO49442') |
| ('RP1-167A14.2', 'DO49442') |
| ('RP1-167F1.2', 'DO51964') |
| ('RP1-23K20.2', 'DO49454') |
| ('RP1-240B8.3', 'DO51954') |
| ('RP1-261D10.2', 'DO49454') |
| ('RP1-272J12.1', 'DO49454') |
| ('RP1-272J12.1', 'DO52743') |
| ('RP1-28O10.1', 'DO51954') |
| ('RP1-65P5.1', 'DO51954') |
| ('RP1-91G5.3', 'DO52510') |
| ('RP1-93I3.1', 'DO51954') |
| ('RP11-100L22.2', 'DO51954') |
| ('RP11-1017G21.4', 'DO51954') |
| ('RP11-103J8.1', 'DO49454') |
| ('RP11-1069G10.1', 'DO51964') |
| ('RP11-1084E5.1', 'DO51954') |
| ('RP11-1103G16.1', 'DO46412') |
| ('RP11-1103G16.1', 'DO51954') |
| ('RP11-1129I3.1', 'DO35350') |
| ('RP11-1129I3.1', 'DO51954') |
| ('RP11-114H24.5', 'DO49454') |
| ('RP11-115D19.1', 'DO49442') |
| ('RP11-115D19.1', 'DO51964') |
| ('RP11-122D10.1', 'DO52743') |
| ('RP11-125B21.2', 'DO51954') |
| ('RP11-1277A3.2', 'DO51954') |
| ('RP11-130F10.1', 'DO49442') |
| ('RP11-135J2.4', 'DO52743') |
| ('RP11-13G14.4', 'DO51954') |
| ('RP11-13J10.1', 'DO49442') |
| ('RP11-13J10.1', 'DO49454') |
| ('RP11-13J10.1', 'DO51954') |
| ('RP11-142C4.6', 'DO51954') |
| ('RP11-144L1.4', 'DO51954') |
| ('RP11-145A3.1', 'DO51954') |
| ('RP11-150C16.1', 'DO51964') |
| ('RP11-152L20.3', 'DO49442') |
| ('RP11-152L20.3', 'DO51964') |
| ('RP11-152L20.3', 'DO52737') |
| ('RP11-152P17.2', 'DO49454') |
| ('RP11-154D6.1', 'DO51964') |
| ('RP11-154D6.1', 'DO52736') |
| ('RP11-155G15.2', 'DO52737') |
| ('RP11-158J3.2', 'DO51954') |
| ('RP11-159L20.2', 'DO51954') |
| ('RP11-15B17.1', 'DO49454') |
| ('RP11-163M18.1', 'DO51964') |
| ('RP11-166B2.8', 'DO51954') |
| ('RP11-167H9.4', 'DO49442') |
| ('RP11-167N24.3', 'DO52510') |
| ('RP11-175P19.2', 'DO51964') |
| ('RP11-17A1.3', 'DO51954') |
| ('RP11-17E2.2', 'DO51954') |
| ('RP11-17E2.2', 'DO51964') |
| ('RP11-17E2.2', 'DO52510') |
| ('RP11-17E2.2', 'DO52743') |
| ('RP11-184A2.3', 'DO52736') |
| ('RP11-193H5.1', 'DO49442') |
| ('RP11-196E1.3', 'DO51964') |
| ('RP11-1L12.3', 'DO49454') |
| ('RP11-230B22.1', 'DO49454') |
| ('RP11-231I13.2', 'DO220891') |
| ('RP11-231I13.2', 'DO49454') |
| ('RP11-231I13.2', 'DO51954') |
| ('RP11-231P20.2', 'DO51954') |
| ('RP11-23D24.2', 'DO51954') |
| ('RP11-274B21.1', 'DO49454') |
| ('RP11-274B21.1', 'DO51954') |
| ('RP11-280O1.2', 'DO51954') |
| ('RP11-284M14.1', 'DO49454') |
| ('RP11-285J16.1', 'DO51954') |
| ('RP11-286H14.4', 'DO49454') |
| ('RP11-290F24.3', 'DO49442') |
| ('RP11-310I9.1', 'DO52736') |
| ('RP11-313J2.1', 'DO49454') |
| ('RP11-324H6.5', 'DO51954') |
| ('RP11-326E22.1', 'DO51954') |
| ('RP11-32K4.1', 'DO49442') |
| ('RP11-32K4.1', 'DO51954') |
| ('RP11-32K4.1', 'DO51964') |
| ('RP11-32K4.1', 'DO52510') |
| ('RP11-32K4.1', 'DO52736') |
| ('RP11-333E1.1', 'DO49454') |
| ('RP11-33N16.3', 'DO49454') |
| ('RP11-33N16.3', 'DO51964') |
| ('RP11-342D14.1', 'DO49442') |
| ('RP11-342D14.1', 'DO51964') |
| ('RP11-343D2.11', 'DO51954') |
| ('RP11-356I2.4', 'DO49442') |
| ('RP11-357C3.3', 'DO51964') |
| ('RP11-366F6.2', 'DO51964') |
| ('RP11-368J21.3', 'DO49454') |
| ('RP11-368M16.3', 'DO51964') |
| ('RP11-370I10.6', 'DO51964') |
| ('RP11-379F4.4', 'DO51954') |
| ('RP11-380P13.1', 'DO49442') |
| ('RP11-382A20.4', 'DO49454') |
| ('RP11-385J1.2', 'DO51964') |
| ('RP11-3B12.1', 'DO51954') |
| ('RP11-3B12.1', 'DO51964') |
| ('RP11-3B12.1', 'DO52737') |
| ('RP11-402C9.1', 'DO49454') |
| ('RP11-418J17.1', 'DO49442') |
| ('RP11-420N3.2', 'DO49442') |
| ('RP11-420N3.2', 'DO49454') |
| ('RP11-420N3.2', 'DO51954') |
| ('RP11-420N3.2', 'DO51964') |
| ('RP11-420N3.2', 'DO52512') |
| ('RP11-420N3.2', 'DO52737') |
| ('RP11-422J8.1', 'DO51964') |
| ('RP11-423H2.3', 'DO51964') |
| ('RP11-428C19.4', 'DO49454') |
| ('RP11-429B14.1', 'DO51964') |
| ('RP11-431M7.2', 'DO51954') |
| ('RP11-431M7.2', 'DO52510') |
| ('RP11-431M7.3', 'DO51954') |
| ('RP11-435M3.2', 'DO51964') |
| ('RP11-438D8.2', 'DO49442') |
| ('RP11-439L18.3', 'DO51954') |
| ('RP11-439L18.3', 'DO51964') |
| ('RP11-445F12.1', 'DO51954') |
| ('RP11-446J8.1', 'DO51954') |
| ('RP11-446J8.1', 'DO52510') |
| ('RP11-454C18.2', 'DO51954') |
| ('RP11-482H16.1', 'DO52743') |
| ('RP11-492A10.1', 'DO52736') |
| ('RP11-505P4.7', 'DO51964') |
| ('RP11-508N22.8', 'DO52512') |
| ('RP11-513G19.1', 'DO51954') |
| ('RP11-519G16.3', 'DO51964') |
| ('RP11-521M14.1', 'DO49442') |
| ('RP11-525K10.3', 'DO49442') |
| ('RP11-525K10.3', 'DO51954') |
| ('RP11-526D8.7', 'DO51954') |
| ('RP11-532F12.5', 'DO51964') |
| ('RP11-538P18.2', 'DO51954') |
| ('RP11-541P9.3', 'DO48727') |
| ('RP11-541P9.3', 'DO51954') |
| ('RP11-541P9.3', 'DO51964') |
| ('RP11-542K23.7', 'DO52743') |
| ('RP11-54D18.3', 'DO51964') |
| ('RP11-550I24.2', 'DO52510') |
| ('RP11-550P17.5', 'DO51954') |
| ('RP11-550P17.5', 'DO51964') |
| ('RP11-551L14.1', 'DO51954') |
| ('RP11-556E13.1', 'DO49442') |
| ('RP11-556E13.1', 'DO51954') |
| ('RP11-562L8.1', 'DO49454') |
| ('RP11-562L8.1', 'DO51964') |
| ('RP11-572M11.4', 'DO49454') |
| ('RP11-572M11.4', 'DO51964') |
| ('RP11-57H12.5', 'DO49442') |
| ('RP11-586K2.1', 'DO49454') |
| ('RP11-586K2.1', 'DO51954') |
| ('RP11-586K2.1', 'DO51964') |
| ('RP11-586K2.1', 'DO52510') |
| ('RP11-58B2.1', 'DO51954') |
| ('RP11-611E13.2', 'DO51964') |
| ('RP11-624C23.1', 'DO49442') |
| ('RP11-624C23.1', 'DO49454') |
| ('RP11-624C23.1', 'DO51964') |
| ('RP11-624L4.1', 'DO51954') |
| ('RP11-624L4.1', 'DO52510') |
| ('RP11-631F7.1', 'DO52736') |
| ('RP11-634B7.4', 'DO49454') |
| ('RP11-634B7.4', 'DO51964') |
| ('RP11-644F5.16', 'DO52510') |
| ('RP11-649A16.1', 'DO49442') |
| ('RP11-649A16.1', 'DO51954') |
| ('RP11-649A16.1', 'DO51964') |
| ('RP11-657O9.1', 'DO52510') |
| ('RP11-665G4.1', 'DO49442') |
| ('RP11-665G4.1', 'DO51964') |
| ('RP11-66D17.5', 'DO51964') |
| ('RP11-678G14.2', 'DO51964') |
| ('RP11-679C8.2', 'DO49454') |
| ('RP11-679C8.2', 'DO51964') |
| ('RP11-680B3.2', 'DO49442') |
| ('RP11-680F20.9', 'DO49454') |
| ('RP11-692D12.1', 'DO51954') |
| ('RP11-693J15.4', 'DO49442') |
| ('RP11-702H23.4', 'DO51954') |
| ('RP11-706C16.8', 'DO51954') |
| ('RP11-707M1.1', 'DO49454') |
| ('RP11-707M1.1', 'DO51954') |
| ('RP11-708B6.2', 'DO49454') |
| ('RP11-708B6.2', 'DO52510') |
| ('RP11-731J8.2', 'DO52736') |
| ('RP11-742B18.1', 'DO49454') |
| ('RP11-742B18.1', 'DO51954') |
| ('RP11-744N12.3', 'DO51954') |
| ('RP11-752G15.9', 'DO51954') |
| ('RP11-75N4.2', 'DO51954') |
| ('RP11-76N22.2', 'DO51964') |
| ('RP11-770E5.1', 'DO49454') |
| ('RP11-770E5.1', 'DO51954') |
| ('RP11-77K12.5', 'DO49454') |
| ('RP11-788M5.3', 'DO51954') |
| ('RP11-800A3.4', 'DO49442') |
| ('RP11-804A23.1', 'DO49454') |
| ('RP11-804A23.2', 'DO46881') |
| ('RP11-804A23.2', 'DO51954') |
| ('RP11-804N13.1', 'DO51964') |
| ('RP11-81K13.1', 'DO52737') |
| ('RP11-820L6.1', 'DO51954') |
| ('RP11-820L6.1', 'DO51964') |
| ('RP11-826N14.1', 'DO51964') |
| ('RP11-846C15.2', 'DO49442') |
| ('RP11-84A1.3', 'DO49442') |
| ('RP11-867G2.8', 'DO49454') |
| ('RP11-86L19.2', 'DO51954') |
| ('RP11-87M18.2', 'DO49454') |
| ('RP11-89K10.1', 'DO49454') |
| ('RP11-89K10.1', 'DO51964') |
| ('RP11-92C4.3', 'DO51964') |
| ('RP11-933H2.4', 'DO49442') |
| ('RP11-93K22.6', 'DO51964') |
| ('RP11-944L7.4', 'DO52737') |
| ('RP11-978I15.10', 'DO51954') |
| ('RP11-98D18.15', 'DO51964') |
| ('RP13-492C18.2', 'DO51954') |
| ('RP13-492C18.2', 'DO51964') |
| ('RP13-578N3.3', 'DO52512') |
| ('RP3-323P13.2', 'DO51954') |
| ('RP3-340N1.5', 'DO51964') |
| ('RP3-399L15.3', 'DO51954') |
| ('RP3-399L15.3', 'DO51964') |
| ('RP3-428L16.1', 'DO49442') |
| ('RP4-605O3.4', 'DO49442') |
| ('RP4-630C24.3', 'DO51954') |
| ('RP4-651E10.4', 'DO51964') |
| ('RP4-694A7.4', 'DO51964') |
| ('RP4-724E13.2', 'DO49454') |
| ('RP4-735C1.4', 'DO52510') |
| ('RP4-755D9.1', 'DO49454') |
| ('RP4-756H11.3', 'DO52736') |
| ('RP4-777D9.2', 'DO52510') |
| ('RP4-798P15.3', 'DO49442') |
| ('RP5-1091N2.9', 'DO49454') |
| ('RP5-1121H13.4', 'DO51954') |
| ('RP5-896L10.1', 'DO51964') |
| ('RP5-905H7.3', 'DO51954') |
| ('RP5-921G16.1', 'DO49442') |
| ('RP5-921G16.1', 'DO52736') |
| ('RP5-991G20.1', 'DO51964') |
| ('RPA3-AS1', 'DO52510') |
| ('RPL10', 'DO27793') |
| ('RPL10', 'DO27795') |
| ('RPL22', 'DO218693') |
| ('RPL22', 'DO35083') |
| ('RPL22', 'DO45287') |
| ('RPL22', 'DO48682') |
| ('RPL23AP79', 'DO51954') |
| ('RUNDC1', 'DO51964') |
| ('RUNX1T1', 'DO220828') |
| ('RUNX1T1', 'DO220846') |
| ('RUNX1T1', 'DO220851') |
| ('RUNX1T1', 'DO220862') |
| ('RUNX1T1', 'DO220887') |
| ('RUNX1T1', 'DO220899') |
| ('RUNX1T1', 'DO27851') |
| ('RUNX1T1', 'DO45159') |
| ('RUNX1T1', 'DO45299') |
| ('RUNX1T1', 'DO50314') |
| ('RUNX1T1', 'DO50348') |
| ('RUNX1T1', 'DO51126') |
| ('RUSC2', 'DO45141') |
| ('SAGE1', 'DO51964') |
| ('SCARNA17', 'DO49442') |
| ('SCN10A', 'DO49454') |
| ('SCN4A', 'DO51954') |
| ('SCRG1', 'DO49454') |
| ('SCRN1', 'DO51964') |
| ('SDHA', 'DO36221') |
| ('SDHA', 'DO51474') |
| ('SEC14L4', 'DO51954') |
| ('SEC24B-AS1', 'DO51954') |
| ('SEC24B-AS1', 'DO52743') |
| ('SEC63', 'DO49454') |
| ('SEMA5A', 'DO49454') |
| ('SEPT14', 'DO51964') |
| ('SERPINB11', 'DO52736') |
| ('SERPINB9', 'DO52510') |
| ('SETBP1', 'DO220895') |
| ('SETBP1', 'DO220906') |
| ('SETBP1', 'DO48893') |
| ('SETBP1', 'DO50331') |
| ('SETBP1', 'DO50441') |
| ('SETBP1', 'DO51953') |
| ('SETBP1', 'DO52739') |
| ('SETD2', 'DO52150') |
| ('SETD2', 'DO6934') |
| ('SF3B1', 'DO217786') |
| ('SF3B1', 'DO217826') |
| ('SF3B1', 'DO217931') |
| ('SF3B1', 'DO218560') |
| ('SF3B1', 'DO218736') |
| ('SF3B1', 'DO221544') |
| ('SF3B1', 'DO35216') |
| ('SF3B1', 'DO36221') |
| ('SF3B1', 'DO45297') |
| ('SF3B1', 'DO46787') |
| ('SF3B1', 'DO49439') |
| ('SF3B1', 'DO50309') |
| ('SF3B1', 'DO50818') |
| ('SF3B1', 'DO51520') |
| ('SF3B1', 'DO52708') |
| ('SF3B1', 'DO6360') |
| ('SF3B1', 'DO6362') |
| ('SF3B1', 'DO6690') |
| ('SFRP4', 'DO51954') |
| ('SFXN1', 'DO49442') |
| ('SGK1', 'DO221123') |
| ('SGK1', 'DO27779') |
| ('SGK1', 'DO27809') |
| ('SGK1', 'DO27827') |
| ('SGK1', 'DO27853') |
| ('SGK1', 'DO51958') |
| ('SGK1', 'DO51965') |
| ('SGK1', 'DO52650') |
| ('SGK1', 'DO52664') |
| ('SGK1', 'DO52674') |
| ('SGK1', 'DO52684') |
| ('SGK1', 'DO52686') |
| ('SGK1', 'DO52692') |
| ('SGK223', 'DO52736') |
| ('SH2B3', 'DO52736') |
| ('SHANK2', 'DO51964') |
| ('SHANK3', 'DO49454') |
| ('SIGLEC6', 'DO51964') |
| ('SIGLEC9', 'DO49454') |
| ('SIGLECL1', 'DO49442') |
| ('SIX1', 'DO218693') |
| ('SIX1', 'DO220878') |
| ('SLAIN2', 'DO49454') |
| ('SLAMF6', 'DO51954') |
| ('SLC10A7', 'DO51964') |
| ('SLC15A1', 'DO52510') |
| ('SLC16A10', 'DO51964') |
| ('SLC18A2', 'DO51954') |
| ('SLC24A5', 'DO220877') |
| ('SLC25A32', 'DO51964') |
| ('SLC25A38', 'DO51964') |
| ('SLC25A44', 'DO49442') |
| ('SLC26A2', 'DO51954') |
| ('SLC30A2', 'DO51964') |
| ('SLC31A1', 'DO51954') |
| ('SLC46A1', 'DO49454') |
| ('SLC46A1', 'DO51954') |
| ('SLC5A3', 'DO51954') |
| ('SLC7A14', 'DO51964') |
| ('SLC8A1', 'DO51954') |
| ('SLC8A1-AS1', 'DO51954') |
| ('SLFN5', 'DO51964') |
| ('SMAD2', 'DO220851') |
| ('SMAD2', 'DO220878') |
| ('SMAD2', 'DO220895') |
| ('SMAD2', 'DO48682') |
| ('SMAD4', 'DO218550') |
| ('SMAD4', 'DO34656') |
| ('SMAD4', 'DO35116') |
| ('SMAD4', 'DO35136') |
| ('SMAD4', 'DO35228') |
| ('SMAD4', 'DO49193') |
| ('SMAD4', 'DO50314') |
| ('SMAD4', 'DO50338') |
| ('SMAD4', 'DO50355') |
| ('SMAD4', 'DO50384') |
| ('SMAD4', 'DO50411') |
| ('SMAD4', 'DO51524') |
| ('SMAD4', 'DO51534') |
| ('SMAD4', 'DO51543') |
| ('SMAD4', 'DO51548') |
| ('SMARCA2', 'DO49454') |
| ('SMARCA4', 'DO218506') |
| ('SMARCA4', 'DO220878') |
| ('SMARCA4', 'DO27763') |
| ('SMARCA4', 'DO27795') |
| ('SMARCA4', 'DO27821') |
| ('SMARCA4', 'DO27825') |
| ('SMARCA4', 'DO27851') |
| ('SMARCA4', 'DO35442') |
| ('SMARCA4', 'DO35555') |
| ('SMARCA4', 'DO45299') |
| ('SMARCA4', 'DO48964') |
| ('SMARCA4', 'DO50338') |
| ('SMARCA4', 'DO50382') |
| ('SMARCA4', 'DO51090') |
| ('SMARCA4', 'DO51484') |
| ('SMARCA4', 'DO52763') |
| ('SMARCD2', 'DO220902') |
| ('SMO', 'DO35547') |
| ('SMO', 'DO48888') |
| ('SMO', 'DO48909') |
| ('SMO', 'DO48915') |
| ('SNAPC1', 'DO51954') |
| ('SND1', 'DO220879') |
| ('SND1', 'DO45041') |
| ('SNHG11', 'DO49442') |
| ('SNHG14', 'DO49442') |
| ('SNHG14', 'DO49454') |
| ('SNORA26', 'DO51954') |
| ('SNORA26', 'DO51964') |
| ('SNORA31', 'DO52736') |
| ('SNORA51', 'DO51954') |
| ('SNORA63', 'DO46838') |
| ('SNORD2', 'DO52512') |
| ('SNORD29', 'DO51954') |
| ('SNORD81', 'DO52737') |
| ('SNRPD2', 'DO51954') |
| ('SNX29', 'DO51958') |
| ('SNX29', 'DO51962') |
| ('SNX7', 'DO49442') |
| ('SOCS1', 'DO27779') |
| ('SOCS1', 'DO27851') |
| ('SOCS1', 'DO52648') |
| ('SOCS1', 'DO52661') |
| ('SOCS1', 'DO52663') |
| ('SOCS1', 'DO52675') |
| ('SOCS1', 'DO52679') |
| ('SOCS1', 'DO52692') |
| ('SORL1', 'DO51954') |
| ('SOX21', 'DO51962') |
| ('SP100', 'DO52510') |
| ('SPANXA2-OT1', 'DO52512') |
| ('SPATA3', 'DO49442') |
| ('SPECC1', 'DO218693') |
| ('SPECC1', 'DO45299') |
| ('SPECC1', 'DO51960') |
| ('SPEN', 'DO52747') |
| ('SPHK1', 'DO49454') |
| ('SPOP', 'DO46792') |
| ('SPOP', 'DO46795') |
| ('SPOP', 'DO50431') |
| ('SPOP', 'DO51086') |
| ('SPOP', 'DO51088') |
| ('SPOP', 'DO51158') |
| ('SPOP', 'DO51964') |
| ('SPTBN4', 'DO52736') |
| ('SRD5A2', 'DO51964') |
| ('SRD5A3-AS1', 'DO51954') |
| ('SRGAP2-AS1', 'DO49454') |
| ('SRGAP2B', 'DO51954') |
| ('SRGAP3', 'DO220882') |
| ('SRGAP3', 'DO220899') |
| ('SSPO', 'DO49454') |
| ('SSPO', 'DO51954') |
| ('STAG1', 'DO45189') |
| ('STAG1', 'DO45299') |
| ('STAG2', 'DO51959') |
| ('STAG2', 'DO51965') |
| ('STARD13', 'DO51964') |
| ('STARD4-AS1', 'DO52510') |
| ('STAT5B', 'DO45299') |
| ('STAT5B', 'DO51474') |
| ('STAT6', 'DO27801') |
| ('STAT6', 'DO27835') |
| ('STAT6', 'DO52512') |
| ('STAT6', 'DO52656') |
| ('STAT6', 'DO52664') |
| ('STAT6', 'DO52667') |
| ('STEAP2-AS1', 'DO49454') |
| ('STEAP2-AS1', 'DO52743') |
| ('STIL', 'DO51962') |
| ('STXBP5-AS1', 'DO49442') |
| ('SUCO', 'DO51964') |
| ('SUGT1P3', 'DO49454') |
| ('SULT1C2P1', 'DO52736') |
| ('SUPT20H', 'DO51954') |
| ('SUPT6H', 'DO52737') |
| ('SUSD2', 'DO49442') |
| ('SVILP1', 'DO49442') |
| ('SWT1', 'DO49454') |
| ('SYT4', 'DO52736') |
| ('TAL1', 'DO218306') |
| ('TAL1', 'DO46591') |
| ('TAOK3', 'DO51964') |
| ('TBL1XR1', 'DO1005') |
| ('TBL1XR1', 'DO23543') |
| ('TBL1XR1', 'DO51954') |
| ('TBL1XR1', 'DO51958') |
| ('TCF12', 'DO51965') |
| ('TCF21', 'DO51964') |
| ('TCF7L2', 'DO220878') |
| ('TCF7L2', 'DO45115') |
| ('TCHH', 'DO51954') |
| ('TCL1A', 'DO220857') |
| ('TCL1A', 'DO220886') |
| ('TCL1A', 'DO220891') |
| ('TCL1A', 'DO220903') |
| ('TECTA', 'DO49454') |
| ('TENC1', 'DO51964') |
| ('TERT', 'DO218697') |
| ('TERT', 'DO220905') |
| ('TET2', 'DO51965') |
| ('TET2', 'DO52736') |
| ('TET2', 'DO52744') |
| ('TEX26-AS1', 'DO51964') |
| ('TFAP2B', 'DO51954') |
| ('TFAP2C', 'DO49454') |
| ('TFEB', 'DO10809') |
| ('TFEB', 'DO52699') |
| ('TG', 'DO52736') |
| ('TGFBR2', 'DO10844') |
| ('TGFBR2', 'DO218173') |
| ('TGFBR2', 'DO27851') |
| ('TGFBR2', 'DO51549') |
| ('TGFBR2', 'DO51962') |
| ('TGS1', 'DO51954') |
| ('THEMIS2', 'DO49442') |
| ('THSD1', 'DO51954') |
| ('TLE3', 'DO49454') |
| ('TLR8-AS1', 'DO49454') |
| ('TM4SF1-AS1', 'DO52737') |
| ('TM9SF4', 'DO220881') |
| ('TMEM121', 'DO220872') |
| ('TMEM132B', 'DO52736') |
| ('TMEM154', 'DO51964') |
| ('TMEM161B-AS1', 'DO49442') |
| ('TMEM200C', 'DO51954') |
| ('TMEM200C', 'DO51964') |
| ('TMEM230', 'DO49454') |
| ('TMEM254-AS1', 'DO51954') |
| ('TMEM38B', 'DO51964') |
| ('TMPRSS2', 'DO51965') |
| ('TMPRSS4-AS1', 'DO51954') |
| ('TMSB4X', 'DO221123') |
| ('TMSB4X', 'DO27779') |
| ('TMSB4X', 'DO27783') |
| ('TMSB4X', 'DO27789') |
| ('TMSB4X', 'DO27801') |
| ('TMSB4X', 'DO27807') |
| ('TMSB4X', 'DO27827') |
| ('TMSB4X', 'DO27849') |
| ('TMSB4X', 'DO27851') |
| ('TMSB4X', 'DO27853') |
| ('TMSB4X', 'DO27855') |
| ('TMSB4X', 'DO52655') |
| ('TMSB4X', 'DO52657') |
| ('TMSB4X', 'DO52663') |
| ('TMSB4X', 'DO52664') |
| ('TMSB4X', 'DO52666') |
| ('TMSB4X', 'DO52670') |
| ('TMSB4X', 'DO52672') |
| ('TMSB4X', 'DO52674') |
| ('TMSB4X', 'DO52675') |
| ('TMSB4X', 'DO52679') |
| ('TMSB4X', 'DO52681') |
| ('TMSB4X', 'DO52682') |
| ('TMSB4X', 'DO52684') |
| ('TMSB4X', 'DO52686') |
| ('TMSB4X', 'DO52687') |
| ('TMSB4X', 'DO52692') |
| ('TMSB4X', 'DO52718') |
| ('TNRC18', 'DO52736') |
| ('TNS3', 'DO51954') |
| ('TOP3B', 'DO49454') |
| ('TP53', 'DO1001') |
| ('TP53', 'DO1005') |
| ('TP53', 'DO1010') |
| ('TP53', 'DO1016') |
| ('TP53', 'DO10815') |
| ('TP53', 'DO10839') |
| ('TP53', 'DO10840') |
| ('TP53', 'DO10841') |
| ('TP53', 'DO10843') |
| ('TP53', 'DO10858') |
| ('TP53', 'DO217800') |
| ('TP53', 'DO217818') |
| ('TP53', 'DO217822') |
| ('TP53', 'DO217850') |
| ('TP53', 'DO217987') |
| ('TP53', 'DO218030') |
| ('TP53', 'DO218075') |
| ('TP53', 'DO218088') |
| ('TP53', 'DO218139') |
| ('TP53', 'DO218205') |
| ('TP53', 'DO218227') |
| ('TP53', 'DO218269') |
| ('TP53', 'DO218280') |
| ('TP53', 'DO218282') |
| ('TP53', 'DO218306') |
| ('TP53', 'DO218417') |
| ('TP53', 'DO218428') |
| ('TP53', 'DO218440') |
| ('TP53', 'DO218442') |
| ('TP53', 'DO218462') |
| ('TP53', 'DO218502') |
| ('TP53', 'DO218535') |
| ('TP53', 'DO218550') |
| ('TP53', 'DO218651') |
| ('TP53', 'DO218673') |
| ('TP53', 'DO218684') |
| ('TP53', 'DO218693') |
| ('TP53', 'DO218695') |
| ('TP53', 'DO218709') |
| ('TP53', 'DO218736') |
| ('TP53', 'DO218773') |
| ('TP53', 'DO218796') |
| ('TP53', 'DO218828') |
| ('TP53', 'DO220828') |
| ('TP53', 'DO220857') |
| ('TP53', 'DO220874') |
| ('TP53', 'DO220877') |
| ('TP53', 'DO220881') |
| ('TP53', 'DO220891') |
| ('TP53', 'DO220903') |
| ('TP53', 'DO220904') |
| ('TP53', 'DO220906') |
| ('TP53', 'DO221539') |
| ('TP53', 'DO221542') |
| ('TP53', 'DO222299') |
| ('TP53', 'DO23508') |
| ('TP53', 'DO23509') |
| ('TP53', 'DO23513') |
| ('TP53', 'DO23526') |
| ('TP53', 'DO23535') |
| ('TP53', 'DO23539') |
| ('TP53', 'DO23545') |
| ('TP53', 'DO23550') |
| ('TP53', 'DO23551') |
| ('TP53', 'DO27769') |
| ('TP53', 'DO27775') |
| ('TP53', 'DO27795') |
| ('TP53', 'DO27821') |
| ('TP53', 'DO27825') |
| ('TP53', 'DO27829') |
| ('TP53', 'DO27833') |
| ('TP53', 'DO27851') |
| ('TP53', 'DO32831') |
| ('TP53', 'DO32837') |
| ('TP53', 'DO32860') |
| ('TP53', 'DO32893') |
| ('TP53', 'DO32900') |
| ('TP53', 'DO32980') |
| ('TP53', 'DO33000') |
| ('TP53', 'DO33008') |
| ('TP53', 'DO33032') |
| ('TP53', 'DO33042') |
| ('TP53', 'DO33128') |
| ('TP53', 'DO33160') |
| ('TP53', 'DO33168') |
| ('TP53', 'DO33200') |
| ('TP53', 'DO33208') |
| ('TP53', 'DO33248') |
| ('TP53', 'DO33344') |
| ('TP53', 'DO33376') |
| ('TP53', 'DO33392') |
| ('TP53', 'DO33400') |
| ('TP53', 'DO33408') |
| ('TP53', 'DO33512') |
| ('TP53', 'DO33544') |
| ('TP53', 'DO33600') |
| ('TP53', 'DO34312') |
| ('TP53', 'DO34504') |
| ('TP53', 'DO34608') |
| ('TP53', 'DO34616') |
| ('TP53', 'DO34656') |
| ('TP53', 'DO34720') |
| ('TP53', 'DO34728') |
| ('TP53', 'DO34736') |
| ('TP53', 'DO34785') |
| ('TP53', 'DO34793') |
| ('TP53', 'DO35083') |
| ('TP53', 'DO35085') |
| ('TP53', 'DO35126') |
| ('TP53', 'DO35128') |
| ('TP53', 'DO35148') |
| ('TP53', 'DO35152') |
| ('TP53', 'DO35200') |
| ('TP53', 'DO35228') |
| ('TP53', 'DO35236') |
| ('TP53', 'DO35258') |
| ('TP53', 'DO35376') |
| ('TP53', 'DO35406') |
| ('TP53', 'DO35424') |
| ('TP53', 'DO35454') |
| ('TP53', 'DO45035') |
| ('TP53', 'DO45039') |
| ('TP53', 'DO45041') |
| ('TP53', 'DO45047') |
| ('TP53', 'DO45057') |
| ('TP53', 'DO45065') |
| ('TP53', 'DO45071') |
| ('TP53', 'DO45075') |
| ('TP53', 'DO45083') |
| ('TP53', 'DO45091') |
| ('TP53', 'DO45093') |
| ('TP53', 'DO45161') |
| ('TP53', 'DO45173') |
| ('TP53', 'DO45185') |
| ('TP53', 'DO45265') |
| ('TP53', 'DO45267') |
| ('TP53', 'DO45273') |
| ('TP53', 'DO45299') |
| ('TP53', 'DO46327') |
| ('TP53', 'DO46328') |
| ('TP53', 'DO46330') |
| ('TP53', 'DO46331') |
| ('TP53', 'DO46333') |
| ('TP53', 'DO46334') |
| ('TP53', 'DO46338') |
| ('TP53', 'DO46350') |
| ('TP53', 'DO46352') |
| ('TP53', 'DO46354') |
| ('TP53', 'DO46356') |
| ('TP53', 'DO46358') |
| ('TP53', 'DO46362') |
| ('TP53', 'DO46366') |
| ('TP53', 'DO46372') |
| ('TP53', 'DO46374') |
| ('TP53', 'DO46376') |
| ('TP53', 'DO46378') |
| ('TP53', 'DO46380') |
| ('TP53', 'DO46388') |
| ('TP53', 'DO46390') |
| ('TP53', 'DO46396') |
| ('TP53', 'DO46398') |
| ('TP53', 'DO46402') |
| ('TP53', 'DO46404') |
| ('TP53', 'DO46408') |
| ('TP53', 'DO46412') |
| ('TP53', 'DO46416') |
| ('TP53', 'DO46420') |
| ('TP53', 'DO46448') |
| ('TP53', 'DO46493') |
| ('TP53', 'DO46542') |
| ('TP53', 'DO46551') |
| ('TP53', 'DO46561') |
| ('TP53', 'DO46568') |
| ('TP53', 'DO46571') |
| ('TP53', 'DO46581') |
| ('TP53', 'DO46586') |
| ('TP53', 'DO46597') |
| ('TP53', 'DO46606') |
| ('TP53', 'DO46611') |
| ('TP53', 'DO46783') |
| ('TP53', 'DO46893') |
| ('TP53', 'DO46913') |
| ('TP53', 'DO48679') |
| ('TP53', 'DO48682') |
| ('TP53', 'DO48684') |
| ('TP53', 'DO48715') |
| ('TP53', 'DO48730') |
| ('TP53', 'DO48741') |
| ('TP53', 'DO48757') |
| ('TP53', 'DO49074') |
| ('TP53', 'DO49079') |
| ('TP53', 'DO49087') |
| ('TP53', 'DO49127') |
| ('TP53', 'DO49135') |
| ('TP53', 'DO49138') |
| ('TP53', 'DO49168') |
| ('TP53', 'DO49172') |
| ('TP53', 'DO49178') |
| ('TP53', 'DO49181') |
| ('TP53', 'DO49193') |
| ('TP53', 'DO49204') |
| ('TP53', 'DO49418') |
| ('TP53', 'DO49420') |
| ('TP53', 'DO49421') |
| ('TP53', 'DO49424') |
| ('TP53', 'DO49436') |
| ('TP53', 'DO49448') |
| ('TP53', 'DO49451') |
| ('TP53', 'DO49454') |
| ('TP53', 'DO49463') |
| ('TP53', 'DO49478') |
| ('TP53', 'DO50306') |
| ('TP53', 'DO50309') |
| ('TP53', 'DO50311') |
| ('TP53', 'DO50314') |
| ('TP53', 'DO50316') |
| ('TP53', 'DO50318') |
| ('TP53', 'DO50319') |
| ('TP53', 'DO50320') |
| ('TP53', 'DO50323') |
| ('TP53', 'DO50325') |
| ('TP53', 'DO50326') |
| ('TP53', 'DO50327') |
| ('TP53', 'DO50328') |
| ('TP53', 'DO50329') |
| ('TP53', 'DO50331') |
| ('TP53', 'DO50332') |
| ('TP53', 'DO50334') |
| ('TP53', 'DO50336') |
| ('TP53', 'DO50337') |
| ('TP53', 'DO50338') |
| ('TP53', 'DO50341') |
| ('TP53', 'DO50343') |
| ('TP53', 'DO50345') |
| ('TP53', 'DO50346') |
| ('TP53', 'DO50348') |
| ('TP53', 'DO50350') |
| ('TP53', 'DO50355') |
| ('TP53', 'DO50357') |
| ('TP53', 'DO50362') |
| ('TP53', 'DO50364') |
| ('TP53', 'DO50370') |
| ('TP53', 'DO50372') |
| ('TP53', 'DO50381') |
| ('TP53', 'DO50383') |
| ('TP53', 'DO50385') |
| ('TP53', 'DO50387') |
| ('TP53', 'DO50388') |
| ('TP53', 'DO50389') |
| ('TP53', 'DO50390') |
| ('TP53', 'DO50393') |
| ('TP53', 'DO50398') |
| ('TP53', 'DO50401') |
| ('TP53', 'DO50406') |
| ('TP53', 'DO50407') |
| ('TP53', 'DO50408') |
| ('TP53', 'DO50409') |
| ('TP53', 'DO50411') |
| ('TP53', 'DO50412') |
| ('TP53', 'DO50430') |
| ('TP53', 'DO50434') |
| ('TP53', 'DO50437') |
| ('TP53', 'DO50438') |
| ('TP53', 'DO50439') |
| ('TP53', 'DO50440') |
| ('TP53', 'DO50442') |
| ('TP53', 'DO50443') |
| ('TP53', 'DO50444') |
| ('TP53', 'DO50445') |
| ('TP53', 'DO50446') |
| ('TP53', 'DO50448') |
| ('TP53', 'DO50449') |
| ('TP53', 'DO50453') |
| ('TP53', 'DO50454') |
| ('TP53', 'DO50778') |
| ('TP53', 'DO50791') |
| ('TP53', 'DO50799') |
| ('TP53', 'DO50806') |
| ('TP53', 'DO50815') |
| ('TP53', 'DO50820') |
| ('TP53', 'DO50829') |
| ('TP53', 'DO50832') |
| ('TP53', 'DO50844') |
| ('TP53', 'DO50850') |
| ('TP53', 'DO51046') |
| ('TP53', 'DO51070') |
| ('TP53', 'DO51074') |
| ('TP53', 'DO51087') |
| ('TP53', 'DO51103') |
| ('TP53', 'DO51128') |
| ('TP53', 'DO51137') |
| ('TP53', 'DO51466') |
| ('TP53', 'DO51467') |
| ('TP53', 'DO51470') |
| ('TP53', 'DO51475') |
| ('TP53', 'DO51476') |
| ('TP53', 'DO51478') |
| ('TP53', 'DO51479') |
| ('TP53', 'DO51480') |
| ('TP53', 'DO51481') |
| ('TP53', 'DO51483') |
| ('TP53', 'DO51484') |
| ('TP53', 'DO51485') |
| ('TP53', 'DO51488') |
| ('TP53', 'DO51489') |
| ('TP53', 'DO51490') |
| ('TP53', 'DO51493') |
| ('TP53', 'DO51495') |
| ('TP53', 'DO51497') |
| ('TP53', 'DO51498') |
| ('TP53', 'DO51501') |
| ('TP53', 'DO51504') |
| ('TP53', 'DO51505') |
| ('TP53', 'DO51506') |
| ('TP53', 'DO51509') |
| ('TP53', 'DO51511') |
| ('TP53', 'DO51512') |
| ('TP53', 'DO51515') |
| ('TP53', 'DO51518') |
| ('TP53', 'DO51523') |
| ('TP53', 'DO51529') |
| ('TP53', 'DO51532') |
| ('TP53', 'DO51533') |
| ('TP53', 'DO51535') |
| ('TP53', 'DO51536') |
| ('TP53', 'DO51537') |
| ('TP53', 'DO51538') |
| ('TP53', 'DO51540') |
| ('TP53', 'DO51542') |
| ('TP53', 'DO51543') |
| ('TP53', 'DO51544') |
| ('TP53', 'DO51958') |
| ('TP53', 'DO51959') |
| ('TP53', 'DO51960') |
| ('TP53', 'DO52124') |
| ('TP53', 'DO52161') |
| ('TP53', 'DO52506') |
| ('TP53', 'DO52516') |
| ('TP53', 'DO52538') |
| ('TP53', 'DO52543') |
| ('TP53', 'DO52545') |
| ('TP53', 'DO52546') |
| ('TP53', 'DO52547') |
| ('TP53', 'DO52549') |
| ('TP53', 'DO52550') |
| ('TP53', 'DO52553') |
| ('TP53', 'DO52555') |
| ('TP53', 'DO52556') |
| ('TP53', 'DO52557') |
| ('TP53', 'DO52561') |
| ('TP53', 'DO52564') |
| ('TP53', 'DO52574') |
| ('TP53', 'DO52576') |
| ('TP53', 'DO52582') |
| ('TP53', 'DO52594') |
| ('TP53', 'DO52597') |
| ('TP53', 'DO52605') |
| ('TP53', 'DO52610') |
| ('TP53', 'DO52664') |
| ('TP53', 'DO52677') |
| ('TP53', 'DO52696') |
| ('TP53', 'DO52710') |
| ('TP53', 'DO52717') |
| ('TP53', 'DO52738') |
| ('TP53', 'DO52752') |
| ('TP53', 'DO6398') |
| ('TP53', 'DO7166') |
| ('TPT1-AS1', 'DO52510') |
| ('TPTE2P1', 'DO51954') |
| ('TPTE2P1', 'DO52736') |
| ('TRAPPC3', 'DO52510') |
| ('TRAPPC3L', 'DO51964') |
| ('TRAV27', 'DO51954') |
| ('TRAV27', 'DO51964') |
| ('TRBV28', 'DO49454') |
| ('TRBV6-5', 'DO52510') |
| ('TRBV6-8', 'DO51964') |
| ('TREML3P', 'DO49442') |
| ('TRGC2', 'DO52743') |
| ('TRIM3', 'DO49454') |
| ('TRIM33', 'DO51964') |
| ('TRIM69', 'DO51964') |
| ('TRPC5', 'DO52736') |
| ('TRPM1', 'DO220892') |
| ('TRPM6', 'DO49454') |
| ('TRRAP', 'DO220907') |
| ('TRRAP', 'DO220908') |
| ('TRRAP', 'DO45287') |
| ('TRRAP', 'DO48888') |
| ('TSC1', 'DO52763') |
| ('TSPAN32', 'DO49454') |
| ('TSSC2', 'DO52510') |
| ('TTN', 'DO51954') |
| ('TYW1B', 'DO49454') |
| ('TYW1B', 'DO51954') |
| ('U2AF1', 'DO218139') |
| ('U2AF1', 'DO50832') |
| ('U2AF1', 'DO51481') |
| ('U2AF1', 'DO51485') |
| ('U2AF1', 'DO52747') |
| ('U3', 'DO49454') |
| ('U3', 'DO51964') |
| ('U8', 'DO51964') |
| ('UBE2Q2', 'DO51964') |
| ('UCHL1-AS1', 'DO52743') |
| ('UCK1', 'DO49442') |
| ('UFL1-AS1', 'DO51964') |
| ('UGDH-AS1', 'DO49442') |
| ('UHRF1', 'DO49442') |
| ('UMODL1', 'DO51964') |
| ('UNC5B', 'DO49442') |
| ('UNG', 'DO51964') |
| ('URB1', 'DO51964') |
| ('USP15', 'DO51954') |
| ('USP2-AS1', 'DO49454') |
| ('USP45', 'DO217850') |
| ('USP6', 'DO1002') |
| ('USP6', 'DO218075') |
| ('USP6', 'DO220903') |
| ('USP6', 'DO50410') |
| ('USP8', 'DO51965') |
| ('UVRAG', 'DO51964') |
| ('VAV1', 'DO51959') |
| ('VHL', 'DO46832') |
| ('VHL', 'DO46885') |
| ('VHL', 'DO46909') |
| ('VHL', 'DO46917') |
| ('VHL', 'DO47016') |
| ('VHL', 'DO47068') |
| ('VHL', 'DO47165') |
| ('VNN2', 'DO51964') |
| ('VPS37A', 'DO49442') |
| ('VSTM4', 'DO51964') |
| ('VTI1A', 'DO218693') |
| ('VTI1A', 'DO52675') |
| ('VTI1B', 'DO51964') |
| ('VWA3B', 'DO52736') |
| ('WDR11-AS1', 'DO49442') |
| ('WDR5B', 'DO51964') |
| ('WDR74', 'DO51954') |
| ('WDR83OS', 'DO49454') |
| ('WIF1', 'DO27799') |
| ('WIF1', 'DO50436') |
| ('WNK2', 'DO52512') |
| ('WSCD1', 'DO49454') |
| ('WWTR1', 'DO45299') |
| ('WWTR1', 'DO52704') |
| ('XKR8', 'DO49454') |
| ('XPO1', 'DO27833') |
| ('XPO1', 'DO52679') |
| ('XPO1', 'DO52706') |
| ('XPO1', 'DO6364') |
| ('XRCC5', 'DO51954') |
| ('Y_RNA', 'DO49442') |
| ('Y_RNA', 'DO49454') |
| ('Y_RNA', 'DO51954') |
| ('Y_RNA', 'DO51964') |
| ('Y_RNA', 'DO52510') |
| ('Y_RNA', 'DO52737') |
| ('Z93241.1', 'DO49442') |
| ('Z95704.4', 'DO51964') |
| ('ZBED3-AS1', 'DO51964') |
| ('ZBED5-AS1', 'DO51964') |
| ('ZBTB38', 'DO51964') |
| ('ZBTB7C', 'DO52736') |
| ('ZC3H11A', 'DO49442') |
| ('ZCCHC11', 'DO52736') |
| ('ZCCHC4', 'DO52512') |
| ('ZCCHC8', 'DO36223') |
| ('ZFHX3', 'DO220910') |
| ('ZFHX3', 'DO27767') |
| ('ZFHX3', 'DO27857') |
| ('ZFHX3', 'DO46877') |
| ('ZFHX3', 'DO49113') |
| ('ZFHX3', 'DO51475') |
| ('ZFHX3', 'DO51538') |
| ('ZFHX3', 'DO51959') |
| ('ZFHX3', 'DO52664') |
| ('ZFHX4', 'DO49454') |
| ('ZFHX4-AS1', 'DO49454') |
| ('ZFHX4-AS1', 'DO51954') |
| ('ZFHX4-AS1', 'DO51964') |
| ('ZFYVE21', 'DO51954') |
| ('ZNF106', 'DO51964') |
| ('ZNF20', 'DO52736') |
| ('ZNF207', 'DO51954') |
| ('ZNF222', 'DO52736') |
| ('ZNF341', 'DO51964') |
| ('ZNF37BP', 'DO52510') |
| ('ZNF384', 'DO52513') |
| ('ZNF521', 'DO34448') |
| ('ZNF521', 'DO50342') |
| ('ZNF527', 'DO51954') |
| ('ZNF665', 'DO51954') |
| ('ZNF75D', 'DO49442') |
| ('ZNF815P', 'DO51964') |
| ('ZNF827', 'DO51964') |
| ('ZNF844', 'DO51954') |
| ('ZNRD1-AS1', 'DO51954') |
| ('ZPLD1', 'DO46402') |
| ('ZRANB2-AS1', 'DO51954') |
| ('ZRANB2-AS2', 'DO51954') |
| ('hsa-mir-490', 'DO49454') |
| ('hsa-mir-490', 'DO51954') |
| ('snoU13', 'DO49454') |
| ('snoU13', 'DO51954') |
| ('snoU13', 'DO51964') |
| ('snoU13', 'DO52736') |
| ('snoU13', 'DO52743') |

**Supplementary Table 7.** Edge weights > 2 in one mode gene projections for sample gene bipartite network for each cancer type. Some cancers did not have edges with weight > 1 in their gene projections and hence are not presented in this table.

| **Gene1** | **Gene2** | **weight** | **Tissue** |
| --- | --- | --- | --- |
| TP53 | PIK3CA | 3 | Breast |
| SF3B1 | PIK3CA | 3 | Breast |
| PTEN | PIK3CA | 3 | Breast |
| PIK3CA | GATA3 | 6 | Breast |
| ERBB2 | TP53 | 3 | Eso |
| PIK3CA | TP53 | 3 | Eso |
| CDKN2A | TP53 | 3 | Eso |
| TP53 | GNAS | 3 | Eso |
| NRG1 | TP53 | 4 | Eso |
| TP53 | SMAD4 | 4 | Eso |
| TP53 | ARID1A | 4 | Eso |
| APC | TP53 | 5 | Eso |
| PIK3CA | CTNNB1 | 3 | Liver |
| TP53 | CTNNB1 | 8 | Liver |
| BCL6 | PIM1 | 3 | Lymph |
| SGK1 | BCL2 | 3 | Lymph |
| TP53 | MYC | 3 | Lymph |
| ID3 | MYC | 3 | Lymph |
| B2M | BCL2 | 3 | Lymph |
| PIM1 | SOCS1 | 3 | Lymph |
| MYD88 | BCL2 | 3 | Lymph |
| CARD11 | BCL2 | 3 | Lymph |
| TMSB4X | BTG1 | 4 | Lymph |
| TP53 | SMARCA4 | 4 | Lymph |
| BCL6 | SGK1 | 4 | Lymph |
| TMSB4X | B2M | 4 | Lymph |
| PIM1 | BCL2 | 5 | Lymph |
| PIM1 | SGK1 | 5 | Lymph |
| STAT6 | BCL2 | 5 | Lymph |
| BCL6 | TMSB4X | 5 | Lymph |
| TMSB4X | SOCS1 | 6 | Lymph |
| TMSB4X | BCL2 | 7 | Lymph |
| PIM1 | TMSB4X | 8 | Lymph |
| TMSB4X | SGK1 | 9 | Lymph |
| EZH2 | BCL2 | 9 | Lymph |
| CREBBP | BCL2 | 10 | Lymph |
| GNAS | TP53 | 3 | Panc |
| ZFHX3 | KRAS | 3 | Panc |
| TP53 | FAT4 | 3 | Panc |
| GNAS | KRAS | 4 | Panc |
| SMAD4 | TP53 | 4 | Panc |
| KRAS | SF3B1 | 4 | Panc |
| SMAD4 | KRAS | 9 | Panc |
| CDKN2A | TP53 | 10 | Panc |
| CDKN2A | KRAS | 18 | Panc |
| KRAS | TP53 | 102 | Panc |
| RP11-17E2.2 | CTD-3006G17.2 | 3 | Prost |
| CTD-3006G17.2 | MEF2C-AS1 | 3 | Prost |
| RP11-586K2.1 | Y_RNA | 3 | Prost |
| RP11-586K2.1 | MEF2C-AS1 | 3 | Prost |
| RP11-586K2.1 | CTD-3006G17.2 | 3 | Prost |
| RP11-32K4.1 | RP11-586K2.1 | 3 | Prost |
| RP11-32K4.1 | Y_RNA | 3 | Prost |
| RP11-32K4.1 | MEF2C-AS1 | 3 | Prost |
| RP11-32K4.1 | CTD-3006G17.2 | 3 | Prost |
| CTD-3006G17.2 | Y_RNA | 3 | Prost |
| AC079610.1 | RP11-32K4.1 | 3 | Prost |
| AC079610.1 | Y_RNA | 3 | Prost |
| AC079610.1 | MEF2C-AS1 | 3 | Prost |
| AC079610.1 | CTD-3006G17.2 | 3 | Prost |
| RP11-17E2.2 | RP11-32K4.1 | 3 | Prost |
| RP11-17E2.2 | RP11-586K2.1 | 3 | Prost |
| RP11-17E2.2 | AC079610.1 | 3 | Prost |
| RP11-17E2.2 | Y_RNA | 3 | Prost |
| RP11-17E2.2 | MEF2C-AS1 | 3 | Prost |
| AC079610.1 | RP11-586K2.1 | 3 | Prost |
| MEF2C-AS1 | Y_RNA | 3 | Prost |
| GRIN2A | TP53 | 3 | Skin |
| MUC16 | PTEN | 3 | Skin |
| TCL1A | TP53 | 3 | Skin |
| CTNNA2 | PTPRT | 3 | Skin |
| PBX1 | PTPRT | 3 | Skin |
| CDH10 | MUC16 | 3 | Skin |
| CCND2 | MUC16 | 3 | Skin |
| PBX1 | CDKN2A | 3 | Skin |
| PREX2 | BRAF | 3 | Skin |
| GRIN2A | CDKN2A | 3 | Skin |
| TCL1A | CDKN2A | 3 | Skin |
| ERBB4 | MECOM | 3 | Skin |
| TP53 | CDKN2A | 3 | Skin |
| TP53 | BRAF | 3 | Skin |
| ERBB4 | RUNX1T1 | 3 | Skin |
| ERBB4 | CNTNAP2 | 3 | Skin |
| PTPRB | BRAF | 3 | Skin |
| ERBB4 | NRAS | 3 | Skin |
| ERBB4 | MUC16 | 3 | Skin |
| CNTNAP2 | BRAF | 4 | Skin |
| MUC16 | PREX2 | 4 | Skin |
| MUC16 | TCL1A | 4 | Skin |
| MUC16 | PTPRB | 4 | Skin |
| MUC16 | CDKN2A | 4 | Skin |
| CSMD3 | PTPRT | 4 | Skin |
| ERBB4 | CSMD3 | 4 | Skin |
| GRIN2A | PTPRT | 4 | Skin |
| MUC16 | PBX1 | 4 | Skin |
| CDKN2A | BRAF | 4 | Skin |
| TP53 | PTPRT | 5 | Skin |
| ERBB4 | PTPRT | 5 | Skin |
| GRIN2A | MUC16 | 5 | Skin |
| MUC16 | TP53 | 6 | Skin |
| CNTNAP2 | MUC16 | 6 | Skin |
| CNTNAP2 | PTPRT | 6 | Skin |
| NRAS | MUC16 | 6 | Skin |
| ERBB4 | BRAF | 6 | Skin |
| PTPRT | BRAF | 7 | Skin |
| MUC16 | BRAF | 7 | Skin |
| MUC16 | PTPRT | 9 | Skin |
